# Aminothiazolone Inhibitors Disrupt the Protein–RNA Interaction of METTL16 and Modulate the m^6^A RNA Modification

**DOI:** 10.1021/jacsau.3c00832

**Published:** 2024-03-21

**Authors:** Yang Liu, Georg L. Goebel, Laurin Kanis, Oguz Hastürk, Claus Kemker, Peng Wu

**Affiliations:** † Chemical Genomics Centre, Max Planck Institute of Molecular Physiology, Dortmund 44227, Germany; ‡ Department of Chemical Biology, Max Planck Institute of Molecular Physiology, Dortmund 44227, Germany; § Faculty of Chemistry and Chemical Biology, TU Dortmund University, Dortmund 44227, Germany

**Keywords:** RNA modification, RNA-binding protein, post-transcriptional regulation, protein-RNA interaction, small-molecule inhibitor

## Abstract

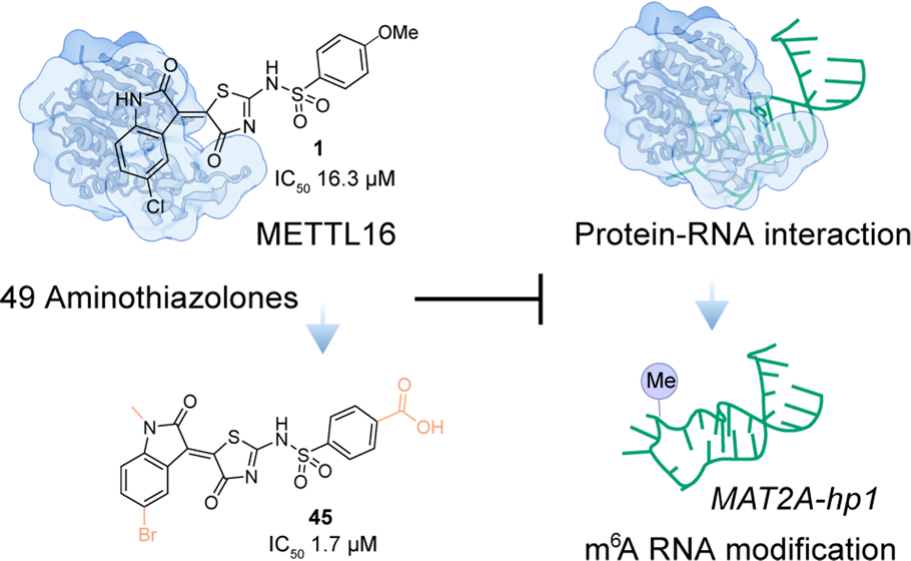

Targeting RNA-binding and modifying proteins via small molecules to modulate post-transcriptional modifications have emerged as a new frontier for chemical biology and therapeutic research. One such RNA-binding protein that regulates the most prevalent eukaryotic RNA modification, *N*^6^-methyladenosine (m^6^A), is the methyltransferase-like protein 16 (METTL16), which plays an oncogenic role in cancers by cofunctioning with other nucleic acid-binding proteins. To date, no potent small-molecule inhibitor of METTL16 or modulator interfering with the METTL16–RNA interaction has been reported and validated, highlighting the unmet need to develop such small molecules to investigate the METTL16-involved regulatory network. Herein, we described the identification of a series of first-in-class aminothiazolone METTL16 inhibitors via a discovery pipeline that started with a fluorescence-polarization (FP)-based screening. Structural optimization of the initial hit yielded inhibitors, such as compound **45**, that showed potent single-digit micromolar inhibition activity against the METTL16-RNA binding. The identified aminothiazolone inhibitors can be useful probes to elucidate the biological function of METTL16 upon perturbation and evaluate the therapeutic potential of METTL16 inhibition via small molecules at the post-transcriptional level.

## Introduction

**Figure 1:**
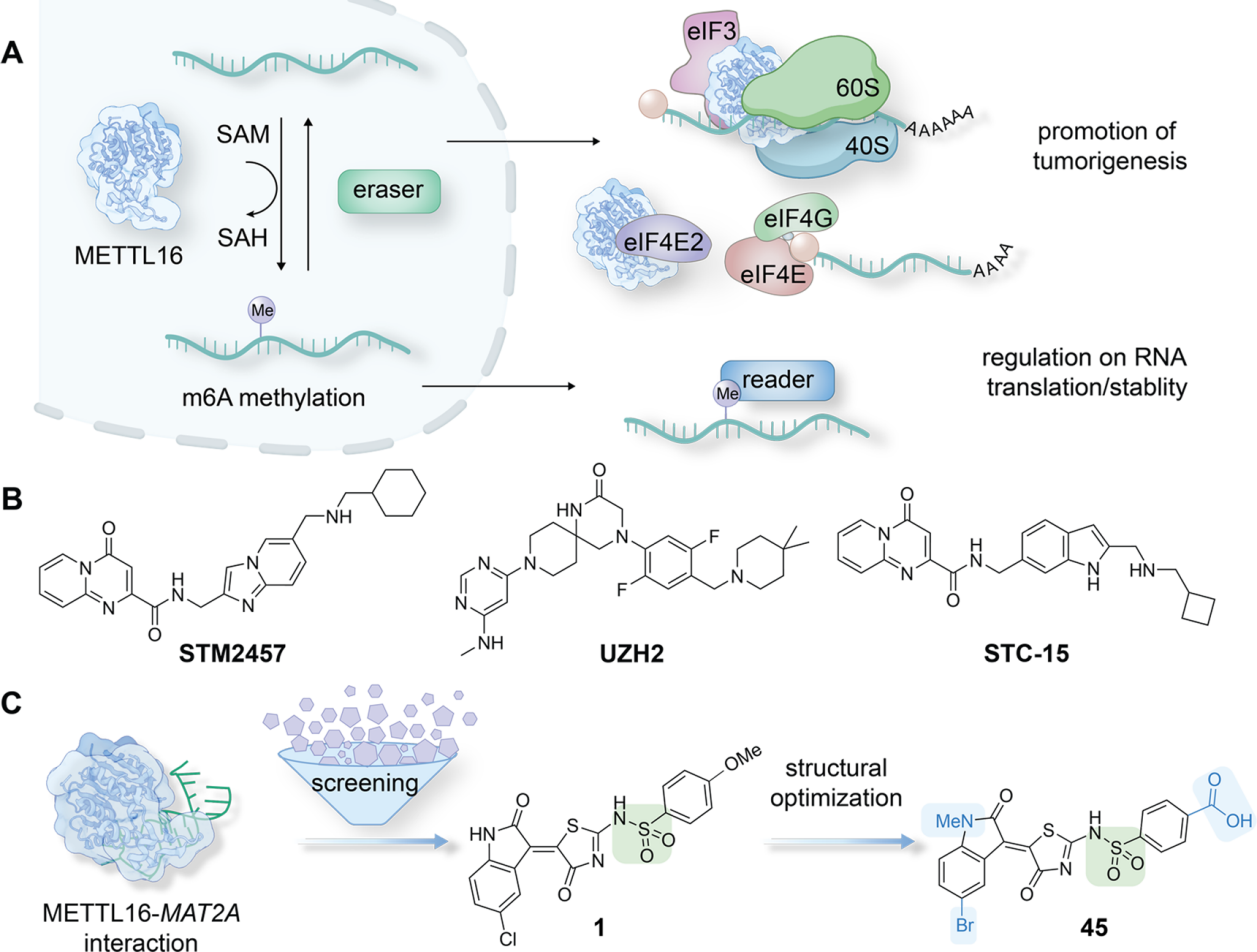
Development of the small-molecule inhibitors of METTL16. (A) A simplified illustration of METTL16-involved pathways. METTL16 installs m^6^A to specific RNA substrates in the nucleus, leading to the recognition of m^6^A readers. METTL16 interacts with eukaryotic initiation factors in the cytosol to promote the translation of mRNAs. (B) Reported anticancer small-molecule inhibitors targeting another human methyl transferase, METTL3. (C) Workflow of this study: screening of a compound collection resulted in the identification of compound **1**. Subsequent structure–activity relationship investigations led to compound **45** as the most promising METTL16 inhibitor, showing low micromolar potency.

RNAs are effector molecules and intermediates in protein synthesis and also directly influence gene expression, processing, and stability at the transcription levels regulated by diverse chemical modifications.^1−3^ Among the plethora of identified RNA modifications is the methylation at the *N*^6^ position of adenosine (m^6^A), which is the most abundant RNA modification in eukaryotes.^4^ The dynamic m^6^A modification is tightly controlled by a suite of RNA-binding and/or modifying proteins, including the deposition by the so-called m^6^A ‘writers’, recognized by m^6^A ‘readers’, and removed by m^6^A ‘erasers’.^5^ To date, the identified m^6^A writers include the methyltransferase-like proteins 3 and 14 complex (METTL3/14),^6^ METTL16,^7−11^ METTL5–tRNA methyltransferase 112 complex (TRMT112),^12^ and zinc-finger CCHC domain-containing protein 4 (ZCCHC4).^13,14^ The heterodimeric m^6^A writer complex of METTL3/14 cotranscriptionally installs the modification on transcripts with a conserved RRA(m^6^A)CH motif (*R*=A or G; H=A, C, or U)^6^; in comparison, METTL16 was found to methylate a selected number of noncoding RNAs, including U6 small nuclear RNA (snRNA) and mRNA (*MAT*2*A*), with the UACA(m^6^A)GAGAA consensus sequence and a specific secondary structure.^7,9,11,15^ METTL16 is essential for mammalian cell viability and embryonic development,^7,16^ as demonstrated in METTL16-knockout studies in mouse embryos.^8^ In the nucleus, METTL16 mediates the methylation of the *MAT2A* mRNA that encodes for the rate-limiting *S*-adenosylmethionine (SAM) synthetase MAT2A, regulating cellular SAM homeostasis.^7^ Besides the methyltransferase activity in the nucleus, it was demonstrated that METTL16 is preferentially distributed in the cytosol where it interacts with eukaryotic initiation factors (eIFs), eIF3a/b and eIF4E2, thereby promoting translation of thousands of mRNA transcripts and facilitates oncogenic protein synthesis (Figure 1A).^17,18^ In addition to the gene regulatory function, the interaction between METTL16 and the triple helix of *MALAT1* may contribute to the oncogenic activity of the lncRNA.^19^ Besides *MALAT1* RNA, METTL16 putatively binds toward diverse cellular RNA substrates, the function of which is yet to be determined.^11^ Following the establishment of the association between m^6^A-binding and modifying proteins and human cancers in recent years,^20−22^ inhibitors targeting the m^6^A writer METTL3 have been reported, of which the METTL3 inhibitor STC-15 is the first inhibitor being progressed into clinical trials, for the treatment of advanced malignancies (NCT05584111) (Figure 1B).^23,24^ In parallel, small molecules targeting both disease-associated RNAs^25−32^ and other RNA-binding proteins (RBPs) demonstrated the resilience in addressing the post-transcriptional modifications from different angles.^33,34^

A recent study showed that METTL16 is one of the most important genes for various cancer cell survivability, especially among the human methyltransferase family,^17^ suggesting the promising potential of METTL16 inhibition via small molecules as a new anticancer strategy. However, no such small molecules targeting METTL16 have been reported yet, nor has there been an efficient discovery approach. In this study, we established a screening method for the identification of small molecules that disrupt the METTL16–*MAT2A-*hp1 mRNA interaction. Application of the screening assay led to the discovery of aminothiazolones that were characterized as first-in-class METTL16 inhibitors (Figure 1C). Furthermore, through the collective synthesis of 49 analogues, we presented here the first and only systematic structure–activity relationship associated with METTL6-targeting small molecules. The identified METTL16 inhibitors can be useful probes to unravel unknown functions of METTL16 and set the foundation for the development of therapeutic agents disrupting the function of METTL16.

## Results and Discussion

### Aminothiazolone Hit as METTL16 Inhibitors

**Figure 2:**
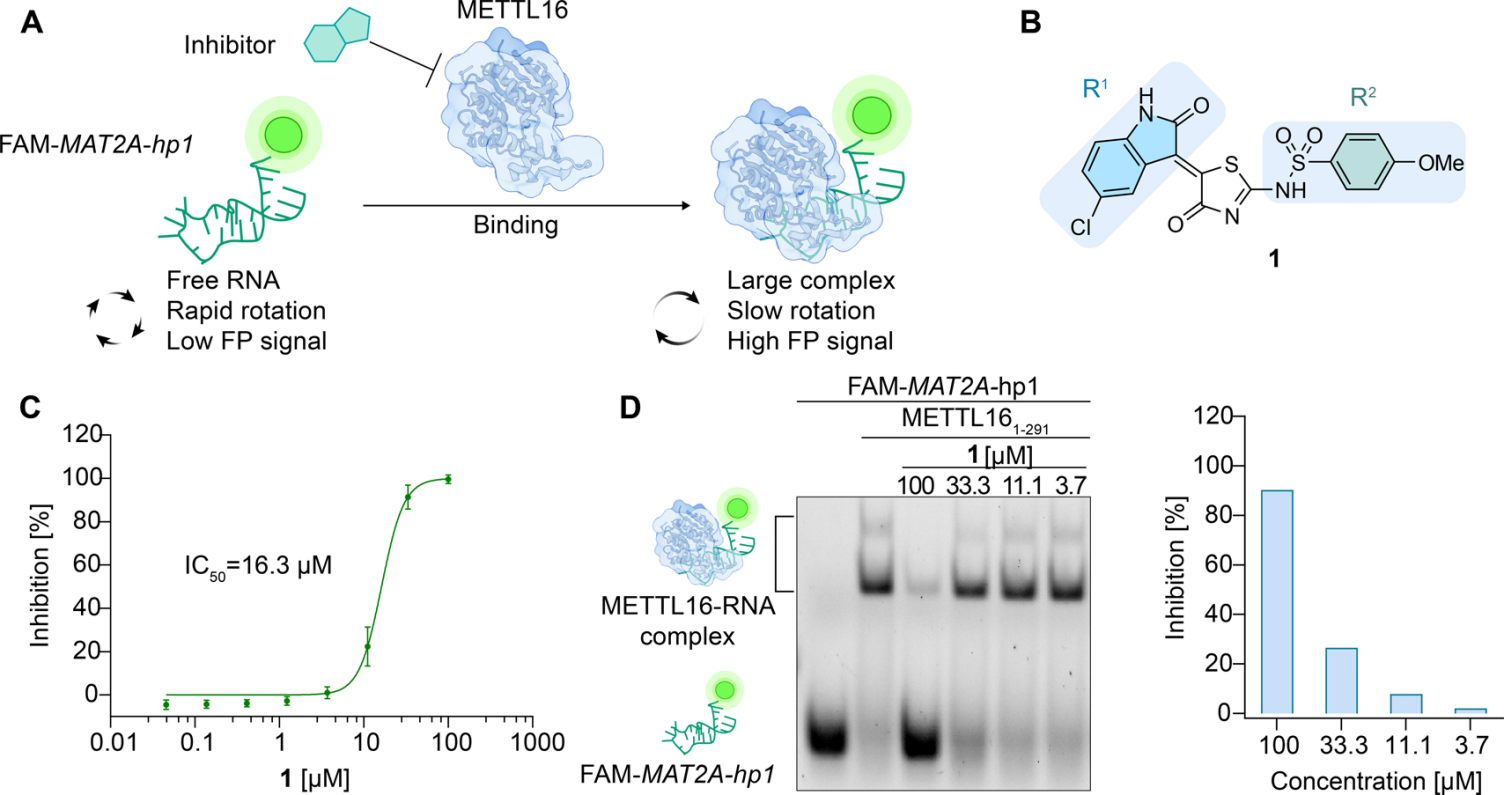
Identification of the aminothiazolones as METTL16 inhibitors. (A) The established FP assay measures the disruption of the interaction between the METTL16 and RNA interaction. Small-molecule inhibitors disrupted the interaction, leading to a weak FP signal. (B) The identified hit compound **1**. (C) **1** inhibited METTL16–RNA interaction with an IC_50_ of 16.3 μM in the FP assay with FAM-labeled *MAT2A*-hp1 RNA. Data are shown as mean ± SEM, *n* = 4. (D) **1** disrupted the METTL16–RNA interaction in the EMSA assay in a dose-dependent manner.

To enable an efficient discovery approach for the discovery of small-molecule modulators targeting the METTL16–*MAT2A* mRNA interaction, we started with the establishment of a fluorescence polarization (FP) assay using an FAM-labeled *MAT2A-*hp1 RNA probe (FAM-CUUGUUGGCGU AGGCUACAGAGAAGCCUUCAAG) and the methyltransferase domain (MTD) of human METTL16 (1–291) (Figures 2A and S1A–C). The binding of METTL16 to the FAM-labeled RNA probe formed a complex that led to a higher FP signal in comparison to that of the unbound RNA probe (Figure 2A). Titrations of FAM-labeled RNA concentrations and protein concentrations were performed to retrieve the optimal assay condition, which was revealed to be 80 nM METTL16 and 2 nM FAM-*MAT2A* RNA, together with the assessment of the varied incubation time (Figure S2A–C). The Z-factor of 0.91 indicated the suitability of the assay condition (Figure S2D). The unlabeled *MAT2A-*hp1 RNA was used as a control inhibitor, which disrupted the interaction between METTL16 and the FAM-labeled *MAT2A-*hp1 RNA in a dose-dependent manner with an IC_50_ value of 60.4 nM (Figure S2E), while the reported affinity of *MAT2A*-hp1 toward METTL16 MTD is 110 nM (*K*_D_ value).^35^ An in-house compound library containing ∼25000 small molecules was then screened using the established FP assay to identify METTL16 inhibitors (Figure S2F). Among the revealed hits was (*Z*)-5-(2-oxoindolin-3-ylidene)thiazol-4(5H)-one compound **1** that showed an IC_50_ of 16.3 μM in the FP assay (Figure 2B,C). A subsequent electrophoretic mobility shift assay (EMSA) validated the dose-dependent inhibition of compound **1** disrupting the binding between METTL16 and the *MAT2A*-hp1 RNA (Figure 2D). In both FP assay and EMSA, a final concentration of 50 nM METTL16 and 5 nM FAM-*MAT2A*-hp was used. The METTL16 MTD (amino acids 1–291) was used in this study unless specifically indicated otherwise.

### Structural Optimization Based on Hit Compound 1

**Figure 3:**
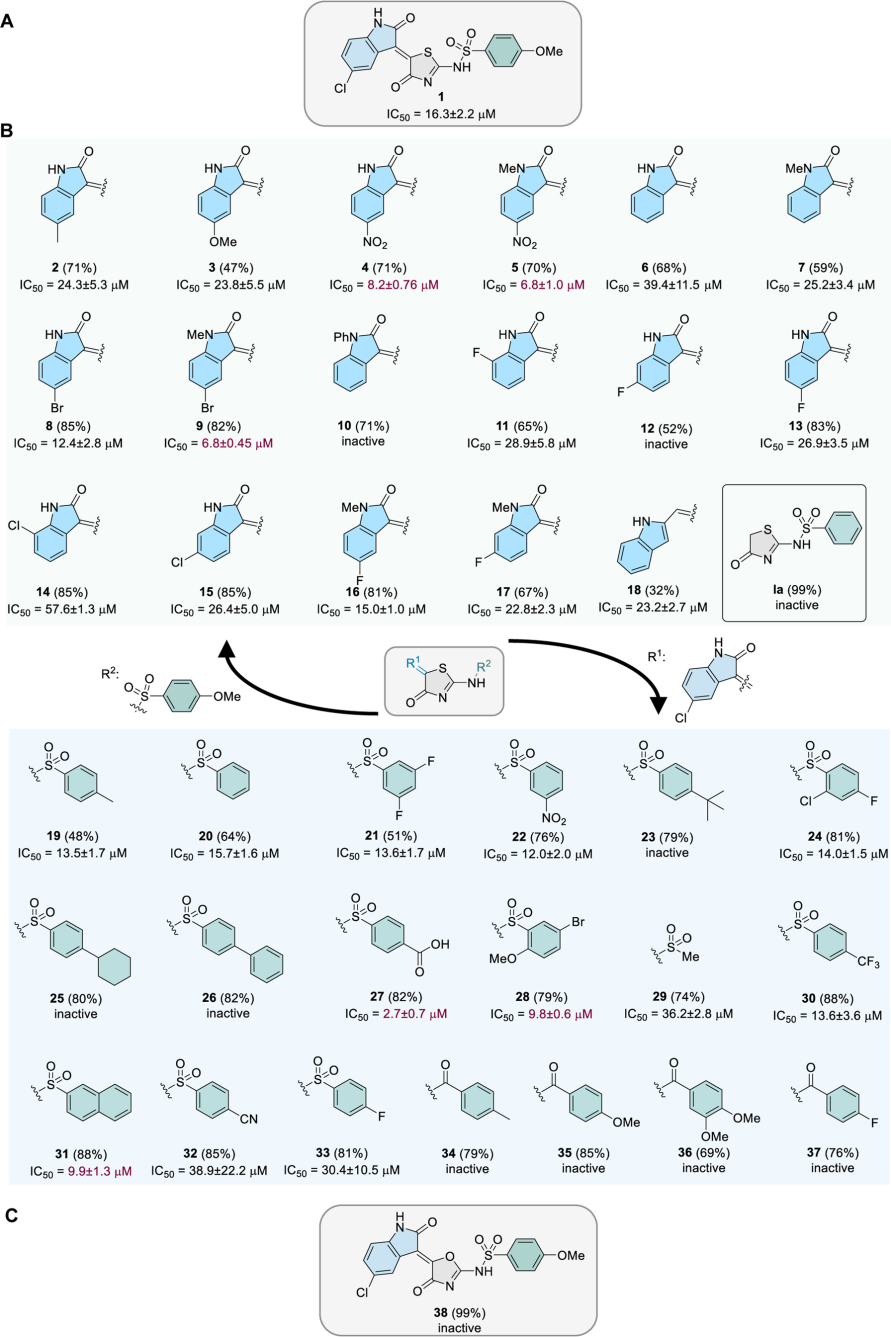
Small-molecule aminothiazolone analogues **1**–**38** were evaluated in this study. (A) The aminothiazolone hit **1** was identified via a FP-based screening. (B) Aminothiazolone analogues **2**–**37** with structural modifications in the oxindole (R^1^) and sulfonamide groups (R^2^). (C) The inactive aminooxazolone analogue was **38**. Isolated yields are shown in brackets; ‘red font’: inhibitory IC_50_ less than 10 μM; ‘inactive’: IC_50_ > 100 μM.

To explore the structural features required to achieve potent METTL16 inhibition surrounding the (*Z*)-5-(2-oxoindolin-3-ylidene)thiazol-4(5*H*)-one sulfonamide scaffold of compound **1** (Figure 3A), we performed structural modifications on the oxindole (R^1^) and sulfonamide (R^2^) moieties (Figure 3B), as well as on the core thiazolone scaffold (Figure 3C), which resulted in a collection of 37 derivatives (**2–38**) showing a variety of substituents decorated with electron-donating (**3**, **28**) and electron-withdrawing groups (**4**, **5**, **22**, **27**, **30**, and **32**), hydrophobic and bulky substituents (**10**, **19**, **23**, **25**, **26**, **31**, and **34**) and the introduction of a methyl group at the *N-*1 position of the oxindole moiety (R^1^) (**4**, **7**, **9**, **16**, and **17**), as well as the exchange of the aminothiazolone core with an oxazolone core (**38**) and lead to compounds that showed single-digit micromole IC_50_ against METTL16 (Figures 3 and S3).

Based on the inhibitory data obtained from the FP assay and EMSA, as shown in Figure 3 (IC_50_ values obtained from FP assay), we observed the following trend for the analogues **2**–**38**. Substituent changes on the oxindole group (R^1^) were mostly tolerated to varied extents, such as the addition of a halide group (**8**, **9**, **11**, **12**, **13**, **14**, **15**, **16**, and **17**), hydrophobic residues (**2**), electron-donating (**3**) or electron-withdrawing groups (**4**), and methylation on the *N-*1 position (**5**, **7**, **9**, **16**, and **17**) of the afore-modified oxindole. Compounds **5** and **9** were among the best-performed analogues that showed single-digit micromolar inhibitory potencies. In comparison, the replacement of the oxindole moiety (R^1^) with an indole moiety (**18**) decreased the activity. The introduction of hydrophobic and bulky sulfonamides (R^2^) led to either a decrease or a complete loss of activity (**23**, **25**, and **26**). The carboxylic acid containing analogue **27** showed the most potent inhibition against METTL16 with an IC_50_ of 2.7 μM. The importance of a carboxylic acid group for small-molecule modulators targeting RBPs has been demonstrated in other inhibitors,^36−39^ which can be attributed to the ionic interactions formed between the negatively charged carboxylic acid and the positively charged lysine-rich RNA-binding surface on RBPs. In addition to the above-mentioned modifications, the exchange of the sulfonamide group (R^2^) with an amide group resulted in four amide analogues **34**–**37** that did not show any inhibitory activity against METTL16, demonstrating the importance of the sulfonamide group, which probably functions as a crucial hydrogen-bond acceptor with key residues on METTL16. Additionally, replacing the thiazolone core with an oxazole core resulted in compound **38**, which did not show inhibitory activity.

**Figure 4:**
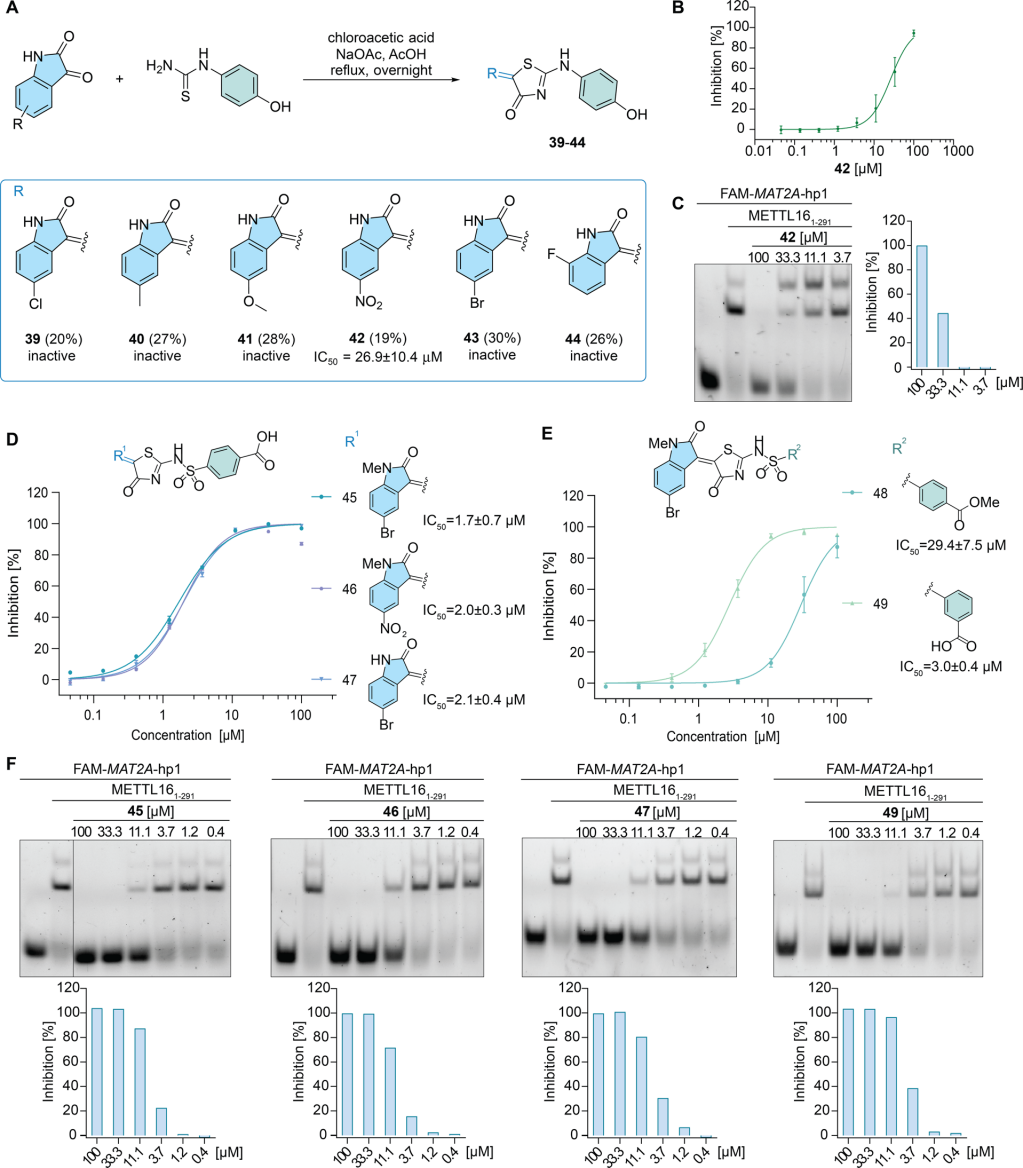
Sulfonamide moiety is critical for the activity, and structural modifications yield potent METTL16 inhibitors. (A) Synthesis route and structures of analogues **39**–**44** without the sulfonamide moiety. (B) FP assay result for compound **42**. Data are shown as mean ± SEM, *n* = 4. (C) EMSA assay result for compound **42**. (D) Combination of the best-performed aminothiazolones with carboxylic acid residue yielded potent inhibitors **45**, **46**, and **47**. Data are shown as mean ± SEM, *n* = 4. (E) Evaluation of carboxylic acid residue and methyl ester analogue **48** decreased the activity, while *meta*-position carboxylic acid analogue **49** maintained the activity. Data are shown as mean ± SEM, *n* = 4. (F) EMSA results and gel quantification data for compounds **45**, **46**, **47**, and **49**.

Furthermore, the impact of the sulfonamide moiety (R^2^) was evaluated by synthesizing and testing the aminothiazolone analogues **39**–**44** that lacked the sulfonamide or amide group (Figure 4A). Most of these compounds did not show any activity, except for compound **42** that showed a decreased inhibitory potency (IC_50_ = 26.9 μM) in comparison with that of compound **1** in the FP assay (Figure 4B,C). Compared to compound **4**, which has the same 5-nitrooxindole moiety (R^1^), the activity of **42** decreased by 3-fold. The observed inhibitory effect could be explained by an interaction of METTL16 with the nitro group of **42**. To visualize a potential binding mode of **42** to METTL16, docking analysis was performed (Figure S4). Generally, the activity loss or reduction of compounds **34**–**37** and **39**–**44** suggested the important role of the sulfonamide group in METTL16 inhibition.

Given the obtained results from the above structural modifications, we proceeded with the synthesis of another collection of modified analogues by combining the key structural features from the best-performed aminothiazolones (**5**, **9**, and **27**), which resulted in analogues **45** to **47** (Figure 4D, F). To our delight, this effort yielded the most potent METTL16 inhibitor **45** with an IC_50_ value of 1.7 μM, together with *N*-methyl-5-nitro containing analogue **46** and the bromo analogue **47** that showed equivalent inhibitory activities with IC_50_ values of 2.0 and 2.1 μM, respectively. To further investigate the impact of the carboxylic acid residue, we evaluated the methyl ester containing analogue **48**, which showed a 20-fold decrease in the inhibitory activity. Given that in a previous study the change of the carboxylic acid group from the *para*- to *meta*-position significantly increased the potency of an RBP inhibitor,^36^ we synthesized the corresponding analogue **49**, which retained but did not show improved inhibition against METTL16 (IC_50_: 3.0 μM) (Figure 4E,F). Collectively, through extensive structural optimization based on the aminothiazolone scaffold of **1**, we identified a series of compounds that showed single-digit micromolar inhibitory potency against METTL16.

### Aminothiazolones Disrupted METTL16–RNA Interaction via METTL16 Binding

**Figure 5:**
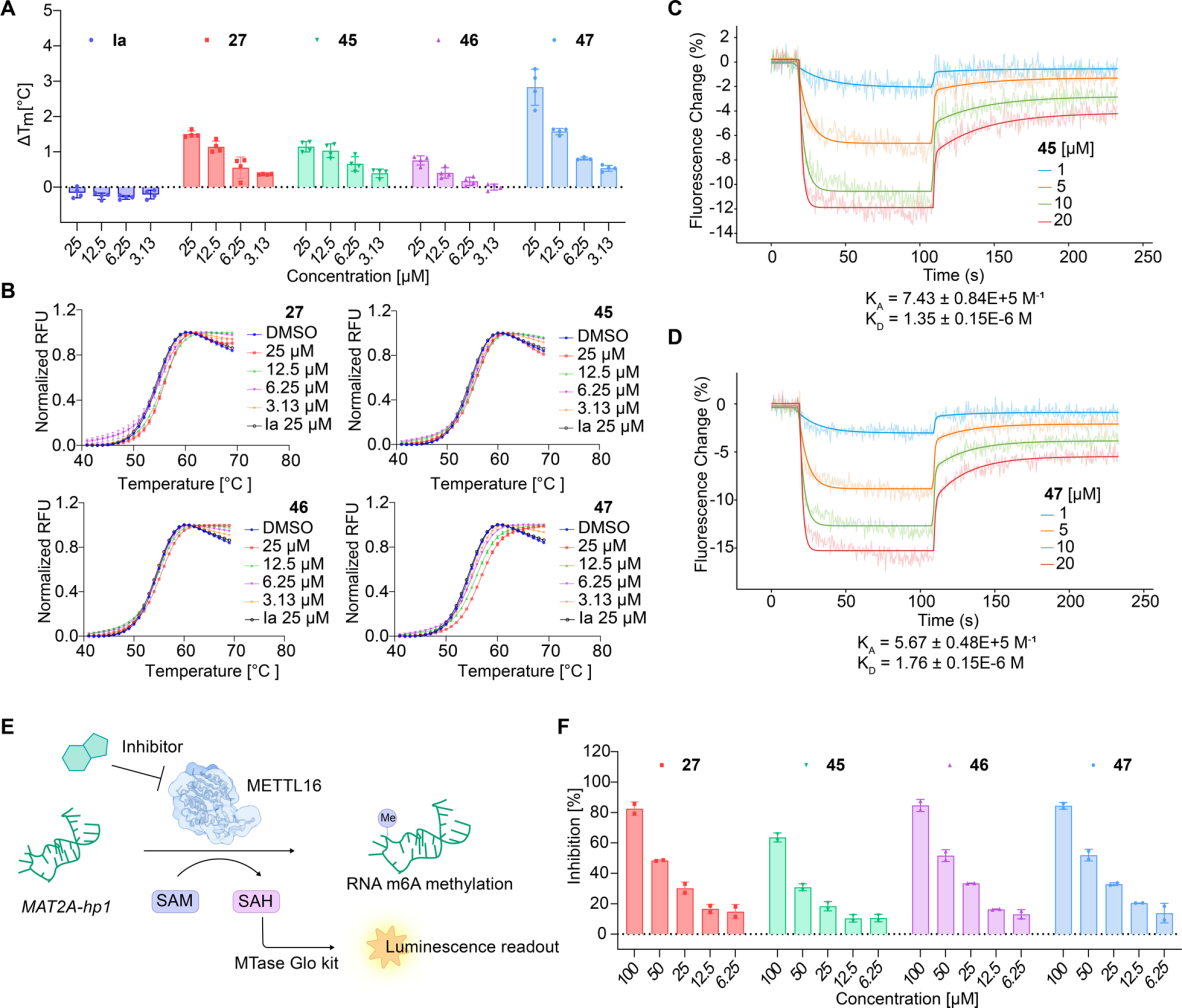
Aminothiazolones bind to METTL16 and inhibit the methyltransferase activity of METTL16. (A) Δ*T*_m_ values of different inhibitors. Data are shown as mean ± SEM, *n* = 4. (B) Denaturation curves of METTL16 with tested compounds. In contrast to the control compound **Ia**, inhibitors **27**, **45**, **46**, and **47**, dose-dependently change the thermal stability of the METTL16 protein. Data are shown as mean ± SD, *n* = 2. (C) and (D) Biosensor assay evaluated the binding affinities of compounds **45** and **47** toward METTL16. (E) Schematic of aminothiazolones inhibiting the methyltransferase activity of METTL16 through disruption of the protein–RNA interaction of METTL16 and *MAT2A-*hp1 mRNA. Inhibitors were preincubated with the METTL16 MTD protein for 30 min, and a mixture of *MAT2A*-hp1 and SAM was added to the reaction. After reacting for 1 h at room temperature, MTase Glo reagents were added, and the luminescence was measured by TECAN. (F) Compounds **27**, **45**, **46**, and **47** dose-dependently inhibited the methyltransferase activity of METTL16. The potassium salt of compound **45** was used in the biosensor assay. Data are shown as mean ± SD, *n* = 2.

To probe the inhibition mechanism of the aminothiazolones toward the METTL16–*MAT2A-*hp1 mRNA interaction, we evaluated the direct binding between the identified inhibitors and METTL16 via differential scanning fluorimetry (DSF).^40^ We measured the thermal stability of METTL16 MTD (the midpoint of the transition, *T*_m_ value) for the four most active compounds (**27**, **45**, **46**, and **47**). Compound **Ia** was used as a negative control (METTL16, IC_50_: > 100 μM) (Figure S5A,B). The DSF result showed that compounds **27**, **45**, **46**, and **47** dose-dependently stabilized METTL16 upon binding (Figures 5A,B and S5D). In comparison, the negative control **Ia** did not show a detectable change in the thermal stability of METTL16 (Figures 5B, S5C). The binding affinities of inhibitors toward METTL16 were evaluated through the switchSENSE biosensor assay; compounds **45** and **47** showed *K*_D_ values of 1.35 and 1.76 μM, respectively (Figure 5C,D). The affinity of compound **47** was further validated via isothermal titration calorimetry (ITC), and a *K*_D_ value of 5.12 μM was obtained (Figure S5E).

Next, we evaluated the inhibition of the METTL16 methylation using the MTase Glo assay kit (Promega) measuring the methyltransferase activity based on the formation of the methylation product *S*-adenosyl homocysteine, which was converted to ADP by the MTase-Glo reagent to trigger a subsequent luciferase reaction (Figure 5E). The result revealed that the aminothiazolones, especially **27**, **46**, and **47**, exhibited potent inhibitory activity against the methyltransferase activity of METTL16 MTD toward the RNA substrate *MAT2A-*hp1 in a dose-dependent manner, e.g., compound **47** exhibited >50% inhibition on methylation at 50 μM (Figure 5F).

### Aminothiazolones Disrupted METTL16 Interaction with Diverse RNA Substrates

**Figure 6:**
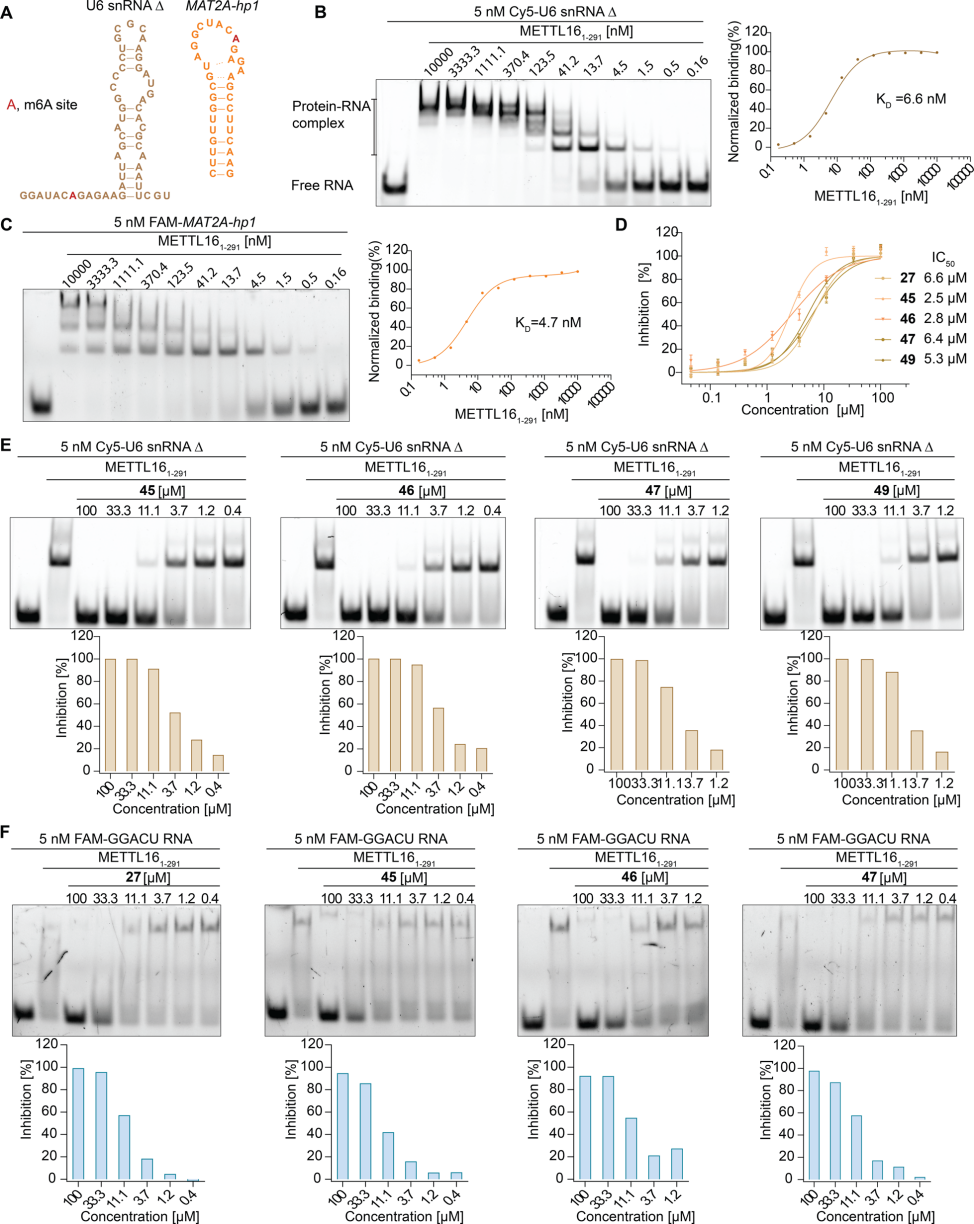
Aminothiazolones disrupted METTL16–RNA interaction for other RNA substrates. (A) Predicted secondary structure of U6 snRNA deletion using RNA structure web service^41^ and secondary structure of *MAT2A*-hp1. (B) METTL16 MTD binds to U6 snRNA Δ in EMSA. (C) METTL16 MTD binds to *MAT2A-*hp1 RNA substrate in EMSA. (D) Compounds **27**, **45**, **46, 47**, and **49** inhibited the binding interaction of U6 snRNA Δ with METTL16 MTD in the FP assay. Data are shown as mean ± SEM, *n* = 4. (E) The EMSA results of **45**, **46, 47**, and **49** with U6 snRNA Δ. (F) The EMSA results of **27**, **45**, **46** and **47** with GGACU-containing RNA substrates.

METTL16 methylates U6 small nuclear RNA (snRNA) in the conserved UACA(m^6^A)GAGAA motif. We evaluated the binding affinity of METTL16 MTD with a U6 snRNA deletion (U6 snRNA Δ), a telestem deletion that has been reported to be methylated by METTL16 MTD (Figures 6A and S6A, U6 snRNA_Δ*T*S3 from reference),^35^ through FP assay and EMSA. As it has been demonstrated that the RNA sequence is methylated by METTL16,^35^ our findings showed similar binding affinities of METTL16 MTD against U6 snRNA Δ and *MAT2A*-hp1. Measured via the EMSA experiment, the *K*_D_ values of U6 snRNA Δ and *MAT2A*-hp1 toward METTL16 MTD were 6.6 and 4.7 nM, respectively (Figure 6B,C). The affinities were measured by titrating the RNA concentrations with 50 nM METTL16 MTD protein using the FP assay as well. Here, the *K*_D_ values were 32.14 nM (U6 snRNA Δ) and 29.42 nM (*MAT2A*-hp1) (Figure S6B,C).

We further evaluated the aminothiazolone inhibitors’ ability to disrupt the interaction between METTL16 and U6 snRNA Δ in the FP assay as well as the EMSA assay, with 5 nM Cy5-labeled U6 snRNA Δ and 50 nM METTL16 MTD protein. In the FP assay, compounds **27**, **45**, **46, 47**, and **49** inhibited the binding interaction with IC_50_ values of 6.6, 2.5, 2.8, 6.4, and 5.3 μM, respectively (Figure 6D), which showed relatively similar inhibitory potency with METTL16 against *MAT2A-*hp1. The EMSA assay further confirmed the inhibition, where compounds **27**, **45**, **46, 47**, and **49** dose-dependently interrupted METTL16 MTD-U6 snRNA Δ interaction (Figures 6E and S6D).

Although METTL16 has been reported as an RBP toward diverse RNA substrates,^11^ it is not clear whether METTL16 can bind GGACU-containing RNA substrate, which is the substrate sequence targeted by the methyltransferase METTL3/14 complex.^6^ In this context, we evaluated the binding between METTL16 and GGACU-containing RNAs during the assay establishment steps for the characterization of the identified small-molecule inhibitors. To our surprise, the METTL16 MTD bound to a GGACU-motif-containing RNA, albeit with a weaker affinity in comparison with that of the binding to the *MAT2A*-hp1 RNA (Figures 6C and S6F). The binding interaction was further confirmed by using the unlabeled GGACU-containing RNA substrate (Figure S6G). The unlabeled GGACU RNA binds to the METLL16 protein, disrupting the interaction between METTL16 and the FAM-labeled *MAT2A-*hp1 RNA with an IC_50_ value of 813.8 nM, whereas unlabeled *MAT2A-*hp1 shows an IC_50_ of 47.2 nM, which confirmed that GGACU-motif-containing RNA bound to METTL16, but with a weaker affinity in comparison with that of the binding to *MAT2A-*hp1 RNA. Naturally, we were curious if the GGACU-RNA’s binding to METTL16 would induce the corresponding methylation activity. A subsequent *in vitro* methylation assay showed that the methylation activity of METTL16 toward GGACU motif-containing RNA was not significantly changed in comparison with the methylation level on the *MAT2A-*hp1 substrate (Figure S6H). The results suggested that METTL16 may function as an RBP without imposing methylation activity on GGACU-containing RNA substrates.

We then evaluated the aminothiazolone inhibitors’ ability to disrupt the protein–RNA interaction between METTL16 and GGACU-containing RNA substrates in EMSA with 5 nM GGCAU-containing RNA and 600 nM METTL16 MTD protein (Figure 6F), and the results showed that inhibitors **27**, **45**, **46**, and **47** dose-dependently inhibited the binding interaction between METTL16 and the GGACU RNA. Additionally, the binding affinity of METTL16 with two precursor microRNAs was tested in the FP assay by using unlabeled precursor microRNA hairpins, including pre-*miR-17-*hp, pre*-miR-21-*hp, and a pre-*miR-17-*hp mutant with two bulges being base-paired (pre-*miR-17-*hp *bp*) (Figure S6I). Both the pre-*miR-17-*hp and pre-*miR-21-*hp showed more potent inhibitory activity than that of the GGACU-motif RNA and the base-paired mutant pre-*miR-17-*hp *bp*. This result indicated that METTL16 may serve as an RBP for a wide range of RNA substrates that harbor secondary structural elements including precursor microRNAs.^11^ In summary, we verified that METTL16 serves as an RBP without imposing a methylation activity for certain RNA substrates. Furthermore, aminothiazolone inhibitors were able to disrupt the METTL16–RNA binding interaction involving different RNA substrates.

### Inhibition Mode via Competing at the *MAT2A-*Hp1 Binding Site

To evaluate whether the aminothiazolones covalently bind to METTL16, we performed the LC–MS analysis after incubating the protein with aminothiazolones for 30 and 150 min, respectively. In the case of covalent binding, we would expect to observe the formation of covalent adducts, which would accrue over time. However, in comparison with the DMSO control (Figure S7A), no such covalent adduct with a mass shift was observed after incubation with compounds **45** (Figure S7B), **46** (Figure S7C), or **47** (Figure S7D) for either 30 or 150 min, indicating that aminothiazolones are not covalent binders to METTL16. Besides the LC–MS analysis result, an irreversible inhibition counter screening indicated the reversible inhibitory activity of the aminothiazolones (Figure S8A).

FP and EMSA assays indicated that the aminothiazolones disrupted METTL16-RNA binding. Therefore, we hypothesized that these compounds bind to the RNA-binding pocket of METTL16 and competed with the RNA substrate; however, compounds that bind to the SAM pocket with an extended part reaching the RNA pocket would also present such an effect by competing with both RNA and SAM. To exclude this possibility, different concentrations of SAM were incubated with METTL16 before the FP assay. If the presence of SAM competes with aminothiazolones, then the inhibitory activity of aminothiazolones will decrease. As expected, the presence of SAM did not have any impact on the compounds’ activity, and the IC_50_ value of compounds **45**, **46**, and **47** remained under different SAM concentrations (Figure S8B). Compound **47** showed a noncompetitive inhibition mode with SAM from a Michaelis–Menten kinetics study through an *in vitro* methylation assay as well (Figure S8C). However, other binding pockets, e.g., allosteric binding sites that are not the SAM- or RNA-binding sites, would also be possible. Compounds bound to those pockets will change the conformation of METTL16 and thereby inhibit the RNA-binding interaction.

**Figure 7:**
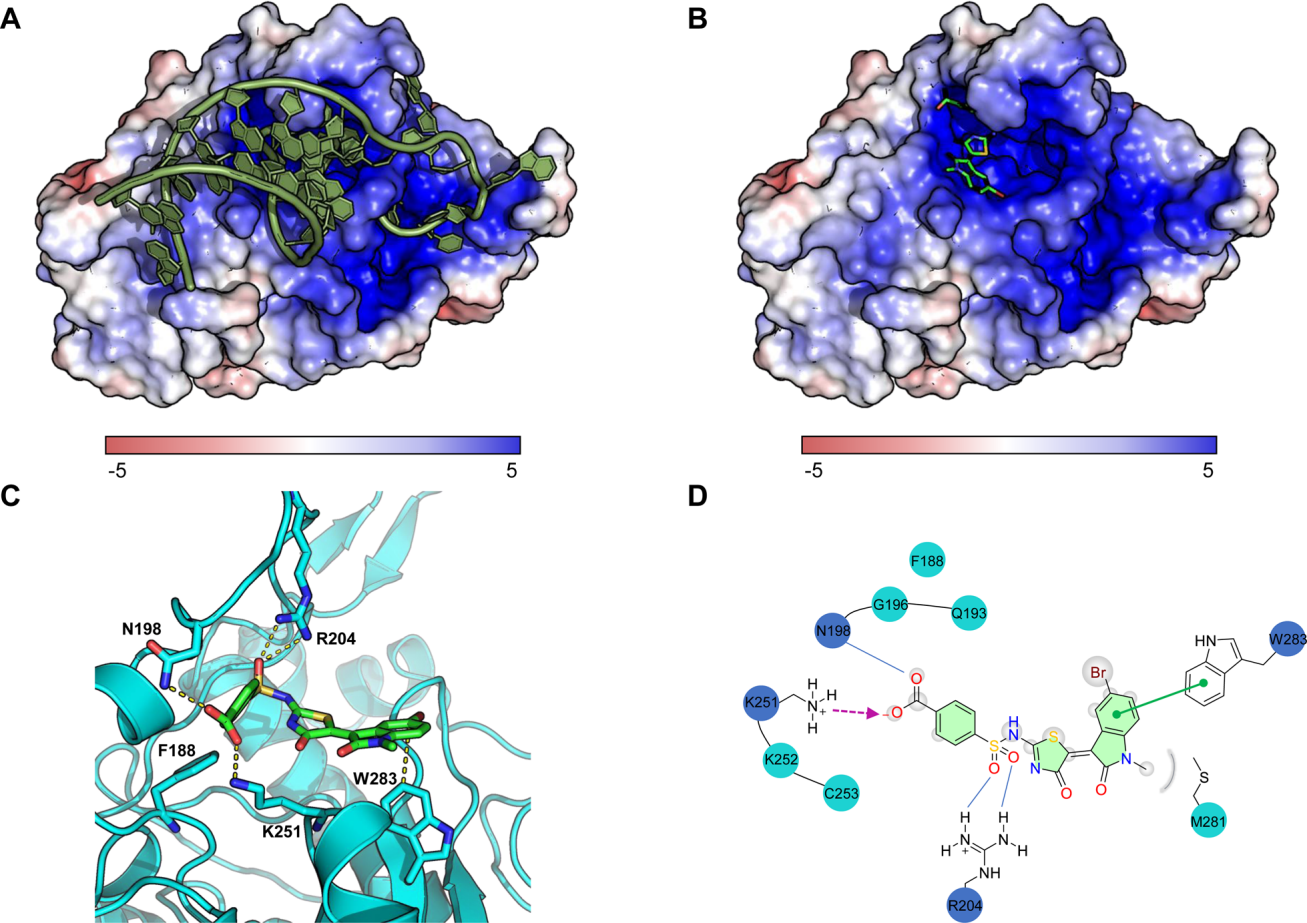
Molecular docking analysis of compound **45** with METTL16. (A) The catalytic *N*-terminal methyltransferase domain of METTL16 in complex with *MAT2A*-hp1 3′UTR hairpin (PDB: 6DU4, residues 1–310). The protein is shown in the charged surface. (B) An optimal docking configuration of **45** in complex with the RNA-binding site of METTL16 (PDB: 6DU4). METTL16 is shown as the charged surface and **45** as the green carbon backbone. (C) The ribbon illustration of METTL16 (in cyan) with **45** (in green carbon backbone). Selected key interacting residues are depicted as sticks. (D) 2D illustration of the interaction between **45** and METTL16. Residues directly involved in compound interaction are indicated in blue circles, and residues indirectly involved in compound interaction are indicated in cyan circles. The proposed binding mode shows a salt bridge (Lys251) and a hydrogen bond (Asp198) between METTL16 and the carboxylic acid residue of **45**. Arg204 thereby forms additional hydrogen bonds with sulfonamide oxygens. Depicted in green, **45** forms a π–π interaction with Trp283.

To study the binding mode of the aminothiazolones, we performed a molecular docking analysis based on the complex structure between METTL16 MTD and *MAT2A-*hp1 (Figure 7A,B).^9^ An optimal potential binding mode between aminothiazolone **45** and the RNA-binding site of METTL16 showed a few key interactions that are consistent with our experimental observation. First, the crucial role of carboxylic acid was demonstrated by a stable salt bridge involving Lys251 (Figure 7C,D). Second, the hydrogen bonds between Arg204 and the sulfonamide oxygens echoed the importance of the sulfonamide group, as demonstrated in the experimental data for compounds **34**–**37** and **39**–**44** that showed either reduced or loss of activity (Figures 3,4). Third, the phenyl ring of the oxindole moiety formed a π–π stacking interaction with Trp283 with another potential π–π interaction between the sulfonamide moiety and Phe188 due to the dynamic configuration of the binding that may lead to rotation in closer proximity to **45** (Figure 7C,D). The proposed binding mode of **45** offers a plausible explanation for its inhibitory impact on METTL16 as it involves amino acids in the proximity of the conserved NPPF catalytic motif (Arg204, Phe188). Consequently, the interaction of **45** with METTL16 has the potential to obstruct the RNA-binding site, thereby disrupting the protein–RNA interaction between METTL16 and *MAT2A*-hp1. In addition to the proposed binding mode of compound **45** using the MTD of METTL16 in complex with *MAT2A*-hp1, we performed another docking study involving only the methyltransferase domain of METTL16, which revealed a similar binding mode at the RNA-binding site (Figure S9).

### Anticancer Activities Against Human Cancer Cells and Downstream Cellular Effects

**Figure 8:**
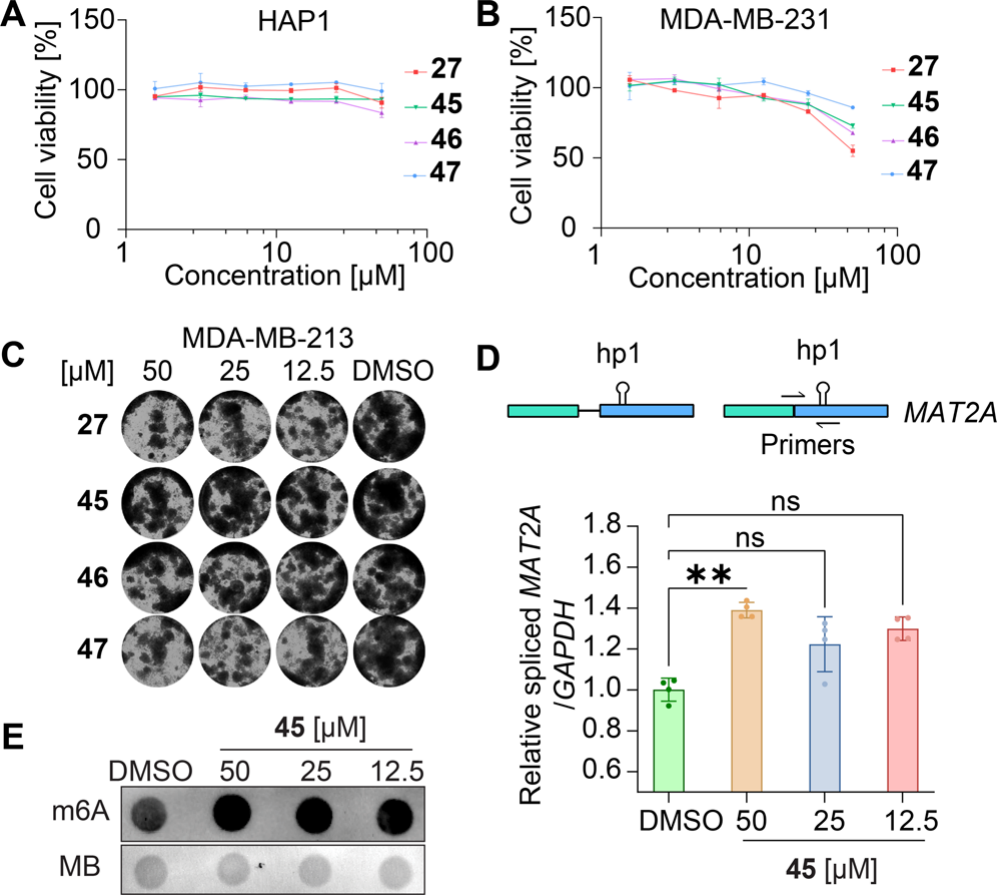
Cellular effect of selected aminothiazolone METTL16 inhibitors **27**, **45**, **46**, and **47**. (A) Suppression of cell viability in HAP1 cells. Data are shown as mean ± SEM, *n* = 3. (B) Suppression on cell viability in MDA-MB-231 cells. Data are shown as mean ± SEM, *n* = 3. (C) Inhibition of colony formation in MDA-MB-231 cells. (D) Compound **45** treatment in MDA-MB-231 cells increases *MAT2A* mRNA splicing. Data are shown as mean ± SEM, *n* = 4. **p < 0.01, ns *p* > 0.05. (E) Compound **45** treatment increased the total m^6^A level in MDA-MB-231 cells; MB, methylene blue. **45** potassium salt was used for *MAT2A* splicing and total m^6^A level evaluation.

In light of the anticancer activities for reported small-molecule inhibitors targeting the METTL3/14 complex, we proceeded to evaluate the anticancer potential of the obtained METTL16 inhibitor against human cancer cells. First, the selected aminothiazolones showed a minimal to partial effect on cell viability against the chronic myeloid leukemia-derived HAP1 cells at the tested concentrations (Figure 8A). Also, in the lung cancer cell line A549 and colorectal carcinoma cell line HCT116, the selected aminothiazolones showed a rather negligible effect on cell viability (Figure S10A,B). We observed a mild antiviability effect of compound **27** through the cell viability assay against the triple-negative breast cancer cells MDA-MB-231 (Figure 8B). Then we evaluated the inhibition on colony formation for the aminothiazolone inhibitors against MDA-MB-231, which showed varied results among tested compounds (Figure 8C). For example, compounds **45** and **46** did not show obvious inhibition on colony formation, while compounds **27** and **47** that showed equivalent METTL16 inhibition in *in vitro* evaluations demonstrated mild inhibitory activity against colony formation in all three tested concentrations. The disparity between the anticancer activities in cells and the *in vitro* data for the involved inhibitors indicated that other physicochemical properties, e.g., cellular permeability, probably played important non-negligible roles in the translation from biochemical activity to cellular activity.

We further evaluated the impact of the inhibitors on *MAT2A* splicing and the total RNA m^6^A level in MDA-MB-231 and A549 cells. The treatment of **45** at 50 μM promoted *MAT2A* splicing in both cell lines (Figures 8D and S10C) and increased the total m^6^A mRNA levels (Figures 8E and S10D). The observed results might be explained by the dynamical regulation network among METTL16, *MAT2A* splicing, and SAM levels.^7^ Treatment with inhibitor **45** might induce METTL16 autoinhibition, which could occlude SAM binding^9^ and impede METTL16 from methylation. Consequently, increased splicing could occur to promote SAM biosynthesis. As SAM is involved in various RNA methylation processes mediated by other methyltransferases, e.g., METTL3/14, the occurrence of higher SAM levels might explain the upregulation of m^6^A RNA levels. However, the underlying mechanism requires further investigation to understand the dynamic m^6^A methylation network and the effects of METTL16 inhibitors.

## Conclusion

The human methyltransferase METTL16 is a crucial RNA-binding and -modifying protein regulating the abundant m^6^A RNA modification, while no potent small-molecule inhibitors targeting METTL16 have been reported and validated to date. In this study, we established an efficient discovery pipeline initiated by an FP screening assay to identify such METTL16-targeting small molecules that disrupted the interaction between METTL16 and its RNA substrates. Starting with the initial compound **1**, we collectively evaluated a total of 49 aminothiazolones as novel METTL16 inhibitors and presented the only systematic study available to date on the structural features required for METTL16 inhibition surrounding this first-in-class METTL16-targeting aminothiazolone scaffold. The identified inhibitors, such as **45** and **47**, disrupted the METTL16–RNA interaction (single-digit micromolar inhibition potency), bound to METTL16 MTD (single-digit micromolar *K*_D_), inhibited the methyltransferase activity of METTL16 MTD, suppressed cell viability and colony formation against cancer cells at varied extents, and modulated METTL16-related cellular pathways. On the other hand, further investigation to study the inhibitory mechanism and evaluate the selectivity profile, especially against other methyltransferases and RBPs, is needed to fully examine the associated biological and therapeutic potential of such METTL16-inhibiting small molecules. A common Rossmann fold is shared among all human m^6^A writers and SAM is a common methyl donor to catalyze the methylation mediated by m^6^A writers (Figure S11A),^10^ so it is difficult to achieve selectivity among human m^6^A writers by using SAM analogues or small molecules bound to the SAM-binding site. In comparison, the development of non-SAM-competitive inhibitors targeting m^6^A writers is a promising strategy as the primary sequence and secondary structure of METTL16 and METTL3/14 are significantly different (Figure S11B) and the RNA binding pocket of METTL16 is diverged.^9,10^ We showed in our study that the aminothiazolones are non-SAM competitive inhibitors; thus, selective inhibition of METTL16 over other writers is possible but warrants further evaluation against mechanisms of inhibition involving other RBPs. While an allosteric binding mode of the aminothiazolone inhibitors against METTL16 cannot be definitively excluded, our experimental findings predominantly suggested an RNA-competitive inhibition mode that was not aligned with SAM-competitive interactions. In addition to the limitations of the study mentioned above, we used truncated forms of the protein–RNA complexes in our evaluations, including the MTD instead of the full-length METTL16, the deletion form of the U6 snRNA (U6 snRNA Δ), and the *MAT2A* hairpin. Consequently, the inhibitors may behave differently in the physiological environment involving the full-length versions of the RBP and RNAs. Although the full anticancer potential of the strategy to target METTL16 with small-molecule inhibitors requires further extensive investigation concerning the current set of data, especially the observed modest cellular activity, the identified aminothiazolone inhibitors can be useful probes to study unknown functions of METTL16 and lay the basis for the development of small-molecule therapeutic agents disrupting the function of METTL16.

## Experimental Section

### Protein Expression and Purification

Plasmid encoding full-length human METTL16 sequence is a gift from Prof. Jessica A. Brown’s lab. METTL16 MTD (1–291) was subcloned to pOPINB plasmid with an N-terminal His tag followed by an HRV 3C cleavage site; used primers are listed in Table S1. The plasmid was transformed into *E. coli* Rosetta (DE3) competent cells, expressed and purified as previously described with some modifications.^10^ Briefly, cells were cultured in fresh LB medium supplemented with 50 μg/mL kanamycin and 34 μg/mL chloramphenicol at 37 °C and 170 rpm shaker, after OD reached 0.8, chilled to 18 °C, IPTG was added to a final concentration of 0.5 mM to induce protein expression at 18 °C and 170 rpm for 16–20 h. Cells were harvested by 5000*g* centrifugation at 18 °C for 15 min, and then the cell pellet was resuspended in lysis buffer (50 mM HEPES, pH 7.5, 500 mM NaCl, 5% v/v glycerol, 0.5 mM TCEP, 5 mM imidazole) and supplemented with 1 mM PMSF before being lysed by sonication on ice. The cell lysate was centrifuged at 25000*g* at 4 °C for 30 min, and the supernatant was filtered and loaded to a nickel-affinity column (Ni Sepharose 6 Fast Flow, GE Healthcare). The column was washed by 50 mL of lysis buffer, 50 mL of lysis buffer supplemented with 20 mM imidazole, 30 mL of lysis buffer supplemented with 30 mM imidazole subsequently, and finally eluted using 15 mL of lysis buffer supplemented with 300 mM imidazole. The elution was treated with His-HRV-3C protease in the ratio of 1:30 w/w and dialysis in dialysis buffer (50 mM HEPES, pH 7.5, 250 mM NaCl, 5% v/v glycerol, 0.5 mM TCEP) overnight to lower the imidazole concentration to around 20 mM and to cleave the His-tag. Then the protein sample was loaded onto a nickel-affinity column again to remove the cleaved His-tag and his-HRV-3C protease. The flow-through was collected and concentrated using an Amicon ultracentrifugal filter unit (Millipore). The protein was further purified by the SEC 75 column using SEC buffer (20 mM pH 7.5, 200 mM NaCl, 0.5 mM TCEP,2% v/v glycerol). Protein purity and size were confirmed by SDS–PAGE and LC–MS. Purified protein was concentrated to 10 mg/mL, aliquoted, snap frozen by liquid nitrogen, and stored at −80 °C for future experiments. His-tagged METTL16 (1–291) was purified using the same protocol, without protease cleavage and the reverse nickel column.

### Compound Screening

The compound screening was performed against a chemical library containing about 25000 compounds provided by COMAS (Compound Management and Screening Center, MPI Dortmund), using the FP assay described below with 30 μM compound, 80 nM METTL16 protein, 2 nM FAM-*MAT2A-*hp1 RNA (FAM-CUUGUUGGCGUAGGCUACAGAGAAGCCUU CAAG) in 384-well black plates (4514, Corning) with a final volume of 18 μL. Then, 0.27 μL (2 mM compounds stock solution) or 0.054 μL (10 mM compounds stock solution) compounds and the same volume of DMSO as the control and blank group were transferred to the plates using ECHO machine, followed by the dispensation of 9 μL 160 nM protein solution (or 9 uL buffer as the Blank group) to the plates using Multidrop dispenser. After 30 min of incubation at room temperature, 9 μL of 4 nM FAM-RNA solution was dispended to each well, and the fluorescence polarization was measured by a TECAN Spark plate reader. Protein and FAM-RNA solutions were prepared using the FP buffer (20 mM HEPES, pH 7.5, 50 mM NaCl, 0.05% v/v Tween 20, and 0.05 mg/mL BGG). Primary screen hits were tested in serial dilutions to determine the IC_50_ value, validating the potential lead compounds through the orthogonal EMSA assay.

### Fluorescence Polarization (FP) Assay

The FP assay was performed using 384-well black plates (Corning #4514) in a total reaction volume of 20 μL, with the final concentration of protein and RNA being 50 and 5 nM respectively. Compounds were diluted in FP buffer and incubated with protein for 30 min at room temperature, then FAM-*MAT2A-*hp1 RNA probe was added and the fluorescence polarization was measured using a TECAN Spark plate reader, under the excitation wavelength of 485 nm and the emission wavelength of 535 nm with bandwidth of 20 nm. 1% DMSO was used as the control. The inhibition was calculated with the equation: inhibition = 100%(Control – X)/(Control – Blank); Control: DMSO with protein and FAM-RNA; Blank: DMSO with FAM-RNA; X: compound with protein and FAM-RNA. The IC_50_ value was further determined using GraphPad Prism 9. The FP assay using U6 snRNA Δ followed the same protocol aforementioned, and the final concentration of METTL16 MTD protein and U6 snRNA are 50 and 5 nM, respectively. The polarization was measured under an excitation wavelength of 630 nm and an emission wavelength of 680 nm with a bandwidth of 20 nm.

### Electrophoretic Mobility Shift Assay (EMSA)

METTL16 MTD protein (1–291) was incubated with the indicated compound or DMSO in a buffer containing 20 mM HEPES, pH 7.5, 50 mM NaCl, 0.05% v/v Tween 20, and 0.05 mg/mL BGG at room temperature for 30 min; subsequently, FAM-*MAT2A*-hp1 RNA probe was added and incubated at room temperature for 10 min, the final of protein and RNA are 50 and 5 nM respectively, and 1% DMSO was used as the control. After incubation with the RNA probe, the sample was loaded to 6.6% native PAGE gel with 6× loading buffer (45% H_2_O, 40% glycerol, 15% 10× TBE buffer, 0.1% bromphenol blue) and separated by electrophorese with 0.5xTBE buffer as the running buffer, at 120 V for 40 min at 4 °C in the dark. The gel was detected and imaged by Chemi Doc MP (Bio-Rad). EMSA assay using U6 snRNA Δ followed the same protocol, and the final concentration of METTL16 MTD protein and U6 snRNA are 50 and 5 nM, respectively. EMSA for testing compounds with GGAUC-motif containing RNA and METTL16 MTD interaction was performed using the method described above, expecting that the final of METTL16 MTD protein and FAM-labeled-GGACU RNA are 600 nM and 5 nM respectively. EMSA for RNA binding: indicated concentrations of fluorophore-labeled RNA were incubated with indicated concentrations of protein in EMSA buffer for 15 min at room temperature, then loaded to native PAGE gels and imaged with the method described above.

### Differential Scanning Fluorimetry

The DSF assay was performed in PBS buffer containing 2 mm DTT, in a total reaction volume of 20 μL with the final concentration of 1 μM METTL16 MTD protein and 5 × SYPRO Orange fluorescent dye (Sigma S5692) and 0.35% DMSO (including DMSO from SYPRO Orange). The melt curve was measured at a temperature range from 25 to 95 °C and in increments of 1 °C for 30 s using a Bio-Rad CFX96 Real-Time PCR Detection System with the FRET scan mode. The midpoint of the transition (*T*_m_) was obtained by fitting the melting curve to Boltzmann sigmoidal in GraphPad Prism and the thermal shift (Δ*T*_m_) was calculated using the equation Δ*T*_m_= *T*_m(compound)_ – *T*_m(DMSO)_.

### SwitchSENSE Biosensor Assay

The biosensor assay was performed using a heliX instrument (Dynamic Biosensors) with a heliX adapter chip. The His-capture kit (HK-NTA-1) was used to functionalize and regenerate the chip. 100 nM His-tagged METTL16 MTD was captured to the surface and regenerated with imidazole solution (250 mM in 10 mM Tris, pH 7.4, 140 mM NaCl, 0.05% Tween 20, 50 μM EDTA, 50 μM EGTA) each time after measurement. PE140 buffer was used as the running buffer (10 mM Na_2_HPO_4_/NaH_2_PO_4_, pH 7.4, 140 mM NaCl, 0.05% Tween 20, 50 μM EDTA) and 0.2% DMSO was used as concertation 0. The association and dissociation flow rates were 200 μL/min, with an association time of 90 s and a dissociation time of 120 s. Data were analyzed using heliOS using the ‘kinetics mono- & biphasic-free amplitudes fitting’ mode.

### Isothermal Titration Calorimetry

ITC was performed by using the MicroCal PEAQ-ITC system (Malvern) at 25 °C. METTL16 MTD protein was directly used after purification. 700 μM protein in SEC buffer (20 mM pH 7.5, 200 mM NaCl, 0.5 mM TCEP,2% v/v glycerol) supplemented with 0.5% DMSO were loaded to the syringe, 50 μM compound in SEC buffer (final 0.5%DMSO) was loaded to the cell, both samples were adjusted to 25 °C and degassed before loading. The experiment was carried out using the reference power of 10, with 19 injections. Data were analyzed using the MicroCal PEAQ-ITC Analysis Software.

#### *In Vitro* Methylation Assay

The methyltransferase activity of METTL16 MTD was measured using an MTase Glo kit (Promega V7601) following the manufacturer’s instructions. The reaction was carried out in white 384-well plates (Corning #3824), with a total methyltransferase reaction volume of 8 μL, and the final concentrations of METTL16 MTD, *MAT2A-*hp1, and SAM were 1, 1, and 10 μM, respectively, and 1% of DMSO was used as a control. Compounds were diluted in the reaction buffer (20 mM Tris, pH 8.0, 50 mM NaCl, 1 mM EDTA, 3 mM MgCl_2_, 0.1 mg/mL BSA, 1 mM DTT) and preincubated with METTL16 MTD protein for 30 min at room temperature, 2× substrate solution freshly prepared in 1× reaction buffer containing *MAT2A-*hp1 and SAM (supplemented in MTase Glo kit) was added to the wells and allowed to stand for 1 h at room temperature, then 5× MTase-Glo Reagent was added and incubated 30 min at room temperature, after incubation the MTase-Glo Detection Solution was added and incubated for another 30 min at room temperature, luminescence was measured using a TECAN Spark plate reader. To use SAM at a different final concentration, the concentration of SAM was adjusted accordingly in a 2× substrate solution. The inhibition was calculated with the equation: Inhibition = 100% (Control – X)/(Control – Blank), Control: DMSO with protein and RNA; Blank: DMSO with RNA; X, compound with protein and RNA.

#### Cell Culture

MDA-MB-231 and HCT116 were purchased from DSMZ (German Collection of Microorganisms and Cell Cultures), and A549 was purchased from ATCC (American Type Culture Collection). MDA-MB-231, HCT116, and A549 were cultured in the high-glucose DMEM medium (Gibco 61965026) with 10% FBS (Gibco 10500064) and 1% penicillin–streptomycin (Gibco 15140122). HAP1 was purchased from Horizon and was cultured in the IMEM medium (Gibco 12 440 046) supplemented with 10% FBS (Gibco 10500064) and 1% penicillin–streptomycin (Gibco 15140122). All cells were cultured at 37 °C with 5% CO_2_ atmosphere.

#### Antiproliferation Assay

HCT116 and HAP1 cells were seeded in 96-well plates with 2000 cells per well. After being cultured overnight, compounds were added, and the DMSO treatment (0.5%) was used as a control. After 72 h treatment, CCK-8 solution (Vazyme, A311) was added to the wells and incubated at 37 °C for 2 h, and then the absorbance was measured at 450 nM using a TECAN Spark plate reader. Cell viability was calculated with the following equation: Cell viability = 100% (X – Blank)/(Control – Blank); Control: the absorbance of DMSO treatment; Blank: the absorbance of only medium; X: the absorbance of compound treatment.

#### Colony Formation Assay

MDA-MB-231 cells were collected and seeded with the density of 1000 cells per well into 24-well plates; after being cultured overnight, the medium was exchanged and treated with indicated compounds or DMSO (0.5%) as the control. The medium change and treatment were performed every 3 days. After 7 days, the cell culture medium was discarded, and cells were washed with PBS and fixed with 4% paraformaldehyde solution at room temperature for 15 min; the paraformaldehyde solution was removed, and cells were washed with PBS again to remove the paraformaldehyde. Then cells were stained with 0.1% (w/v) crystal violet. After staining for 15 min, the cells were washed with H_2_O to remove the extra dye and then photographed.

#### RNA Purification

MDA-MB-231 cells and A549 cells were seeded into 6-well plates at 70% confluency and cultured overnight. Afterward they were treated with compound **45** potassium salt for 24 h. After the compound treatment, the cell culture medium was discarded and cells were washed with DPBS three times. The total RNA was then purified using RNeasy Mini Kit (Qiagen 74106) following the manufacturer’s protocol.

#### Reverse Transcription-Quantitative Polymerase Chain Reaction (RT-qPCR)

The reverse transcription was performed by using High-Capacity cDNA Reverse Transcription Kits (Thermo Fisher 4368814) with 500 ng of total RNA. qPCR was performed using PowerUp SYBR Green Master Mix (Thermo Fisher, A25742) with a 10 μL volume. The cycling was performed using Bio-Rad CFX96 Real-Time PCR Detection System following the standard cycling mode (primer Tm ≥ 60 °C) on the manufacturer’s protocol. The primers used are listed in Table S1. The *p* value was calculated using GraphPad Prism software’s one-way ANOVA analysis.

#### Dot Blot

Concentrations of the total RNA were measured by NanoDrop and each sample was calibrated to the same concentration using RNase-free water. RNA samples were heated at 95 °C for 3 min and immediately chilled on ice to disrupt the secondary structures. The total RNA (1 μg) was dropped onto a positively charged nylon membrane (Invitrogen, AM10102). The membrane with RNA side up was immediately transferred to the UVP Cross-linker (Analytik Jena) with 254 nM bulb and cross-linked under the UV light for 5 min two times, then the membrane was washed in TBST (1xTBS with 0.1% Tween 20) for 10 min at room temperature and blocked with 5% skimmed milk in TBST for 1 h at room temperature. The membrane was incubated with the anti-m^6^A antibody (Synaptic systems 202003, 1 μg/mL) overnight at 4 °C. After washing in TBST for 10 min three times, the membrane was incubated with the secondary antibody HRP-conjugated Goat Anti-Rabbit (Proteintech, SA00001–2, 1:3000) at 37 °C shaker for 1 h. The membrane was further washed with TBST and detected with the Amersham ECL Prime Western Blotting Detection Reagent. After imaging, the membrane was washed with TBST and stained with methylene blue (0.2% methylene blue in 0.2 M sodium acetate and 0.2 M acetic acid) as the loading control.

## Supporting Information Available

Supplementary Figures S1–S13, supplementary Table S1, supplementary methods, general chemistry information, synthetic procedures, compound characterizations, and ^1^H and ^13^C NMR spectra (PDF)
(XLSX)

## Abbreviations

DSF: differential scanning fluorimetry
IF3a/b: eukaryotic initiation factor 3a/b
FP: fluorescence polarization
m^6^A: *N*^6^-methyladenosine
METTL3: methyltransferase-like protein 3
METTL14: methyltransferase-like protein 14
METTL16: methyltransferase-like protein 16
MTD: methyltransferase domain
SAM: *S*-adenosylmethionine
snRNA: small nuclear RNA
TRMT112: tRNA-methyltrasnferase 112 complex
