# Supplementary material for: Aminothiazolone Inhibitors Disrupt the Protein–RNA Interaction of METTL16 and Modulate the m6A RNA Modification
Source: JACS Au. 2024 Mar 21;4(4):1436–49. doi: 10.1021/jacsau.3c00832 (PMC11040665; doi:10.1021/jacsau.3c00832)
Supplement: Supplementary file 1 — au3c00832_si_001.pdf [file au3c00832_si_001.pdf]

Supporting Information

Aminothiazolone Inhibitors Disrupt the Protein–RNA Interaction of METTL16 and Modulate the m^6A RNA Modification

Yang Liu,[†,‡,§,#] Georg L. Goebel,[†,‡, §,#] Laurin Kanis,[†,‡,§] Oguz Hastürk,[†,‡,§] Claus Kemker,[†,‡,§] and Peng Wu[†,‡,*]

[†]Chemical Genomics Centre, Max Planck Institute of Molecular Physiology, Dortmund 44227, Germany

[‡]Department of Chemical Biology, Max Planck Institute of Molecular Physiology, Dortmund 44227, Germany

[§]Faculty of Chemistry and Chemical Biology, TU Dortmund University, Dortmund 44227, Germany

[#]equally contributed authors

*Corresponding Author: P. Wu, email: peng.wu@mpi-dortmund.mpg.de

CONTENTS

SUPPLEMENTARY FIGURES ……………………………………………..…S03

SUPPLEMENTARY TABLE ………………………………………………..S16

SUPPLEMENTARY METHODS……………………………………..……..S17

GENERAL CHEMISTRY INFORMATION ………………………………..S18

General Procedure A (thiazolidinonesulfonamide formation) ……..……… ..S19

General Procedure B (thiazolidinoneamide formation) ……….……………..S25

General Procedure C (Knoevenagel condensation) …………………………..S27

General Procedure D (One-pot condensation) ………………………………S49

NMR SPECTRA …………………………………………………………..S53

REFERENCES ……………… ………………………………………..…S150

SUPPLEMENTARY FIGURES

A

```
        10         20         30         40         50         60
MALSKSMHAR NRYKDKPPDF AYLASKYPDF KQHVQINLNG RVSLNFKDPE AVRALTCTLL
        70         80         90        100        110        120
REDFGLSIDI PLERLIPTVP LRLNYIHWVE DLIGHQDSDK STLRRGIDIG TGASCIYPLL
       130        140        150        160        170        180
GATLNGWYFL ATEVDDMCFN YAKKNVEQNN LSDLIKVVKV PQKTLLMDAL KEESEIIYDF
       190        200        210        220        230        240
CMCNPPFFAN QLEAKGVNSR NPRRPPPSSV NTGGITEIMA EGGELEFVKR IIHDSLQLKK
       250        260        270        280        290        300
RLRWYSCMLG KKCSLAPLKE ELRIQGVPKV TYTEFCQGRT MRWALAWSFY DDVTVPSPPS
       310        320        330        340        350        360
KRRKLEKPRK PITFVVLASV MKELSLKASP LRSETAEGIV VVTTWIEKIL TDLKVQHKRV
       370        380        390        400        410        420
PCGKEEVSLF LTAIENSWIH LRRKKRERVR QLREVPRAPE DVIQALEEKK PTPKESGNSQ
       430        440        450        460        470        480
ELARGPQERT PCGPALREGE AAAVEGPCPS QESLSQEENP EPTEDERSEE KGGVEVLESC
       490        500        510        520        530        540
QGSSNGAQDQ EASEQFGSPV AERGKRLPGV AGQYLFKCLI NVKKEVDDAL VEMHWVEGQN
       550        560
RDLMNQLCTY IRNQIFRLVA VN
```

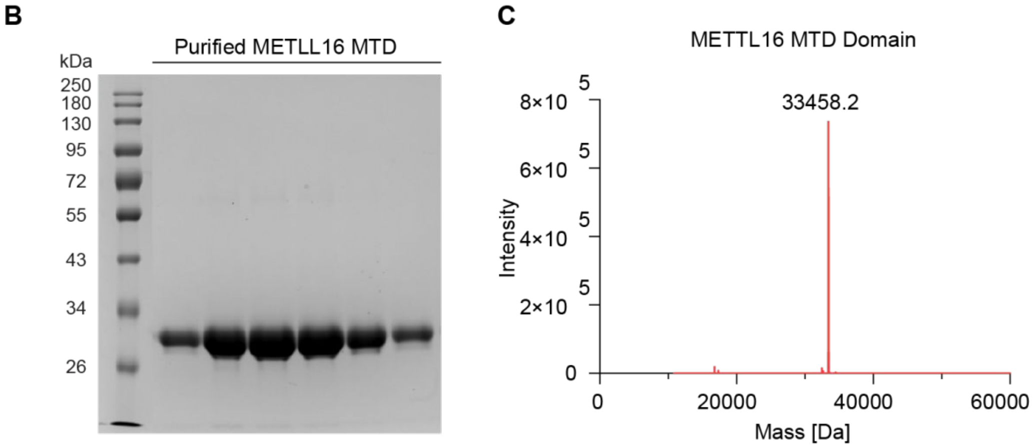

Figure S1. METTL16 (1-291) protein purification. (A) Sequence of METTL16 full-length protein, purple fonts: the sequence 1–291 used in this project. (B) SDS-PAGE analysis of the purified METTL16 protein. (C) LC-MS analysis of purified METTL16 protein. Expected Mass for METTL16 (1-291), 33455.7 Da; Found: 33458.2 Da.

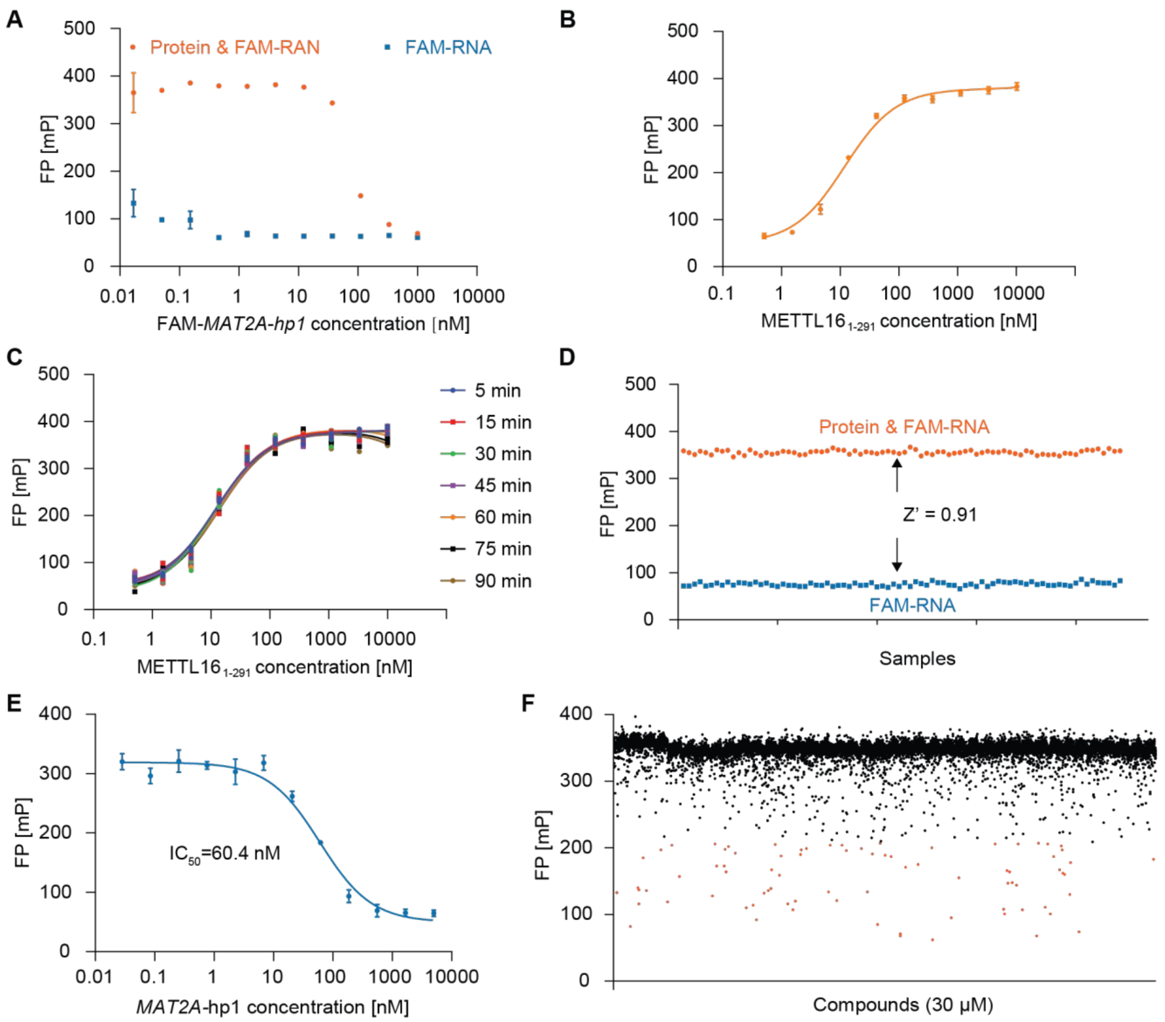

Figure S2. Establishment of the FP assay and screening for small-molecule METTL16 inhibitors. (A) FAM-*MAT2A-hp1* RNA concentration titration with 1000 nM METTL16. The increased concentration of FAM-RNA probe led to a decrease in the FP signal. Based on the titration result, a final concentration of 2 nM FAM-*MAT2A-hp1* RNA was chosen to achieve an optimal FP signal. Data are shown as mean ± SEM, n=4. (B) METTL16 protein concentration titration with 2 nM FAM-*MAT2A-hp1*. The presence of 80 nM METTL16 protein led to 85% saturation of FAM-*MAT2A-hp1* probe, leading to a good signal-to-background ratio. Data are shown as mean ± SEM, n=4. (C) Varied RNA incubation time ranging between 5 min and 90 min did not have a significant impact on the FP signal. Thus, an incubation time of 5 min was used in our assays. Data are shown as mean ± SEM, n=4. (D) Testing with a smaller set of compounds to evaluate the assay. Z-factor calculated with the equation: $Z' = 1-(3\sigma_{c+}=3\sigma_{c-})/(|\mu_{c+} - \mu_{c-}|) = 0.91$; σ_{c+} and σ_{c-} are standard deviations of the signals for protein binding with the FAM-RNA probe and the unbind FAM-RNA probe alone, respectively; μ_{c+} and μ_{c-} are means of bound (protein with FAM-RNA probe) and unbound (FAM-RNA probe only) signals, respectively. (E) Competition experiment with unlabeled *MAT2A-hp1* RNA, which competitively bound to METLL16 in a dose-dependent manner. Hence the binding between METTL16 with FAM labeled RNA is inhibited with subsequently decreased FP signal. (F) Primary screening result, orange dots indicate primary hits that showed an inhibition of no less than 50%.

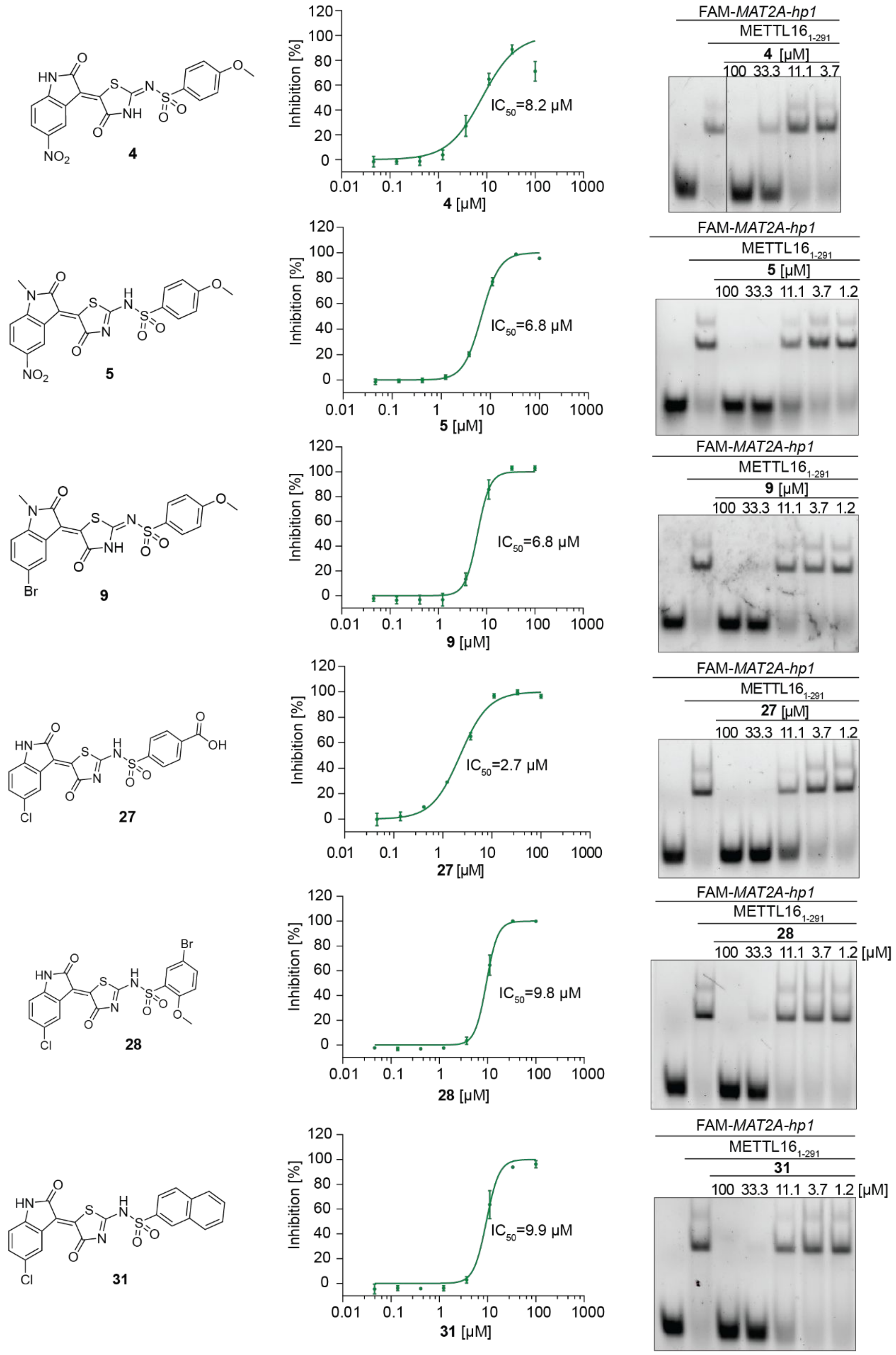

Figure S3. Potent compounds selected from Figure 3 that showed single-digit micromolar inhibitory potency against METTL16 (Left: structure; middle: FP results; right: EMSA results). FP data are shown as mean ± SEM, n=4.

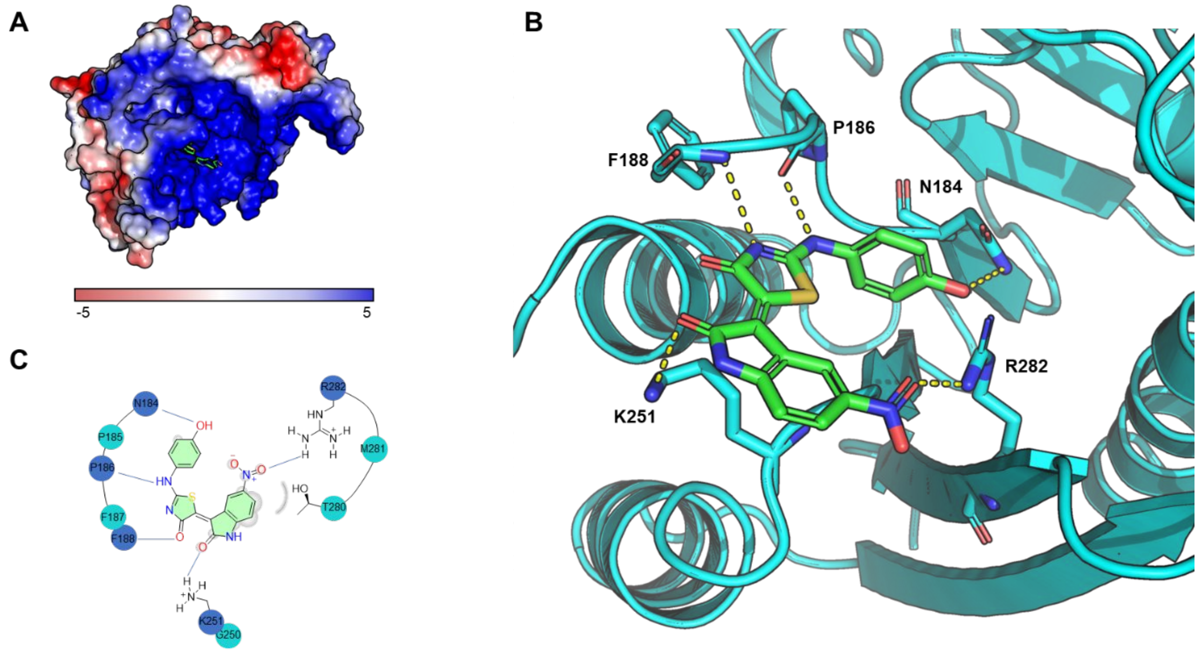

Figure S4. Molecular docking analysis of compound **42** with METTL16. (A) An optimal docking configuration of **42** in complex with the RNA-binding site of METTL16 (PDB code: 6B91). METTL16 is shown in charged surface and **42** in green carbon backbone. (B) The ribbon illustration of METTL16 (in cyan) with **42** (in green carbon backbone). Selected key interacting residues are depicted as sticks. (C) 2D illustration of the weaker binding interaction between **42** and METTL16. The proposed binding mode of **42** shows hydrogen bonds between Asn184 and the phenolic hydrogen of **42**. Another hydrogen bond between the backbone of Pro186 and the exocyclic amine of the pseudothiohydantoin core. Another hydrogen bond is formed by the backbone of Phe188 and the oxygen of **42**. The indolone moiety of **42** shows weak hydrophobic interactions with Thr280 and additionally the nitro group forms another hydrogen bond with Arg282 underlining the importance of the functional group contributing to the binding activity against METTL16. Presumably due to these rather weak hydrogen bonds and the missing salt bridge, the decreased inhibitory activity of 43 against METTL16 could be explained.

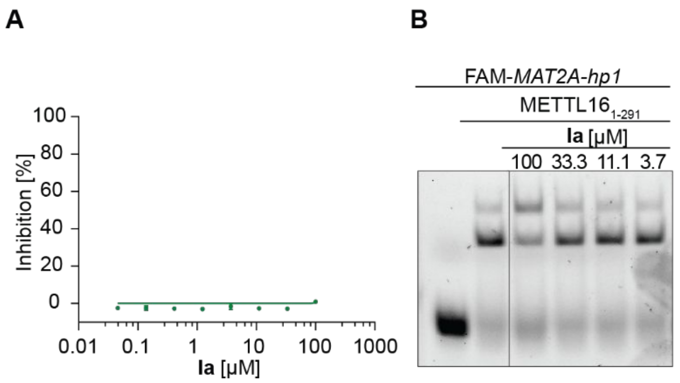

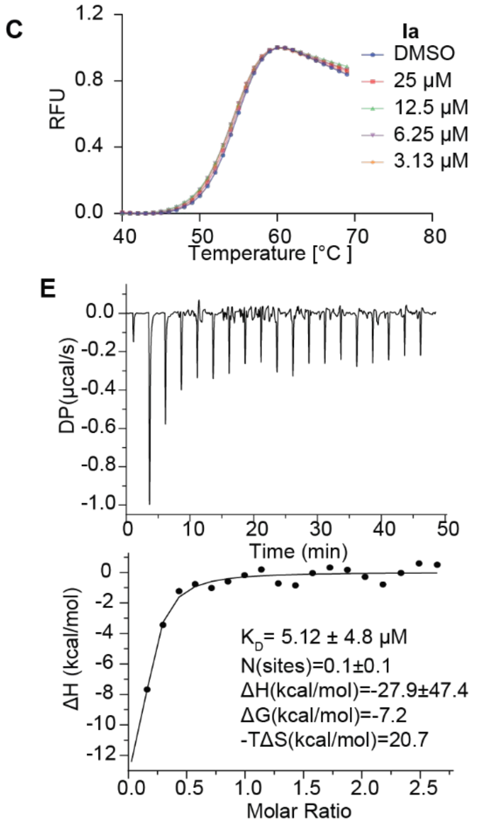

D

T_m [°C]	25 μM	12.5 μM	6.25 μM	3.13 μM
DMSO	53.83±0.12			
Ia	53.77±0.19	53.68±0.11	53.64±0.07	53.72±0.08
27	55.43±0.12	55.07±0.20	54.48±0.28	54.30±0.05
45	55.08±0.10	54.96±0.15	54.59±0.18	54.32±0.11
46	54.68±0.16	54.33±0.11	54.10±0.08	53.93±0.05
47	56.74±0.58	55.48±0.09	54.74±0.03	54.44±0.15

Figure S5. Compound **Ia** as a negative control for DSF assay ,T_m values of tested compounds and ITC result of compound **47**. (A) FP assay result of **Ia**, IC_{50}: > 100 μM. Data are shown as mean ± SEM, n=4. (B) EMSA assay result of compound **Ia**. (C) Melting curve of METTL16 in the DSF assay treated with different concentrations of **Ia**. Data are shown as mean ± SD, n=2. (D) T_m values of DMSO control and compound **Ia**, **27**,**45**,**46**, and **47**, assay was conducted with two biological repeats in duplicate. Data are shown as mean ± SEM, n=4. (E) ITC validation of compound **47**, using 700 μM METTL16 (1-291) protein to titrate 50 μM compound **47**.

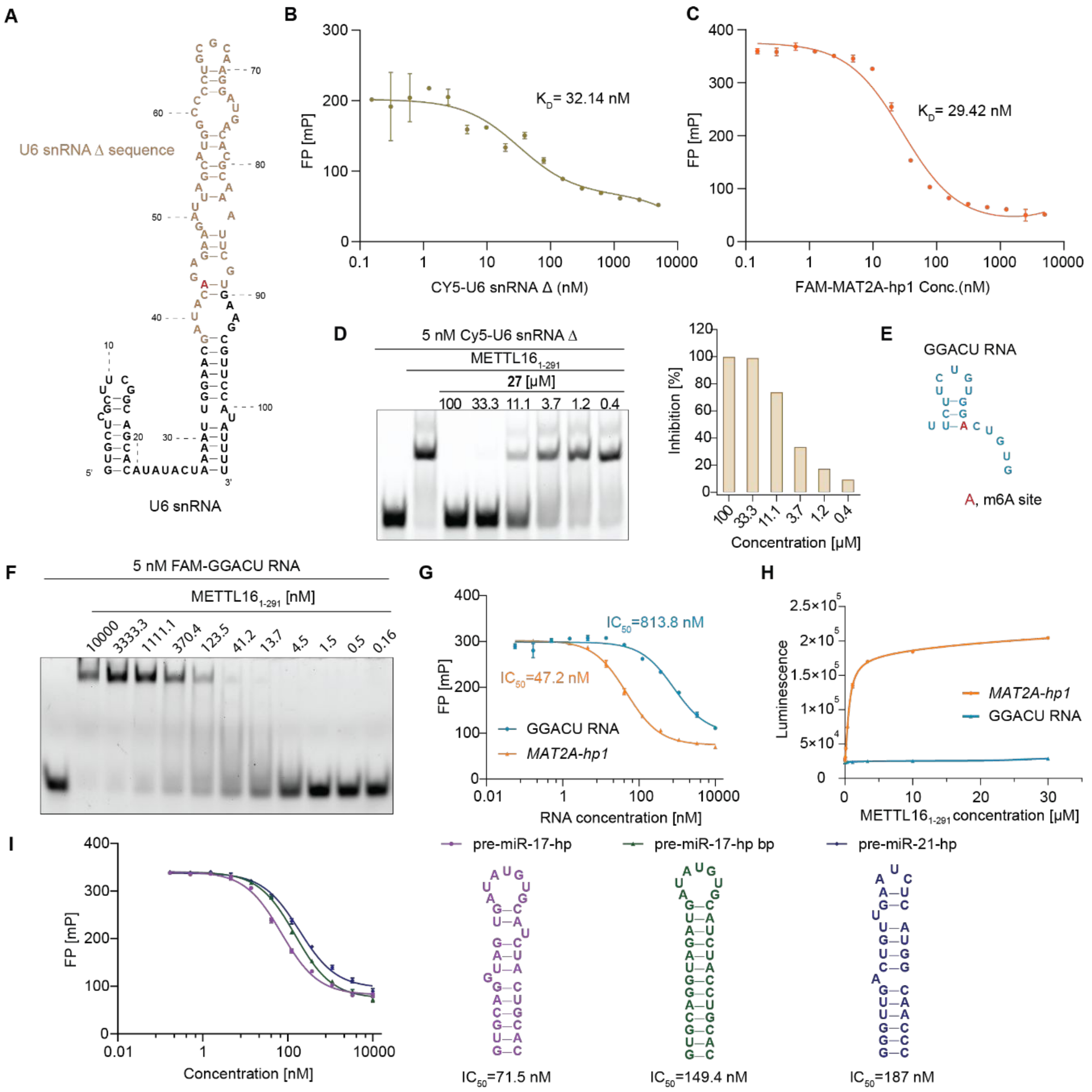

Figure S6. U6 snRNA Δ binds to METTL16 MTD with sane affinity as *MAT2A-hp1* and aminothiazolones disrupted the binding. (A) U6 snRNA sequence and the U6 snRNA deletion sequence (marked with earth color) used in this study, structures were predicted using the RNA structure web service.[1] (B) The affinity of U6 snRNA Δ towards METTL16 MTD measured by FP assay. Data are shown as mean ± SEM, n=4. (C) The affinity of *MAT2A-hp1* towards METTL16 MTD measured by FP assay. Data are shown as mean ± SEM, n=4. (D) Compound **27** dose-dependently interrupted METTL16 MTD-U6 snRNA Δ interaction. (E) Predicted GGACU-RNA structure using the RNA structure web service.[1] (F) METTL16 MTD domain binds to the GGACU-containing RNA in EMSA. (G) Unlabelled GGACU-RNA and *MAT2A-hp1* RNA disrupted the interaction between METTL16 and the FAM-labelled *MAT2A-hp1* RNA in the FP assay. Data are shown as mean ± SEM, n=4. (H) In vitro methylation assay using different RNA substrates, METTL16 catalysed the methylation on *MAT2A-hp1* RNA substrate. In contrast, METTL16 showed no catalytic activity towards a GGACU-containing RNA. Data are shown as mean ± SD, n=2. (I) Precursor micro-RNA hairpins competed METTL16-*MAT2A*-hp1 interaction in the FP assay. pre-*miR-17-hp* bp: base-paired RNA. RNA secondary structures were predicted using the RNA structure web service.[1] Data are shown as mean ± SEM, n=4.

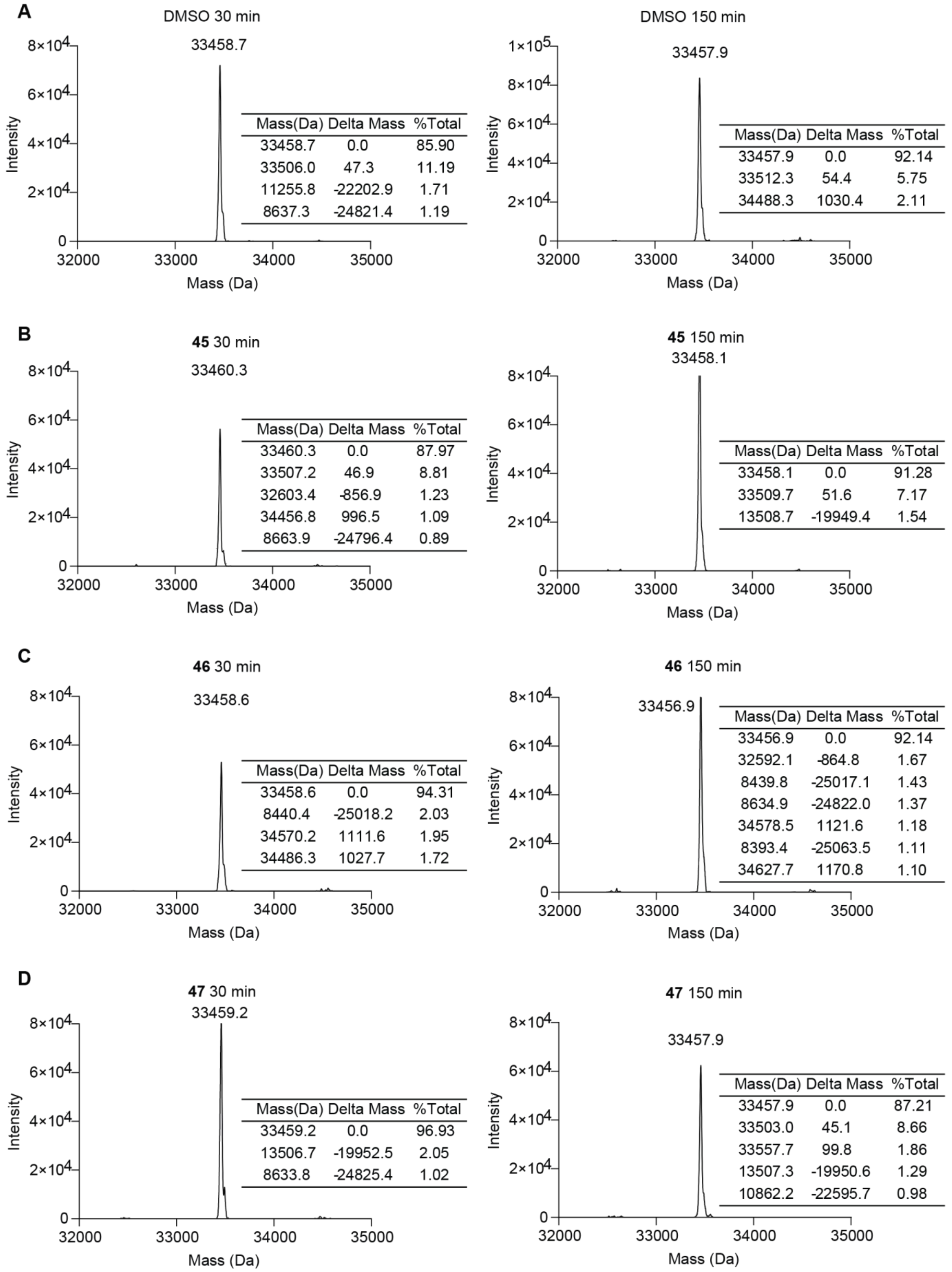

Figure S7. Covalent evaluation of METTL16 inhibitors via LC-MS. (A) Deconvoluted mass spectrum of 30 µM METTL16 (1-291) incubated with 1% DMSO for 30 min (left) and 150 min (right). (B), (C), (D) Deconvoluted mass spectrum of 30 µM METTL16 (1-291) incubated with 100 µM **45, 46** and **47** for 30 min (left) and 150 min (right). Expected Mass for METTL16 (1-291), 33455.7 Da. The observed small mass shift of ~50 Da appeared in both the DMSO control and compound-treated samples was probably formed by the solvent used in the LC-MS.

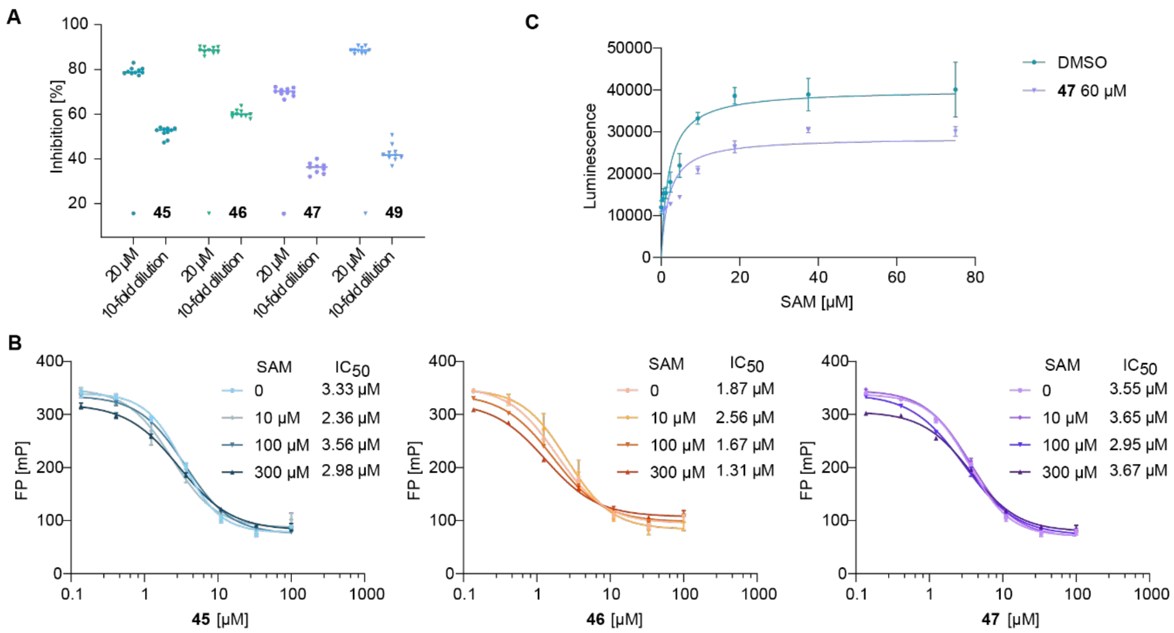

Figure S8. Aminothiazolones showed reversible and SAM-independent inhibition against METTL16. (A) Irreversible inhibition counter screen by incubating 5× METTL16 (1-291) (final assay concentration 250 nM) with 5 × IC_{50} compounds (final concentration 20 μM). After incubation, the FP was measured for both the 5x incubation samples (20 μM) and 10-fold diluted samples with FAM-labeled *MAT2A*-hp1 RNA (final concentration 5 nM). Data are shown as mean ± SEM, n=10. (B) FP assay by incubating SAM (Abcam, ab142221) with METTL16 (1-291) before measuring. Data are shown as mean ± SEM, n=4. (C) In vitro methylation assay using different SAM concentrations, start from 75 μM and two-fold dilution. Compounds or DMSO was incubated with protein for 30 min at room temperature, and a final concentration of 1 μM *MAT2A*-hp1 substrate with different SAM (supplemented in MTase Glo kit) concentrations was added to the reaction with a total reaction volume of 8 μL, after incubating for 1 h, 2 μL 0.5% TFA was added and the luminescence was further detected using the MTase Glo Kit and TECAN plate reader. Data are shown as mean ± SD, n=2.

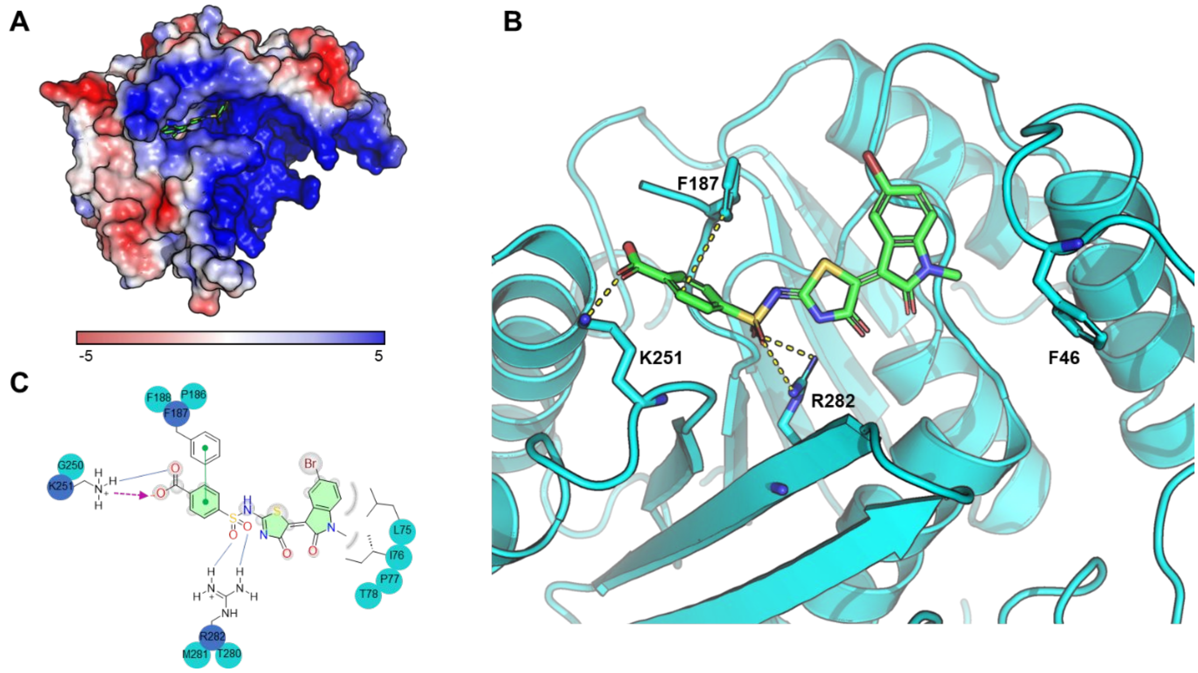

Figure S9. Molecular docking analysis of **45** with METTL16. (A) An optimal docking configuration of **45** in complex with the RNA-binding site of METTL16 (PDB code: 6B91). METTL16 is shown in charged surface and **45** in green carbon backbone. (B) The ribbon illustration of METTL16 (in cyan) with **45** (in green carbon backbone). Selected key interacting residues are depicted as sticks. (C) 2D illustration of the interaction between **45** and METTL16. The proposed binding mode shows a salt bridge and a hydrogen bond formed between Lys251 and the carboxylic acid residue of **45**, similar to the binding mode proposed in the main text. Arg282 thereby forms additional hydrogen bonds with the sulfonamide oxygens emphasizing the importance of the moiety to interact with METTL16. Depicted in blue **45** forms a π-π interaction with Phe182. Additional hydrophobic interactions with Leu75 and Ile76 are depicted in cyan.

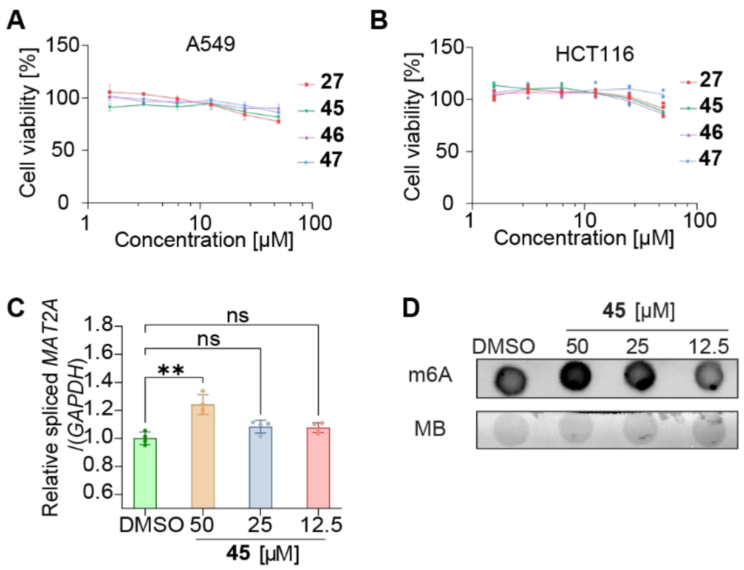

Figure S10. Cellular evaluation of selected METTL16 inhibitors. (A)Suppression on cell viability in A549 cells. Data are shown as mean ± SEM, n=3. (B) Suppression on cell viability in HCT116 cells. Data are shown as mean ± SEM, n=3. (C) Spliced *MAT2A* in A549 cells increased after treated 24 h with indicated dose of **45**. Data are shown as mean ± SEM, n=4. ***P*<0.01, ns *P*>0.05. (D) Total RNA m^{6}A level in A549 cells after treated 24 h with **45**. **45** potassium salt was used for *MAT2A* splicing and total RNA m^{6}A level evaluation.

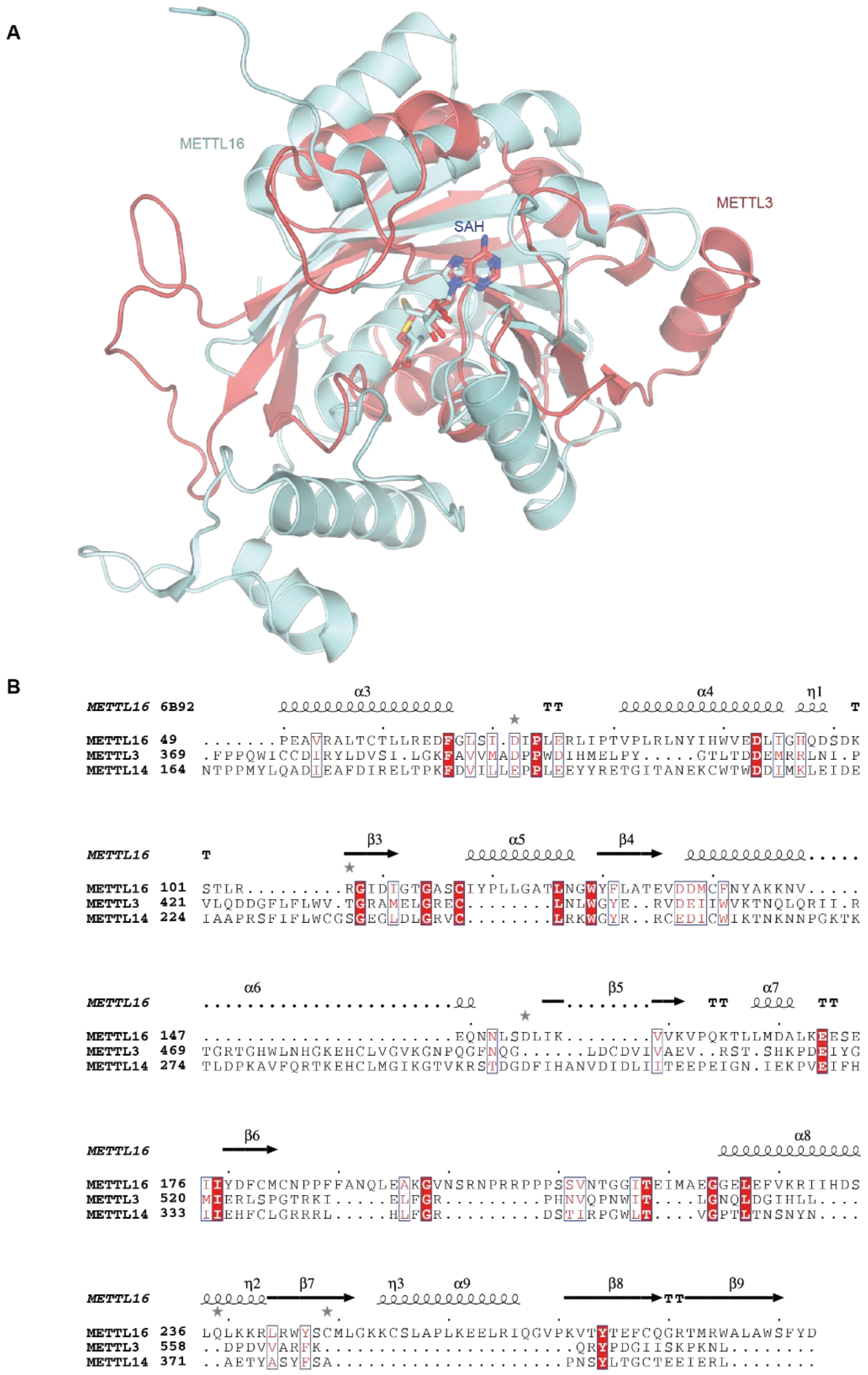

Figure S11. Alignment of METTL16 with METTL3/14. (A) Structure alignment of the SAM pocket of METTL16 (light-blue, 6B92) with METTL3(dark-red,5K7W), SAH (dark blue), S-adenosyl-L-homocysteine. (B) Sequence alignment of the Rossmann fold of METLL16(49-291), METTL3(369-580) and METTL14(164-380).

Fig 2D

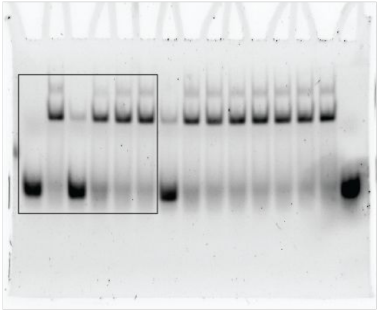

Fig 4C

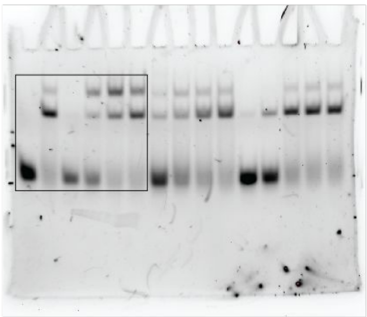

Fig 4F

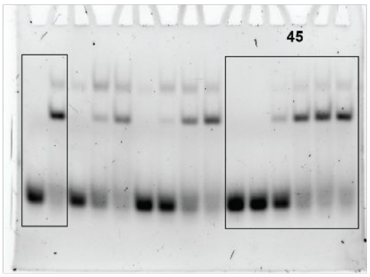

Fig 4F

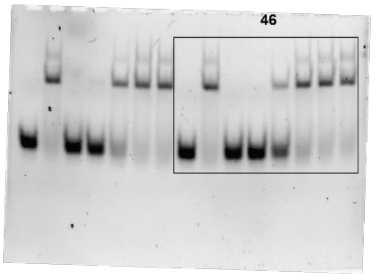

Fig 4F

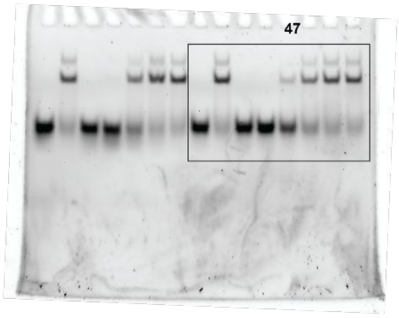

Fig 4F

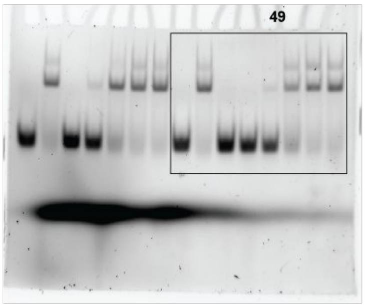

Figure S12. Uncropped gel images related to data shown in Figures 2 and 4 in the main manuscript.

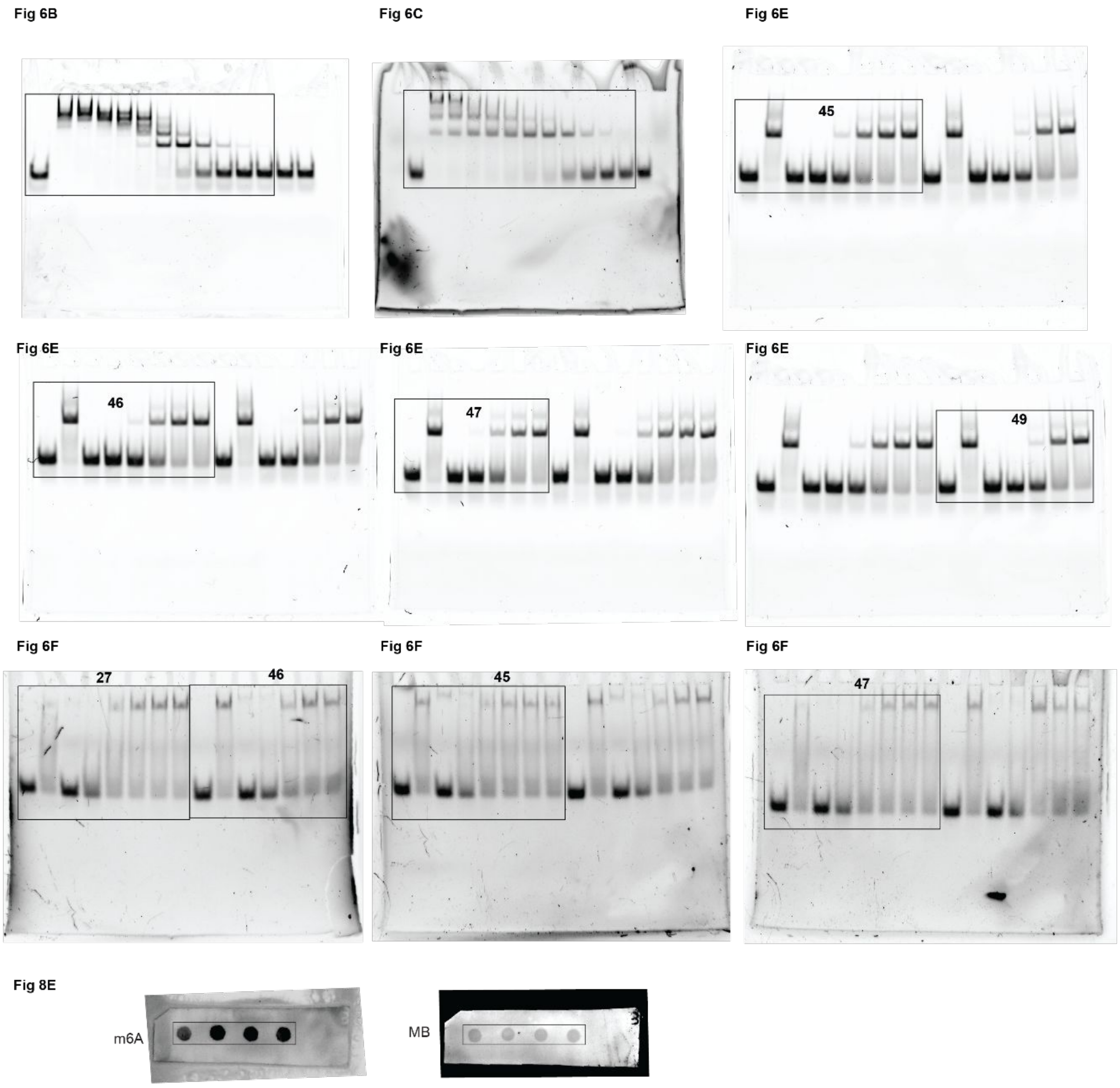

Figure S13. Uncropped gel images and blot images related to data shown in Figures 6 and 8 in the main manuscript.

SUPPLEMENTARY TABLE

Table S1, Sequence of used primers and oligonucleotides [1]

Primers/oligos	Sequence (5'-3')	Experiment	Source
288-METTL16_M1_Fwd	AAGTTCTGTTTCAGGGCCCGATGGCTCT GAGTAAATCAATGCATGCAAG	subclone	IDT
290-METTL16_D291_Rev	ATGGTCTAGAAAGCTTTAATCATAAAAAC TCCAAGCTAAGGCCCATCTC	subclone	IDT
FAM-*MAT2A*-hp1	FAM-CUUGUUGGCGUAGGCUACAGAGA AGCCUUCAAG	FP, EMSA	IDT
MAT2A-hp1	CUUGUUGGCGUAGGCUACAGAGAAGC CUUCAAG	MTase Glo	IDT
Cy5-U6 snRNA Δ	Cy5- GGAUACAGAGAAGAUUAGCAUGGCCCC UGCGCAAGGAUGACACGCAAAUUCGU	FP, EMSA	IDT
FAM- GGACU-RNA	FAM-UUCUUCUGUGGACUGUG	FP, EMSA	IDT
GGACU-RNA	UUCUUCUGUGGACUGUG	MTase Glo	IDT
Pre-miR-17-hp	GUGCAGGUAGUGAUAUGUGCAUCUACU GCAC	FP	IDT
Pre-miR-17-hp bp	GUGCAGGUAGAUGAUAUGUGCAUCUAC CUGCAC	FP	IDT
Pre-miR-21-hp	GGGUUGACUGUUGAAUCUCAUGGCAAC CC	FP	IDT
*GAPDH*_Fwd	GAAGGTGAAGGTCGGAGTC	RT-qPCR	IDT
*GAPDH*_Rev	GAAGATGGTGATGGGATTTC	RT-qPCR	IDT
*MAT2A*_spliced_Fwd	GAATTTCGATCTCCGCCCTG	RT-qPCR	IDT
*MAT2A*_spliced_Rev	AGCCTACGCCAACAAGTCTG	RT-qPCR	IDT

[1]Note: RNAs were pre-folded by heating at 95 ℃ for 3 min and then slowly cooled to room temperature.

SUPPLEMENTARY METHODS

Docking analysis

For computational docking analysis of aminothiazolones to the RNA binding site of METTL16 (PDB code: 6B91)[2] Schrödinger® Maestro 12.3 was used. The three-dimensional structures of compounds were prepared after calculating energy minimization by MM2 with PerkinElmer Chem3D® 22.2. Chemical states were generated with the ligand preparation module and METTL16 conformation with the protein preparation module. Later included hydrogen addition, water molecule removal and energy minimization. Crucial interactions of *MAT2A* 3'UTR with METTL16 were identified based on the resolved structure of the RNP complex.[3].[3] The residues crucial for RNA binding were used to identify binding sites for the 4-thiazolidinones. The binding site for docking was then generated by the grid generation module and using crucial residues of METTL16 involved in the binding of *MAT2A* 3'UTR. The glide dock module was used and the results were evaluated according to interactions between small molecules and METTL16, small molecule orientations, docking scores and solvent exposure patterns. The interactions of 4-thiazolidinones and METTL16 were visualized using PyMOL 2.5.2.

Protein mass spectrometry

METTL16 MTD protein was diluted in SEC buffer (20mM pH 7.5, 200 mM NaCl, 0.5 mM TCEP,5% v/v glycerol) to a final concentration of 30 µM, and incubated with DMSO or 100 µM compound (final 1% DMSO) at room temperature. After incubation (30 min or 150 min), the samples were analyzed on an Agilent 1260 II Infinity system equipped with an electrospray ion source in positive mode, run through a Desalting cartridge (AdvanceBio Desalting-RP, 2.1 mm, 12.5mm, Agilent) with a gradient of 5-80% HPLC-grade acetonitrile (+ 0.1% TFA) in HPLC-grade water (+ 0.1% TFA) (flowrate: 0.4 mL/min, runtime: 6 min). The Spectra were deconvoluted using the ProMass software (Enovatia).

GENERAL CHEMISTRY INFORMATION

Unless otherwise mentioned, all commercially available compounds were used without any further purification. Solvents used for silica gel column chromatography were laboratory grade. Dry solvents were purchased from Acros, Fischer Scientific and/or VWR and used without further treatment. Oxygen and/or moisture-sensitive solutions were transferred under an inert gas atmosphere using cannulas and syringes. Analytical thin-layer chromatography (TLC) was performed on silica-coated aluminum plates (Merck 60 F254) and visualization of products proceeded with UV irradiation (254 nm and/or 356 nm) or through potassium permanganate stain (1.5 g $KMnO_4$, 10 g K_2CO_3 in 1.25 mL of 10% aq. NaOH and 200 mL water). Analytical uHPLC-MS was performed on an Agilent 1260 II Infinity system equipped with a mass detector (uHPLC column: Zorbax Eclipse C18 Rapid Resolution 2.1x1x50 mm 1.8µm; LC-MS column: InfinityLab Poroshell 120 EC-C18, 2.1x150, 2.7 µm). To analyze intermediates and final products, a gradient was applied starting from 10% acetonitrile (+0.1% TFA) in water (+ 0.1% TFA) up to 99.9% acetonitrile (+0.1% TFA) (flowrate: 0.5 mL/min, maximum pressure: 800 bar). The purification of crude products was performed by silica gel column chromatography (Merck 60, particle size 0.040-0.063 mm) using indicated solvents. ^{1}H NMR and ^{13}C NMR spectra were recorded with either a Bruker AV 400 Avance III HD (NanoBay), Agilent Technologies DD2, Bruker AV 500 Avance III HD (Prodigy), Bruker Avance NEO – 500 MHz, Bruker AV 600 Avance III HD (CryoProbe) or a Bruker AV 700 Avance III HD (CryoProbe) spectrometers. Data is reported in parts per million (ppm) with reference to the used deuterated solvent ($CDCl_3$: 7.26 ppm, 77.16 ppm; DMSO-d_6: 2.50 ppm, 39.52 ppm). Chemical shift values are reported in ppm, multiplicity (s = singlet, d = doublet, t = triplet, dd = double doublet and m = multiplet), integration values, and coupling constant values in Hz. Signals were assigned to their corresponding hydrogens or carbons based on 2D NMR correlations (^{1}H/^{1}H COSY, ^{1}H/^{1}H NOESY, ^{1}H/^{13}C HSQC, ^{1}H/^{13}C HMBC). To facilitate accurate analysis of fluoride containing compounds, ^{19}F NMR analyses without {^{1}H} decoupling were conducted. High-resolution mass spectrometry (HRMS) was performed on an LTQ Orbitrap mass spectrometer coupled to an Accela HPLC-System (HPLC column: Hypersyl GOLD, 50 mm x 1 mm, particle size 1.9 µm, ionization method: electron spray ionization (ESI)).

General Procedure A (thiazolidinonesulfonamide formation)

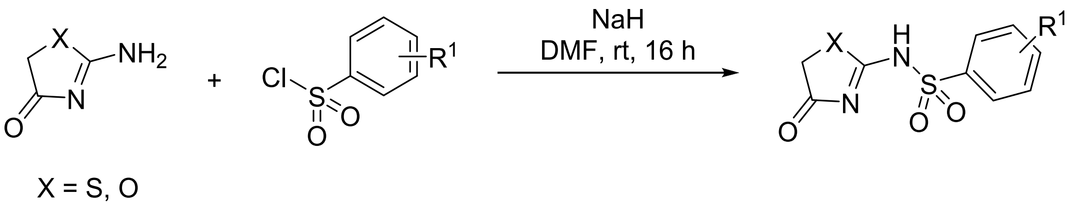

An excess of pseudothiohydantoin (X = S) or 2-imino-4-oxazolidinone (X = O) (3 equiv) was suspended in DMF and sodium hydride (3 equiv; 60% dispersion in mineral oil) was added portion-wise. After stirring for 30 min at room temperature the sulfonyl chloride was added to the milky solution and the reaction mixture was stirred for an additional 16 h. Then 1 M HCl was slowly added to form a precipitate which was isolated *via* vacuum filtration by using a sintered glass funnel. The solid was washed with 1 M HCl, water, ethanol and finally diethyl ether. Subsequently, the solid was dried under a high vacuum to afford the desired sulfonamides. In case no precipitate formed after adding 1 M HCl, the solution was extracted using EtOAc. The organic layer was collected, dried over anhydrous $MgSO_4$, and the solvent was removed under reduced pressure. The crude product was then purified *via* silica gel column chromatography using indicated solvent gradients.[4]

_N_-(4-oxo-4,5-dihydrothiazol-2-yl)benzenesulfonamide (Ia)

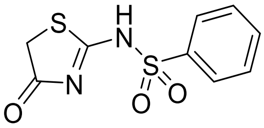

Following the general procedure A, the product was obtained as a beige solid after filtration and washing using 1 M HCl, water, ethanol and diethyl ether (143.7 mg, 0.56 mmol, 99%). **^{1}H NMR** (700 MHz, DMSO-d_6) δ 12.54 (s, 1H), 7.92 – 7.83 (m, 2H), 7.73 – 7.67 (m, 1H), 7.67 – 7.58 (m, 2H), 4.06 (s, 2H). **^{13}C NMR** (176 MHz, DMSO-d_6) δ 174.24, 173.42, 141.02, 133.68, 129.81, 126.86, 35.48.

4-methyl-_N_-(4-oxo-4,5-dihydrothiazol-2-yl)benzenesulfonamide (Ib)

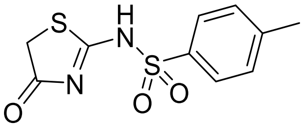

Following the general procedure A, the product was obtained as a beige solid after filtration and washing using 1 M HCl, water, ethanol and diethyl ether (36.2 mg, 0.13 mmol, 51%).

^{1}H NMR (600 MHz, DMSO-d_6) δ 12.50 (s, 1H), 7.79 – 7.71 (m, 2H), 7.43 (d, J = 8.0 Hz, 2H), 4.05 (s, 2H), 2.40 (s, 3H). **^{13}C NMR** (151 MHz, DMSO-d_6) δ 174.22, 173.00, 144.12, 138.19, 130.20, 126.94, 35.42, 21.51.

4-methoxy-*N*-(4-oxo-4,5-dihydrothiazol-2-yl)benzenesulfonamide (Ic)

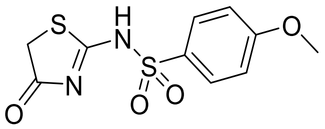

Following the general procedure A, the product was obtained as a beige solid after filtration and washing using 1 M HCl, water, ethanol and diethyl ether (770.0 mg, 2.69 mmol, 74%). **^{1}H NMR** (700 MHz, DMSO-d_6) δ 12.46 (s, 1H), 7.81 – 7.77 (m, 2H), 7.16 – 7.11 (m, 2H), 4.04 (s, 2H), 3.85 (s, 3H). **^{13}C NMR** (176 MHz, DMSO-d_6) δ 174.20, 172.46, 163.24, 132.67, 129.16, 114.94, 56.23, 35.36.

3,5-difluoro-*N*-(4-oxo-4,5-dihydrothiazol-2-yl)benzenesulfonamide (Id)

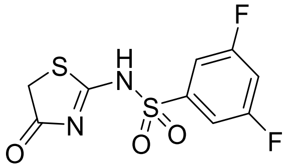

Following the general procedure A, the product was obtained as a yellow solid after filtration and washing using 1 M HCl, water, ethanol and diethyl ether (82.2 mg, 0.28 mmol, 64%). **^{1}H NMR** (600 MHz, DMSO-d_6) δ 12.69 (s, 1H), 7.69 (tt, J = 9.2, 2.3 Hz, 1H), 7.60 – 7.53 (m, 2H), 4.08 (s, 2H). **^{13}C NMR** (151 MHz, DMSO-d_6) δ 175.50, 174.36, 163.49 (d, J = 12.2 Hz), 161.82 (d, J = 12.2 Hz), 144.28 (t, J = 8.6 Hz), 110.77 (d, J = 6.6 Hz), 110.62 (d, J = 6.6 Hz), 109.49 (t, J = 25.7 Hz), 35.82.

3-nitro-*N*-(4-oxo-4,5-dihydrothiazol-2-yl)benzenesulfonamide (Ie)

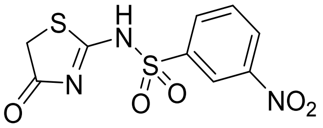

Following the general procedure A, the product was obtained as a yellow solid after filtration and washing using 1 M HCl, water, ethanol and diethyl ether (92.5 mg, 0.31 mmol, 69%). **^{1}H NMR** (700 MHz, DMSO-d_6) δ 12.71 (s, 1H), 8.54 (ddd, J = 8.2, 2.3, 1.0 Hz, 1H), 8.52 (t, J = 2.0 Hz, 1H), 8.30 (ddd, J = 7.8, 1.8, 1.0 Hz, 1H), 7.94 (t, J = 8.0 Hz, 1H), 4.08 (s, 2H). **^{13}C**

NMR (151 MHz, DMSO-d_6) δ 175.32, 174.32, 148.31, 142.51, 132.89, 132.06, 128.28, 121.48, 35.84.

4-(*tert*-butyl)-*N*-(4-oxo-4,5-dihydrothiazol-2-yl)benzenesulfonamide (If)

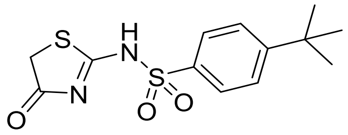

Following the general procedure A, the product was obtained as a white solid after filtration and washing using 1 M HCl, water, ethanol and diethyl ether (123.6 mg, 0.4 mmol, 92%).
^{1}H NMR (600 MHz, DMSO-d_6) δ 12.49 (s, 1H), 7.81 – 7.76 (m, 2H), 7.67 – 7.61 (m, 2H), 4.05 (s, 2H), 1.31 (s, 9H). **^{13}C NMR** (151 MHz, DMSO-d_6) δ 174.24, 173.02, 156.76, 138.21, 126.81, 126.64, 40.53, 35.42, 35.41, 31.24.

2-chloro-4-fluoro-*N*-(4-oxo-4,5-dihydrothiazol-2-yl)benzenesulfonamide (Ig)

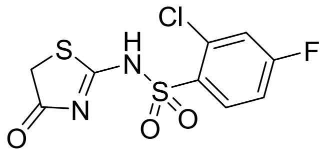

Following the general procedure A, the product was obtained as a yellow solid after filtration and washing using 1 M HCl, water, ethanol and diethyl ether (107.4 mg, 0.35 mmol, 79%).
^{1}H NMR (600 MHz, DMSO-d_6) δ 12.67 (s, 1H), 8.14 (dd, J = 8.9, 5.9 Hz, 1H), 7.76 (dd, J = 8.9, 2.6 Hz, 1H), 7.47 (ddd, J = 8.9, 8.0, 2.6 Hz, 1H), 4.10 (s, 2H). **^{13}C NMR** (151 MHz, DMSO-d_6) δ 174.79, 174.30, 165.44, 163.75, 134.99 (d, J = 3.3 Hz), 133.84 (d, J = 11.6 Hz), 132.68 (d, J = 10.3 Hz), 119.97 (d, J = 26.2 Hz), 115.41 (d, J = 21.9 Hz), 35.78.

4-cyclohexyl-*N*-(4-oxo-4,5-dihydrothiazol-2-yl)benzenesulfonamide (Ih)

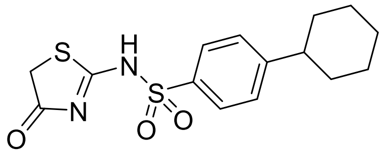

Following the general procedure A, the product was obtained as a yellow solid after filtration and washing using 1 M HCl, water, ethanol and diethyl ether (78.0 mg, 0.23 mmol, 59%).
^{1}H NMR (700 MHz, DMSO-d_6) δ 12.48 (s, 1H), 7.77 (d, J = 8.0 Hz, 2H), 7.47 (d, J = 8.0 Hz, 2H), 4.05 (s, 2H), 2.60 (d, J = 11.6 Hz, 1H), 1.79 (d, J = 11.6 Hz, 4H), 1.71 (d, J = 13.4 Hz, 1H), 1.40 (tt, J = 13.4, 12.4 Hz, 4H), 1.23 (t, J = 12.4 Hz, 1H). **^{13}C NMR** (176 MHz, DMSO-d_6) δ 174.22, 172.95, 153.65, 138.52, 128.07, 127.04, 44.12, 35.40, 33.97, 26.61, 25.90.

N-(4-oxo-4,5-dihydrothiazol-2-yl)-[1,1'-biphenyl]-4-sulfonamide (Ii)

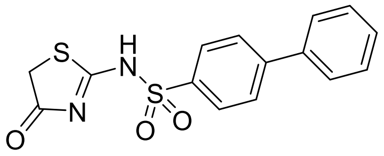

Following the general procedure A, the product was obtained as a yellow solid after filtration and washing using 1 M HCl, water, ethanol and diethyl ether (86.4 mg, 0.26 mmol, 65%).
^{1}H NMR (700 MHz, DMSO-d_6) δ 12.56 (s, 1H), 7.92 (p, J = 10.3 Hz, 4H), 7.74 (t, J = 10.3 Hz, 2H), 7.60 – 7.39 (m, 3H), 4.07 (s, 2H). **^{13}C NMR** (176 MHz, DMSO-d_6) δ 174.25, 173.46, 145.19, 139.72, 138.90, 129.64, 129.12, 128.02, 127.62, 127.58, 55.82, 35.51.

4-(N-(4-oxo-4,5-dihydrothiazol-2-yl)sulfamoyl)benzoic acid (Ij)

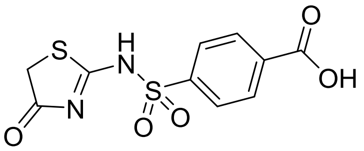

Following the general procedure A, the product was obtained as an orange solid after filtration and washing using 1 M HCl, water and diethyl ether (130.4 mg, 0.43 mmol, 50%).
^{1}H NMR (700 MHz, DMSO-d_6) δ 13.39 (s, 1H), 12.62 (s, 1H), 8.31-8.13 (m, 2H), 8.07-7.96 (m, 2H), 4.07 (s, 2H). **^{13}C NMR** (176 MHz, DMSO-d_6) δ 174.25, 166.58, 144.40, 135.22, 130.67, 130.49, 127.29, 127.21, 35.63.

5-bromo-2-methoxy-N-(4-oxo-4,5-dihydrothiazol-2-yl)benzenesulfonamide (Ik)

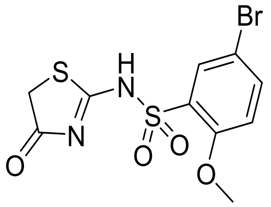

Following the general procedure A, the product was obtained as a yellow solid after filtration and washing using 1 M HCl, water, ethanol and diethyl ether (87.4 mg, 0.24 mmol, 68%).
^{1}H NMR (700 MHz, DMSO-d_6) δ 12.55 (s, 1H), 7.92 – 7.79 (m, 2H), 7.24 (d, J = 8.8 Hz, 1H), 4.10 (s, 2H), 3.89 (s, 3H). **^{13}C NMR** (176 MHz, DMSO-d_6) δ 174.43, 174.35, 156.70, 137.99, 130.99, 130.20, 116.26, 111.42, 57.19, 35.35.

N-(4-oxo-4,5-dihydrothiazol-2-yl)methanesulfonamide (Il)

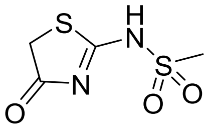

Following the general procedure A, the product was obtained as a yellow solid after filtration and washing using 1 M HCl, water and diethyl ether (112.4 mg, 0.58 mmol, 89%).

^{1}H NMR (600 MHz, DMSO-d_6) δ 12.44 (s, 1H), 4.05 (s, 2H), 3.07 (s, 3H). **^{13}C NMR** (151 MHz, DMSO-d_6) δ 174.34, 172.05, 41.75, 35.30.

***N*-(4-oxo-4,5-dihydrothiazol-2-yl)-4-(trifluoromethyl)benzenesulfonamide (Im)**

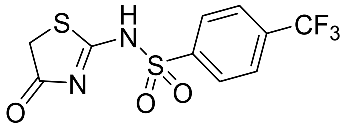

Following the general procedure A, the product was obtained as a yellow solid after filtration and washing using 1 M HCl, water, ethanol and diethyl ether (152.0 mg, 0.47 mmol, 77%).

^{1}H NMR (600 MHz, DMSO-d_6) δ 12.67 (s, 1H), 8.08 (d, J = 8.3 Hz, 2H), 8.02 (d, J = 8.3 Hz, 2H), 4.07 (s, 2H). **^{13}C NMR** (151 MHz, DMSO-d_6) δ 174.91, 174.28, 162.77, 144.80, 133.24 (q, J = 32.3 Hz), 127.92, 127.07 (q, J = 3.7 Hz), 123.89 (q, J = 272.9 Hz), 35.74.

***N*-(4-oxo-4,5-dihydrothiazol-2-yl)naphthalene-2-sulfonamide (In)**

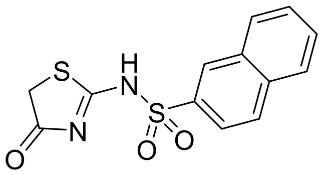

Following the general procedure A, the product was obtained as a beige solid after filtration and washing using 1 M HCl, water, ethanol and diethyl ether (162.9 mg, 0.53 mmol, 81%).

^{1}H NMR (700 MHz, DMSO-d_6) δ 12.56 (s, 1H), 8.56 (s, 1H), 8.22 (d, J = 8.2 Hz, 1H), 8.16 (d, J = 9.1 Hz, 1H), 8.07 (d, J = 8.2 Hz, 1H), 7.85 (d, J = 9.1 Hz, 1H), 7.71 (m, 2H), 4.07 (s, 2H). **^{13}C NMR** (176 MHz, DMSO-d_6) δ 174.23, 173.53, 137.96, 134.90, 132.08, 129.95, 129.87, 129.57, 128.34, 128.21, 127.78, 122.59, 35.53.

4-cyano-*N*-(4-oxo-4,5-dihydrothiazol-2-yl)benzenesulfonamide (Io)

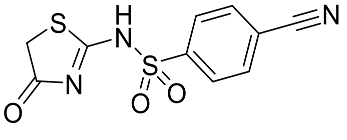

Following the general procedure A, the product was obtained as a yellow solid after filtration and washing using 1 M HCl, water, ethanol and diethyl ether (45.4 mg, 0.16 mmol, 32%).

^{1}H NMR (700 MHz, DMSO-d_6) δ 12.69 (s, 1H), 8.11 (d, J = 8.4 Hz, 2H), 8.03 (d, J = 8.4 Hz, 2H), 4.07 (s, 2H). **^{13}C NMR** (176 MHz, DMSO-d_6) δ 175.14, 174.31, 144.93, 134.03, 127.63, 118.11, 116.01, 35.78.

4-fluoro-*N*-(4-oxo-4,5-dihydrothiazol-2-yl)benzenesulfonamide (Ip)

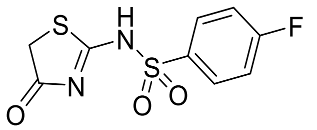

Following the general procedure A, the product was obtained as a yellow solid after filtration and washing using 1 M HCl, water, ethanol and diethyl ether (95.7 mg, 0.35 mmol, 68%).

^{1}H NMR (600 MHz, DMSO-d_6) δ 12.56 (s, 1H), 7.93 (dd, J = 8.9, 5.1 Hz, 2H), 7.47 (t, J = 8.9 Hz, 2H), 4.06 (s, 2H). **^{13}C NMR** (151 MHz, DMSO-d_6) δ 174.24, 173.69, 165.84, 164.17, 137.45 (d, J = 3.1 Hz), 130.00 (d, J = 9.5 Hz), 117.01 (d, J = 22.8 Hz), 35.55.

methyl 4-(*N*-(4-oxo-4,5-dihydrothiazol-2-yl)sulfamoyl)benzoate (Iq)

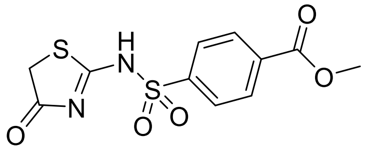

Following the general procedure A, the product was obtained as a yellow solid after filtration and washing using 1 M HCl, water, ethanol and diethyl ether (85.3 mg, 0.27 mmol, 68%).

^{1}H NMR (700 MHz, DMSO-d_6) δ 12.64 (s, 1H), 8.17 (d, J = 8.4 Hz, 2H), 8.00 (d, J = 8.4 Hz, 2H), 4.07 (s, 2H), 3.90 (s, 3H). **^{13}C NMR** (176 MHz, DMSO-d_6) δ 174.45, 174.26, 165.56, 144.86, 133.93, 130.61, 127.36, 53.17, 35.67.

3-(*N*-(4-oxo-4,5-dihydrothiazol-2-yl)sulfamoyl)benzoic acid (Ir)

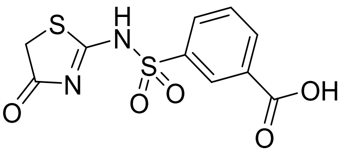

Following the general procedure A, the product was obtained as a yellow solid after purification using silica gel column chromatography (eluent system: MeOH in DCM (0-4%)) (85.3 mg, 0.27 mmol, 68%).

^{1}H NMR (500 MHz, DMSO-d_6) δ 12.63 (s, 2H), 8.64 (t, J = 1.8 Hz, 1H), 8.37 (dt, J = 7.8, 1.6 Hz, 1H), 8.10 (dt, J = 7.8, 1.6 Hz, 1H), 7.80 (t, J = 7.8 Hz, 1H), 4.07 (s, 2H). **^{13}C NMR** (126 MHz, DMSO-d_6) δ 174.35, 174.08, 141.54, 133.95, 131.05, 130.53, 127.56, 49.07, 35.69, 35.11.

4-methoxy-*N*-(4-oxo-4,5-dihydrooxazol-2-yl)benzenesulfonamide (Is)

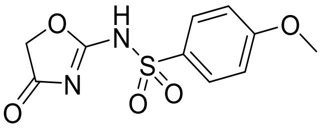

Following the general procedure A, the product was obtained as a white solid after filtration and washing using 1 M HCl, water and diethyl ether (102.5 mg, 0.4 mmol, 79%).

^{1}H NMR (600 MHz, DMSO-d_6) δ 12.53 (s, 1H), 7.85 – 7.74 (m, 2H), 7.15 – 7.03 (m, 2H), 4.91 (s, 2H), 3.84 (s, 3H). **^{13}C NMR** (151 MHz, DMSO-d_6) δ 173.55, 162.71, 161.57, 134.20, 129.21, 114.53 71.86, 56.12.

General Procedure B (thiazolidinoneamide formation)

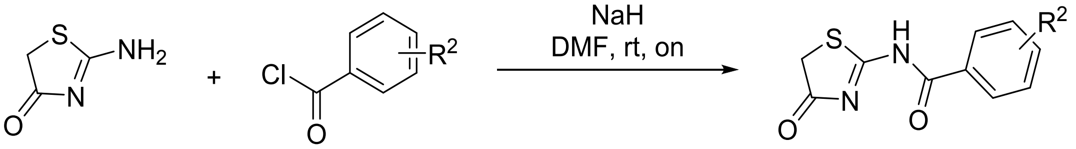

An excess of pseudothiohydantoin (3 equiv) was suspended in DMF and sodium hydride (3 equiv; 60% dispersion in mineral oil) was added portion-wise. After stirring for 30 min at room temperature the benzoyl chloride was added to the milky solution and the reaction mixture was stirred for additional 16 h. Then 1 M HCl was slowly added to form a precipitate which was isolated *via* vacuum filtration by using a sintered glass funnel. The solid was washed with 1 M HCl, water, ethanol and finally diethyl ether. Subsequently, the solid was dried under high vacuum to afford the desired sulfonamides. In case no precipitate formed after adding 1 M HCl, the solution was extracted using EtOAc. The organic layer was collected, dried over anhydrous

$MgSO_4$ and the solvent was removed under reduced pressure. The crude product was then purified *via* silica gel column chromatography using indicated solvent gradients.

4-methyl-*N*-(4-oxo-4,5-dihydrothiazol-2-yl)benzamide (IIa)

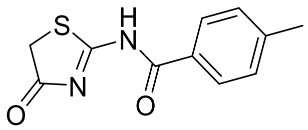

Following the general procedure B, the product was obtained as a yellow solid after filtration and washing using 1 M HCl, water, ethanol and diethyl ether (154.6 mg, 0.66 mmol, 100%).
^{1}H NMR (600 MHz, DMSO-d_6) δ 12.48 (s, 1H), 8.03 (d, J = 8.2 Hz, 2H), 7.34 (d, J = 8.2 Hz, 2H), 3.96 (s, 2H), 2.39 (s, 3H). **^{13}C NMR** (151 MHz, DMSO-d_6) δ 167.77, 165.84, 143.96, 130.29, 130.21, 129.79, 129.67, 128.61, 98.18, 35.10, 21.69.

4-methoxy-*N*-(4-oxo-4,5-dihydrothiazol-2-yl)benzamide (IIb)

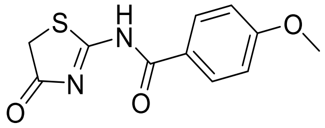

Following the general procedure B, the product was obtained as a yellow solid after filtration and washing using 1 M HCl, water, ethanol and diethyl ether (118.4 mg, 0.47 mmol, 64%).
^{1}H NMR (700 MHz, DMSO-d_6) δ 12.41 (s, 1H), 8.17 – 8.02 (m, 2H), 7.13 – 6.99 (m, 2H), 3.95 (s, 2H), 3.85 (s, 3H). **^{13}C NMR** (176 MHz, DMSO-d_6) δ 176.38, 175.41, 174.71, 163.66, 131.93, 128.19, 114.37, 56.00.

3,4-dimethoxy-*N*-(4-oxo-4,5-dihydrothiazol-2-yl)benzamide (IIc)

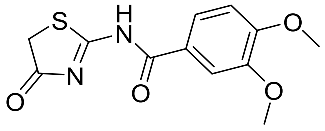

Following the general procedure B, the product was obtained as a yellow solid after filtration and washing using 1 M HCl, water, ethanol and diethyl ether (66.2 mg, 0.24 mmol, 36%).
^{1}H NMR (600 MHz, DMSO-d_6) δ 12.52 (s, 1H), 7.81 (dd, J = 8.4, 2.0 Hz, 1H), 7.65 (d, J = 2.0 Hz, 1H), 7.10 (d, J = 8.4 Hz, 1H), 3.94 (s, 2H), 3.85 (s, 3H), 3.83 (s, 3H).**^{13}C NMR** (151 MHz, DMSO-d_6) δ 167.57, 153.53, 148.89, 124.05, 123.64, 112.34, 112.13, 111.45, 56.20, 55.97.

4-fluoro-*N*-(4-oxo-4,5-dihydrothiazol-2-yl)benzamide (IId)

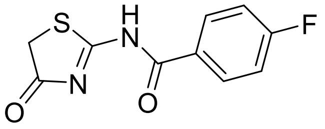

Following the general procedure B, the product was obtained as a yellow solid after filtration and washing using 1 M HCl, water, ethanol and diethyl ether (155.0 mg, 0.65 mmol, 99%).
^{1}H NMR (600 MHz, DMSO-d_6) δ 12.58 (s, 1H), 8.29 – 8.12 (m, 2H), 7.37 (t, *J* = 8.9 Hz, 2H), 3.98 (s, 2H). **^{13}C NMR** (151 MHz, DMSO-d_6) δ 166.83, 166.34, 164.67, 133.31 (d, *J* = 9.8 Hz), 132.55 (d, *J* = 9.4 Hz), 116.87 (d, *J* = 22.3 Hz), 116.14 (d, *J* = 21.9 Hz), 35.49 – 34.78 (m).

1-methyl-5-nitroindoline-2,3-dione (III)

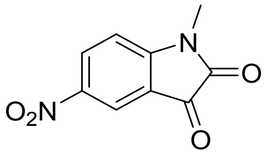

A mixture of 5-nitroindoline-2,3-dione (500 mg, 2.60 mmol, 1 equiv.) in DMF (10 mL) was cooled to 0 °C and NaH (60% dispersion in mineral oil, 122 mg, 3.04 mmol, 1.17 equiv.) was added and stirred for 5 minutes, followed by the dropwise addition of iodomethane (190 µL, 3.04 mmol, 1.17 equiv.). After two hours at rt, the reaction was poured onto NH_4Cl (aq., saturated) and extracted with EtOAc (3x). The combined organic layers were washed with brine and water, dried over $MgSO_4$. The solvents were removed under reduced pressure and purified by flash chromatography (DCM) to give 1-Methyl-5-nitroindoline-2,3-dione (484 mg, 2.35 mmol, 90%).
^{1}H NMR (500 MHz, DMSO-*d6*) δ 8.55 (dd, *J* = 8.8, 2.4 Hz, 1H), 8.23 (d, *J* = 2.4 Hz, 1H), 7.36 (d, *J* = 8.8 Hz, 1H), 3.22 (s, 3H). **^{13}C NMR** (126 MHz, DMSO) δ 181.73, 159.37, 156.13, 143.41, 133.48, 119.43, 118.31, 111.41, 26.97.

General Procedure C (Knoevenagel condensation)

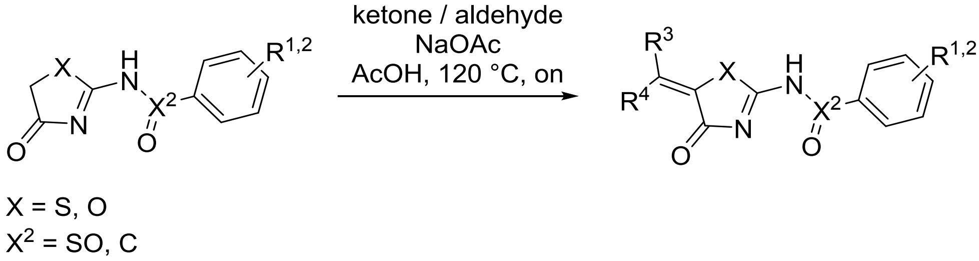

A mixture of ketone/aldehyde (1 equiv), sulfonamide/benzoyl chloride (1 equiv.) and sodium acetate (3 equiv.) in glacial acetic acid (0.06 M) was heated to reflux and then stirred for 16 h. After cooling to room temperature, the resulting precipitate was filtered and washed with water, ethanol and diethyl ether to afford the desired product.

_N_-(5-(5-chloro-2-oxoindolin-3-ylidene)-4-oxo-4,5-dihydrothiazol-2-yl)-4-methoxybenzenesulfonamide (1)

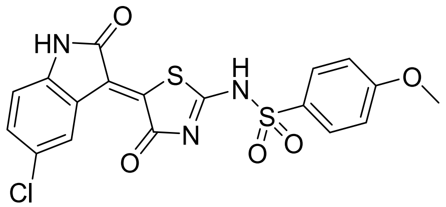

Following the general procedure C, the product was obtained as a red solid after filtration and washing using water, ethanol and diethyl ether (193.9 mg, 0.43 mmol, 53%).
^{1}H NMR (700 MHz, DMSO-d_6) δ 13.33 (s, 1H), 11.40 (s, 1H), 8.78 (d, J = 2.2 Hz, 1H), 7.91 – 7.79 (m, 2H), 7.44 (dd, J = 8.4, 2.2 Hz, 1H), 7.20 – 7.10 (m, 2H), 6.96 (d, J = 8.4 Hz, 1H), 3.85 (s, 3H).**^{13}C NMR** (176 MHz, DMSO-d_6) δ 168.72, 167.69, 167.69, 163.48, 143.16, 133.44, 132.46, 132.22, 129.32, 127.91, 126.64, 126.31, 121.71, 115.13, 112.39, 56.28. **HRMS-**ESI (*m/z*): calculated for $[M+H]^+$ $C_{18}H_{12}ClN_3O_5S_2$, 449.9979; found: 449.9981.

4-methoxy-_N_-(5-(5-methyl-2-oxoindolin-3-ylidene)-4-oxo-4,5-dihydrothiazol-2-yl)benzenesulfonamide (2)

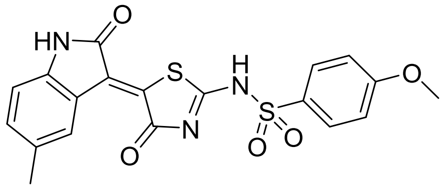

Following the general procedure C, the product was obtained as a red-brown solid after filtration and washing using water, ethanol and diethyl ether (51.8 mg, 0.12 mmol, 71%).
^{1}H NMR (600 MHz, DMSO-d_6) δ 13.20 (s, 1H), 11.15 (s, 1H), 8.59 (q, J = 0.9 Hz, 1H), 7.89 – 7.79 (m, 2H), 7.20 (ddd, J = 8.0, 1.8, 0.9 Hz, 1H), 7.18 – 7.13 (m, 2H), 6.83 (d, J = 8.0 Hz, 1H), 3.85 (s, 3H), 2.29 (s, 3H). **^{13}C NMR** (151 MHz, DMSO-d_6) δ 168.97, 168.11, 167.16, 163.41, 142.35, 133.75, 132.40, 131.25, 130.77, 129.26, 129.11, 128.29, 120.52, 115.09, 110.69, 56.27, 21.39. **HRMS-**ESI (*m/z*): calculated for $[M+Na]^+$ $C_{19}H_{15}N_3NaO_5S_2$, 452.0351 found: 452.0345.

***N*-(5-(5-methoxy-2-oxoindolin-3-ylidene)-4-oxo-4,5-dihydrothiazol-2-yl)benzenesulfonamide (3)**

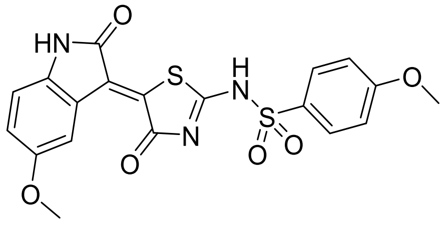

Following the general procedure C, the product was obtained as a red solid after filtration and washing using water, ethanol and diethyl ether (35.1 mg, 0.08 mmol, 47%).

^{1}H NMR (600 MHz, DMSO-d_6) δ 13.22 (s, 1H), 11.07 (s, 1H), 8.45 (d, J = 2.7 Hz, 1H), 7.87 – 7.81 (m, 2H), 7.18 – 7.14 (m, 2H), 7.00 (dd, J = 8.5, 2.7 Hz, 1H), 6.85 (d, J = 8.5 Hz, 1H), 3.85 (s, 3H), 3.75 (s, 3H). **^{13}C NMR** (151 MHz, DMSO-d_6) δ 168.91, 168.07, 167.32, 163.43, 155.05, 138.34, 132.36, 131.42, 129.27, 128.49, 121.06, 118.82, 115.10, 114.59, 111.32, 56.27, 55.96. **HRMS-**ESI (*m/z*): calculated for $[M+H]^+$ $C_{19}H_{15}N_3O_6S_2$, 446.0475; found: 446.0476.

4-methoxy-*N*-(5-(5-nitro-2-oxoindolin-3-ylidene)-4-oxo-4,5-dihydrothiazol-2-yl)benzenesulfonamide (4)

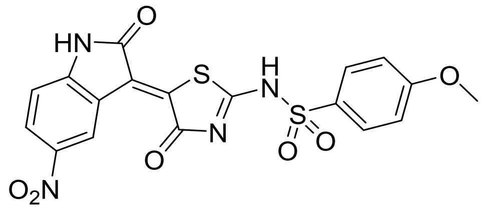

Following the general procedure C, the product was obtained as an orange solid after filtration and washing using water, ethanol and diethyl ether (54.5 mg, 0.12 mmol, 71%).

^{1}H NMR (600 MHz, DMSO-d_6) δ 13.38 (s, 1H), 11.94 (s, 1H), 9.65 (d, J = 2.5 Hz, 1H), 8.31 (dd, J = 8.7, 2.5 Hz, 1H), 7.88 – 7.82 (m, 2H), 7.19 – 7.15 (m, 2H), 7.13 (d, J = 8.7 Hz, 1H), 3.86 (s, 3H). **^{13}C NMR** (151 MHz, DMSO-d_6) δ 172.48, 169.34, 167.26, 163.53, 149.55, 142.69, 135.28, 132.10, 129.37, 128.79, 125.61, 123.83, 120.42, 115.15, 111.19, 56.29. **HRMS-**ESI (*m/z*): calculated for $[M+H]^+$ $C_{18}H_{12}N_4O_7S_2$, 461.0220; found: 461.0223.

4-methoxy-*N*-(5-(1-methyl-5-nitro-2-oxoindolin-3-ylidene)-4-oxo-4,5-dihydrothiazol-2-yl)benzenesulfonamide (5)

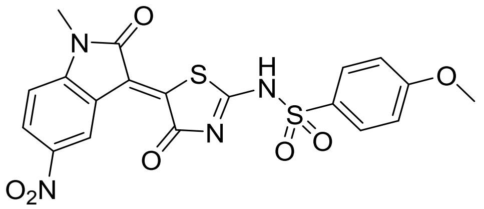

Following the general procedure C, the product was obtained as an orange-brown solid after filtration and washing using water, ethanol and diethyl ether (52.9 mg, 0.11 mmol, 77%).

^{1}H NMR (600 MHz, DMSO-d_6) δ 11.94 (s, 1H), 9.67 (dd, J = 10.7, 2.4 Hz, 1H), 8.40 (dd, J = 8.8, 2.4 Hz, 1H), 7.85 (dd, J = 8.8, 2.4 Hz, 2H), 7.37 (d, J = 8.8 Hz, 1H), 7.18 – 7.14 (m, 2H), 3.85 (s, 3H), 3.34 (s, 3H). **^{13}C NMR** (151 MHz, DMSO) δ 169.37, 167.96, 163.53, 150.15, 143.05, 132.11, 129.40, 129.37, 128.62, 123.83, 123.36, 119.74, 115.15, 111.20, 110.11, 56.30, 40.41, 40.27, 40.14, 40.00, 39.86, 39.72, 39.58, 27.40. **HRMS-**ESI (*m/z*): calculated for $[M+H]^+$ $C_{19}H_{13}N_4O_7S_2$, 475.0376; found: 475.0379.

4-methoxy-*N*-(4-oxo-5-(2-oxoindolin-3-ylidene)-4,5-dihydrothiazol-2-yl)benzenesulfonamide (6)

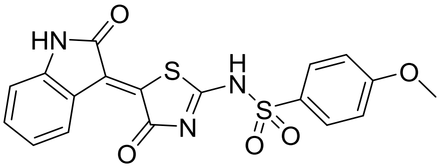

Following the general procedure C, the product was obtained as a red-brown solid after filtration and washing using water, ethanol and diethyl ether (48.0 mg, 0.12 mmol, 68%).

^{1}H NMR (600 MHz, DMSO-d_6) δ 13.21 (s, 1H), 11.27 (s, 1H), 8.79 – 8.74 (m, 1H), 7.87 – 7.82 (m, 2H), 7.40 (td, J = 7.7, 1.3 Hz, 1H), 7.18 – 7.14 (m, 2H), 7.07 (td, J = 7.7, 1.1 Hz, 1H), 6.95 (dt, J = 7.7, 0.9 Hz, 1H), 3.85 (s, 3H). **^{13}C NMR** (151 MHz, DMSO-d_6) δ 168.96, 168.06, 167.27, 163.42, 144.55, 133.31, 132.38, 131.21, 129.27, 128.73, 128.03, 122.57, 120.47, 115.10, 111.00, 56.27. **HRMS-**ESI (*m/z*): calculated for $[M+H]^+$ $C_{18}H_{13}N_3O_5S_2$, 416.0369; found: 416.0369.

4-methoxy-*N*-(5-(1-methyl-2-oxoindolin-3-ylidene)-4-oxo-4,5-dihydrothiazol-2-yl)benzenesulfonamide (7)

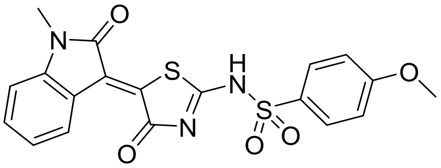

Following the general procedure C, the product was obtained as a red-brown solid after filtration and washing using water, ethanol and diethyl ether (41.1 mg, 0.1 mmol, 59%).

^{1}H NMR (600 MHz, DMSO-d_6) δ 13.25 (s, 1H), 8.77 (dd, J = 8.1, 1.2 Hz, 1H), 7.88 – 7.82 (m, 2H), 7.47 (td, J = 7.7, 1.2 Hz, 1H), 7.19 – 7.15 (m, 2H), 7.15 – 7.10 (m, 2H), 3.85 (s, 3H), 3.25 (s, 3H). **^{13}C NMR** (151 MHz, DMSO-d_6) δ 172.48, 167.83, 167.32, 163.45, 145.45, 133.18, 132.31, 131.86, 129.31, 128.40, 127.08, 123.10, 119.73, 115.11, 109.81, 56.28, 26.84. **HRMS-**ESI (*m/z*): calculated for $[M+Na]^+$ $C_{19}H_{15}N_3NaO_5S_2$, 452.0351; found: 452.0346.

***N*-(5-(5-bromo-2-oxoindolin-3-ylidene)-4-oxo-4,5-dihydrothiazol-2-yl)-4-methoxybenzenesulfonamide (8)**

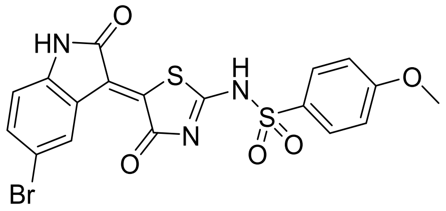

Following the general procedure C, the product was obtained as a red-brown solid after filtration and washing using water, ethanol and diethyl ether (71.1 mg, 0.14 mmol, 85%).

^{1}H NMR (600 MHz, DMSO-d_6) δ 13.30 (s, 1H), 11.40 (s, 1H), 8.92 (s, 1H), 7.84 (d, J = 8.5 Hz, 2H), 7.57 (d, J = 8.5 Hz, 1H), 7.16 (d, J = 8.5 Hz, 2H), 6.91 (d, J = 8.5 Hz, 1H), 3.85 (s, 3H).**^{13}C NMR** (151 MHz, DMSO-d_6) δ 168.61, 167.56, 167.41, 163.48, 143.51, 135.24, 133.45, 132.23, 130.70, 129.32, 126.50, 122.19, 115.13, 114.03, 112.87, 56.28. **HRMS-**ESI (*m/z*): calculated for $[M+H]^+$ $C_{18}H_{12}{}^{79}BrN_3O_5S_2$, 493.9480; found: 493.9457. Calculated for $[M+H]^+$ $C_{18}H_{12}{}^{81}BrN_3O_5S_2$, 495.9460; found: 495.9457.

***N*-(5-(5-bromo-1-methyl-2-oxoindolin-3-ylidene)-4-oxo-4,5-dihydrothiazol-2-yl)-4-methoxybenzenesulfonamide (9)**

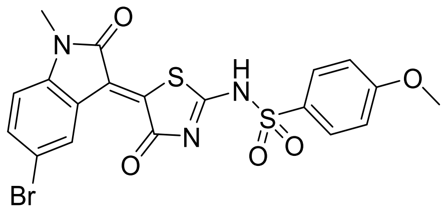

Following the general procedure C, the product was obtained as a red-brown solid after filtration and washing using water, ethanol and diethyl ether (72.5 mg, 0.14 mmol, 82%).

^{1}H NMR (600 MHz, DMSO-d_6) δ 13.36 (s, 1H), 8.92 (d, J = 2.1 Hz, 1H), 7.90 – 7.82 (m, 2H), 7.66 (dd, J = 8.4, 2.1 Hz, 1H), 7.19 – 7.15 (m, 2H), 7.11 (d, J = 8.4 Hz, 1H), 3.85 (s, 3H), 3.24

(s, 3H). **^{13}C NMR** (151 MHz, DMSO-d_6) δ 172.48, 167.46, 166.99, 163.50, 144.44, 135.06, 134.07, 132.17, 130.40, 129.36, 125.50, 121.37, 115.13, 114.69, 111.74, 56.29, 26.99. **HRMS-**ESI (*m/z*): calculated for $[M+H]^+$ $C_{19}H_{14}{}^{79}BrN_3O_5S_2$, 507.9631; found: 507.9635. Calculated for $[M+H]^+$ $C_{19}H_{14}{}^{81}BrN_3O_5S_2$, 509.9610; found: 509.9613.

4-methoxy-*N*-(4-oxo-5-(2-oxo-1-phenylindolin-3-ylidene)-4,5-dihydrothiazol-2-yl)benzenesulfonamide (10)

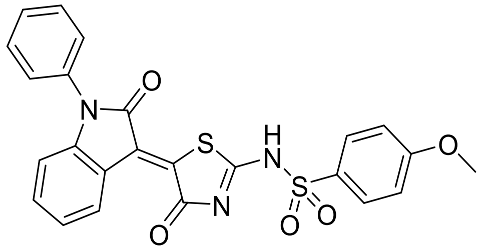

Following the general procedure C, the product was obtained as a red-brown solid after filtration and washing using water, ethanol and diethyl ether (59.0 mg, 0.12 mmol, 71%).

^{1}H NMR (600 MHz, DMSO-d_6) δ 13.32 (s, 1H), 8.91 (dd, J = 8.0, 1.2 Hz, 1H), 7.89 – 7.79 (m, 2H), 7.68 – 7.59 (m, 2H), 7.58 – 7.49 (m, 3H), 7.42 (td, J = 7.7, 1.3 Hz, 1H), 7.20 (td, J = 7.7, 1.2 Hz, 1H), 7.18 – 7.13 (m, 2H), 6.82 (dt, J = 7.9, 0.8 Hz, 1H), 3.85 (s, 3H). **^{13}C NMR** (151 MHz, DMSO-d_6) δ 172.48, 167.64, 167.24 – 167.09 (m), 166.98, 163.47, 145.20, 133.76, 133.18, 132.89 – 132.68 (m), 132.24, 130.25, 129.32, 129.15, 128.77, 127.36, 126.88, 123.69, 119.98, 115.12, 110.12, 56.28. **HRMS-**ESI (*m/z*): calculated for $[M+H]^+$ $C_{24}H_{17}N_3O_5S_2$, 492.02682; found: 492.0685.

***N*-(5-(7-fluoro-2-oxoindolin-3-ylidene)-4-oxo-4,5-dihydrothiazol-2-yl)-4-methoxybenzenesulfonamide (11)**

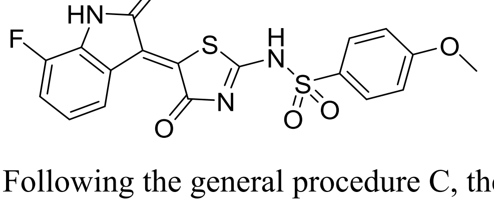

Following the general procedure C, the product was obtained as a red solid after filtration and washing using water, ethanol and diethyl ether (46.3 mg, 0.11 mmol, 65%).

^{1}H NMR (600 MHz, DMSO-d_6) δ 13.28 (s, 1H), 11.80 (s, 1H), 8.62 (dt, J = 7.9, 0.8 Hz, 1H), 7.87 – 7.82 (m, 2H), 7.37-7.33 (m, 1H), 7.19 – 7.14 (m, 2H), 7.09 (td, J = 8.2, 5.1 Hz, 1H), 3.85 (s, 3H). **^{13}C NMR** (151 MHz, DMSO-d_6) δ 168.81, 163.46, 147.86, 146.25, 132.28, 131.50 (d, J = 13.1 Hz), 129.31, 127.00, 124.68, 123.11 (dd, J = 7.5, 4.9 Hz), 119.69 (d, J =

16.7 Hz), 115.12, 56.28. **^{19}F NMR** (470 MHz, DMSO-d_6) δ -132.68 (dd, J = 10.0, 5.1 Hz). **HRMS-**ESI (*m/z*): calculated for $[M+Na]^+$ $C_{18}H_{12}FN_3NaO_5S_2$, 456.0100; found: 456.0095.

***N*-(5-(6-fluoro-2-oxoindolin-3-ylidene)-4-oxo-4,5-dihydrothiazol-2-yl)-4-methoxybenzenesulfonamide (12)**

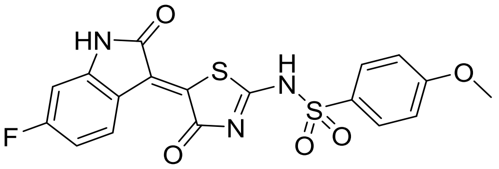

Following the general procedure C, the product was obtained as a red solid after filtration and washing using water, ethanol and diethyl ether (61.2 mg, 0.14 mmol, 83%).

^{1}H NMR (600 MHz, DMSO-d_6) δ 13.31 (s, 1H), 11.29 (s, 1H), 8.55 (dd, J = 10.1, 2.8 Hz, 1H), 7.87 – 7.82 (m, 2H), 7.27 (td, J = 8.8, 2.8 Hz, 1H), 7.19 – 7.14 (m, 2H), 6.94 (dd, J = 8.8, 4.6 Hz, 1H), 3.85 (s, 3H). **^{13}C NMR** (151 MHz, DMSO-d_6) δ 169.33, 167.92, 167.50 – 167.28 (m), 165.71, 164.05, 163.44, 146.69 (d, J = 12.6 Hz), 132.33, 130.98 (d, J = 10.1 Hz), 129.28, 126.87, 117.18, 115.11, 109.16 (d, J = 22.3 Hz), 99.09 (d, J = 27.4 Hz), 56.28. **^{19}F NMR** (565 MHz, DMSO-d_6) δ -104.23. **HRMS-**ESI (*m/z*): calculated for $[M+H]^+$ $C_{18}H_{12}FN_3O_5S_2$, 434.0275; found: 434.0271.

***N*-(5-(5-fluoro-2-oxoindolin-3-ylidene)-4-oxo-4,5-dihydrothiazol-2-yl)-4-methoxybenzenesulfonamide (13)**

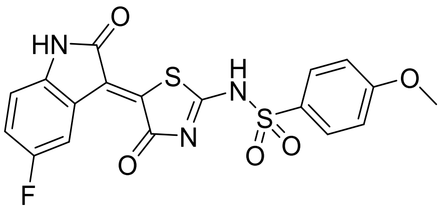

Following the general procedure C, the product was obtained as a red solid after filtration and washing using water, ethanol and diethyl ether (38.6 mg, 0.09 mmol, 52%).

^{1}H NMR (600 MHz, DMSO-d_6) δ 13.23 (s, 1H), 11.44 (s, 1H), 8.81 (dd, J = 8.9, 5.8 Hz, 1H), 7.86 – 7.82 (m, 2H), 7.18 – 7.14 (m, 2H), 6.90 (td, J = 9.1, 2.3 Hz, 1H), 6.77 (dd, J = 8.9, 2.3 Hz, 1H), 3.85 (s, 3H).**^{13}C NMR** (151 MHz, DMSO-d_6) δ 168.93, 167.76, 167.42, 163.48, 158.70, 157.14, 140.89, 132.24, 129.31, 127.30, 127.28, 121.10 (d, J = 10.3 Hz), 119.57 (d, J = 24.2 Hz), 115.31, 115.12, 111.77 (d, J = 8.2 Hz), 56.28, 40.53. **^{19}F NMR** (470 MHz, DMSO-d_6) δ -121.06 (td, J = 9.4, 4.4 Hz). **HRMS-**ESI (*m/z*): calculated for $[M+H]^+$ $C_{18}H_{12}FN_3O_5S_2$, 434.0275; found: 434.0273.

***N*-(5-(7-chloro-2-oxoindolin-3-ylidene)-4-oxo-4,5-dihydrothiazol-2-yl)-4-methoxybenzenesulfonamide (14)**

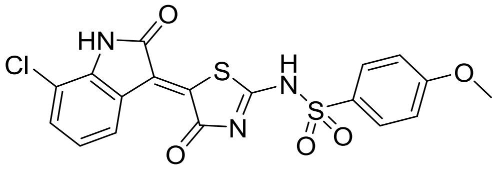

Following the general procedure C, the product was obtained as a red solid after filtration and washing using water, ethanol and diethyl ether (64.8 mg, 0.14 mmol, 85%).

^{1}H NMR (600 MHz, DMSO-d_6) δ 13.29 (s, 1H), 11.69 (s, 1H), 8.74 (d, J = 8.0 Hz, 1H), 7.88 – 7.82 (m, 2H), 7.48 (dt, J = 8.2, 1.2 Hz, 1H), 7.18 – 7.14 (m, 2H), 7.10 (td, J = 8.0, 1.0 Hz, 1H), 3.85 (s, 3H).**^{13}C NMR** (151 MHz, DMSO-d_6) δ 168.96, 167.63, 167.17, 163.47, 141.81, 133.47, 132.56, 132.26, 129.31, 127.12, 123.62, 122.18, 115.15, 115.12, 56.28. **HRMS-**ESI (*m/z*): calculated for $[M+H]^+$ $C_{18}H_{12}ClN_3O_5S_2$, 449.9985; found: 449.9974.

***N*-(5-(6-chloro-2-oxoindolin-3-ylidene)-4-oxo-4,5-dihydrothiazol-2-yl)-4-methoxybenzenesulfonamide (15)**

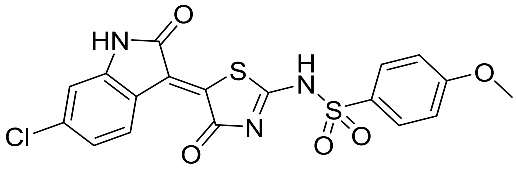

Following the general procedure C, the product was obtained as a red solid after filtration and washing using water, ethanol and diethyl ether (61.7 mg, 0.14 mmol, 81%).

^{1}H NMR (600 MHz, DMSO-d_6) δ 13.27 (s, 1H), 11.42 (s, 1H), 8.73 (d, J = 8.5 Hz, 1H), 7.88 – 7.77 (m, 2H), 7.18 – 7.14 (m, 2H), 7.13 (dd, J = 8.5, 2.0 Hz, 1H), 6.95 (d, J = 2.0 Hz, 1H), 3.85 (s, 3H).**^{13}C NMR** (151 MHz, DMSO-d_6) δ 168.98, 167.76, 167.28, 163.46, 145.74, 137.15, 132.28, 132.11, 129.93, 129.30, 126.68, 122.39, 119.35, 115.11, 110.96, 56.28. **HRMS-**ESI (*m/z*): calculated for $[M+H]^+$ $C_{18}H_{12}ClN_3O_5S_2$, 449.9985; found: 449.9980.

***N*-(5-(5-fluoro-1-methyl-2-oxoindolin-3-ylidene)-4-oxo-4,5-dihydrothiazol-2-yl)-4-methoxybenzenesulfonamide (16)**

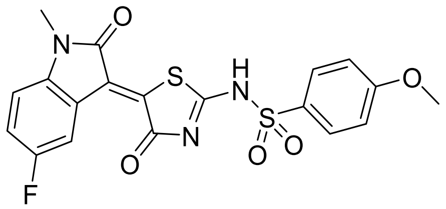

Following the general procedure C, the product was obtained as a red solid after filtration and washing using water, ethanol and diethyl ether (61.1 mg, 0.14 mmol, 82%).

^{1}H NMR (700 MHz, DMSO-d_6) δ 13.34 (s, 1H), 8.58 (dd, J = 10.0, 2.7 Hz, 1H), 7.91 – 7.79 (m, 2H), 7.36 (td, J = 8.8, 2.7 Hz, 1H), 7.18 – 7.12 (m, 3H), 3.85 (s, 3H), 3.25 (s, 3H). **^{13}C NMR** (176 MHz, DMSO-d_6) δ 167.22, 163.49, 159.04, 157.70, 141.84, 132.19, 129.34, 120.41 (d, J = 10.1 Hz), 119.23 (d, J = 24.6 Hz), 115.24, 115.13, 115.08, 110.70 (d, J = 8.1 Hz), 56.29, 27.02. **^{19}F NMR** (470 MHz, DMSO-d_6) δ -120.54 (td, J = 9.3, 4.3 Hz). **HRMS-**ESI (*m/z*): calculated for $[M+H]^+$ $C_{19}H_{14}FN_3O_5S_2$, 448.0431; found: 448.0433.

***N*-(5-(6-fluoro-1-methyl-2-oxoindolin-3-ylidene)-4-oxo-4,5-dihydrothiazol-2-yl)-4-methoxybenzenesulfonamide (17)**

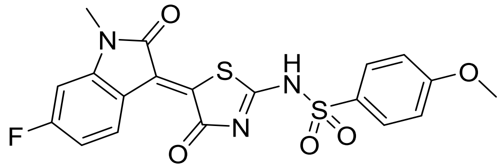

Following the general procedure C, the product was obtained as a red solid after filtration and washing using water, ethanol and diethyl ether (50.0 mg, 0.11 mmol, 67%).

^{1}H NMR (600 MHz, DMSO-d_6) δ 13.25 (s, 1H), 8.83 (dd, J = 8.7, 5.9 Hz, 1H), 7.88 – 7.80 (m, 2H), 7.20 – 7.11 (m, 3H), 6.96 (ddd, J = 9.5, 8.7, 2.5 Hz, 1H), 3.84 (s, 3H), 3.25 (s, 3H). **^{13}C NMR** (151 MHz, DMSO-d_6) δ 167.83, 165.96, 164.30, 163.45, 147.85 (d, J = 12.5 Hz), 132.29, 130.58 (d, J = 10.0 Hz), 129.30, 116.34, 115.11, 109.32 (d, J = 22.3 Hz), 98.67 (d, J = 28.3 Hz), 56.28, 27.15. **^{19}F NMR** (470 MHz, DMSO-d_6) δ -104.18, -106.76 (td, J = 9.8, 6.4 Hz). **HRMS-**ESI (*m/z*): calculated for $[M+H]^+$ $C_{19}H_{14}FN_3O_5S_2$, 448.0431; found: 448.0433.

***N*-(5-((1*H*-indol-2-yl)methylene)-4-oxo-4,5-dihydrothiazol-2-yl)-4-methoxybenzenesulfonamide (18)**

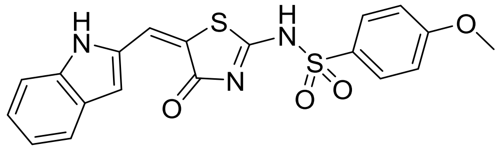

Following the general procedure C, the product was obtained as a yellow solid after filtration and washing using water and diethyl ether (22.7 mg, 0.06 mmol, 32%).

^{1}H NMR (700 MHz, DMSO-d_6) δ 12.46 (s, 1H), 11.97 (s, 1H), 9.86 (s, 1H), 7.81 – 7.78 (m, 2H), 7.75 (dd, J = 8.2, 1.1 Hz, 1H), 7.46 (dq, J = 8.2, 1.0 Hz, 1H), 7.40 (dd, J = 2.2, 1.0 Hz, 1H), 7.34 (ddd, J = 8.2, 6.9, 1.1 Hz, 1H), 7.16 – 7.12 (m, 2H), 4.04 (s, 2H), 3.85 (s, 3H). **^{13}C NMR** (176 MHz, DMSO-d_6) δ 183.47, 174.21, 172.47, 163.24, 138.87, 136.81, 132.69, 129.16, 127.23, 126.82, 123.51, 120.95, 114.93, 114.62, 113.39, 56.22, 35.36. **HRMS-**ESI (*m/z*): calculated for $[M+H]^+$ $C_{19}H_{15}N_3O_4S_2$, 414.0576; found: 414.0576.

***N*-(5-(5-chloro-2-oxoindolin-3-ylidene)-4-oxo-4,5-dihydrothiazol-2-yl)-4-methylbenzenesulfonamide (19)**

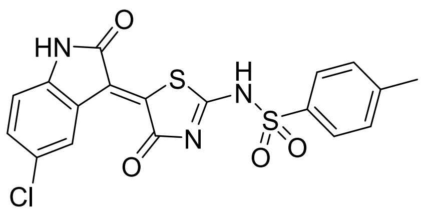

Following the general procedure C, the product was obtained as a red solid after filtration and washing using water, ethanol and diethyl ether (172.0 mg, 0.4 mmol, 48%).

^{1}H NMR (600 MHz, DMSO-d_6) δ 13.36 (s, 1H), 11.40 (s, 1H), 8.79 (d, J = 2.2 Hz, 1H), 7.88 – 7.72 (m, 2H), 7.50 – 7.39 (m, 3H), 6.96 (d, J = 8.4 Hz, 1H), 2.41 (s, 3H). **^{13}C NMR** (151 MHz, DMSO-d_6) δ 168.72, 168.31, 167.47, 144.50, 143.19, 137.81, 133.41, 132.50, 130.36, 127.92, 127.05, 126.70, 126.32, 121.70, 112.40, 21.54. **HRMS-**ESI (*m/z*): calculated for $[M+H]^+$ $C_{18}H_{12}ClN_3O_4S_2$, 434.0030; found: 434.0030.

***N*-(5-(5-chloro-2-oxoindolin-3-ylidene)-4-oxo-4,5-dihydrothiazol-2-yl)benzenesulfonamide (20)**

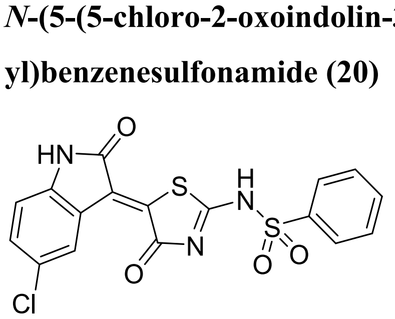

Following the general procedure C, the product was obtained as a red solid after filtration and washing using water, ethanol and diethyl ether (166.5 mg, 0.3 mmol, 64%).

^{1}**H NMR** (700 MHz, DMSO-d_6) δ 13.40 (s, 1H), 11.41 (s, 1H), 8.78 (d, *J* = 2.2 Hz, 1H), 8.00 – 7.86 (m, 2H), 7.77 – 7.70 (m, 1H), 7.69 – 7.63 (m, 2H), 7.44 (dd, *J* = 8.4, 2.2 Hz, 1H), 6.96 (d, *J* = 8.4 Hz, 1H). ^{13}**C NMR** (176 MHz, DMSO-d_6) δ 168.74, 168.71, 167.49, 143.20, 140.65, 133.98, 133.39, 132.52, 129.97, 127.92, 126.98, 126.77, 126.33, 121.68, 112.41. **HRMS-**ESI (*m/z*): calculated for $[M+H]^+$ $C_{17}H_{10}ClN_3O_4S_2$, 419.9874; found: 419.9875.

***N*-(5-(5-chloro-2-oxoindolin-3-ylidene)-4-oxo-4,5-dihydrothiazol-2-yl)-3,5-difluorobenzenesulfonamide (21)**

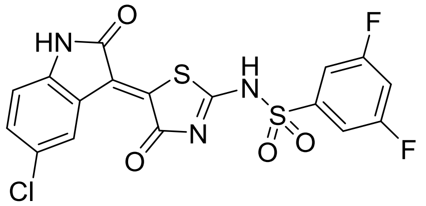

Following the general procedure C, the product was obtained as a red solid after filtration and washing using water, ethanol and diethyl ether (60.4 mg, 0.13 mmol, 78%).

^{1}**H NMR** (600 MHz, DMSO-d_6) δ 13.61 (s, 1H), 11.42 (s, 1H), 8.79 (d, *J* = 2.2 Hz, 1H), 7.71 (tt, *J* = 9.2, 2.4 Hz, 1H), 7.67 – 7.59 (m, 2H), 7.46 (dd, *J* = 8.4, 2.2 Hz, 1H), 6.97 (d, *J* = 8.4 Hz, 1H).^{13}**C NMR** (151 MHz, DMSO-d_6) δ 172.48, 170.96, 170.95, 168.70, 167.93, 167.92, 163.53 (d, *J* = 12.2 Hz), 161.86 (d, *J* = 12.2 Hz), 144.00 (t, *J* = 8.6 Hz), 143.27, 133.90 – 132.63 (m), 132.65, 127.90, 126.94, 126.38, 121.64, 112.47, 111.04 – 110.60 (m), 109.70 (t, *J* = 25.7 Hz), 40.52. ^{19}**F NMR** (470 MHz, DMSO-d_6) δ -105.88 (t, *J* = 6.9 Hz). **HRMS-**ESI (*m/z*): calculated for $[M+H]^+$ $C_{17}H_8ClF_2N_3O_4S_2$, 455.9691; found: 455.9687.

***N*-(5-(5-chloro-2-oxoindolin-3-ylidene)-4-oxo-4,5-dihydrothiazol-2-yl)-3-nitrobenzenesulfonamide (22)**

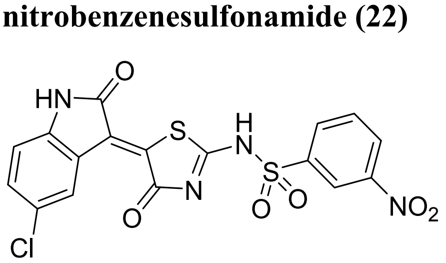

Following the general procedure C, the product was obtained as an orange solid after filtration and washing using water, ethanol and diethyl ether (60.2 mg, 0.13 mmol, 76%).

^{1}**H NMR** (600 MHz, DMSO-d_6) δ 12.69 (s, 1H), 11.42 (s, 1H), 8.80 (d, *J* = 2.2 Hz, 2H), 8.58 – 8.50 (m, 1H), 8.35 (d, *J* = 8.0 Hz, 1H), 7.96 (t, *J* = 8.0 Hz, 1H), 7.47 (dd, *J* = 8.4, 2.2 Hz,

1H), 6.98 (d, J = 8.4 Hz, 1H). **^{13}C NMR** (151 MHz, DMSO-d_6) δ 172.48, 171.02, 168.74, 168.29 148.36, 143.24, 142.34, 133.44, 133.02, 132.62, 132.10, 128.42, 127.90, 126.86, 126.36, 121.69, 112.47. **HRMS-**ESI (*m/z*): calculated for $[M+H]^+$ $C_{17}H_9ClN_4O_6S_2$, 464.9652; found: 464.9628.

4-(*tert*-butyl)-*N*-(5-(5-chloro-2-oxoindolin-3-ylidene)-4-oxo-4,5-dihydrothiazol-2-yl)benzenesulfonamide (23)

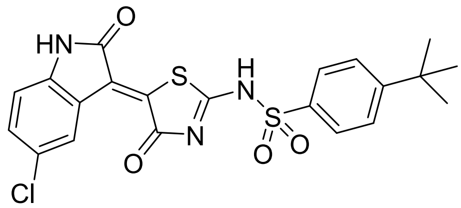

Following the general procedure C, the product was obtained as a red solid after filtration and washing using water, ethanol and diethyl ether (60.0 mg, 0.13 mmol, 79%).

^{1}H NMR (600 MHz, DMSO-d_6) δ 13.35 (s, 1H), 11.41 (s, 1H), 8.79 (d, J = 2.2 Hz, 1H), 7.88 – 7.80 (m, 2H), 7.70 – 7.65 (m, 2H), 7.45 (dd, J = 8.4, 2.2 Hz, 1H), 6.96 (d, J = 8.4 Hz, 1H), 1.31 (s, 9H). **^{13}C NMR** (151 MHz, DMSO-d_6) δ 168.72, 168.33, 167.48, 157.12, 143.19, 137.80, 133.43, 132.50, 127.92, 126.94, 126.82, 126.70, 126.32, 121.71, 112.41, 35.46, 31.21. **HRMS-**ESI (*m/z*): calculated for $[M+H]^+$ $C_{21}H_{18}ClN_3O_4S_2$, 476.0500; found: 476.0503.

2-chloro-*N*-(5-(5-chloro-2-oxoindolin-3-ylidene)-4-oxo-4,5-dihydrothiazol-2-yl)-4-fluorobenzenesulfonamide (24)

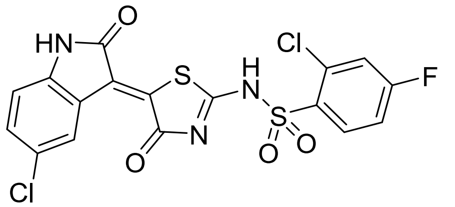

Following the general procedure C, the product was obtained as a red solid after filtration and washing using water, ethanol and diethyl ether (61.5 mg, 0.13 mmol, 81%).

^{1}H NMR (600 MHz, DMSO-d_6) δ 13.51 (s, 1H), 11.41 (s, 1H), 8.79 (d, J = 2.2 Hz, 1H), 8.20 (dd, J = 8.9, 5.9 Hz, 1H), 7.79 (dd, J = 8.6, 2.6 Hz, 1H), 7.51 (ddd, J = 8.9, 8.0, 2.6 Hz, 1H), 7.46 (dd, J = 8.4, 2.2 Hz, 1H), 6.97 (d, J = 8.4 Hz, 1H).**^{13}C NMR** (151 MHz, DMSO-d_6) δ 170.35, 168.71, 167.65, 165.62, 163.92, 143.28, 134.70 (d, J = 3.3 Hz), 133.97 (d, J = 11.6 Hz), 133.18, 133.18, 132.73, 132.66, 132.62, 127.94, 127.02, 126.35, 121.69, 120.06 (d, J =

26.2 Hz), 115.61 (d, J = 22.0 Hz), 112.45. **^{19}F NMR** (470 MHz, DMSO-d_6) δ -103.44. **HRMS-**ESI (m/z): calculated for $[M+H]^+$ $C_{17}H_8Cl_2FN_3O_4S_2$, 471.9390; found: 471.9395.

***N*-(5-(5-chloro-2-oxoindolin-3-ylidene)-4-oxo-4,5-dihydrothiazol-2-yl)-4-cyclohexylbenzenesulfonamide (25)**

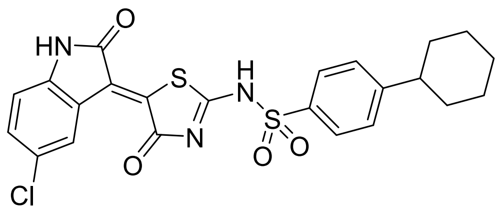

Following the general procedure C, the product was obtained as a red solid after filtration and washing using water, ethanol and diethyl ether (60.1 mg, 0.12 mmol, 80%).

^{1}H NMR (600 MHz, DMSO-d_6) δ 13.35 (s, 1H), 11.40 (s, 1H), 8.78 (d, J = 2.2 Hz, 1H), 7.82 (d, J = 8.4 Hz, 2H), 7.50 (d, J = 8.4 Hz, 2H), 7.44 (dd, J = 8.4, 2.2 Hz, 1H), 6.96 (d, J = 8.4 Hz, 1H), 2.62 (d, J = 29.3 Hz, 1H), 1.81-1.77 (m, 4H), 1.74 – 1.66 (m, 1H), 1.48 – 1.30 (m, 4H), 1.27-1.20 (m, 1H). **^{13}C NMR** (176 MHz, DMSO-d_6) δ 168.72, 168.23, 167.41, 154.00, 143.18, 138.09, 133.43, 132.49, 128.23, 127.91, 127.17, 126.69, 126.32, 121.70, 112.40, 44.14, 33.95, 26.59, 25.88. **LC-MS** (m/z): calculated for $[M+H]^+$ $C_{23}H_{20}ClN_3O_4S_2$, 502.0; found: 502.0.

***N*-(5-(5-chloro-2-oxoindolin-3-ylidene)-4-oxo-4,5-dihydrothiazol-2-yl)-[1,1'-biphenyl]-4-sulfonamide (26)**

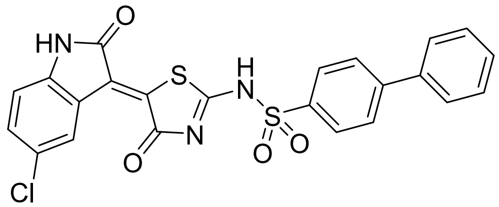

Following the general procedure C, the product was obtained as a red solid after filtration and washing using water, ethanol and diethyl ether (61.1 mg, 0.12 mmol, 82%).

^{1}H NMR (600 MHz, DMSO-d_6) δ 13.41 (s, 1H), 11.41 (s, 1H), 8.79 (d, J = 2.2 Hz, 1H), 8.02 – 7.96 (m, 2H), 7.96 – 7.92 (m, 2H), 7.77 – 7.72 (m, 2H), 7.52 (dd, J = 8.4, 7.0 Hz, 2H), 7.48 – 7.43 (m, 2H), 6.97 (d, J = 8.4 Hz, 1H). **^{13}C NMR** (151 MHz, DMSO-d_6) δ 168.73, 168.60, 167.51, 145.45, 143.21, 143.03, 139.34, 138.83, 133.36, 132.54, 129.65, 129.18, 128.15, 127.93, 127.70, 127.64, 126.78, 126.34, 121.70, 121.66, 112.43. **HRMS-**ESI (m/z): calculated for $[M+H]^+$ $C_{23}H_{14}ClN_3O_4S_2$, 496.0187; found: 496.0191.

4-(*N*-(5-(5-chloro-2-oxoindolin-3-ylidene)-4-oxo-4,5-dihydrothiazol-2-yl)sulfamoyl)benzoic acid (27)

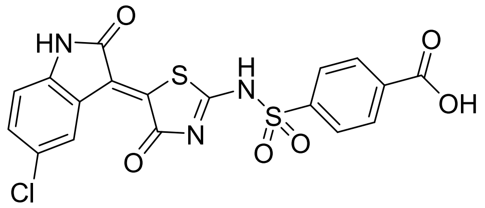

Following the general procedure C, the product was obtained as a red-brown solid after filtration and washing using water, ethanol and diethyl ether (d.r. = 1:1) 47.0 mg, 0.1 mmol, 78%).

^{1}H NMR (600 MHz, DMSO-d_6) δ 13.52 (s, 1H), 11.41 (s, 1H), 8.78 (d, J = 2.0 Hz, 1H), 8.35 (d, J = 2.0 Hz, 1H), 8.18 – 8.15 (m, 1H), 8.06 – 8.02 (m, 2H), 7.44 (dd, J = 4.4, 2.0 Hz, 1H), 6.96 (d, J = 8.4 Hz, 1H), 4.08 (s, 1H). **^{13}C NMR** (151 MHz, DMSO-d_6) δ 174.26, 168.70, 166.54, 144.75, 144.20, 143.31, 135.42, 132.63, 130.79, 127.92, 127.33, 126.88, 126.36, 121.66, 112.44, 35.66. **HRMS-**ESI (*m/z*): calculated for $[M+H]^+$ $C_{18}H_{10}ClN_3O_6S_2$, 463.9772; found: 463.9775.

5-bromo-*N*-(5-(5-chloro-2-oxoindolin-3-ylidene)-4-oxo-4,5-dihydrothiazol-2-yl)-2-methoxybenzenesulfonamide (28)

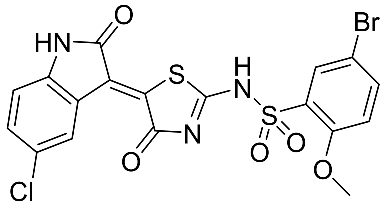

Following the general procedure C, the product was obtained as a red-brown solid after filtration and washing using water, ethanol and diethyl ether (58.5 mg, 0.11 mmol, 79%).

^{1}H NMR (600 MHz, DMSO-d_6) δ 13.42 (s, 1H), 11.40 (s, 1H), 8.82 (d, J = 2.2 Hz, 1H), 7.93 (d, J = 2.6 Hz, 1H), 7.88 (dd, J = 8.9, 2.6 Hz, 1H), 7.46 (dd, J = 8.4, 2.2 Hz, 1H), 7.26 (d, J = 8.9 Hz, 1H), 6.98 (d, J = 8.4 Hz, 1H), 3.85 (s, 3H). **^{13}C NMR** (151 MHz, DMSO-d_6) δ 169.88, 168.75, 167.48, 156.71, 143.23, 138.37, 133.47, 132.51, 130.92, 129.69, 127.92, 126.83, 126.30, 121.77, 116.33, 112.40, 111.62, 57.30. **HRMS-**ESI (*m/z*): calculated for $[M+H]^+$ $C_{18}H_{11}{}^{79}BrClN_3O_5S_2$, 527.9084; found: 527.9091. Calculated for $[M+H]^+$ $C_{18}H_{11}{}^{81}BrClN_3O_5S_2$, 529.9064; found: 529.9066.

***N*-(5-(5-chloro-2-oxoindolin-3-ylidene)-4-oxo-4,5-dihydrothiazol-2-yl)methanesulfonamide (29)**

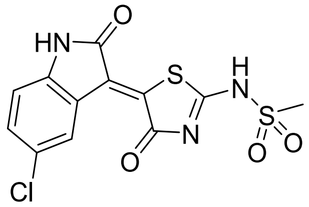

Following the general procedure C, the product was obtained as a brown solid after filtration and washing using water, ethanol and diethyl ether (63.6 mg, 0.18 mmol, 74%).

^{1}H NMR (700 MHz, DMSO-d_6) δ 13.35 (s, 1H), 11.38 (s, 1H), 8.80 (d, *J* = 2.3 Hz, 1H), 7.45 (dd, *J* = 8.3, 2.3 Hz, 1H), 6.96 (d, *J* = 8.3 Hz, 1H), 3.19 (s, 3H). **^{13}C NMR** (151 MHz, DMSO-d_6) δ 168.69, 167.55, 143.14, 133.52, 132.44, 127.90, 127.70, 126.51, 126.32, 121.69, 112.39, 42.12. **HRMS-**ESI (*m/z*): calculated for $[M+H]^+$ $C_{12}H_8ClN_3O_4S_2$, 357.9717; found: 357.9717.

***N*-(5-(5-chloro-2-oxoindolin-3-ylidene)-4-oxo-4,5-dihydrothiazol-2-yl)-4-(trifluoromethyl)benzenesulfonamide (30)**

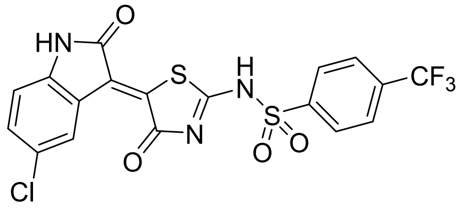

Following the general procedure C, the product was obtained as a red-brown solid after filtration and washing using water, ethanol and diethyl ether (64.1 mg, 0.13 mmol, 85%).

^{1}H NMR (700 MHz, DMSO-d_6) δ 13.51 (s, 1H), 11.42 (d, *J* = 5.1 Hz, 1H), 8.77 (d, *J* = 4.8 Hz, 1H), 8.13 (t, *J* = 6.4 Hz, 2H), 8.04 (dd, *J* = 8.4, 4.8 Hz, 2H), 7.45 (t, *J* = 7.1 Hz, 1H), 6.96 (dd, *J* = 8.4, 4.7 Hz, 1H). **^{13}C NMR** (176 MHz, DMSO-d_6) δ 170.32, 168.68, 167.69, 144.51, 143.26, 133.43 (q, *J* = 34.5, 33.6 Hz), 132.64, 128.05, 127.91, 127.20, 127.18, 126.96, 126.37, 124.63, 123.08, 121.63, 112.46, 55.79. **^{19}F NMR** (470 MHz, DMSO-d_6) δ -61.71. **HRMS-**ESI (*m/z*): calculated for $[M+H]^+$ $C_{18}H_9ClF_3N_3O_4S_2$, 487.9747; found: 487.9752.

***N*-(5-(5-chloro-2-oxoindolin-3-ylidene)-4-oxo-4,5-dihydrothiazol-2-yl)naphthalene-2-sulfonamide (31)**

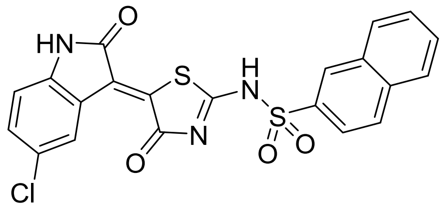

Following the general procedure C, the product was obtained as a red solid after filtration and washing using water, ethanol and diethyl ether (65.6 mg, 0.14 mmol, 88%).

^{1}H NMR (600 MHz, DMSO-d_6) δ 13.41 (s, 1H), 11.41 (s, 1H), 8.78 (d, *J* = 2.2 Hz, 1H), 8.62 (d, *J* = 1.9 Hz, 1H), 8.24 (dd, *J* = 8.4, 1.3 Hz, 1H), 8.18 (d, *J* = 8.7 Hz, 1H), 8.11 – 8.06 (m, 1H), 7.90 (dd, *J* = 8.7, 1.9 Hz, 1H), 7.74 (ddd, *J* = 8.2, 6.8, 1.3 Hz, 1H), 7.70 (ddd, *J* = 8.2, 6.8, 1.3 Hz, 1H), 7.44 (dd, *J* = 8.4, 2.2 Hz, 1H), 6.96 (d, *J* = 8.4 Hz, 1H). **^{13}C NMR** (151 MHz, DMSO-d_6) δ 168.77, 168.72, 167.51, 143.20, 137.66, 135.00, 133.39, 132.52, 132.13, 130.09, 129.94, 129.73, 128.37, 128.28, 127.97, 127.91, 126.77, 126.33, 122.54, 121.69, 112.41. **HRMS-**ESI (*m/z*): calculated for $[M+H]^+$ $C_{21}H_{12}ClN_3O_4S_2$, 470.0030; found: 470.0033.

***N*-(5-(5-chloro-2-oxoindolin-3-ylidene)-4-oxo-4,5-dihydrothiazol-2-yl)-4-cyanobenzenesulfonamide (32)**

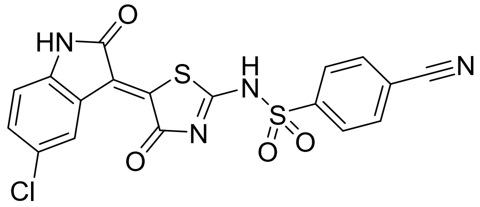

Following the general procedure C, the product was obtained as a bright red solid after filtration and washing using water, ethanol and diethyl ether (49.1 mg, 0.11 mmol, 85%).

^{1}H NMR (600 MHz, DMSO-d_6) δ 12.69 (s, 1H), 11.41 (s, 1H), 8.89 – 8.73 (m, 1H), 8.17 – 8.04 (m, 4H), 7.47 (dd, *J* = 8.4, 2.3 Hz, 1H), 6.98 (d, *J* = 8.4 Hz, 1H). **^{13}C NMR** (151 MHz, DMSO-d_6) δ 168.76, 144.82, 143.20, 134.08, 134.03, 132.55, 127.90, 127.76, 127.63, 126.73, 126.34, 121.72, 118.11, 116.10, 112.44, 21.53. **LC-MS** (*m/z*): calculated for $[M+H]^+$ $C_{18}H_9ClN_4O_4S_2$, 444.9; found: 444.8.

***N*-(5-(5-chloro-2-oxoindolin-3-ylidene)-4-oxo-4,5-dihydrothiazol-2-yl)-4-fluorobenzenesulfonamide (33)**

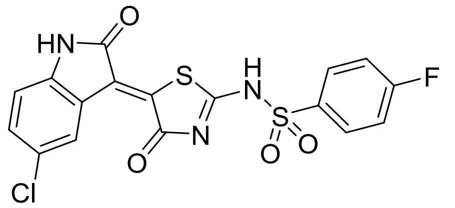

Following the general procedure C, the product was obtained as a red solid after filtration and washing using water, ethanol and diethyl ether (63.9 mg, 0.15 mmol, 81%).

^{1}H NMR (700 MHz, DMSO-d_6) δ 13.41 (s, 1H), 11.41 (s, 1H), 8.80 (s, 1H), 7.98 (d, J = 7.0 Hz, 2H), 7.66 – 7.27 (m, 3H), 6.98 (d, J = 8.3 Hz, 1H).**^{13}C NMR** (176 MHz, DMSO-d_6) δ 168.75, 165.85, 164.42, 143.18, 137.16, 132.52, 130.17 (d, J = 9.7 Hz), 127.91, 126.68, 126.32, 121.72, 117.15 (d, J = 22.5 Hz), 112.43, 55.84. **^{19}F NMR** (470 MHz, DMSO-d_6) δ -105.25. **LC-MS** (*m/z*): calculated for $[M+H]^+$ $C_{17}H_9ClFN_3O_4S_2$, 437.9; found: 437.8.

***N*-(5-(5-chloro-2-oxoindolin-3-ylidene)-4-oxo-4,5-dihydrothiazol-2-yl)-4-methylbenzamide (34)**

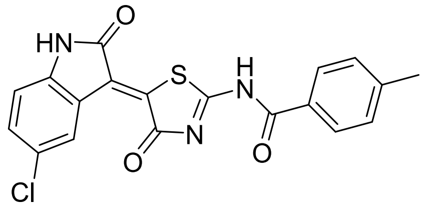

Following the general procedure C, the product was obtained as a red solid after filtration and washing using water, ethanol and diethyl ether (94.7 mg, 0.24 mmol, 79%).

^{1}H NMR (600 MHz, DMSO-d_6) δ 13.31 (s, 1H), 11.30 (s, 1H), 8.91 (s, 1H), 8.07 (d, J = 7.3 Hz, 2H), 7.44 (dd, J = 8.3, 2.2 Hz, 1H), 7.37 (d, J = 7.3 Hz, 2H), 6.95 (d, J = 8.3 Hz, 1H), 2.40 (s, 3H). **^{13}C NMR** (151 MHz, DMSO-d_6) δ 168.89, 144.62, 143.17, 142.02, 136.06, 132.40, 130.04, 129.81, 129.59, 129.02, 128.60, 127.94, 127.71, 126.21, 121.98, 112.22, 21.75. **LC-MS** (*m/z*): calculated for $[M+H]^+$ $C_{19}H_{12}ClN_3O_3S$, 398.0; found: 398.0.

***N*-(5-(5-chloro-2-oxoindolin-3-ylidene)-4-oxo-4,5-dihydrothiazol-2-yl)-4-methoxybenzamide (35)**

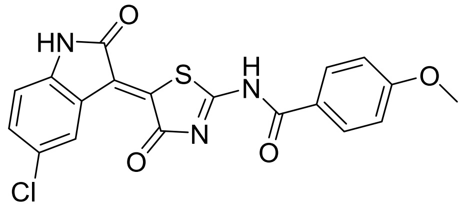

Following the general procedure C, the product was obtained as a red solid after filtration and washing using water, ethanol and diethyl ether (69.7 mg, 0.17 mmol, 84%).

^{1}H NMR (600 MHz, DMSO-*d*$_6$) δ 13.28 (s, 1H), 11.31 (s, 1H), 8.94 (s, 1H), 8.16 (d, *J* = 8.9 Hz, 2H), 7.46 (dd, *J* = 8.4, 2.3 Hz, 1H), 7.11 (d, *J* = 8.9 Hz, 2H), 6.97 (d, *J* = 8.4 Hz, 1H), 3.88 (s, 3H). **^{13}C NMR** (151 MHz, DMSO-*d*$_6$) δ 168.93, 168.76, 167.50, 164.10, 143.14, 143.08, 132.35, 132.26, 127.92, 127.58, 126.19, 126.05, 122.02, 114.58, 112.23, 111.88, 56.10. **LC-MS** (*m/z*): calculated for $[M+H]^+$ $C_{19}H_{12}ClN_3O_4S$, 414.0; found: 414.0.

***N*-(5-(5-chloro-2-oxoindolin-3-ylidene)-4-oxo-4,5-dihydrothiazol-2-yl)-3,4-dimethoxybenzamide (36)**

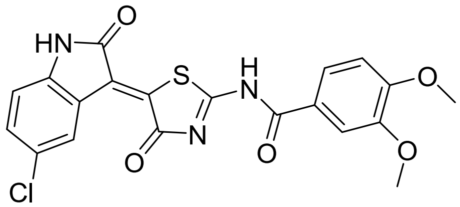

Following the general procedure C, the product was obtained as a red solid after filtration and washing using water, ethanol and diethyl ether (55.4 mg, 0.12 mmol, 69%).

^{1}H NMR (600 MHz, DMSO-*d*$_6$) δ 13.28 (s, 1H), 11.30 (s, 1H), 8.94 (s, 1H), 7.87 (dd, *J* = 8.4, 2.2 Hz, 1H), 7.71 (s, 1H), 7.46 (dd, *J* = 8.4, 2.2 Hz, 1H), 7.15 (d, *J* = 8.6 Hz, 1H), 6.97 (d, *J* = 8.4 Hz, 1H), 3.88 (s, 3H), 3.85 (s, 3H). **^{13}C NMR** (151 MHz, DMSO-*d*$_6$) δ 172.48, 168.94, 153.99, 148.99, 143.15, 132.92, 132.36, 130.12, 127.92, 126.20, 124.57, 123.68, 123.19, 122.03, 112.23, 111.89, 111.60, 56.28, 56.02. **LC-MS** (*m/z*): calculated for $[M+H]^+$ $C_{20}H_{14}ClN_3O_5S$, 444.0; found: 444.8.

***N*-(5-(5-chloro-2-oxoindolin-3-ylidene)-4-oxo-4,5-dihydrothiazol-2-yl)-4-fluorobenzamide (37)**

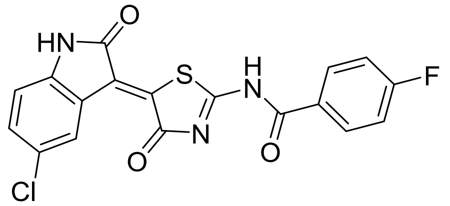

Following the general procedure C, the product was obtained as a red solid after filtration and washing using water, ethanol and diethyl ether (63.9 mg, 0.16 mmol, 76%).

^{1}H NMR (600 MHz, DMSO-*d*$_6$) δ 13.36 (s, 1H), 11.29 (s, 1H), 8.87 (d, *J* = 2.3 Hz, 1H), 8.21 (dd, *J* = 8.7, 5.7 Hz, 2H), 7.44 – 7.33 (m, 3H), 6.92 (d, *J* = 8.3 Hz, 1H). **^{13}C NMR** (151 MHz,

DMSO-d_6) δ 168.82, 166.59, 164.92, 143.20, 143.02, 135.65, 132.86 (d, J = 9.7 Hz), 132.44, 131.46, 127.94, 127.60, 126.22, 121.90, 116.28 (d, J = 22.0 Hz), 112.19. **^{19}F NMR** (470 MHz, DMSO-d_6) δ -105.49. **LC-MS** (*m/z*): calculated for $[M+H]^+$ $C_{18}H_9ClFN_3O_3S$, 402.0; found: 401.6.

***N*-(5-(5-chloro-2-oxoindolin-3-ylidene)-4-oxo-4,5-dihydrooxazol-2-yl)-4-methoxybenzenesulfonamide (38)**

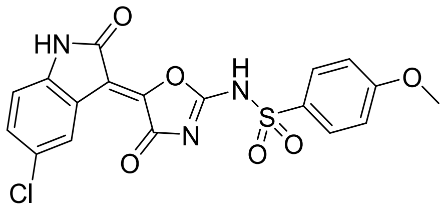

Following the general procedure C, the product was obtained as a beige solid after filtration and washing using water, ethanol and diethyl ether (126.4 mg, 0.2 mmol, 100%).

^{1}H NMR (600 MHz, DMSO-d_6) δ 12.63 (s, 1H), 10.74 (s, 1H), 7.86 (d, J = 8.4 Hz, 2H), 7.32 (dd, J = 8.3, 2.2 Hz, 1H), 7.13 (d, J = 8.4 Hz, 2H), 6.93 (s, 1H), 6.84 (d, J = 8.3 Hz, 1H), 3.85 (s, 3H). **^{13}C NMR** (151 MHz, DMSO-d_6) δ 174.18, 169.94, 162.99, 159.34, 141.81, 133.44, 130.93, 129.68, 128.39, 126.45, 125.23, 114.79, 112.22, 85.33, 75.57, 56.16. **LC-MS** (*m/z*): calculated for $[M+H]^+$ $C_{19}H_{12}BrN_3O_6S_2$, 434.0; found: 434.0.

4-(*N*-(5-(5-bromo-1-methyl-2-oxoindolin-3-ylidene)-4-oxo-4,5-dihydrothiazol-2-yl)sulfamoyl)benzoic acid (45)

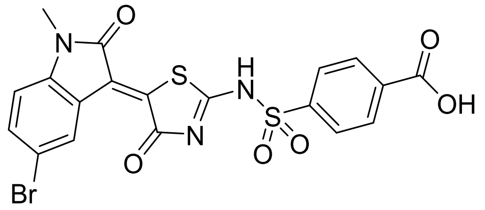

Following the general procedure C, the product was obtained as a diastereomeric mixture as a red-brown solid after filtration and washing using water, ethanol and diethyl ether (74.9 mg, 0.14 mmol, 84%).

^{1}H NMR (600 MHz, DMSO-d_6) δ 13.49 (s, 1H), 12.65 (s, 1H), 8.92 (d, J = 2.2 Hz, 1H), 8.21 – 8.14 (m, 2H), 8.05 – 8.02 (m, 2H), 7.66 (dd, J = 8.5, 2.2 Hz, 1H), 7.11 (d, J = 8.5 Hz, 1H), 3.24 (s, 3H). **^{13}C NMR** (151 MHz, DMSO-d_6) δ 174.26, 172.47, 166.99, 166.54, 144.51, 144.18, 135.43, 135.18, 130.79, 130.49, 130.41, 127.37, 125.71, 121.35, 114.73, 111.80, 27.01.

HRMS-ESI (*m/z*): calculated for $[M+H]^+$ $C_{19}H_{12}{}^{79}BrN_3O_6S_2$, 521.9423; found: 521.9428. Calculated for $[M+H]^+$ $C_{19}H_{12}{}^{81}BrN_3O_6S_2$, 523.9407; found: 523.9407.

Formation of the potassium salt of 45

A 25 mL round bottom flask was charged with **45** (50.0 mg, 0.1 mmol, 1.0 equiv.), and methanol (1.5 mL). After stirring for 5 min, potassium carbonate (13.2 mg, 0.1 mmol, 1.0 equiv.) was slowly added to the solution. The reaction mixture was stirred for 1 h at room temperature. The solvent was then removed under reduced pressure and the resulting solid was dried under vacuum to give the corresponding potassium salt of **45** as an orange solid (quantitative yield).

4-(*N*-(5-(1-methyl-5-nitro-2-oxoindolin-3-ylidene)-4-oxo-4,5-dihydrothiazol-2-yl)sulfamoyl)benzoic acid (46)

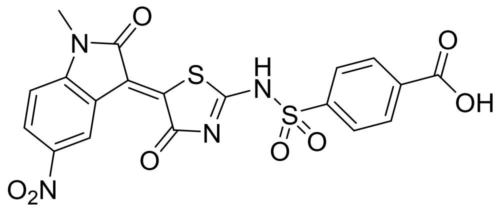

Following the general procedure C, the product was obtained as diastereomeric mixture visible as an orange-brown solid after filtration and washing using water, ethanol and diethyl ether (62.1 mg, 0.13 mmol, 75%).

^{1}H NMR (700 MHz, DMSO-d_6) δ 13.54 (s, 1H), 9.66 (d, J = 2.4 Hz, 1H), 8.42 – 8.28 (m, 2H), 8.17 (d, J = 8.1 Hz, 2H), 8.04 (d, J = 8.1 Hz, 2H), 7.36 (d, J = 8.9 Hz, 1H), 3.33 (s, 3H). **^{13}C NMR** (176 MHz, DMSO-d_6) δ 172.48, 169.33, 168.54, 167.92, 166.54, 150.18, 144.09, 143.05, 142.71, 135.47, 130.79, 128.72, 127.40, 123.34, 119.67, 110.14, 27.41. **LC-MS** (*m/z*): calculated for $[M+H]^+$ $C_{19}H_{12}BrN_3O_6S_2$, 489.0; found: 488.8.

4-(*N*-(5-(5-bromo-2-oxoindolin-3-ylidene)-4-oxo-4,5-dihydrothiazol-2-yl)sulfamoyl)benzoic acid (47)

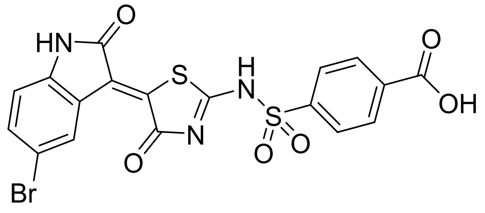

Following the general procedure C, the product was obtained as a red-brown solid after filtration and washing using water, ethanol and diethyl ether (117.1 mg, 0.23 mmol, 69%).

^{1}H NMR (600 MHz, DMSO-d_6) δ 13.51 (s, 1H), 11.41-11.33 (m, 1H), 9.01-8.91 (m, 1H), 8.39 – 8.31 (m, 1H), 8.21 – 8.13 (m, 1H), 8.08 – 7.99 (m, 2H), 7.57 (dd, *J* = 8.3, 2.1 Hz, 1H), 6.91 (dd, *J* = 8.3, 5.1 Hz, 1H), 4.07 (s, 1H). ^{13}C NMR (151 MHz, DMSO) δ 173.88, 173.79, 168.25, 168.13, 166.07, 144.29, 143.75, 143.18, 143.10, 134.94, 130.30, 126.86, 121.90, 121.69, 113.58, 112.44, 39.94, 39.80, 39.66, 39.52, 39.38, 39.24, 39.10. **LC-MS** (*m/z*): calculated for [M+H]$^+$ $C_{18}H_{10}{}^{79}BrN_3O_6S_2$, 507.9; found: 507.8. Calculated for [M+H]$^+$ $C_{18}H_{10}{}^{81}BrN_3O_6S_2$, 509.9; found: 509.8.

methyl-4-(*N*-(5-(5-bromo-1-methyl-2-oxoindolin-3-ylidene)-4-oxo-4,5-dihydrothiazol-2-yl)sulfamoyl)benzoate (48)

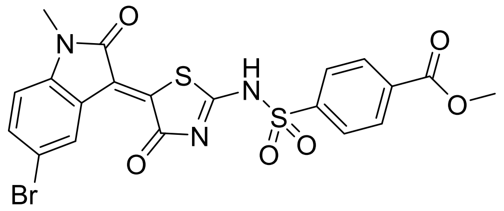

Following the general procedure C, the product was obtained as a red-brown solid after filtration and washing using water, ethanol and diethyl ether (75.4 mg, 0.14 mmol, 88%).
^{1}H NMR (500 MHz, DMSO-d_6) δ 13.54 (s, 1H), 8.94 (d, *J* = 2.7 Hz, 1H), 8.19 (d, *J* = 8.1 Hz, 2H), 8.06 (d, *J* = 8.1 Hz, 2H), 7.74 – 7.60 (m, 1H), 7.13 (d, *J* = 8.4 Hz, 1H), 3.90 (s, 3H), 3.25 (s, 3H). **^{13}C NMR** (126 MHz, DMSO-d_6) δ 171.94, 167.02, 165.53, 156.68, 144.54, 135.19, 134.79, 134.12, 133.84, 130.70, 130.41, 127.51, 125.72, 121.37, 114.72, 111.83, 53.19, 27.02. **HRMS-**ESI (*m/z*): calculated for [M+H]$^+$ $C_{20}H_{14}{}^{81}BrN_3O_6S_2$, 537.9586; found: 537.9563.

3-(*N*-(5-(5-bromo-1-methyl-2-oxoindolin-3-ylidene)-4-oxo-4,5-dihydrothiazol-2-yl)sulfamoyl)benzoic acid (49)

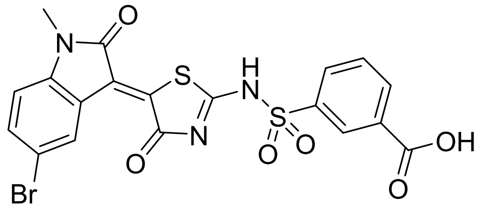

Following the general procedure C, the product was obtained as a red-brown solid after filtration and washing using water, ethanol and diethyl ether ((d.r. = 3:1) 34.8 mg, 0.07 mmol, 74%).
^{1}H NMR (700 MHz, DMSO-d_6) δ 13.57 (s, 1H), 9.44 (s, 1H), 9.19 (s, 1H), 8.95 (s, 1H), 8.39 (s, 1H), 8.24 (d, *J* = 7.8 Hz, 1H), 7.80 (t, *J* = 7.8 Hz, 1H), 7.68 (d, *J* = 8.5 Hz, 1H), 7.13 (d, *J* =

8.5 Hz, 1H), 3.25 (s, 3H). **^{13}C NMR** (176 MHz, DMSO-d_6) δ 180.45, 178.77, 167.64, 167.03, 144.51, 143.45, 141.73, 135.14, 134.35, 133.72, 130.73, 130.21, 127.47, 122.84, 121.38, 114.70, 111.80, 111.24, 27.02. **HRMS-**ESI (*m/z*): calculated for $[M+H]^+$ $C_{19}H_{12}{}^{79}BrN_3O_6S_2$, 521.9423; found: 521.9429. Calculated for $[M+H]^+$ $C_{19}H_{12}{}^{81}BrN_3O_6S_2$, 523.9403; found: 523.9407.

benzoyl isothiocyanate (IV)

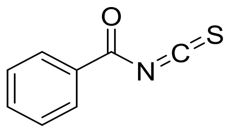

Benzoyl chloride (500.0 µL, 4.3 mmol, 1 equiv.) was added to a freshly prepared solution of NH_4SCN (327.9 mg, 4.3 mmol, 1 equiv.) in acetone (7 mL), and the mixture was stirred for 30 minutes at room temperature: The *in situ*-generated benzoyl isothiocyanate was obtained after removing the solvent under reduced pressure. The product was used in the next step without further purification (703.3 mg, 4.3 mmol, 100%).

***N*-((4-hydroxyphenyl)carbamothioyl)benzamide (V)**

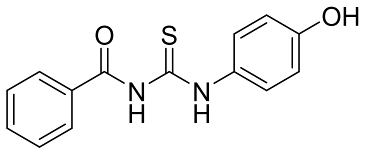

The benzoyl isothiocyanate (703.4 mg, 4.3 mmol, 1 equiv.) was resuspended in ethyl acetate (10 mL) and 4-aminophenol (470.3 mg, 4.3 mmol, 1 equiv.) was added. The mixture was stirred overnight and then poured in ice water with vigorous stirring. The resulting white solid was collected by vacuum filtration and dried to obtain the desired thiourea (688.76 mg, 4.09 mmol, 95%). Compound characterization correlates with previously reported characterization.[1]

1-(4-hydroxyphenyl)thiourea (VI)

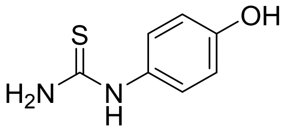

N-((4-hydroxyphenyl)carbamothioyl)benzamide (GG-399) from the previous step was poured into a mixture of aqueous sodium hydroxide (4 M) and ethanol (1:1) and then heated to reflux

for 1.5 h. The precipitating beige solid was collected *via* vacuum filtration and then washed with H_2O to yield the desired product (688.8 mg, 4.1 mmol, 95%).

^{1}H NMR (600 MHz, DMSO-d_6) δ 12.95 (s, 1H), 9.36 (s, 1H), 7.96 (dd, J = 8.3, 1.4 Hz, 2H), 7.65 – 7.60 (m, 1H), 7.51 (t, J = 7.8 Hz, 2H), 6.77 – 6.69 (m, 1H). **^{13}C NMR** (151 MHz, DMSO-d_6) δ 167.78, 133.35, 131.21, 129.73, 129.04.

General Procedure D (One-pot condensation)

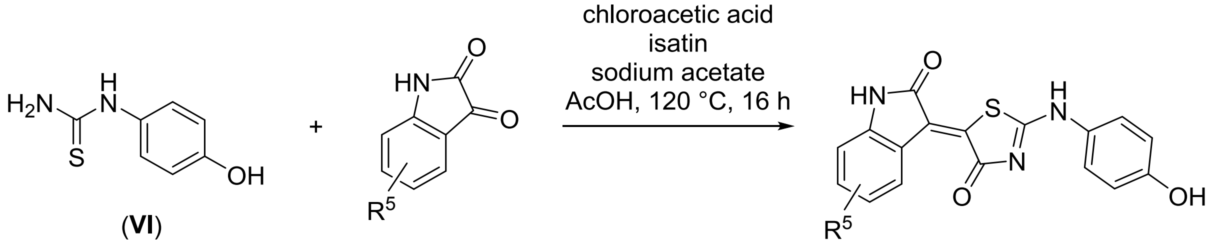

A solution of 1-(4-hydroxyphenyl)thiourea (**V**) (1 equiv.), chloroacetic acid (1 equiv.), isatin (1 equiv.) and sodium acetate (1 equiv.) in acetic acid (0.12 M) was stirred under reflux for 16 h. After cooling to room temperature, the red brownish solid was filtered off, washed with water, ethanol and diethyl ether in sequence to afford the desired 4-thiazolidinones.

5-(5-chloro-2-oxoindolin-3-ylidene)-2-((4-hydroxyphenyl)amino)thiazol-4(5*H*)-one (39)

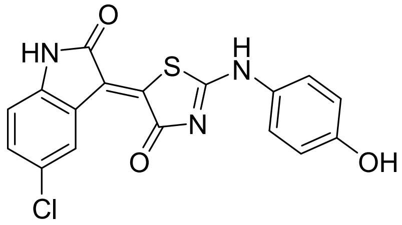

Following the general procedure D, the product was obtained as a red-brown solid after filtration and washing using water, ethanol and diethyl ether (24.4 mg, 0.07 mmol, 20%).

^{1}H NMR (700 MHz, DMSO-d_6) δ 11.60 (s, 1H), 11.23 (d, J = 7.6 Hz, 1H), 9.64 (s, 1H), 9.02 (d, J = 2.2 Hz, 1H), 7.71 – 7.53 (m, 2H), 7.39 (ddd, J = 12.0, 8.3, 2.2 Hz, 1H), 6.96 (d, J = 8.3 Hz, 1H), 6.87 – 6.81 (m, 2H). **^{13}C NMR** (176 MHz, DMSO-d_6) δ 180.72, 172.84, 169.22, 155.78, 142.07, 139.23, 131.15, 130.48, 127.54, 126.08, 124.08, 123.03, 122.26, 116.06, 111.95. **HRMS**-ESI (*m/z*): calculated for $[M+H]^+$ $C_{17}H_{10}ClN_3O_3S$, 372.0204; found: 372.0202.

2-((4-hydroxyphenyl)amino)-5-(5-methyl-2-oxoindolin-3-ylidene)thiazol-4(5*H*)-one (40)

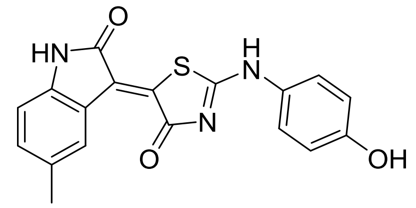

Following the general procedure D, the product was obtained as a red-brown solid after filtration and washing using water, ethanol and diethyl ether (d.r. = 8:1) 34.8 mg, 0.1 mmol, 27%).

^{1}H NMR (700 MHz, DMSO-d_6) δ 11.48 (s, 1H), 10.99 (d, J = 6.9 Hz, 1H), 9.61 (s, 1H), 8.85 – 8.72 (m, 1H), 7.66 – 7.56 (m, 1H), 7.18 – 6.98 (m, 2H), 6.87 – 6.81 (m, 3H), 2.32 (s, 3H). **^{13}C NMR** (176 MHz, DMSO-d_6) δ 180.72, 173.19, 169.50, 155.62, 141.18, 136.90, 132.33, 130.87, 129.79, 128.70, 125.55, 122.94, 121.06, 116.02, 110.27, 21.53. **HRMS-**ESI (*m/z*): calculated for $[M+H]^+$ $C_{17}H_{13}N_4O_5S$, 352.0750; found: 352.0747.

2-((4-hydroxyphenyl)amino)-5-(5-methoxy-2-oxoindolin-3-ylidene)thiazol-4(5*H*)-one (41)

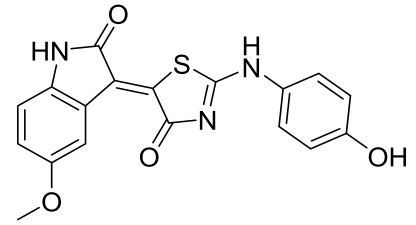

Following the general procedure D, the product was obtained as a brown solid after filtration and washing using water, ethanol and diethyl ether (d.r. = 0.13:1) (37.7 mg, 0.1 mmol, 28%).

^{1}H NMR (700 MHz, DMSO-d_6) δ 11.51 (s, 1H), 10.90 (s, 1H), 9.61 (s, 1H), 8.70-8.62 (m, 1H), 7.66 – 7.56 (m, 1H), 7.03-6.94 (m, 2H), 6.82 (d, J = 6.3 Hz, 3H), 3.76 (s, 3H). **^{13}C NMR** (176 MHz, DMSO-d_6) δ 180.80, 173.14, 169.43, 155.65, 154.98, 137.19, 130.58, 129.82, 125.81, 122.95, 121.65, 117.29, 116.02, 114.45, 110.82, 55.94. **HRMS-**ESI (*m/z*): calculated for $[M+H]^+$ $C_{18}H_{13}N_3O_4S$, 368.0699; found: 368.0696.

2-((4-hydroxyphenyl)amino)-5-(5-nitro-2-oxoindolin-3-ylidene)thiazol-4(5*H*)-one (42)

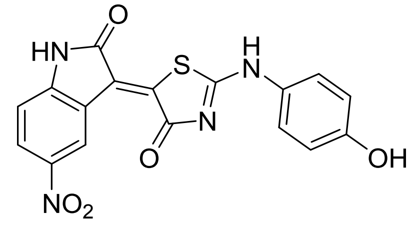

Following the general procedure D, the product was obtained as an orange solid after filtration and washing using water, ethanol and diethyl ether (27.4 mg, 0.07 mmol, 19%).

^{1}H NMR (700 MHz, DMSO-d_6) δ 11.75 (s, 1H), 11.66 (s, 1H), 9.85 (s, 1H), 9.66 (s, 1H), 8.29 – 8.15 (m, 1H), 7.61 (d, J = 8.6 Hz, 1H), 7.10 (s, 1H), 7.03 (s, 1H), 6.84 (d, J = 9.1 Hz, 2H). **^{13}C NMR** (176 MHz, DMSO-d_6) δ 180.56, 172.32, 169.81, 155.88, 148.56, 142.58, 140.95, 130.42, 127.61, 123.50, 123.05, 120.93, 116.43, 116.07, 110.70. **HRMS-**ESI (*m/z*): calculated for $[M+H]^+$ $C_{17}H_{10}N_4O_5S$, 383.0444; found: 383.0444.

5-(5-bromo-2-oxoindolin-3-ylidene)-2-((4-hydroxyphenyl)amino)thiazol-4(5*H*)-one (43)

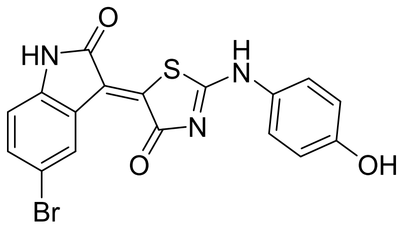

Following the general procedure D, the product was obtained as a red-brown solid after filtration and washing using water, ethanol and diethyl ether (46.2 mg, 0.11 mmol, 30%).

^{1}H NMR (700 MHz, DMSO-d_6) δ 11.60 (s, 1H), 11.25 (d, J = 7.9 Hz, 1H), 9.64 (s, 1H), 9.16 (d, J = 2.1 Hz, 1H), 7.63 – 7.60 (m, 1H), 7.52 (ddd, J = 10.9, 8.3, 2.1 Hz, 1H), 6.92 (d, J = 8.4 Hz, 1H), 6.88 (d, J = 8.3 Hz, 1H), 6.86 – 6.82 (m, 2H). **^{13}C NMR** (176 MHz, DMSO-d_6) δ 180.73, 172.82, 169.11, 155.79, 142.43, 139.24, 133.96, 130.31, 123.95, 123.03, 122.74, 116.41, 116.07, 113.87, 112.46. **LC-MS** (*m/z*): calculated for $[M+H]^+$ $C_{17}H_{10}BrN_3O_3S$, 416.0; found: 416.8.

5-(7-fluoro-2-oxoindolin-3-ylidene)-2-((4-hydroxyphenyl)amino)thiazol-4(5*H*)-one (44)

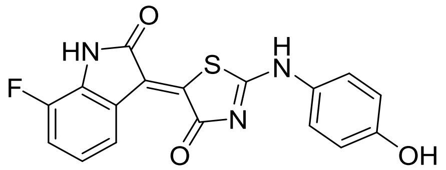

Following the general procedure D, the product was obtained as a red-brown solid after filtration and washing using water, ethanol and diethyl ether (34.3 mg, 0.1 mmol, 26%).

^{1}H NMR (600 MHz, DMSO-d_6) δ 11.62 (s, 1H), 11.57 (s, 1H), 9.63 (s, 1H), 8.83 (d, J = 7.9 Hz, 1H), 7.64 – 7.59 (m, 1H), 7.29 (dd, J = 10.2, 8.3 Hz, 1H), 7.10 (s, 2H), 6.87 – 6.80 (m, 2H). **^{13}C NMR** (151 MHz, DMSO-d_6) δ 180.51, 172.77, 169.31, 158.53, 155.74, 148.23 – 147.60 (m), 146.55 – 145.83 (m), 139.23, 130.51, 129.72, 124.42 (d, J = 3.8 Hz), 123.02,

122.72 (t, J = 5.7 Hz), 116.41, 116.05. **^{19}F NMR** (565 MHz, DMSO-d_6) δ -133.16 (dd, J = 10.3, 5.2 Hz), -133.21 (dd, J = 10.3, 5.2 Hz). **HRMS-**ESI (*m/z*): calculated for $[M+H]^+$ $C_{17}H_{10}FN_3O_3S$, 356.0499; found: 356.0498.

NMR SPECTRA

^{1}**H NMR** Spectrum of **Ia** (GG-352) (700 MHz, DMSO-d_6):

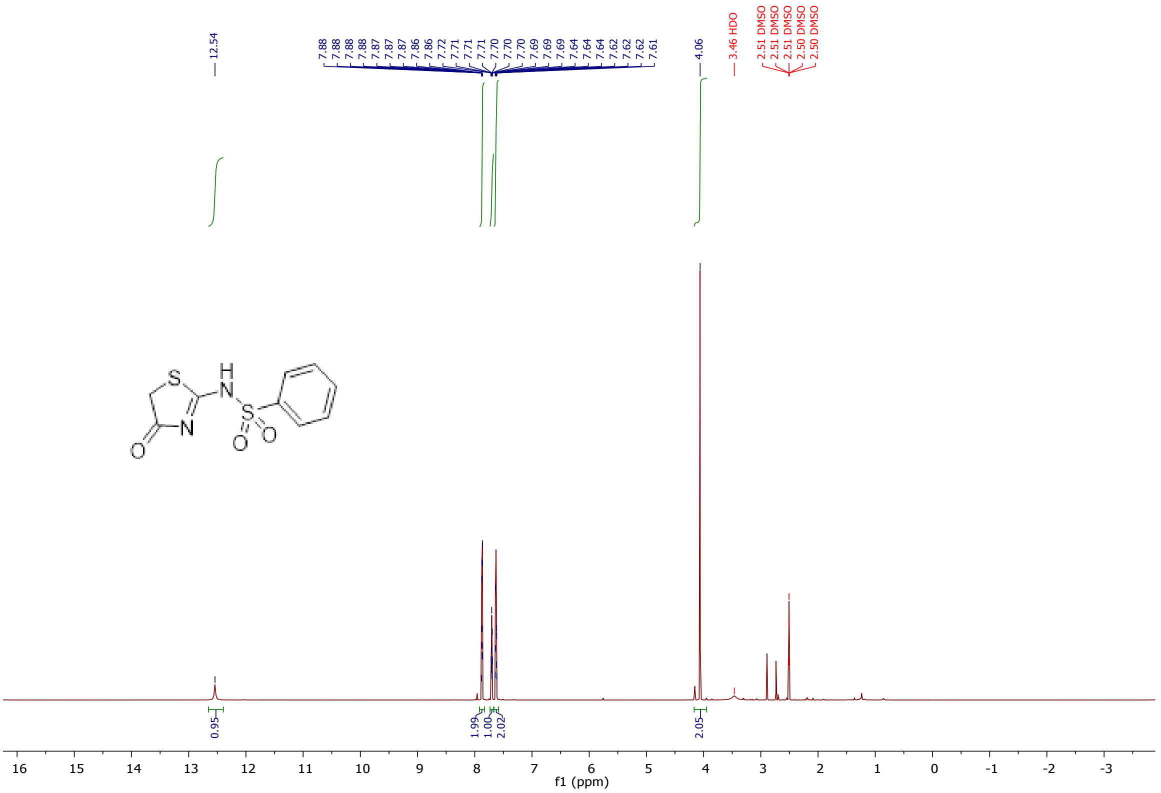

^{13}**C NMR** Spectrum of **Ia** (GG-352) (174 MHz, DMSO-d_6):

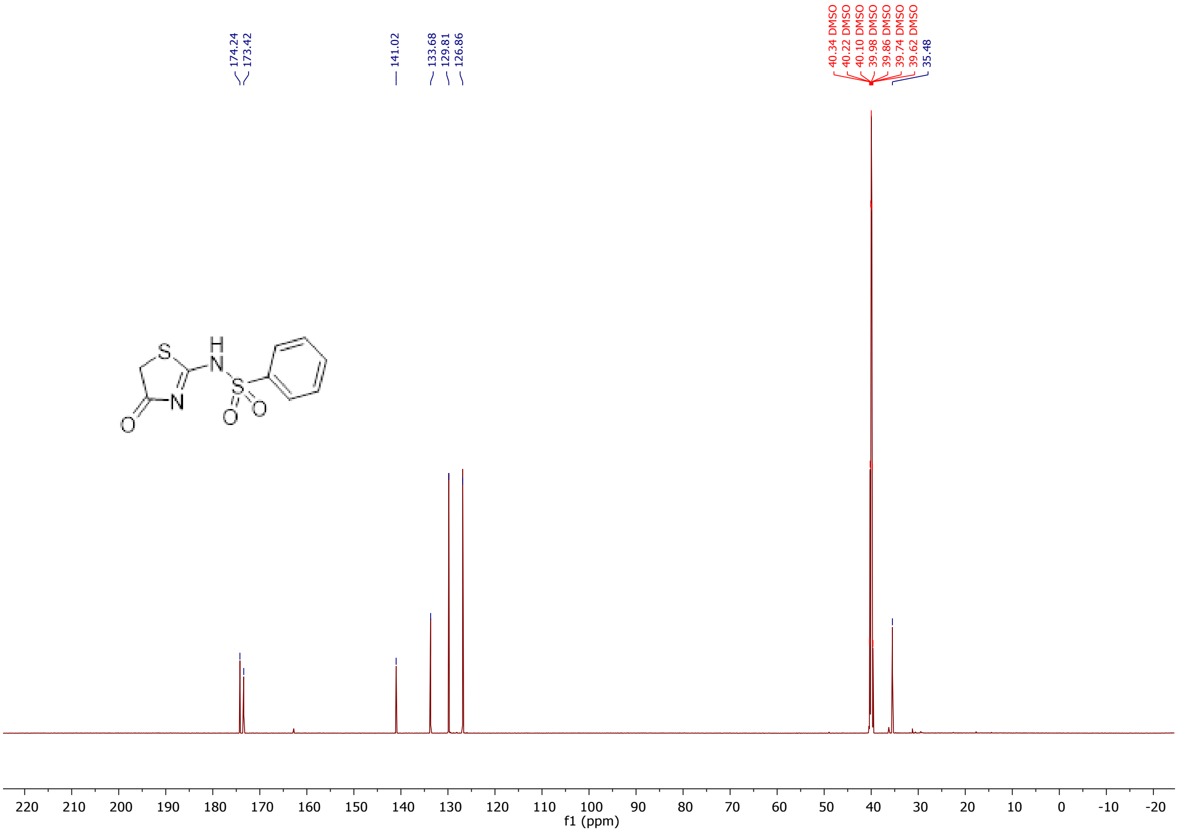

^{1}**H NMR** Spectrum of **Ib** (600 MHz, DMSO-d_6):

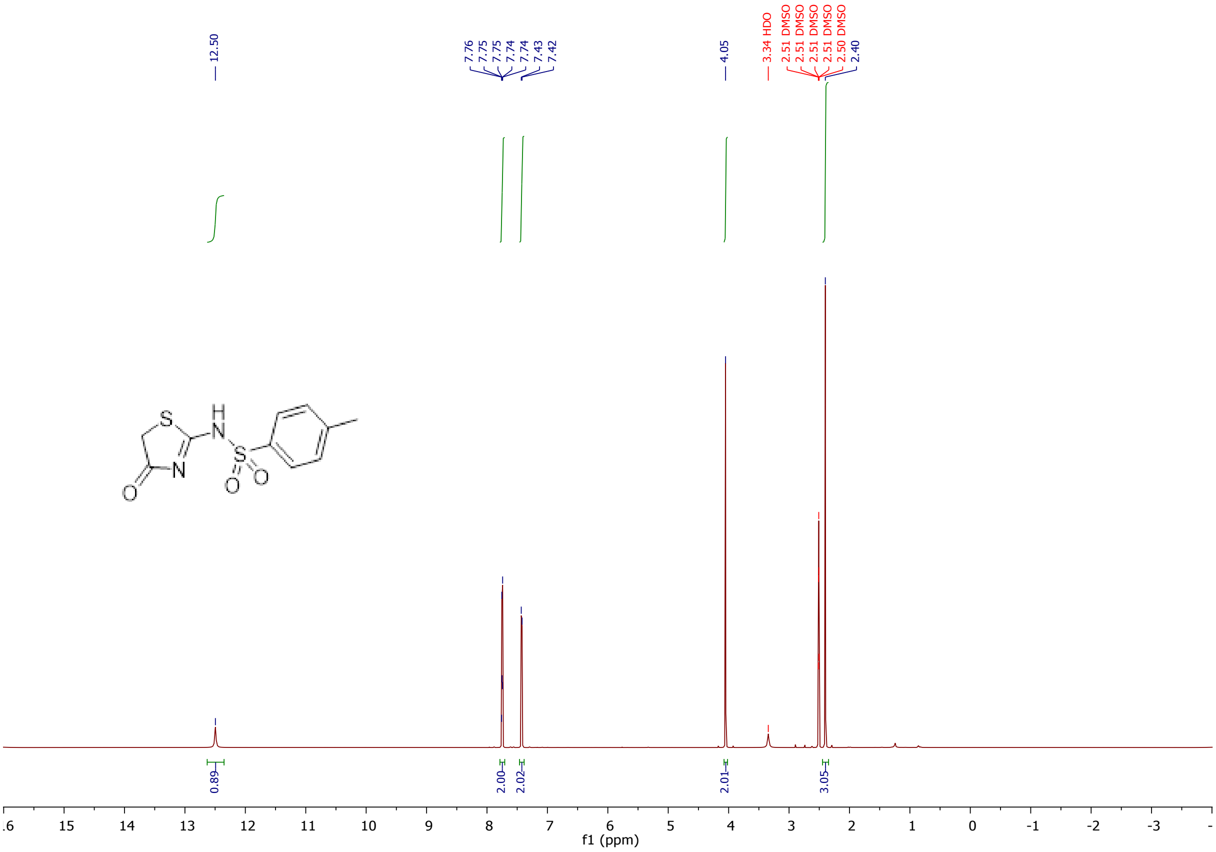

^{13}C NMR Spectrum of **Ib** (151 MHz, DMSO-d_6):

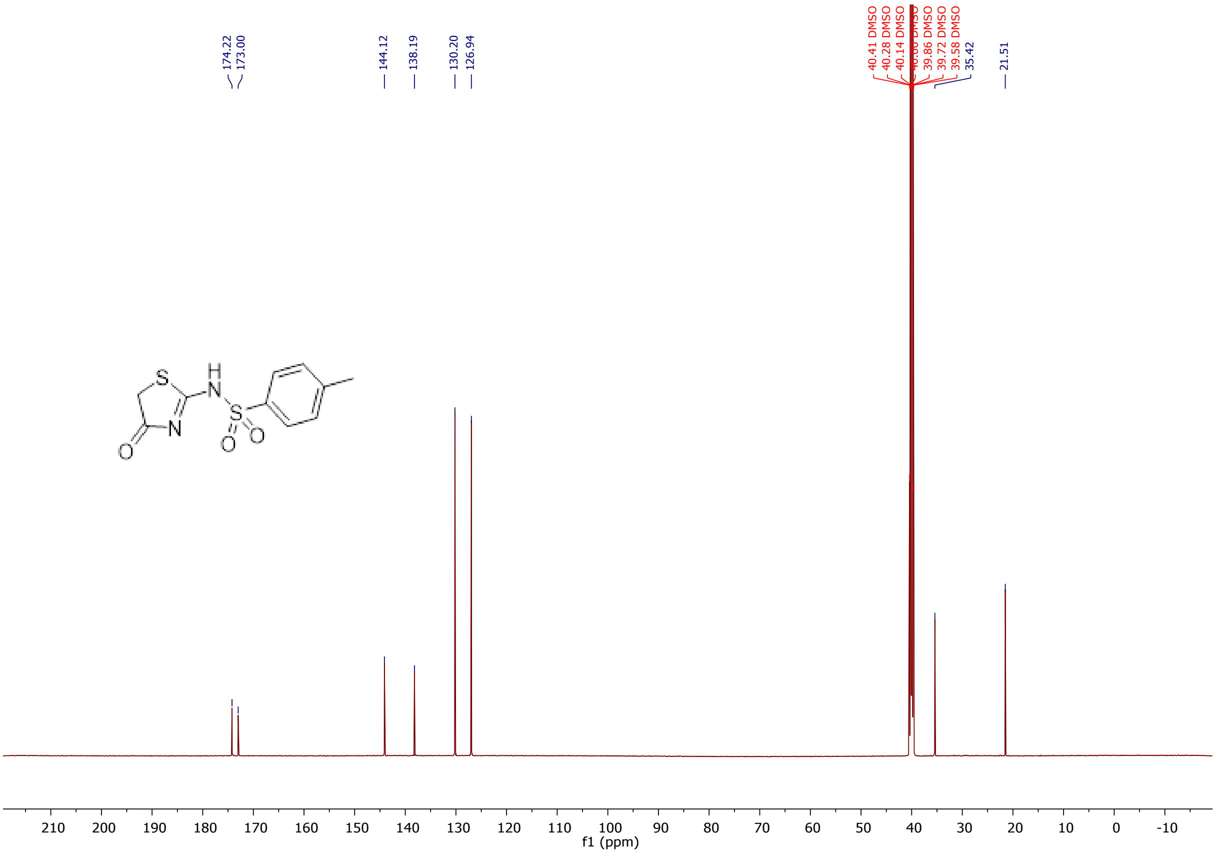

^{1}H NMR Spectrum of **Ic** (600 MHz, DMSO-d_6):

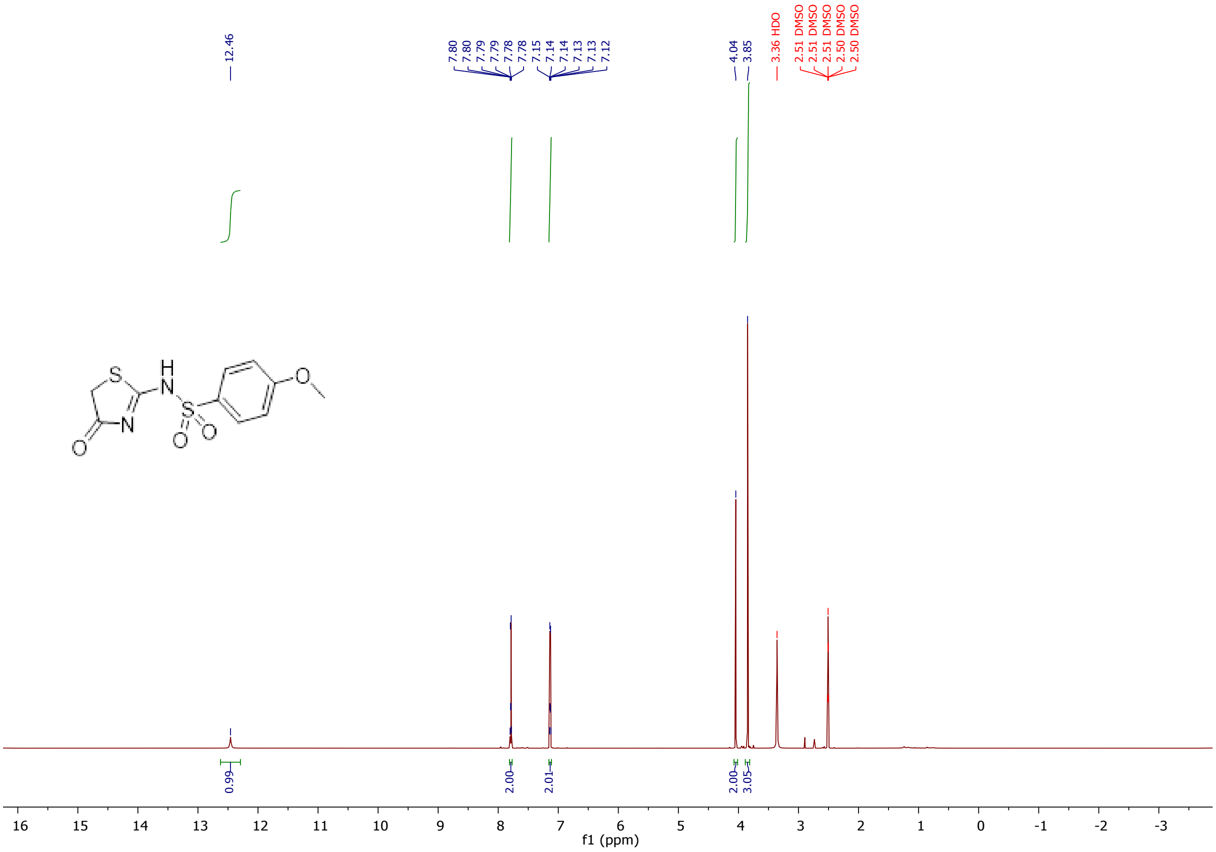

^{13}C NMR Spectrum of **Ic** (151 MHz, DMSO-d_6):

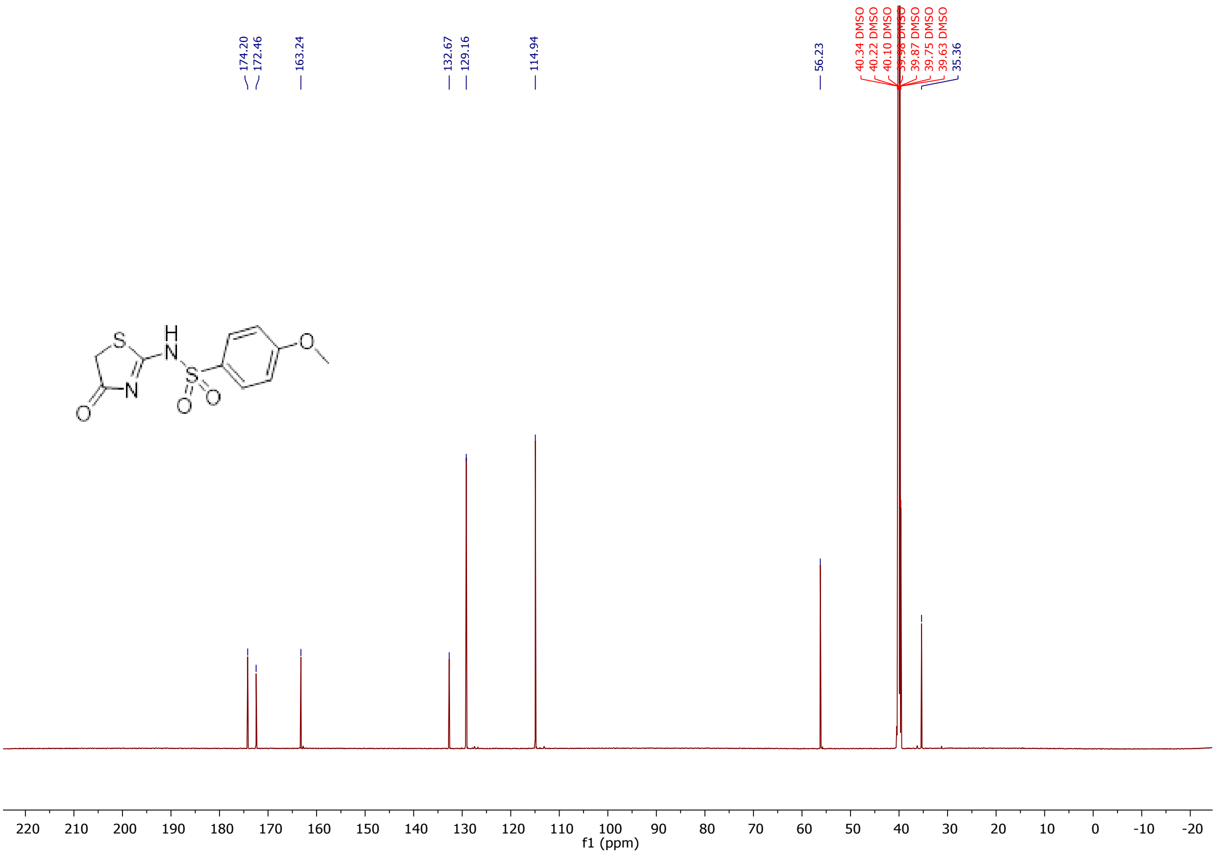

^{1}H NMR Spectrum of **Id** (700 MHz, DMSO-d_6):

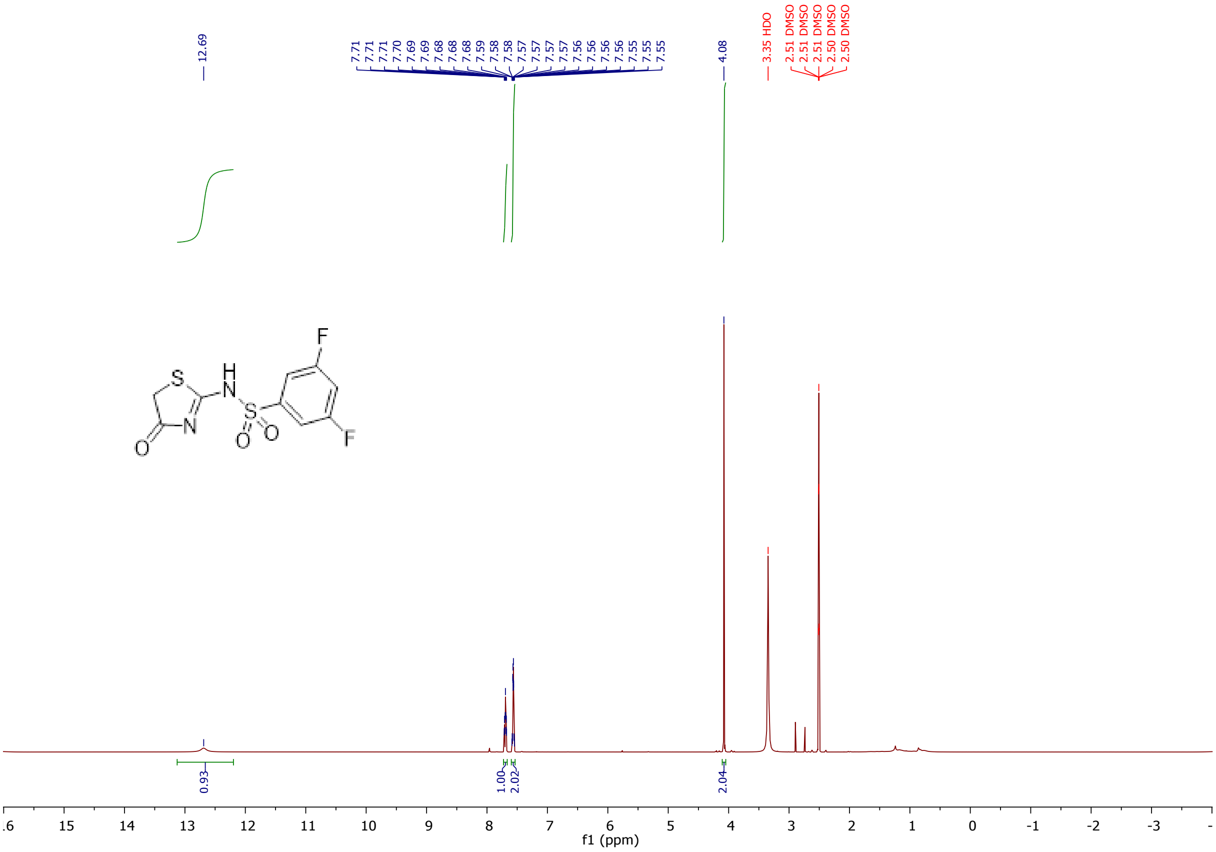

^{13}C NMR Spectrum of **Id** (174 MHz, DMSO-d_6):

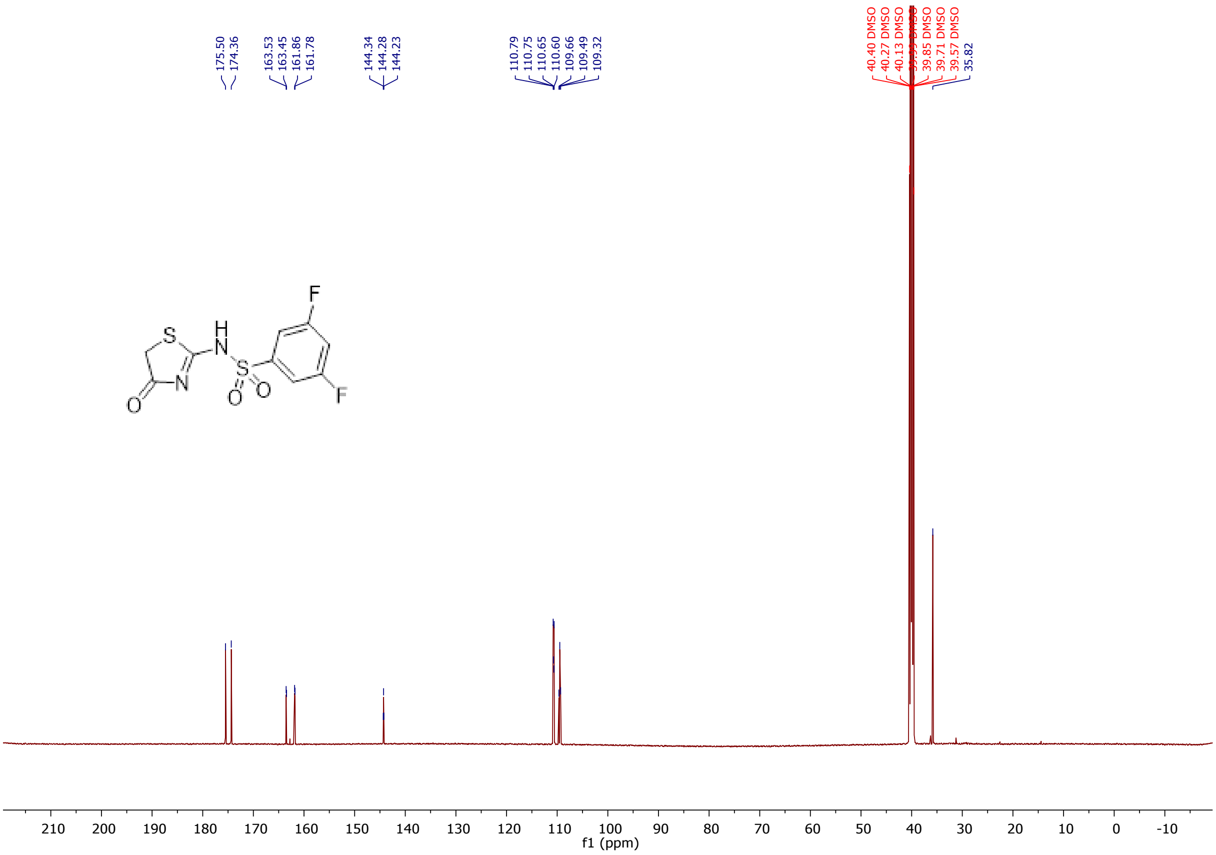

^{1}H NMR Spectrum of **Ie** (600 MHz, DMSO-d_6):

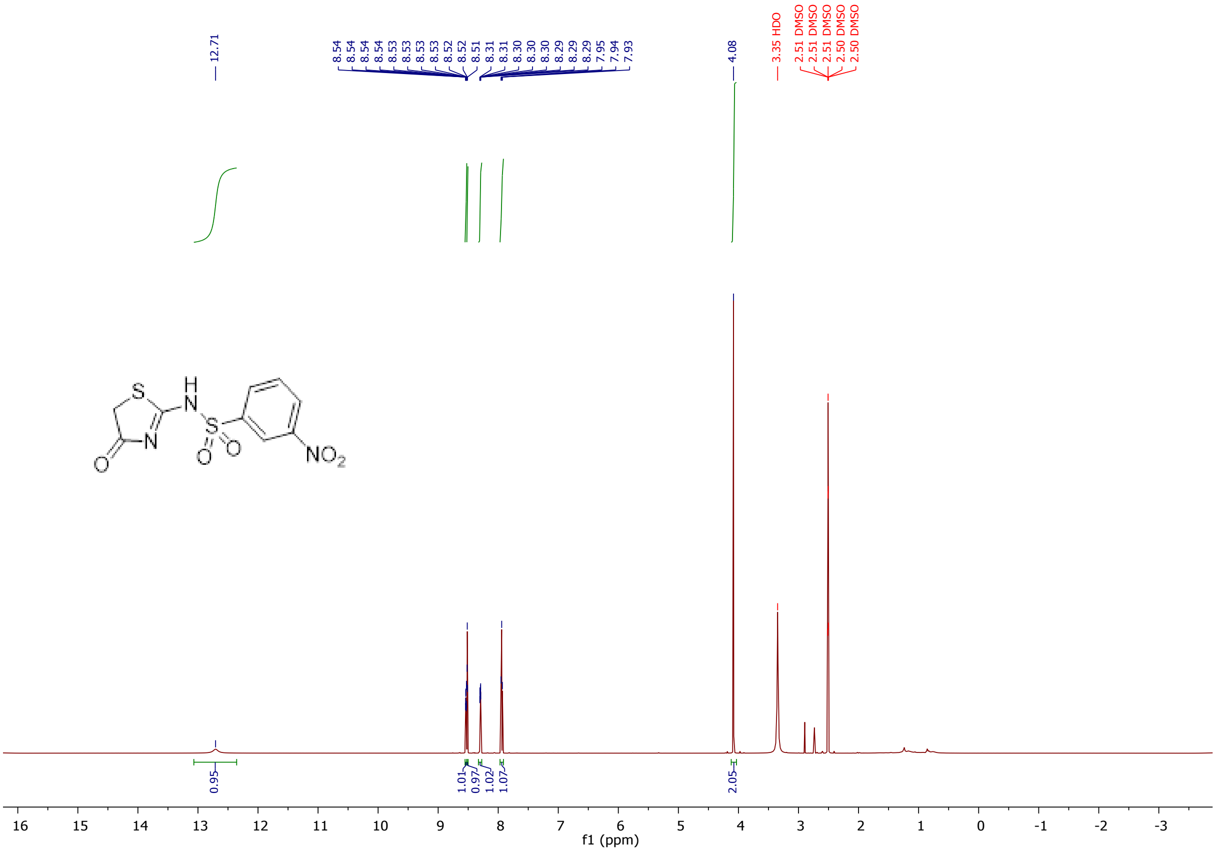

^{13}C NMR Spectrum of **Ie** (151 MHz, DMSO-d_6):

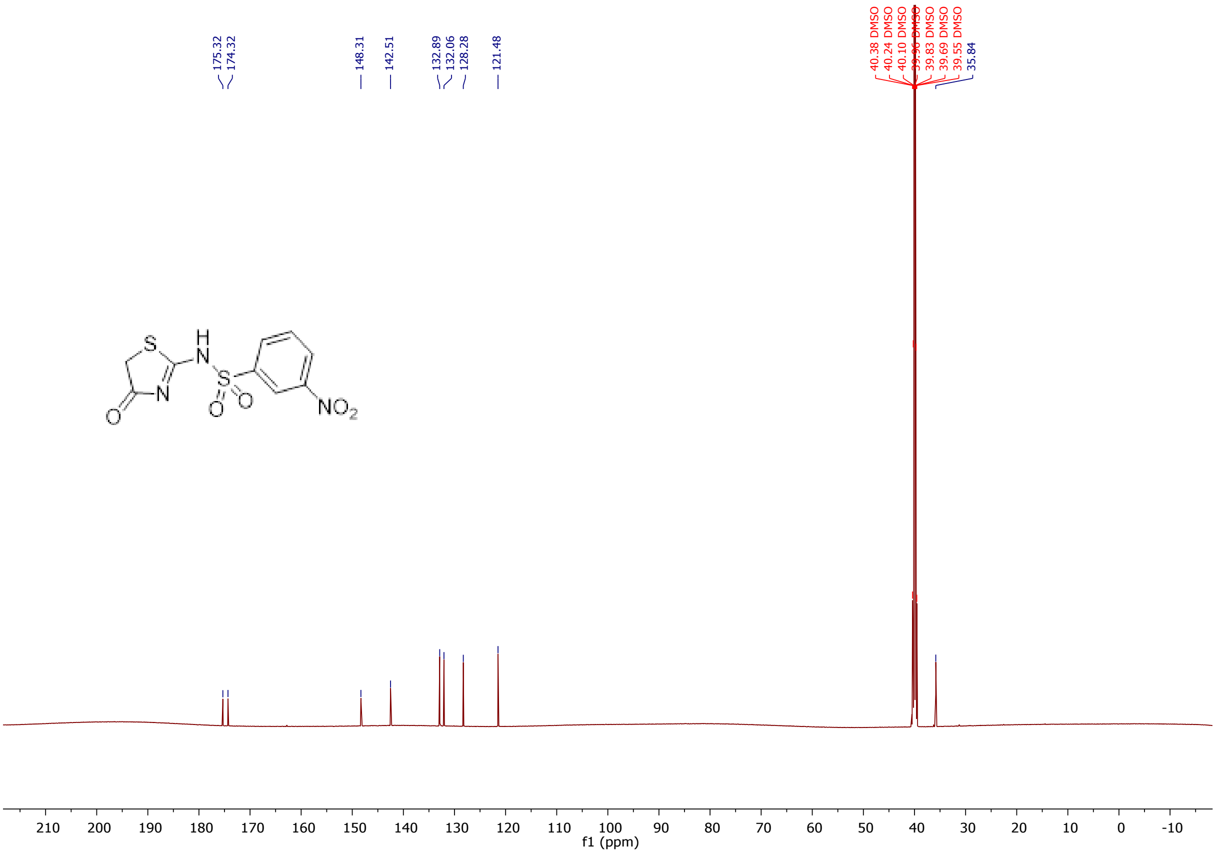

^{1}H NMR Spectrum of **If** (600 MHz, DMSO-d_6):

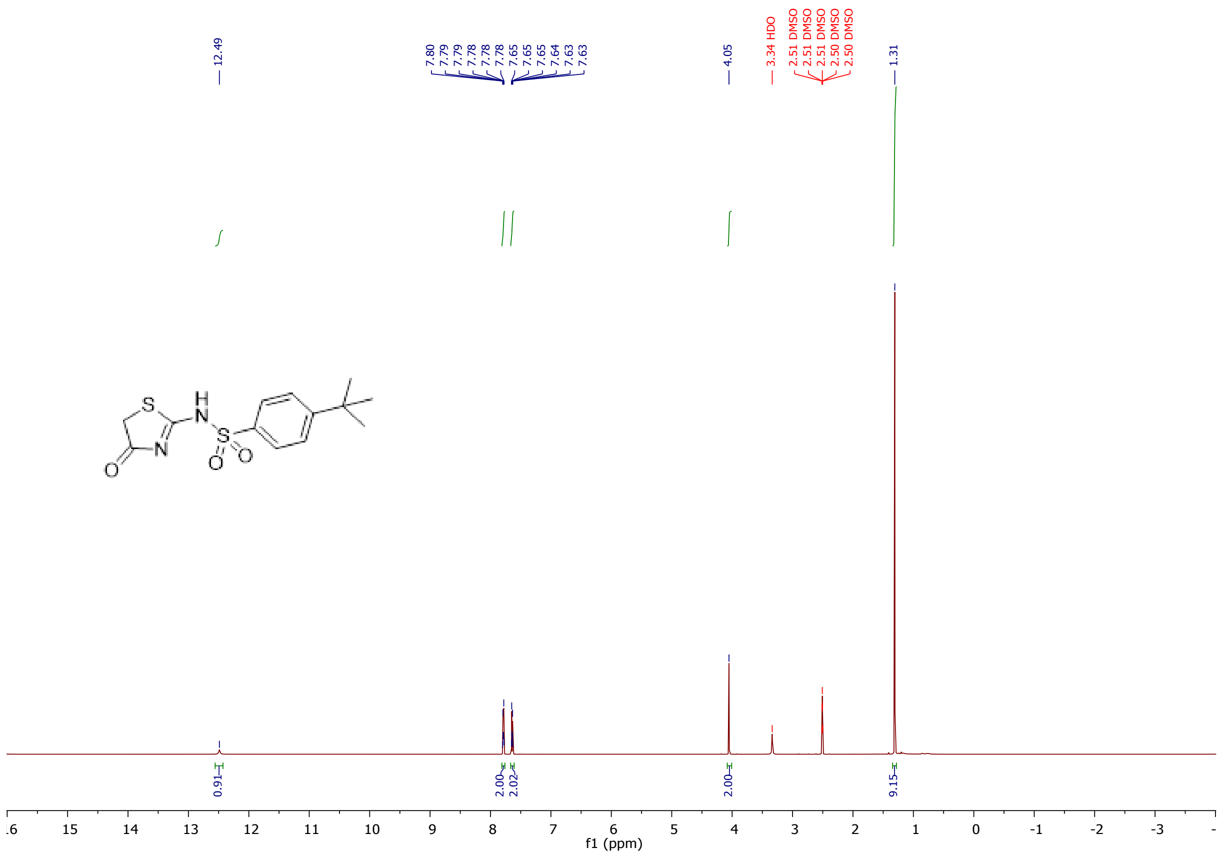

^{13}C NMR Spectrum of **If** (151 MHz, DMSO-d_6):

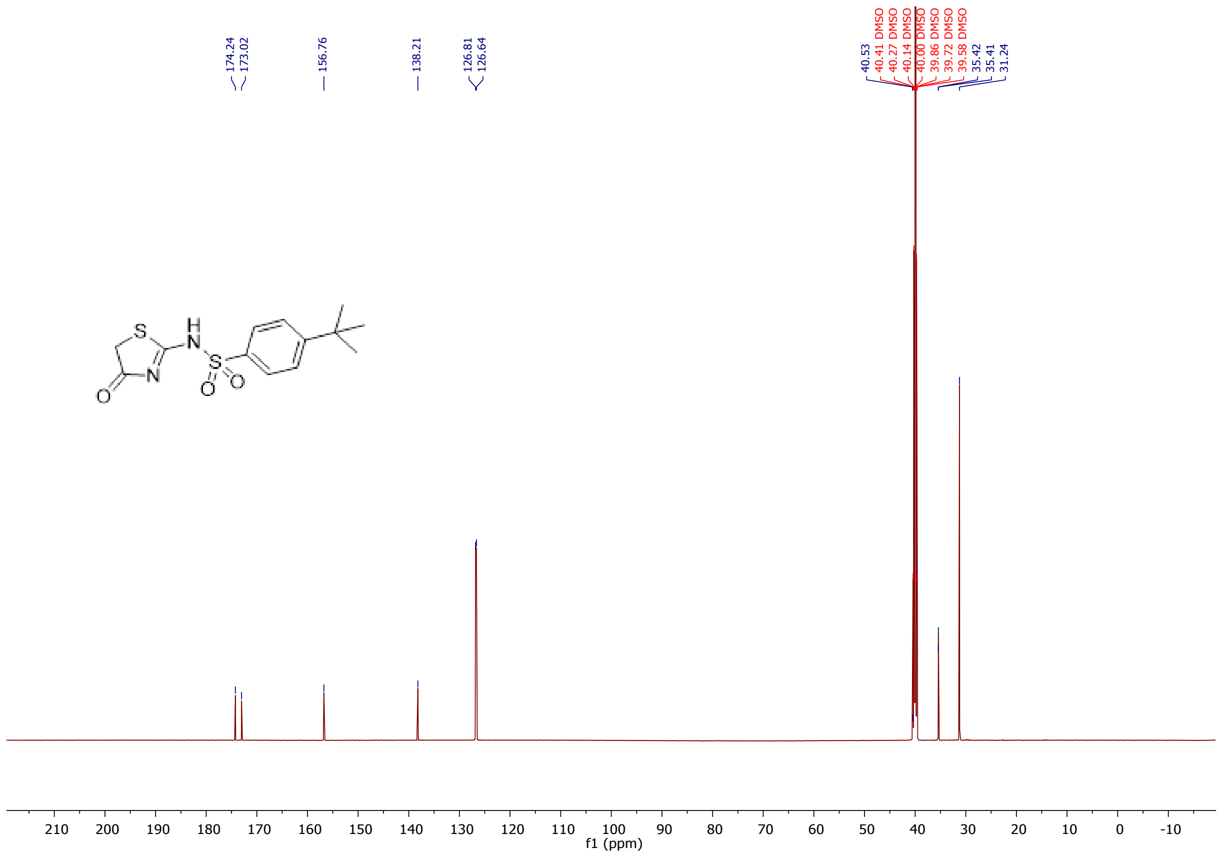

^{1}H NMR Spectrum of **Ig** (600 MHz, DMSO-d_6):

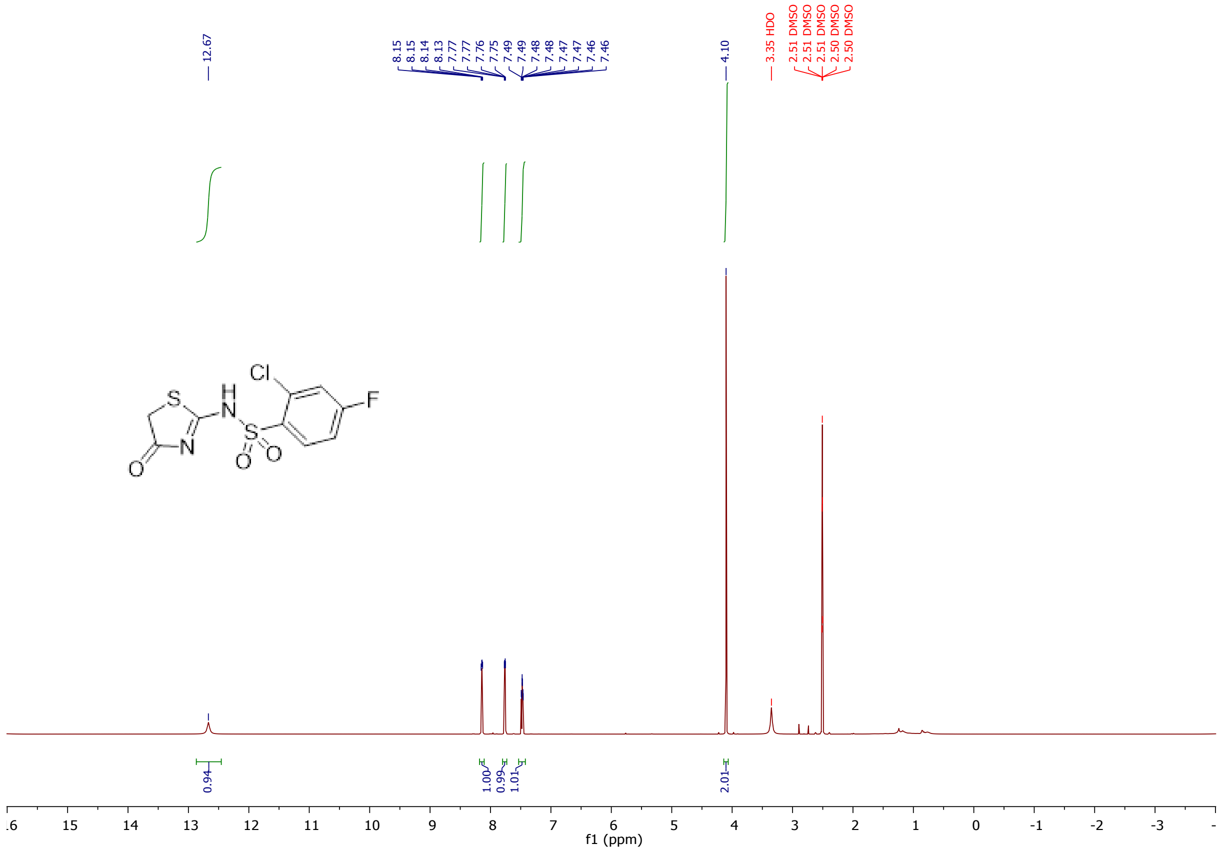

^{13}C NMR Spectrum of **Ig** (151 MHz, DMSO-d_6):

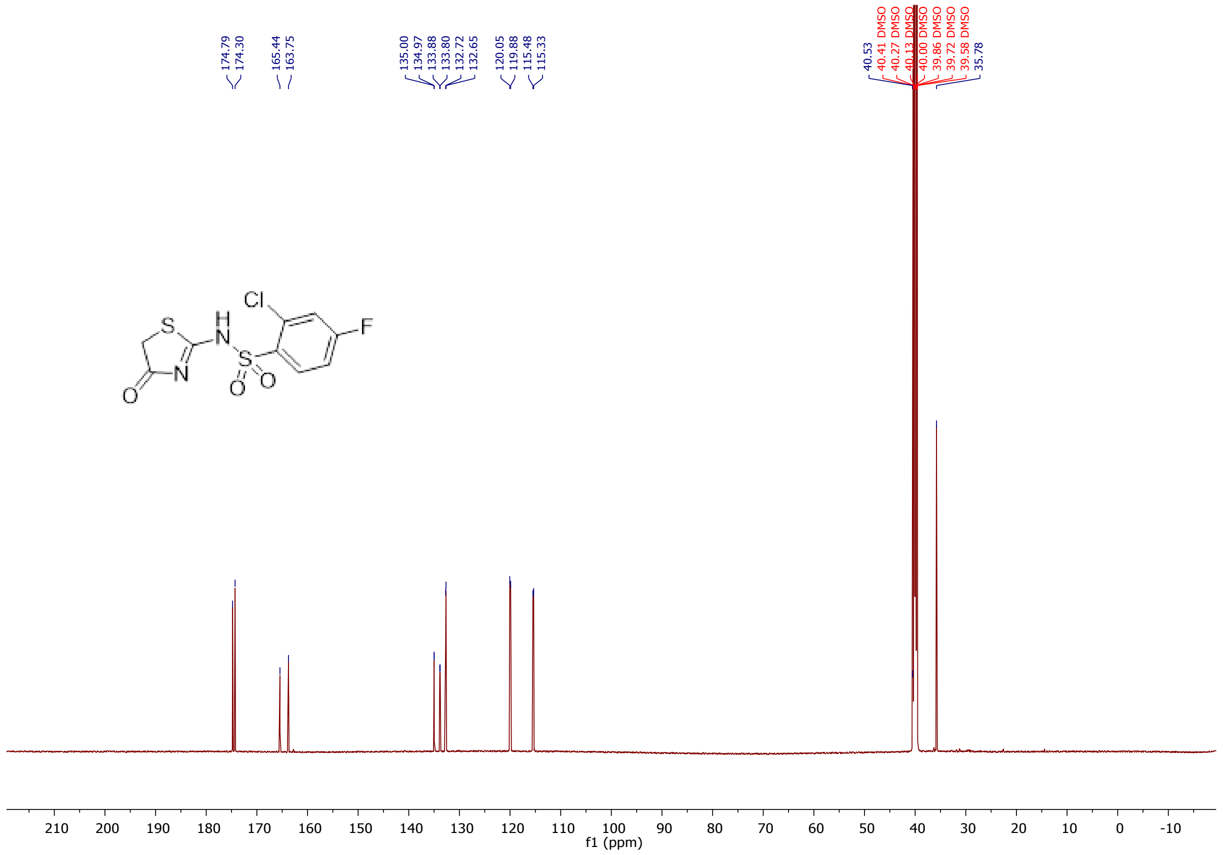

^{1}H NMR Spectrum of **Ih** (700 MHz, DMSO-d_6):

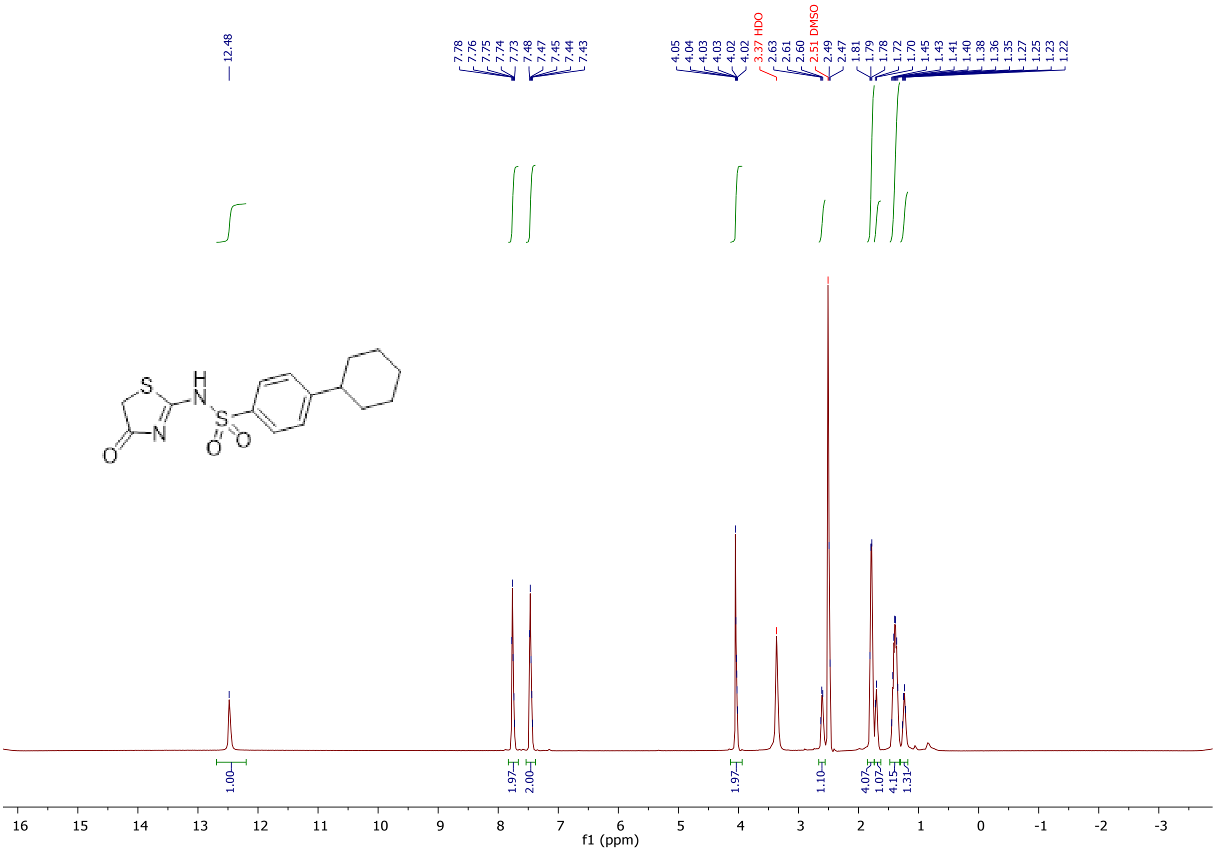

^{13}**C NMR** Spectrum of **Ih** (174 MHz, DMSO-d_6):

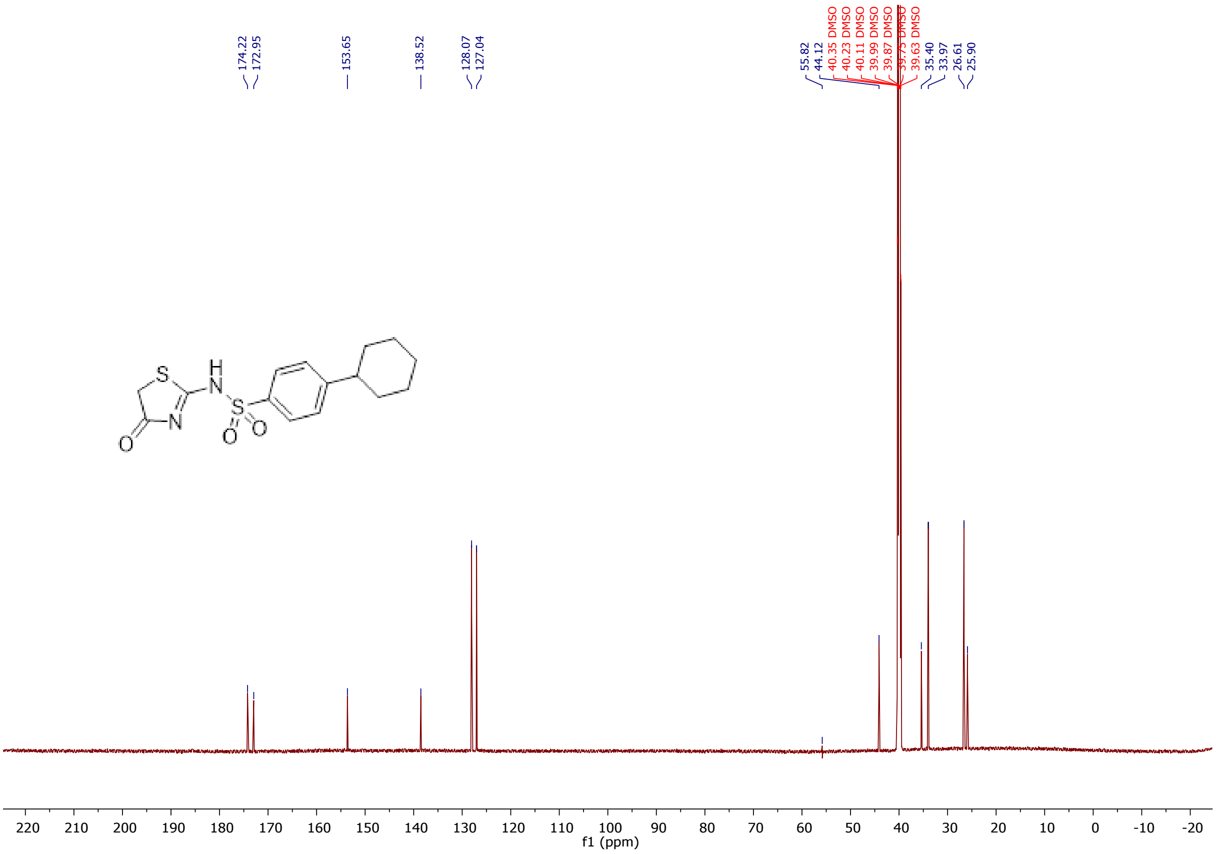

^{1}**H NMR** Spectrum of **Ii** (700 MHz, DMSO-d_6):

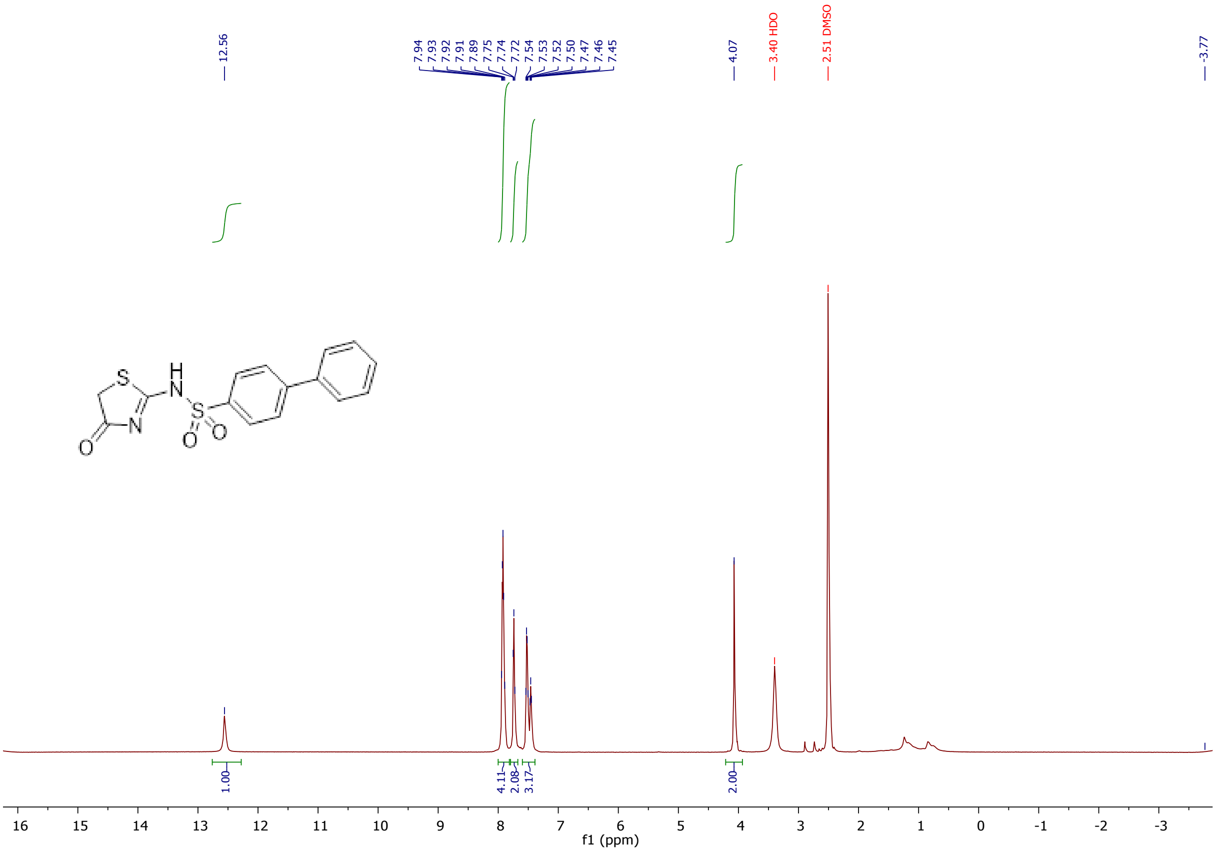

^{13}C NMR Spectrum of **Ii** (174 MHz, DMSO-d_6):

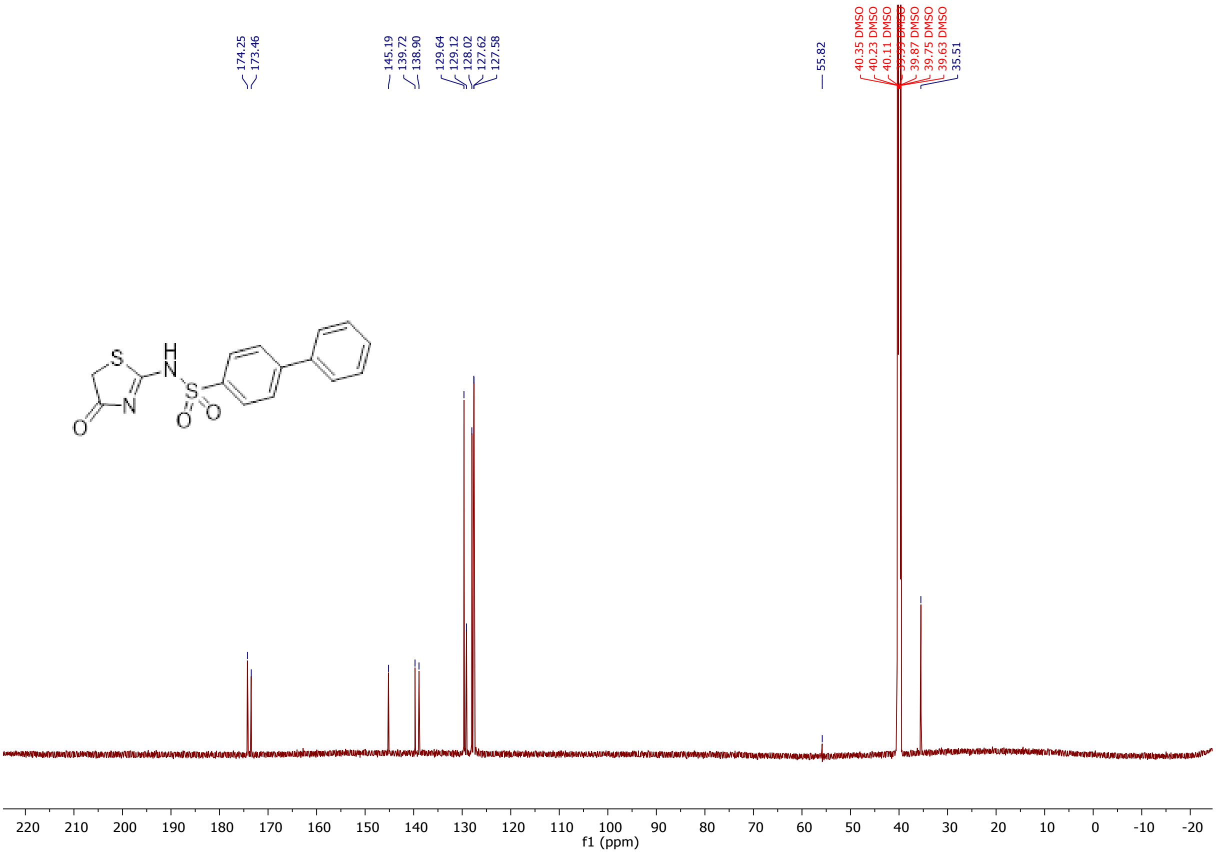

^{1}H NMR Spectrum of **Ij** (700 MHz, DMSO-d_6):

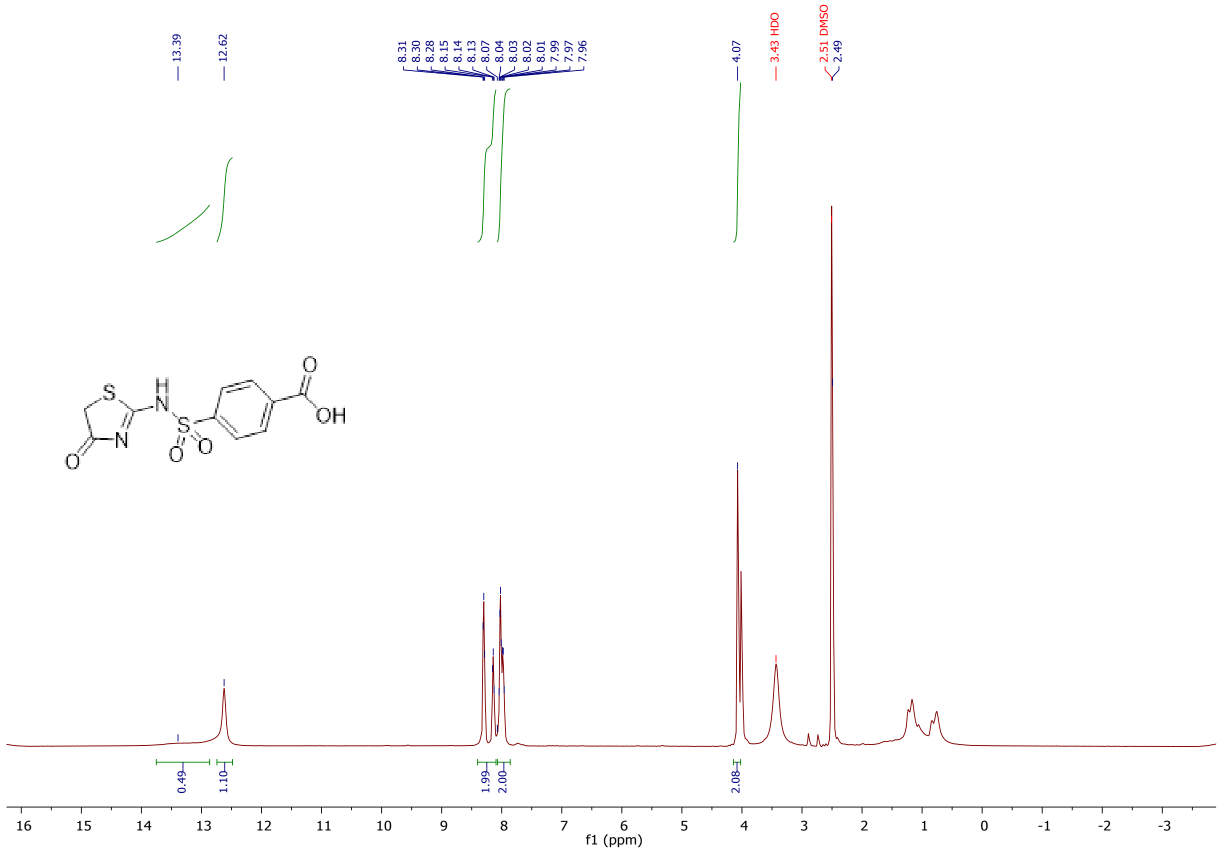

^{13}C NMR Spectrum of **Ij** (174 MHz, DMSO-d_6):

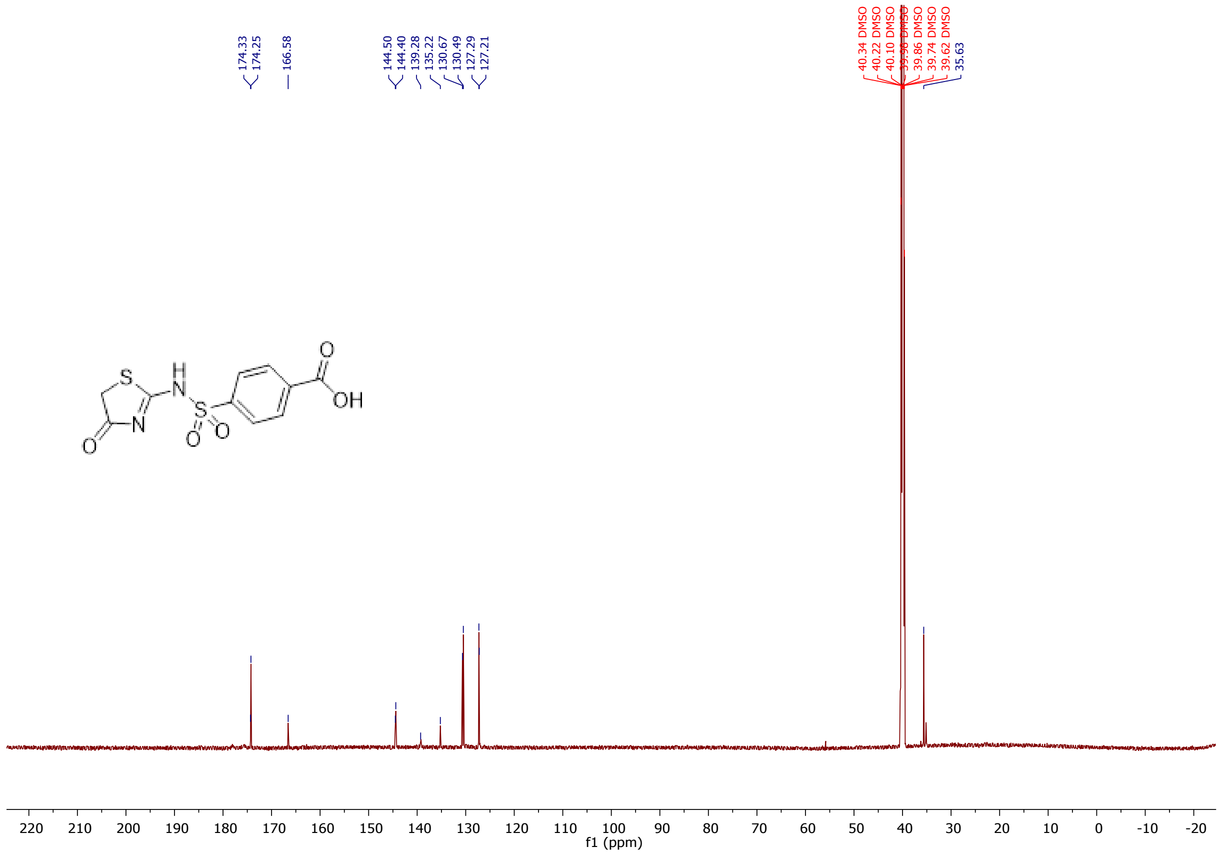

^{1}H NMR Spectrum of **Ik** (700 MHz, DMSO-d_6):

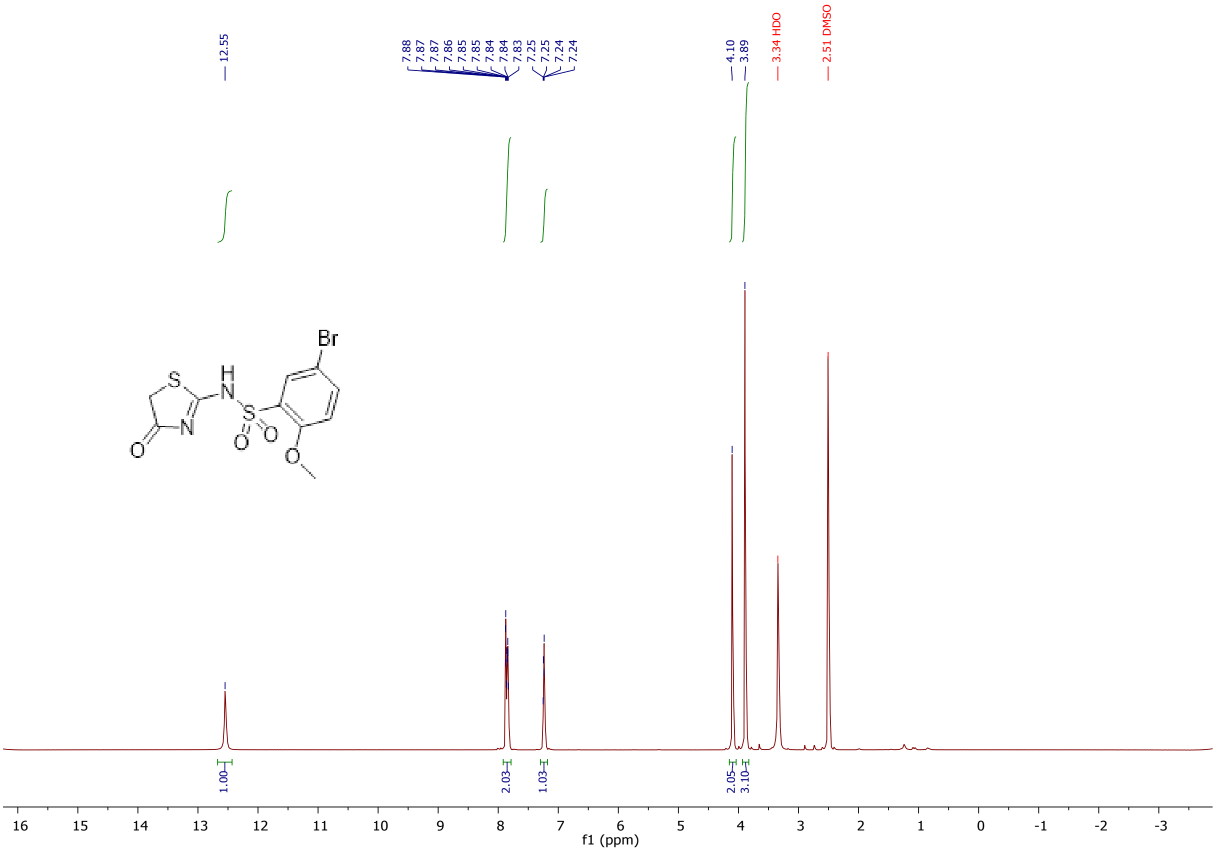

^{13}**C NMR** Spectrum of **Ik** (174 MHz, DMSO-d_6):

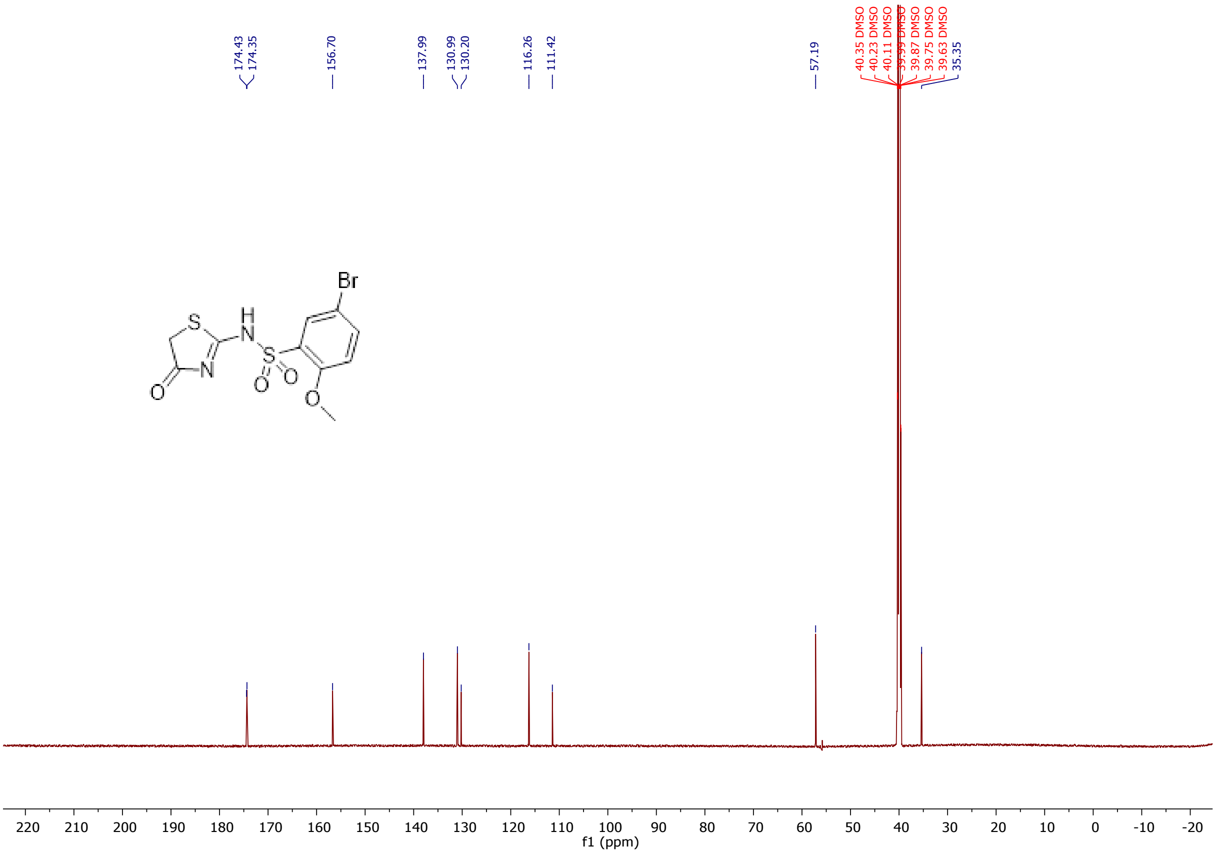

^{1}**H NMR** Spectrum of **Il** (600 MHz, DMSO-d_6):

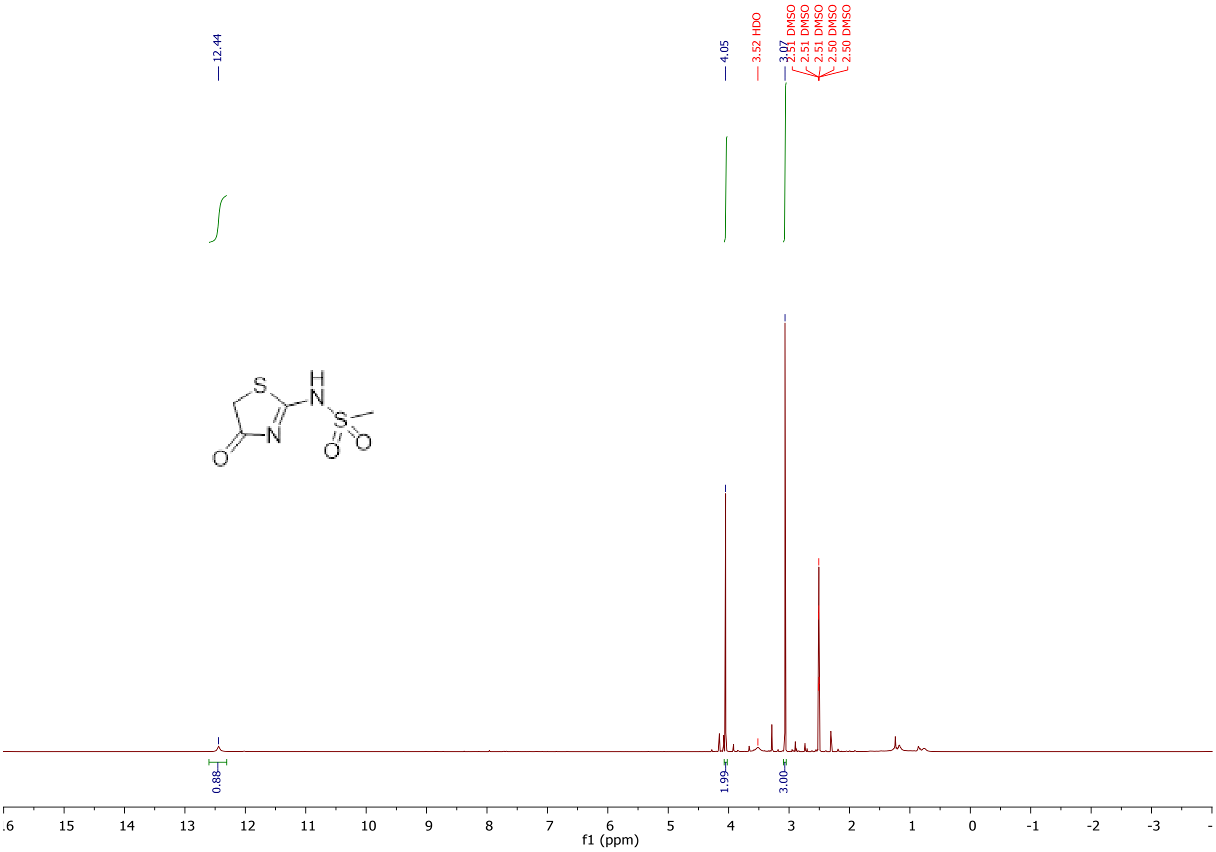

^{13}C **NMR** Spectrum of **Il** (151 MHz, DMSO-d_6):

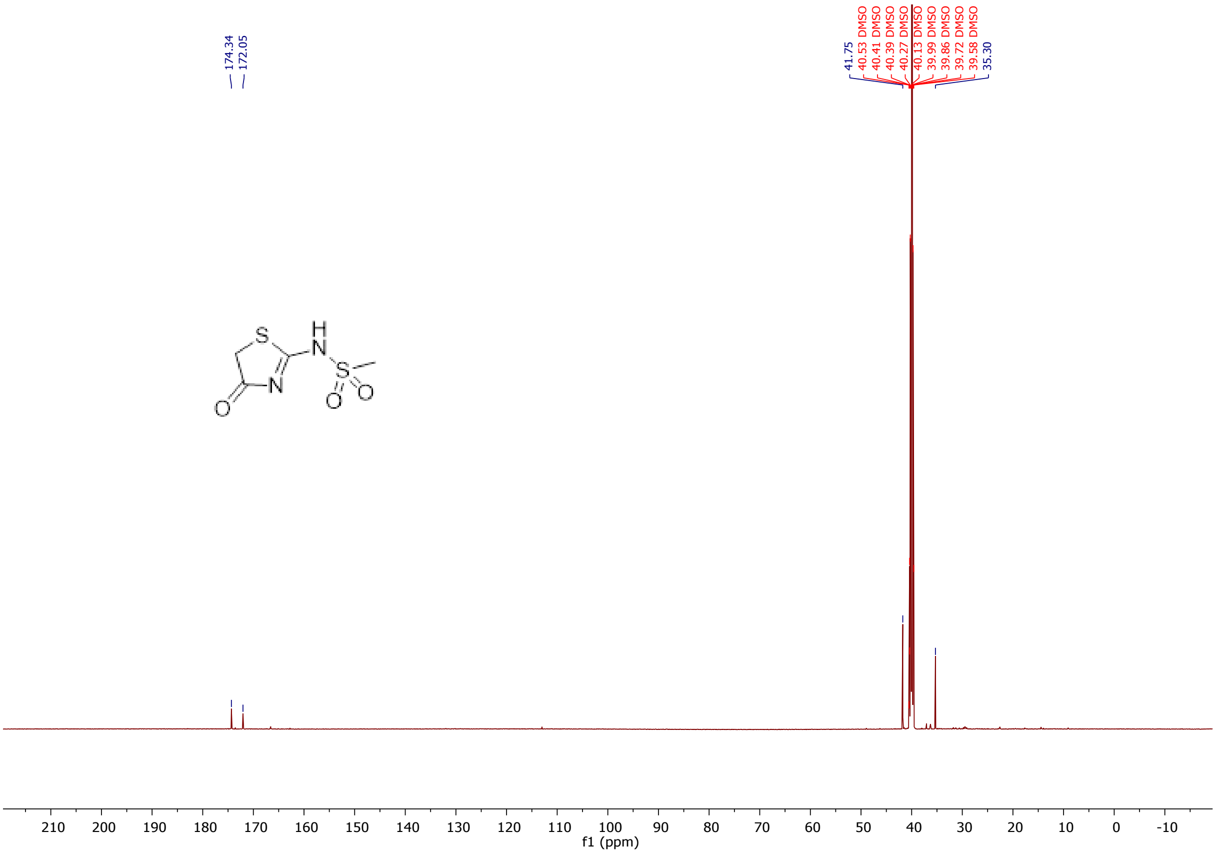

^{1}H **NMR** Spectrum of **Im** (600 MHz, DMSO-d_6):

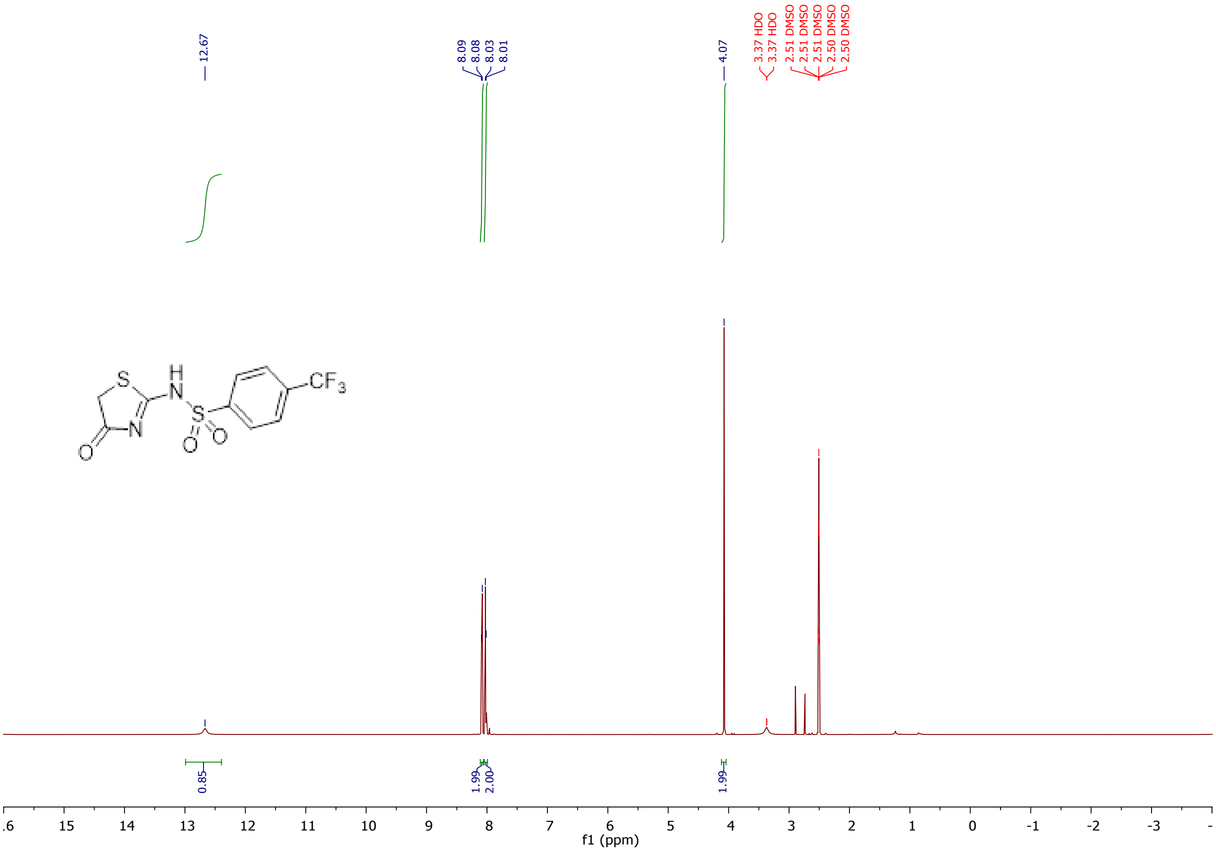

^{13}C NMR Spectrum of **Im** (151 MHz, DMSO-d_6):

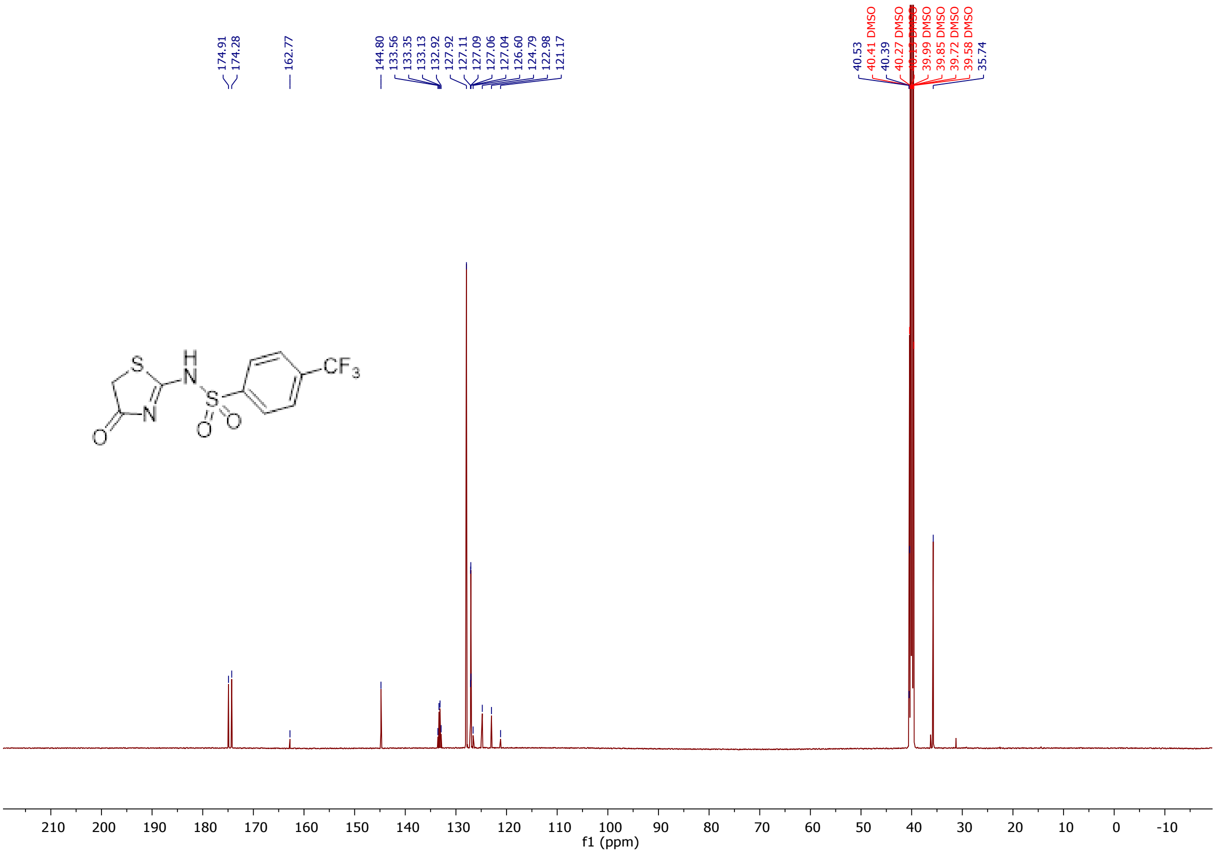

^{1}H NMR Spectrum of **In** (700 MHz, DMSO-d_6):

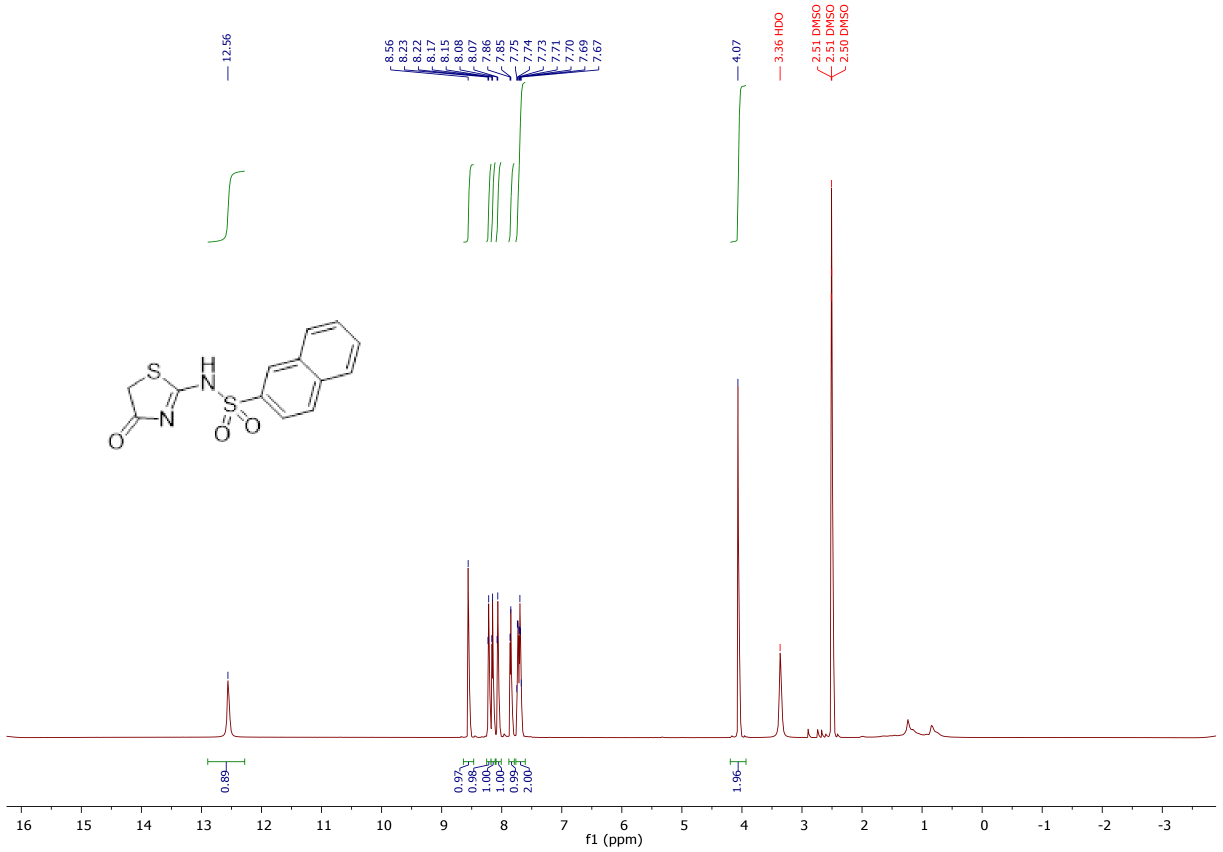

^{13}**C NMR** Spectrum of **In** (174 MHz, DMSO-d_6):

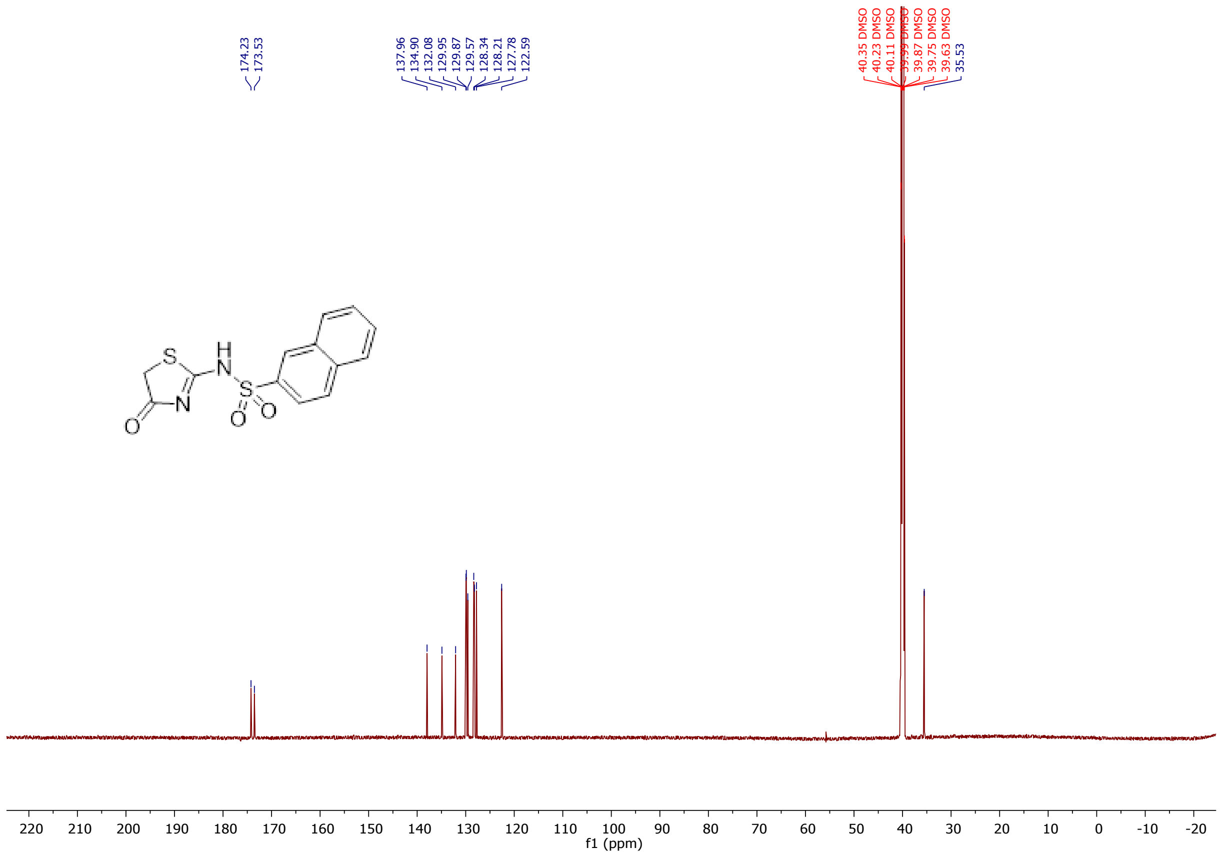

^{1}**H NMR** Spectrum of **Io** (700 MHz, DMSO-d_6):

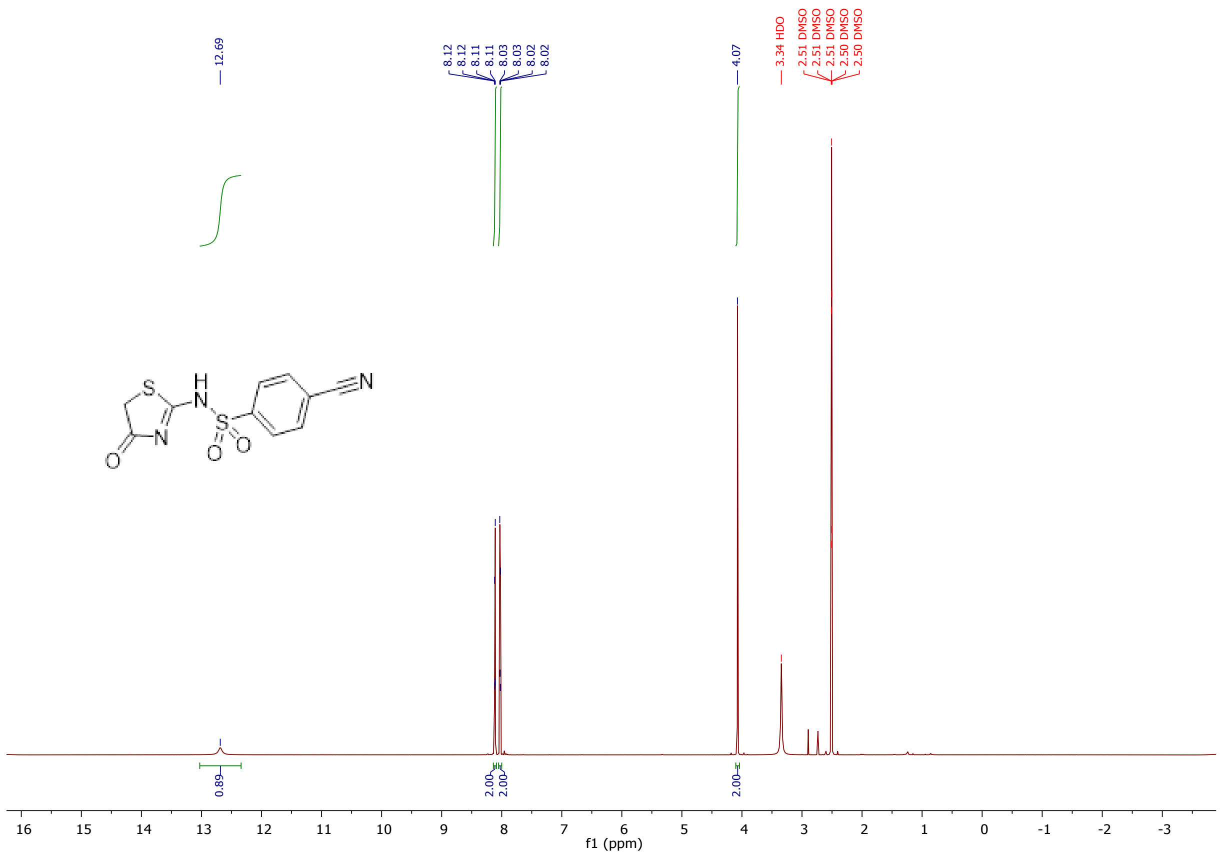

^{13}C NMR Spectrum of **Io** (174 MHz, DMSO-d_6):

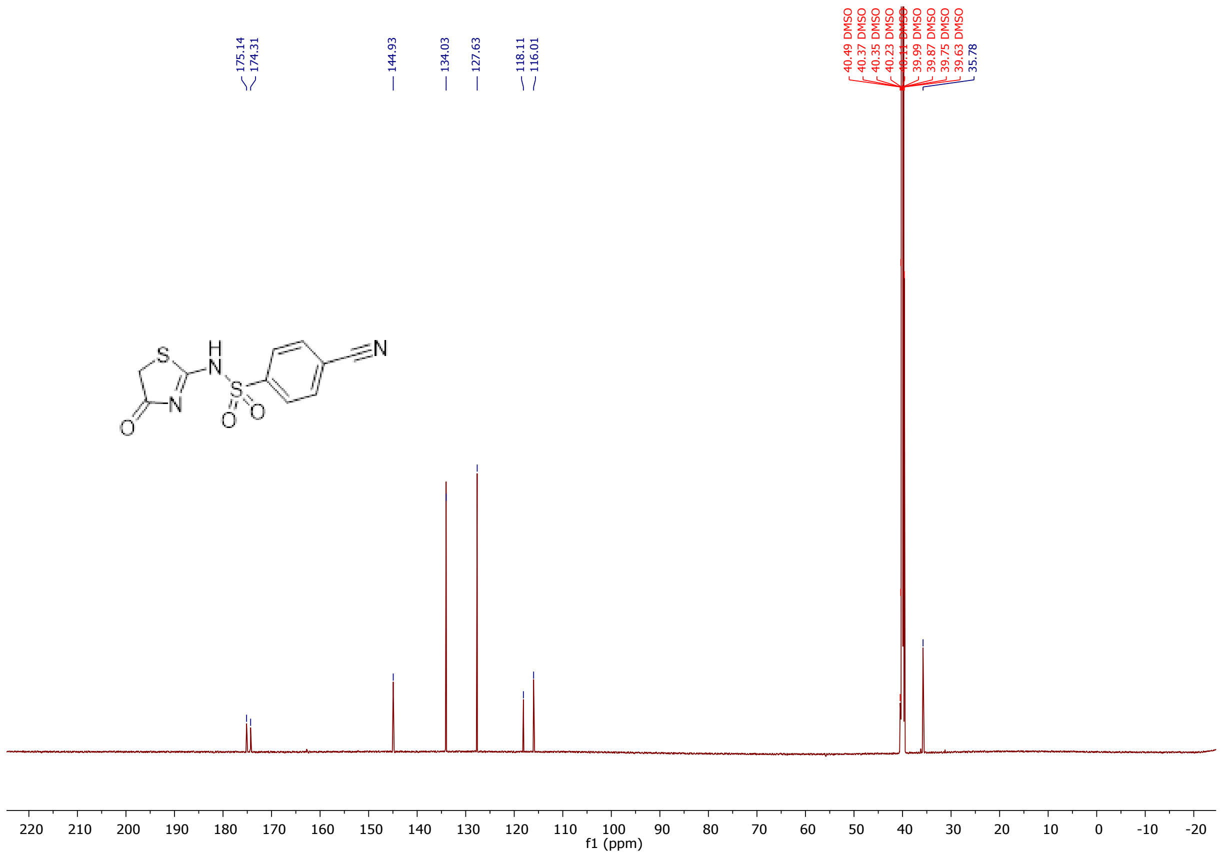

^{1}H NMR Spectrum of **Ip** (600 MHz, DMSO-d_6):

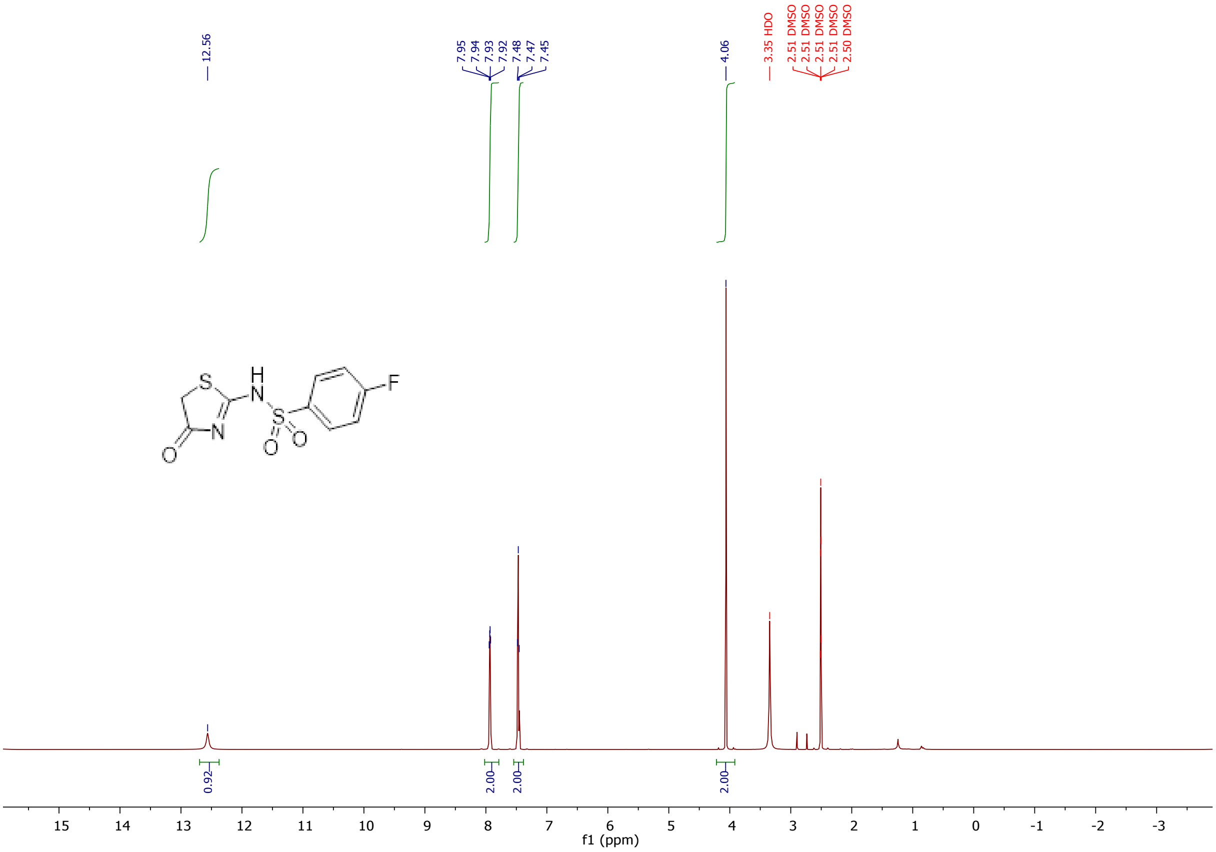

^{13}**C NMR** Spectrum of **Ip** (151 MHz, DMSO-d_6):

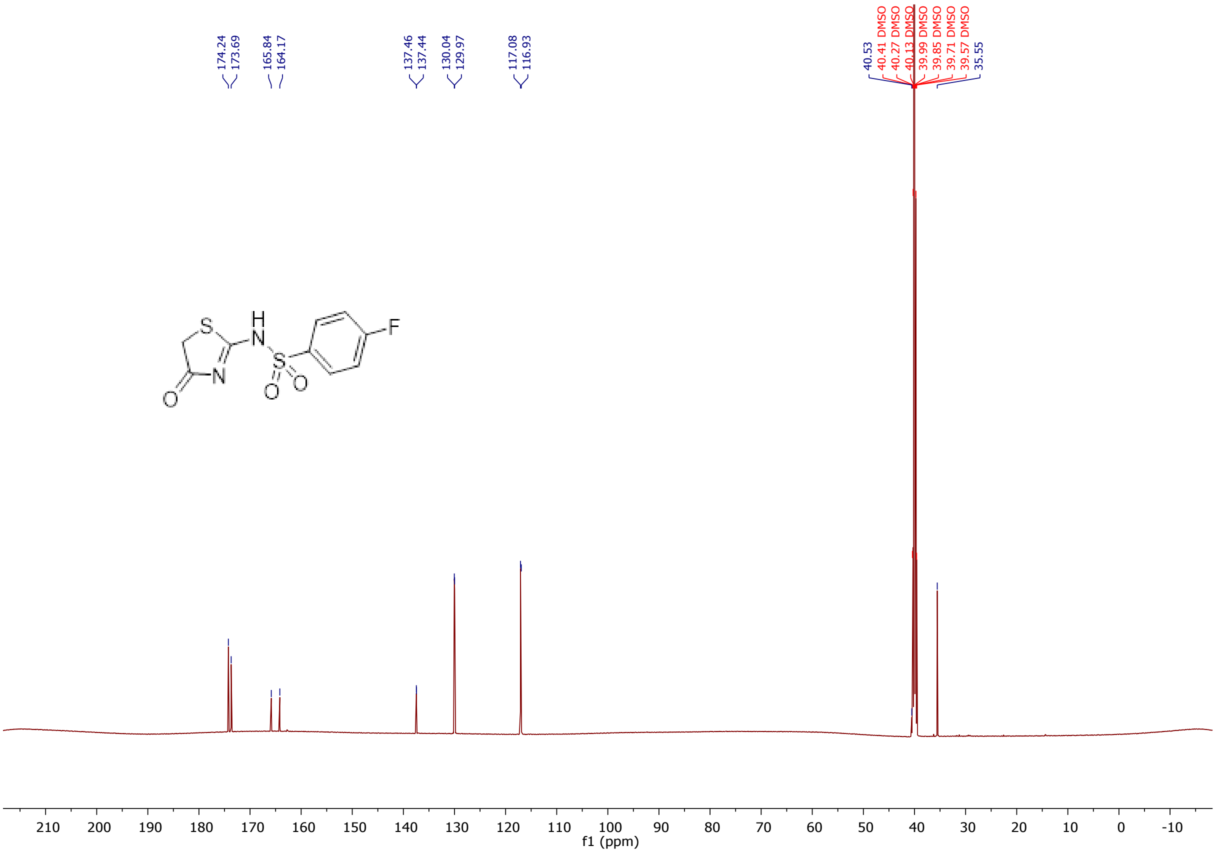

^{1}**H NMR** Spectrum of **Iq** (700 MHz, DMSO-d_6):

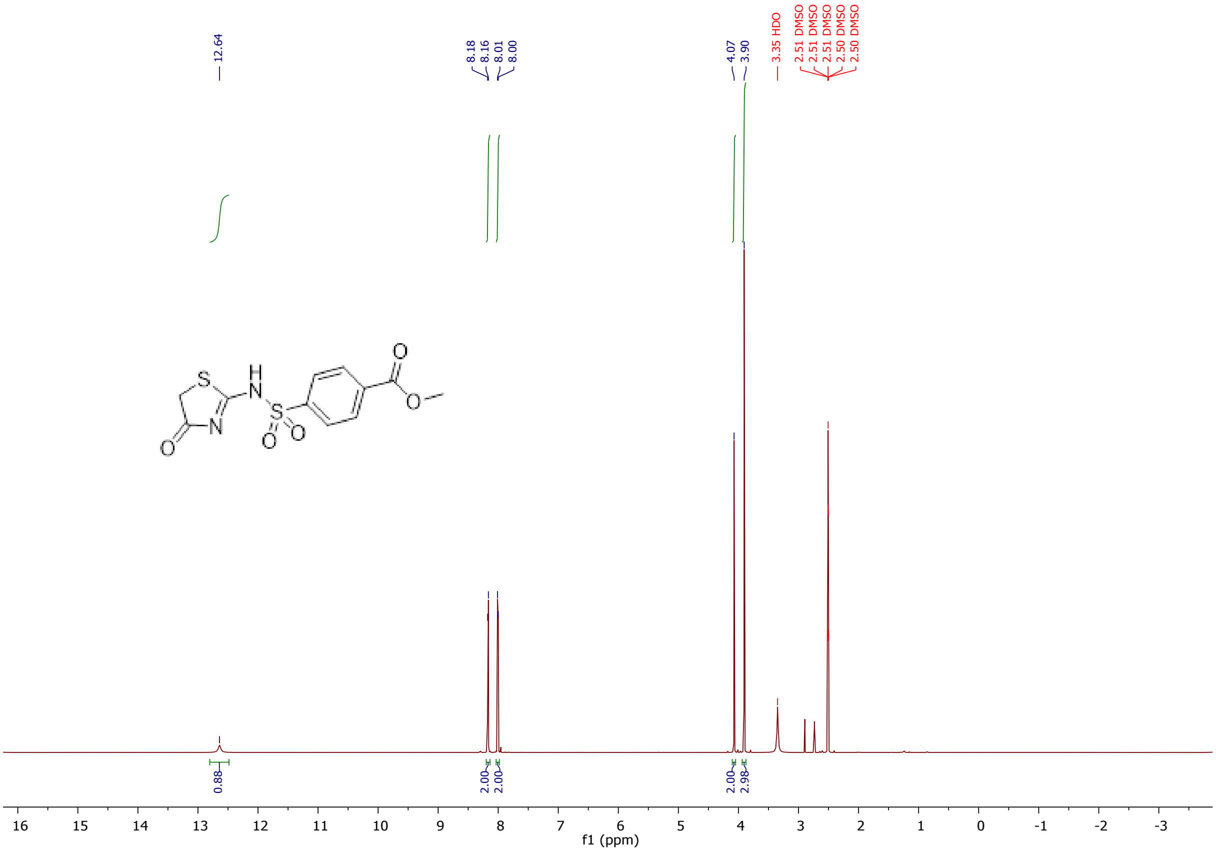

^{13}C NMR Spectrum of **Iq** (174 MHz, DMSO-d_6):

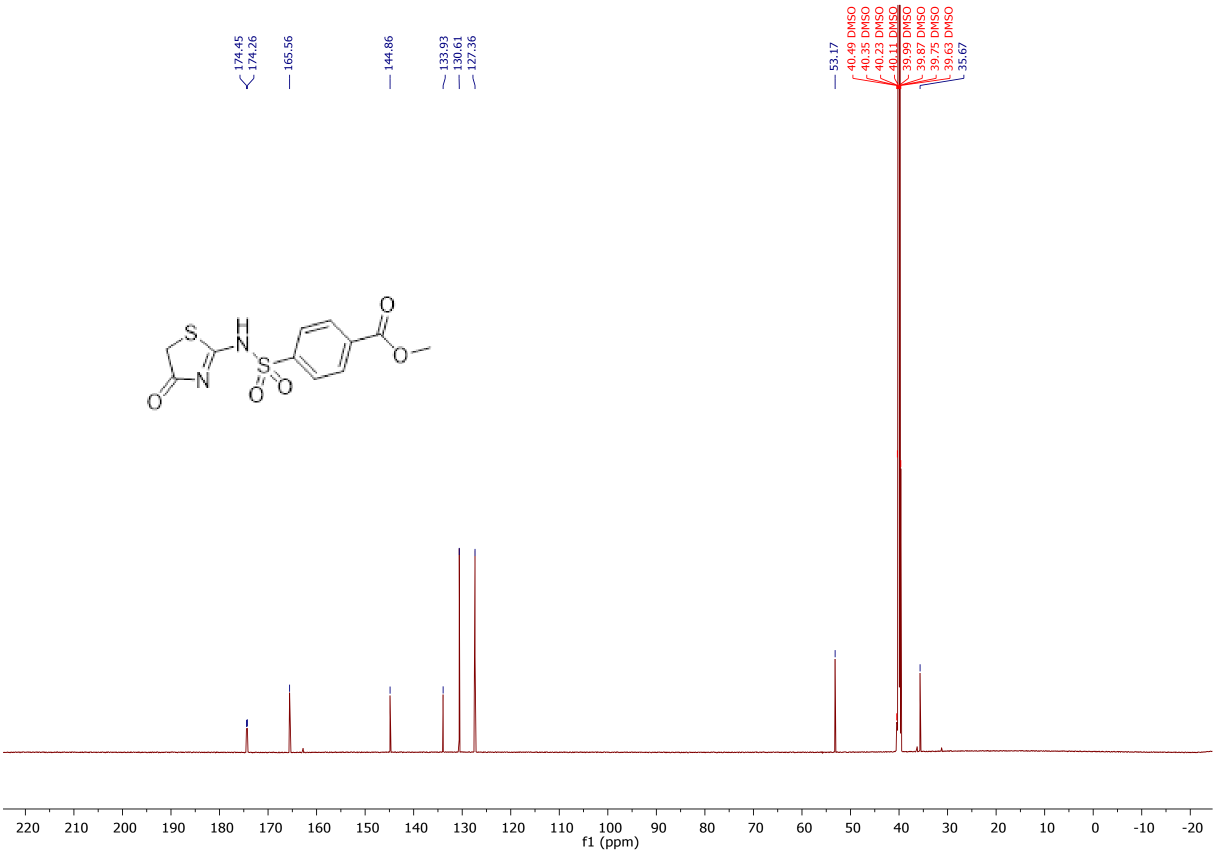

^{1}H NMR Spectrum of **Ir** (500 MHz, DMSO-d_6):

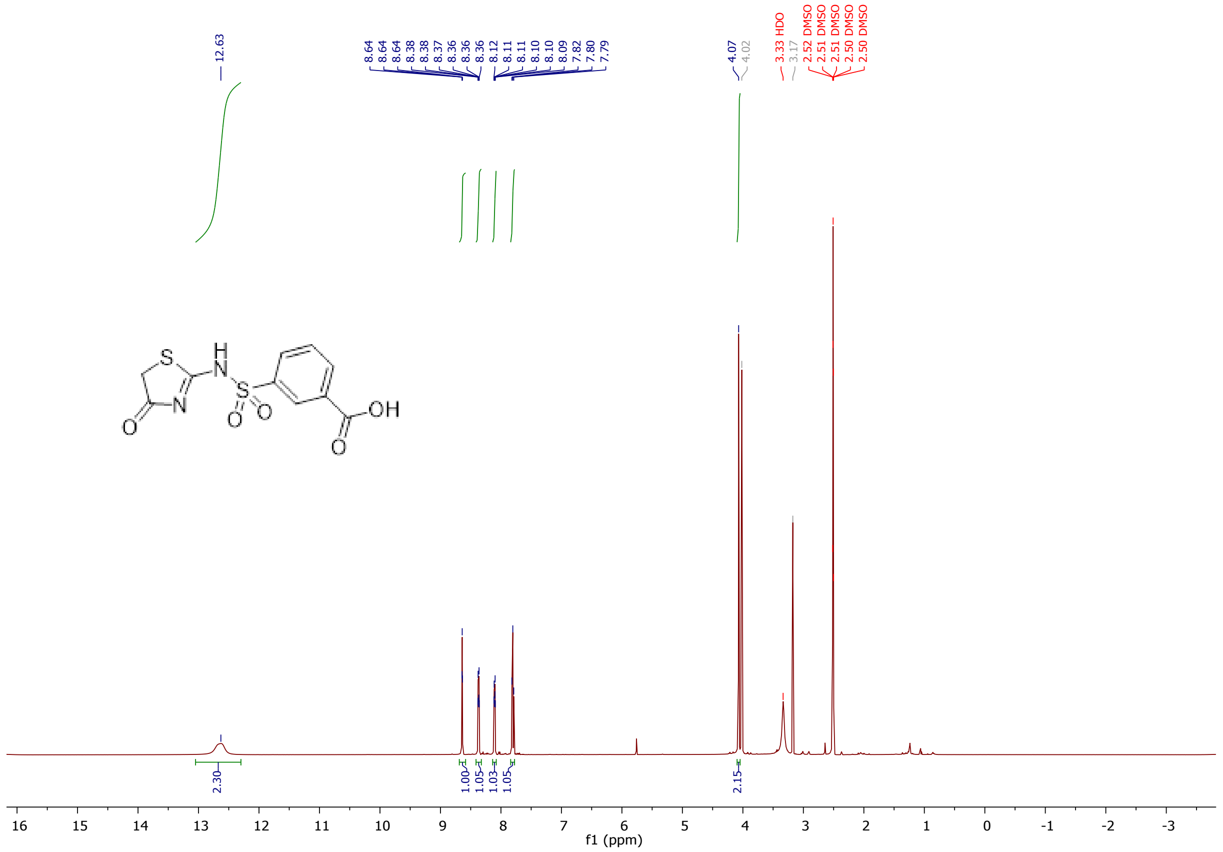

^{13}C NMR Spectrum of **Ir** (126 MHz, DMSO-d_6):

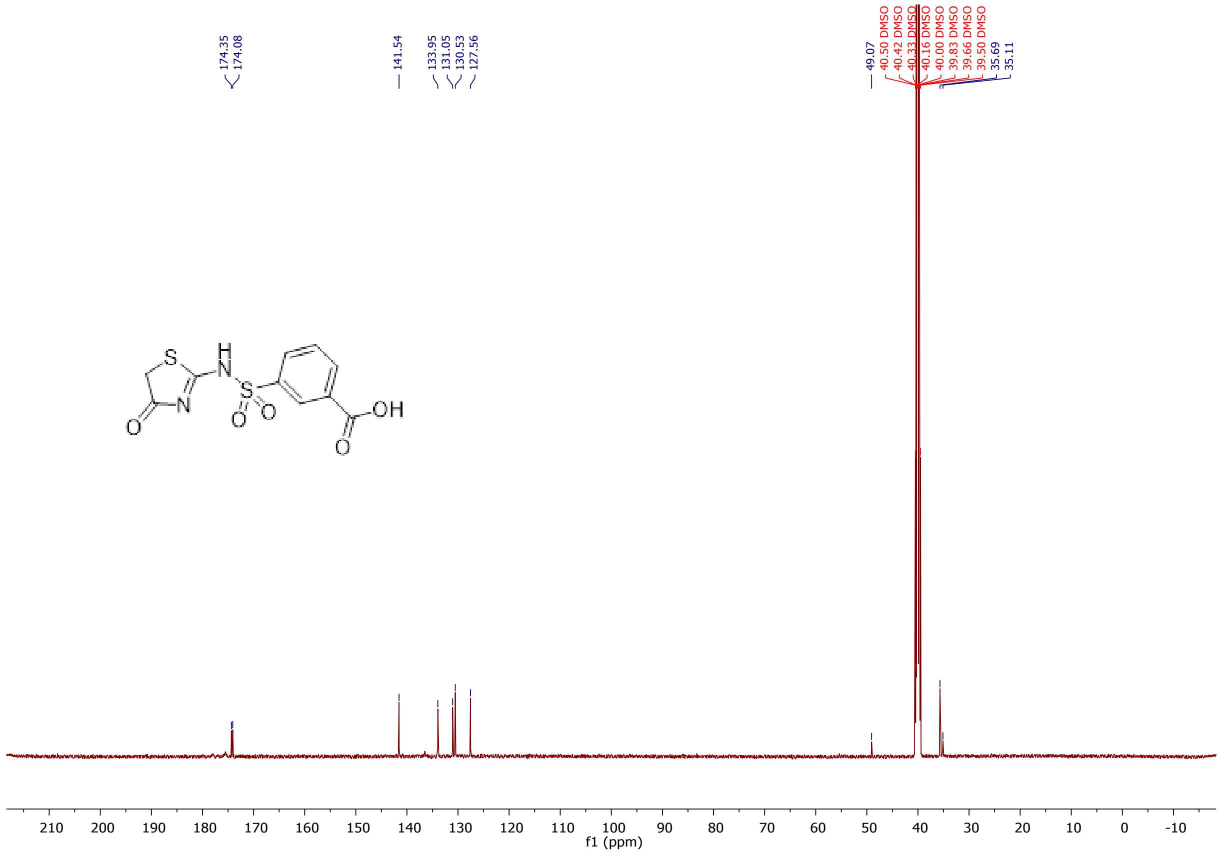

^{1}H NMR Spectrum of **Is** (600 MHz, DMSO-d_6):

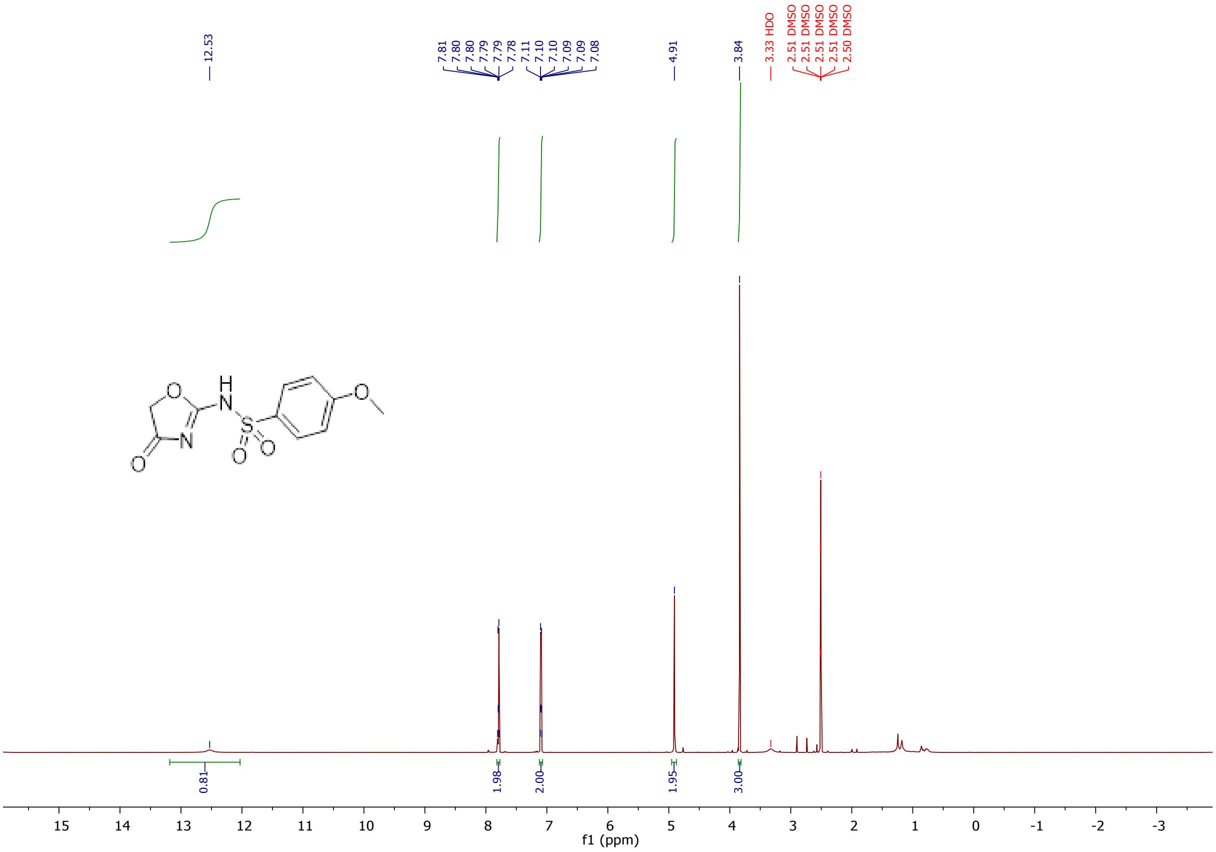

^{13}C NMR Spectrum of **Is** (151 MHz, DMSO-d_6):

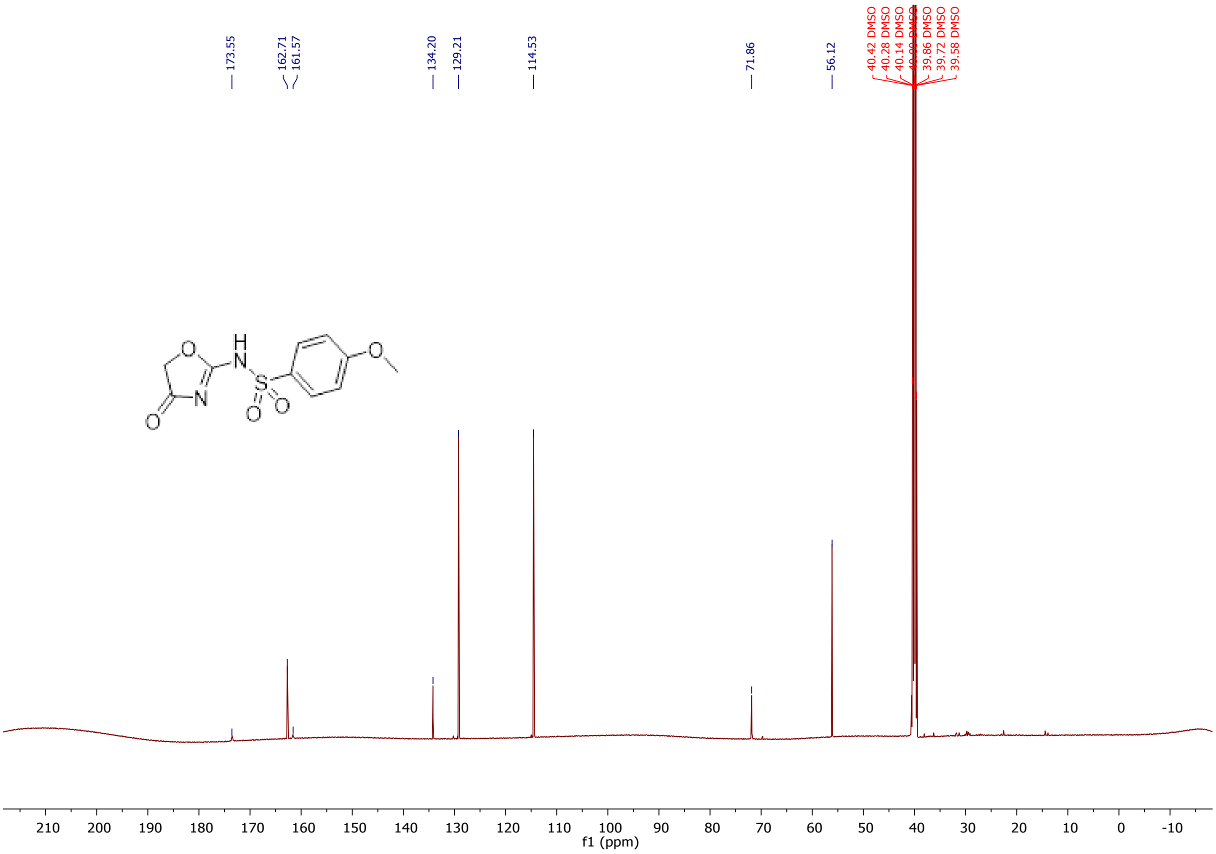

^{1}H NMR Spectrum of **IIa** (600 MHz, DMSO-d_6):

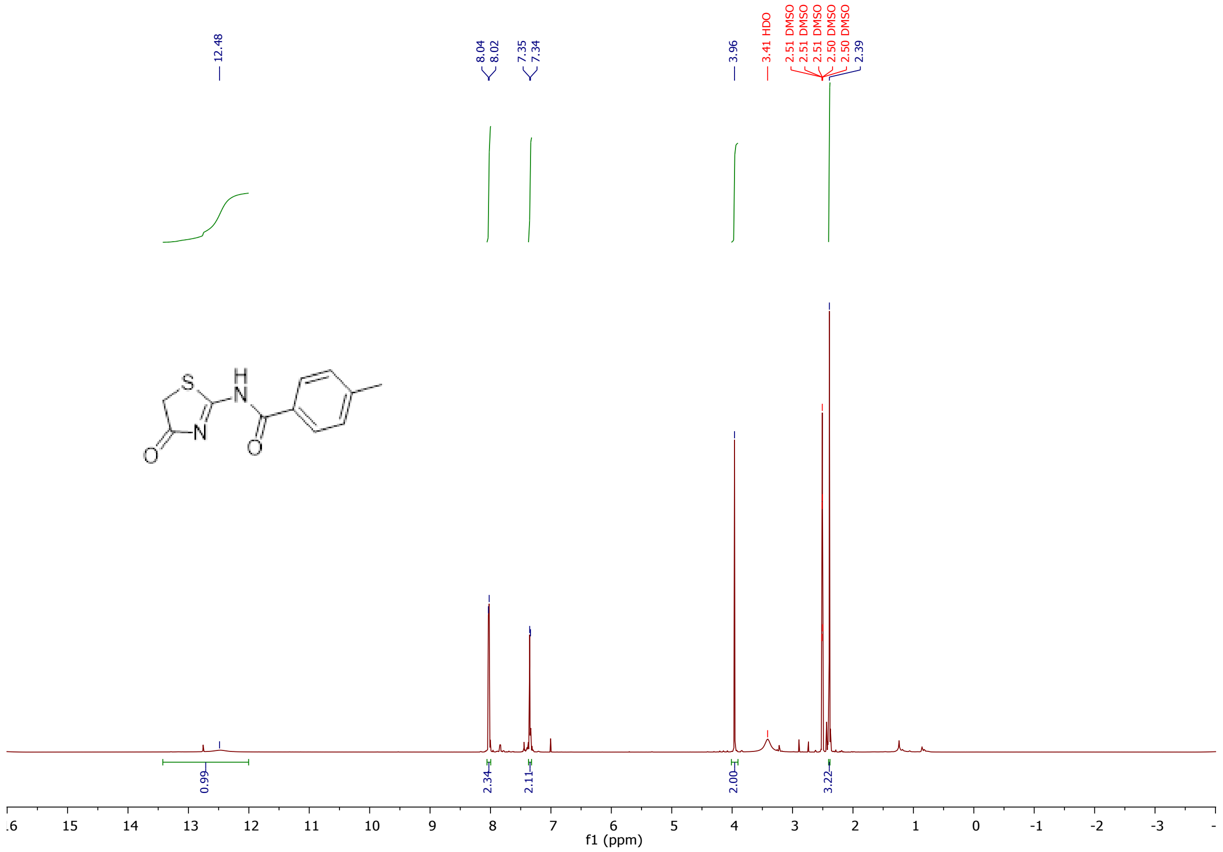

^{13}**C NMR** Spectrum of **IIa** (151 MHz, DMSO-d_6):

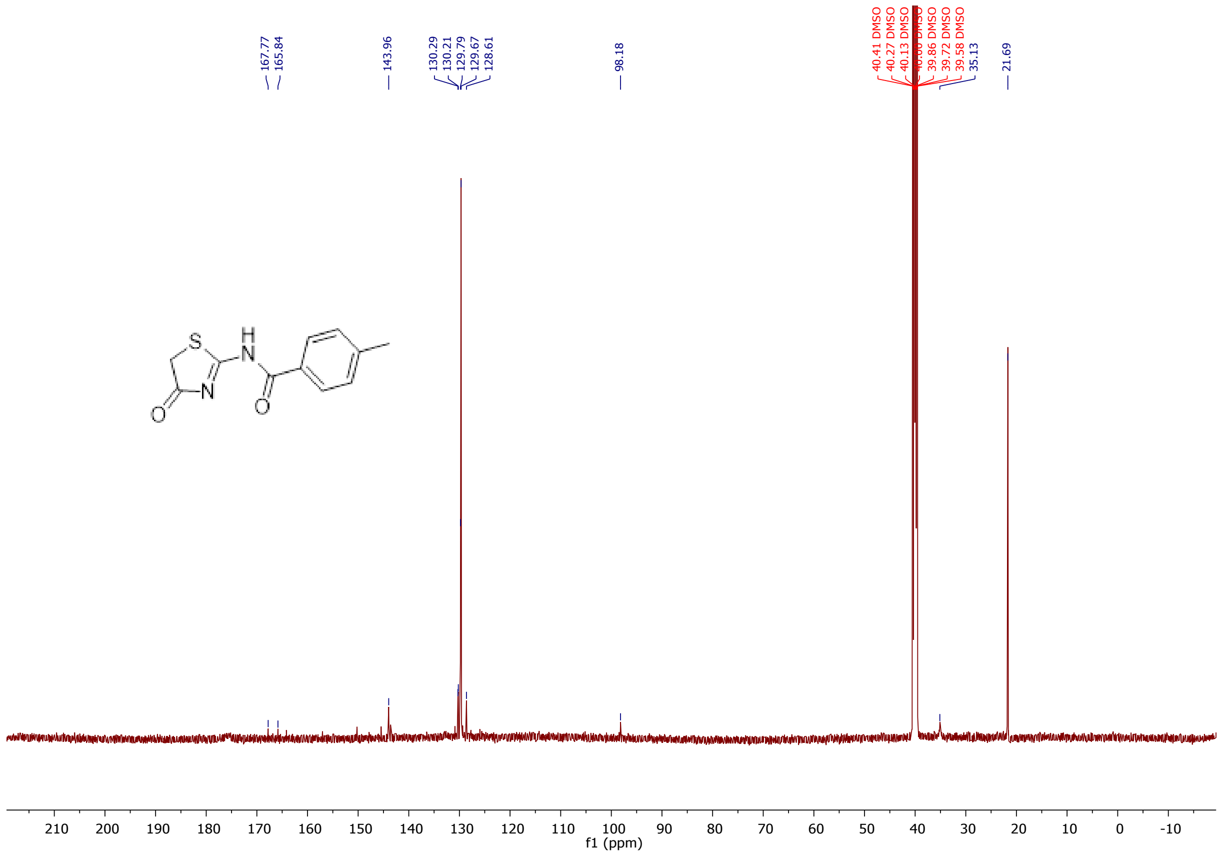

^{1}**H NMR** Spectrum of **IIb** (700 MHz, DMSO-d_6):

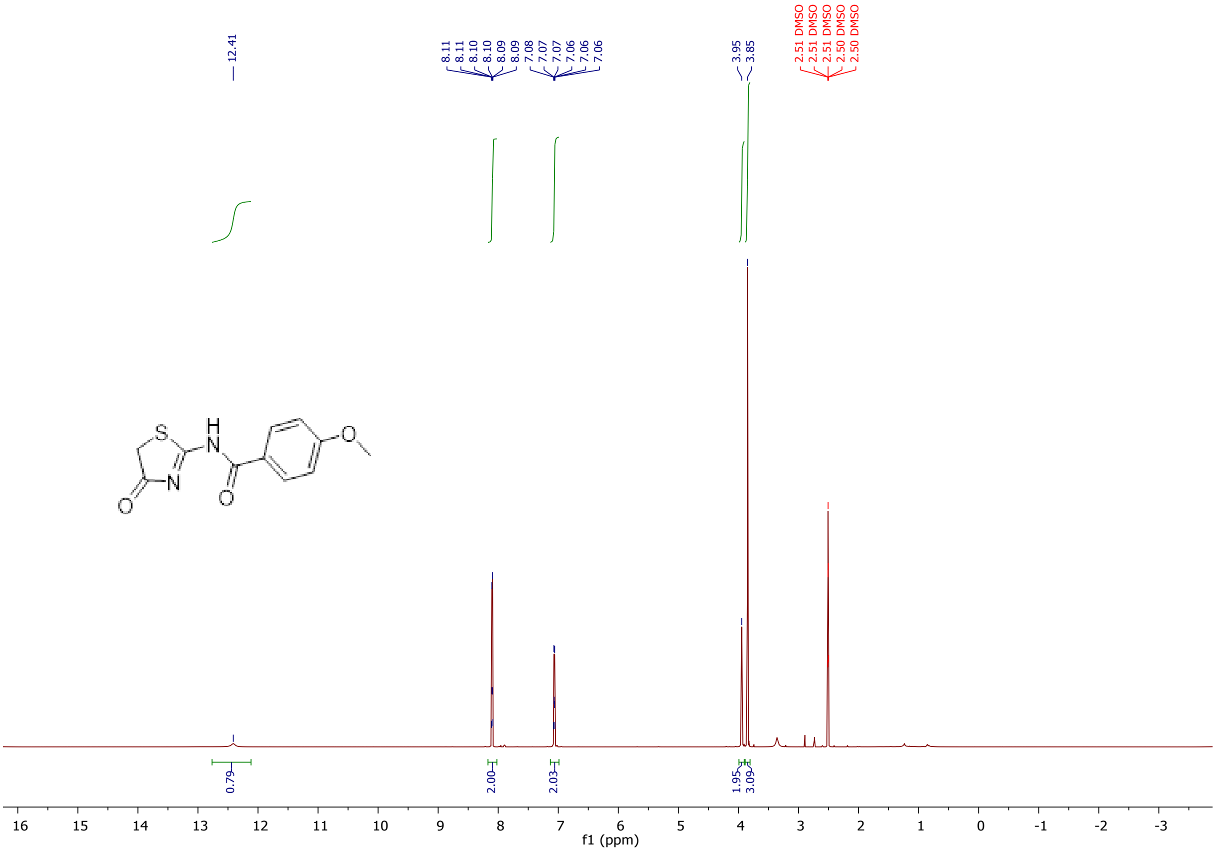

^{13}C NMR Spectrum of **IIb** (174 MHz, DMSO-d_6):

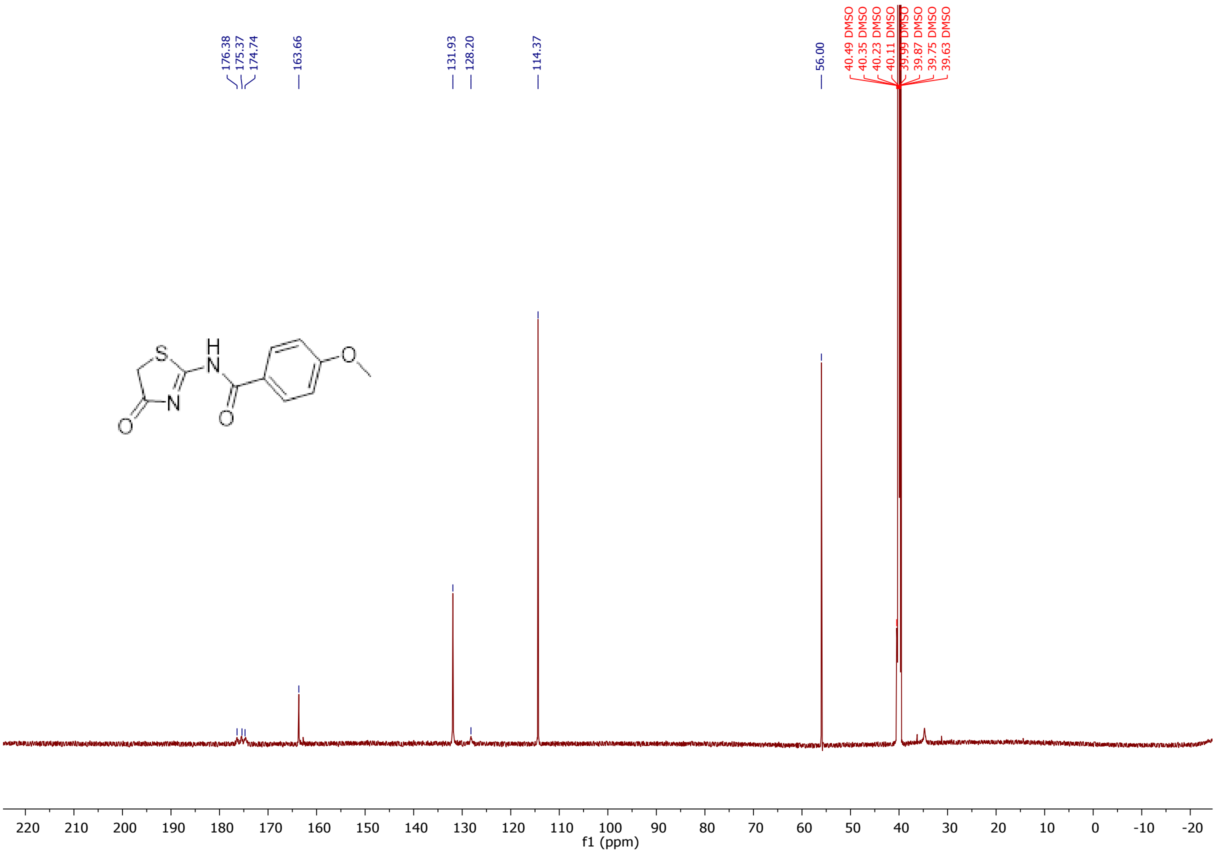

^{1}H NMR Spectrum of **IIc** (600 MHz, DMSO-d_6) (d.r. = 4:1):

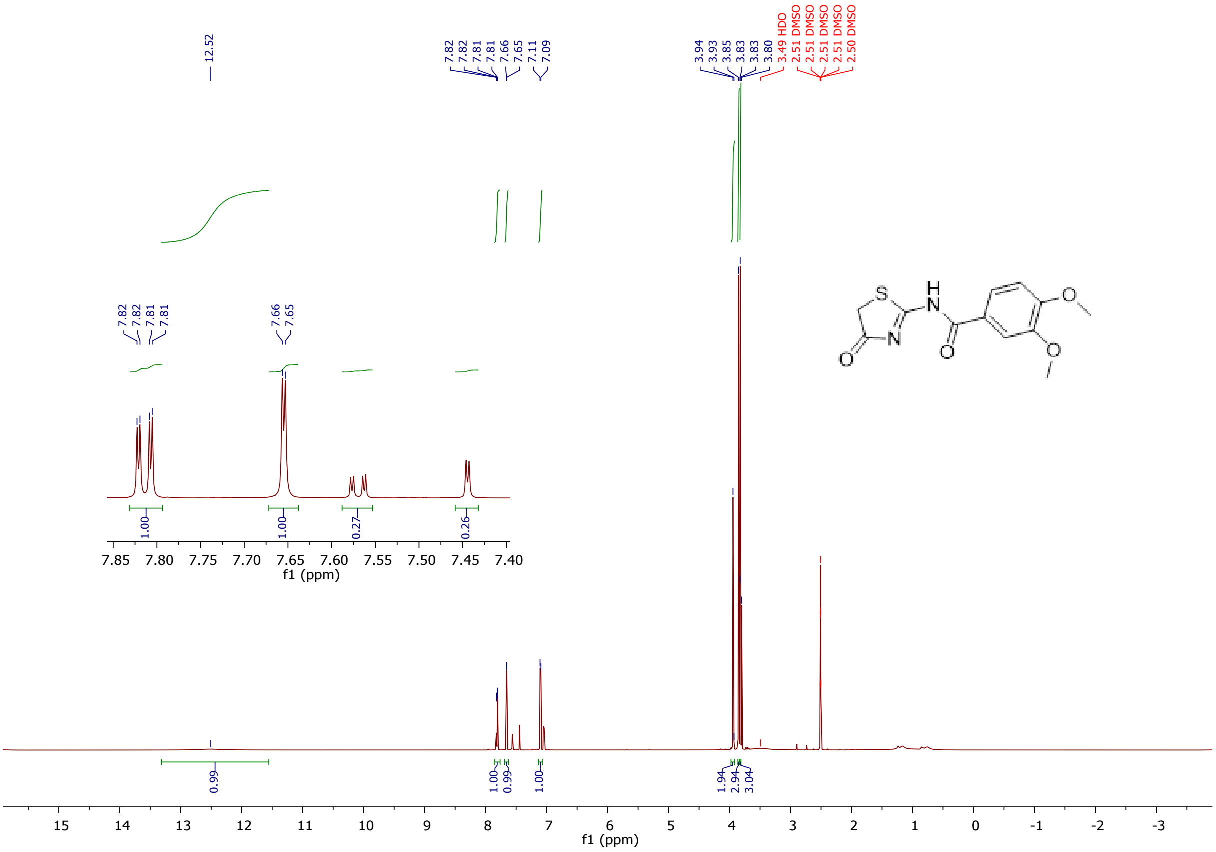

^{13}C NMR Spectrum of **IIc** (151 MHz, DMSO-d_6):

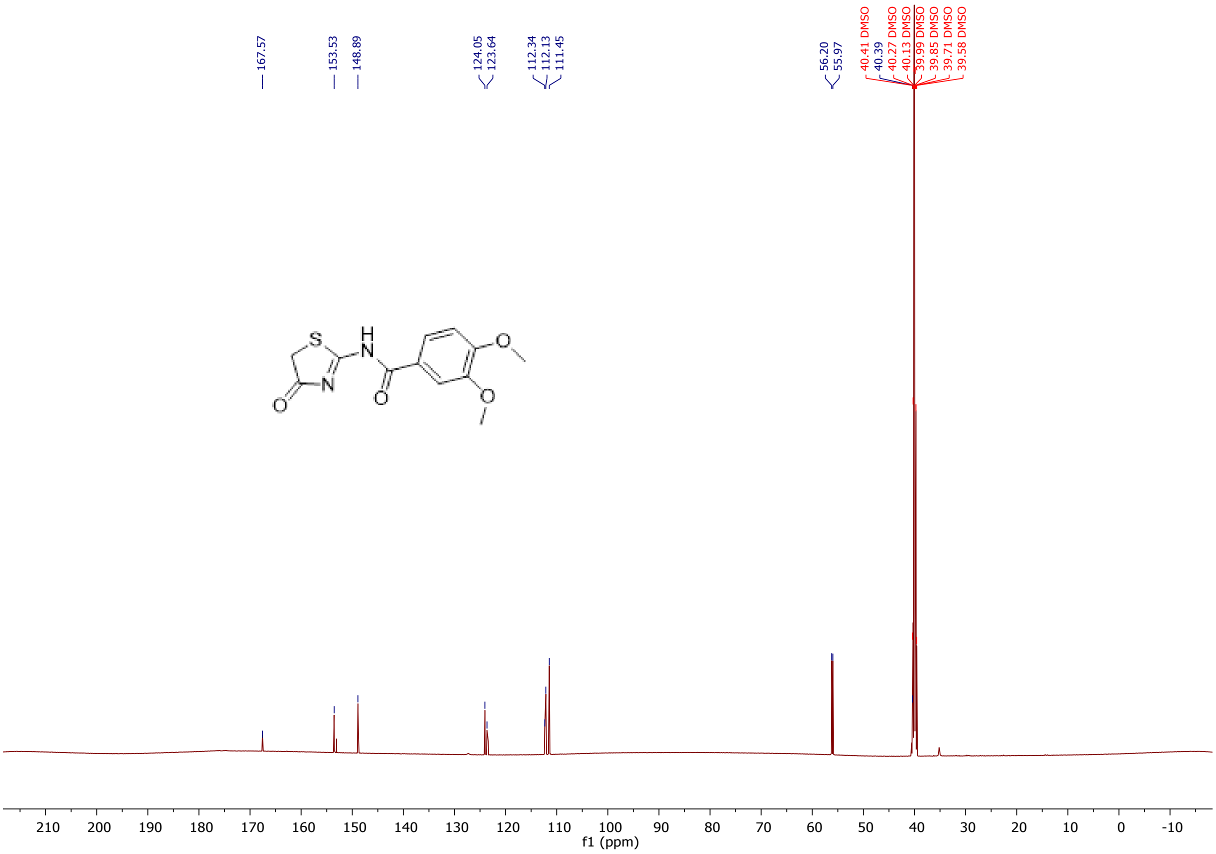

^{1}H NMR Spectrum of **IId** (600 MHz, DMSO-d_6):

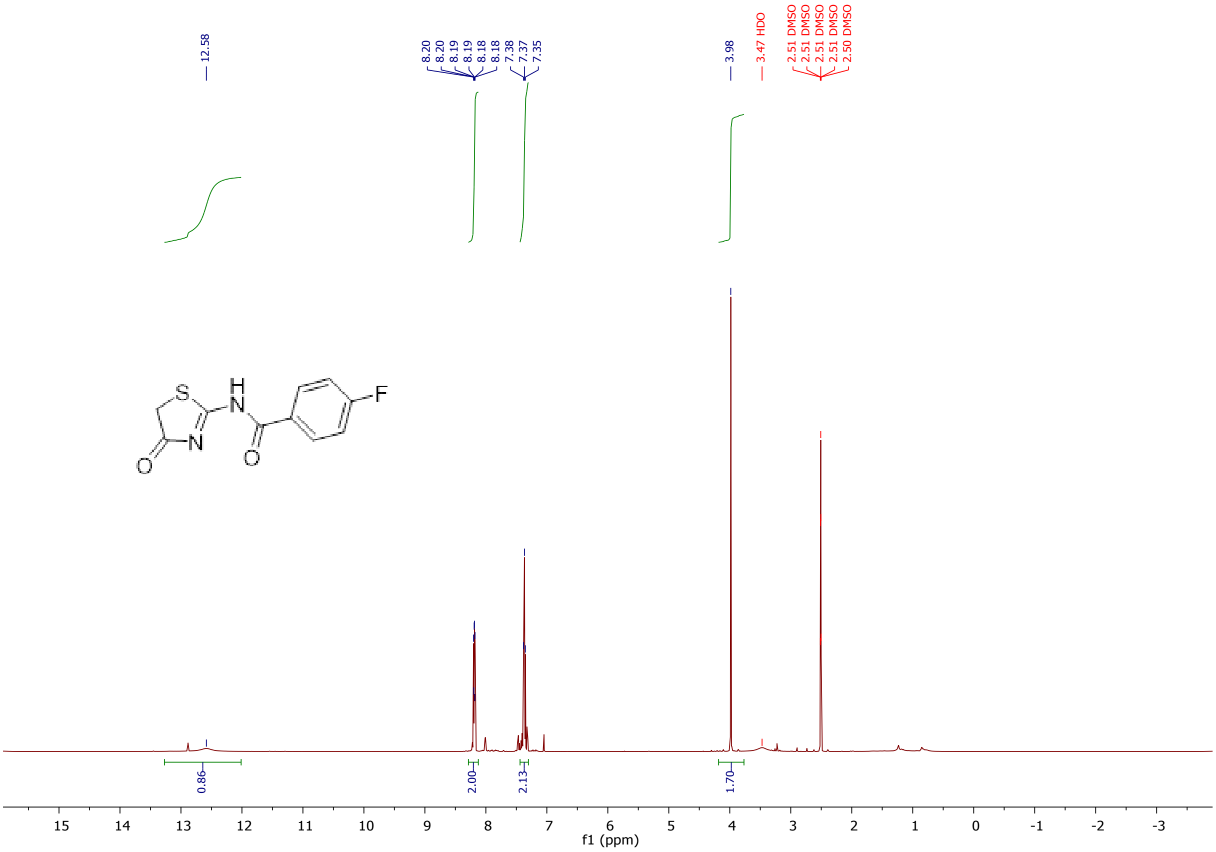

^{13}C NMR Spectrum of **IId** (151 MHz, DMSO-d_6):

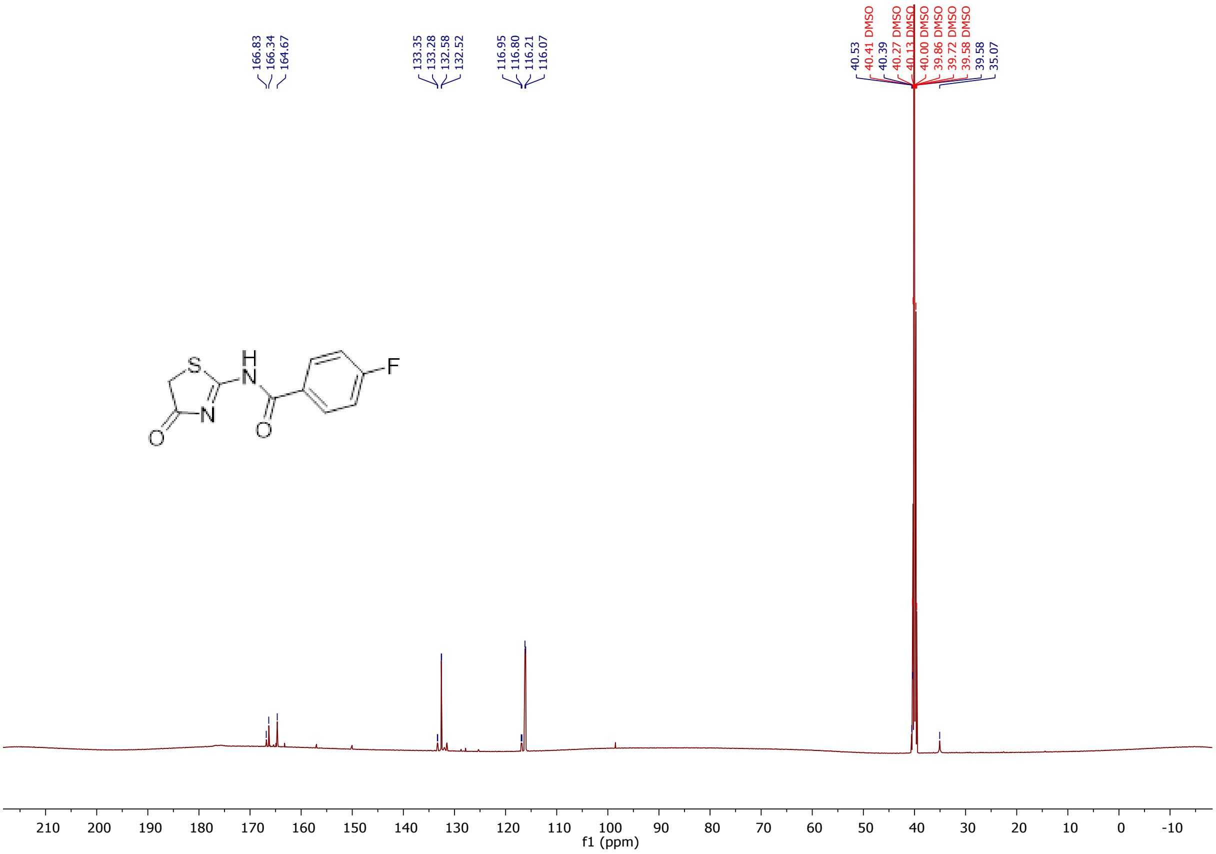

^{1}H NMR Spectrum of **III** (500 MHz, DMSO-d_6):

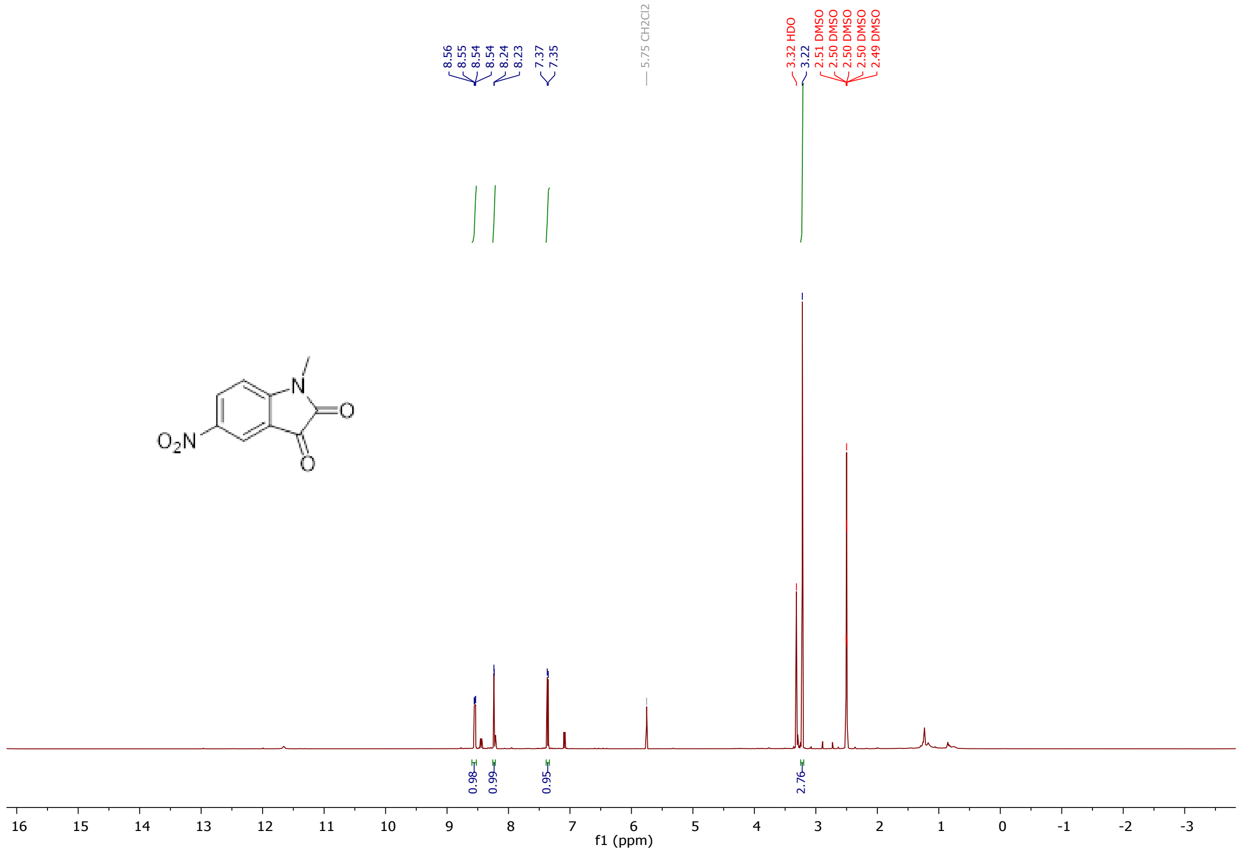

^{13}C NMR Spectrum of **III** (126 MHz, DMSO-d_6):

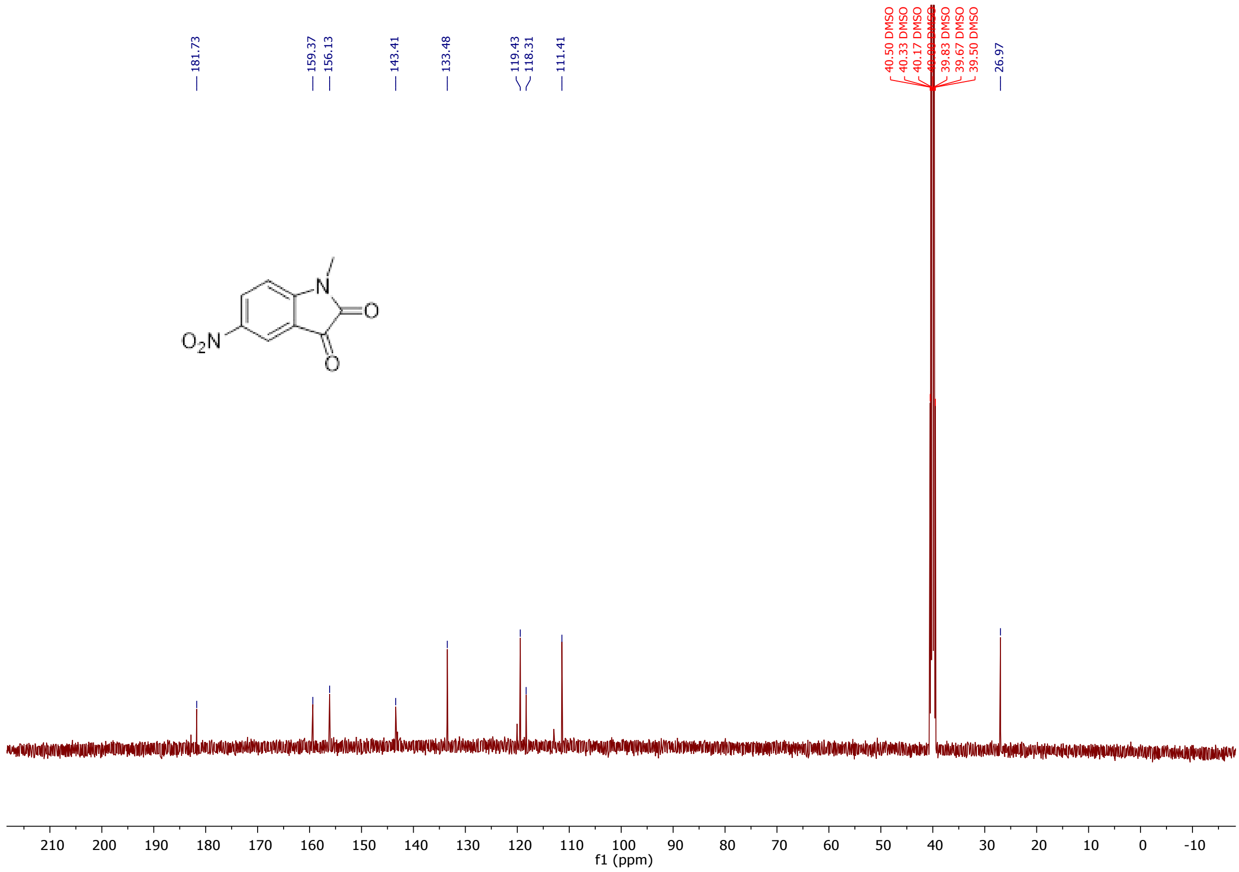

^{1}H NMR Spectrum of **VI** (600 MHz, DMSO-d_6):

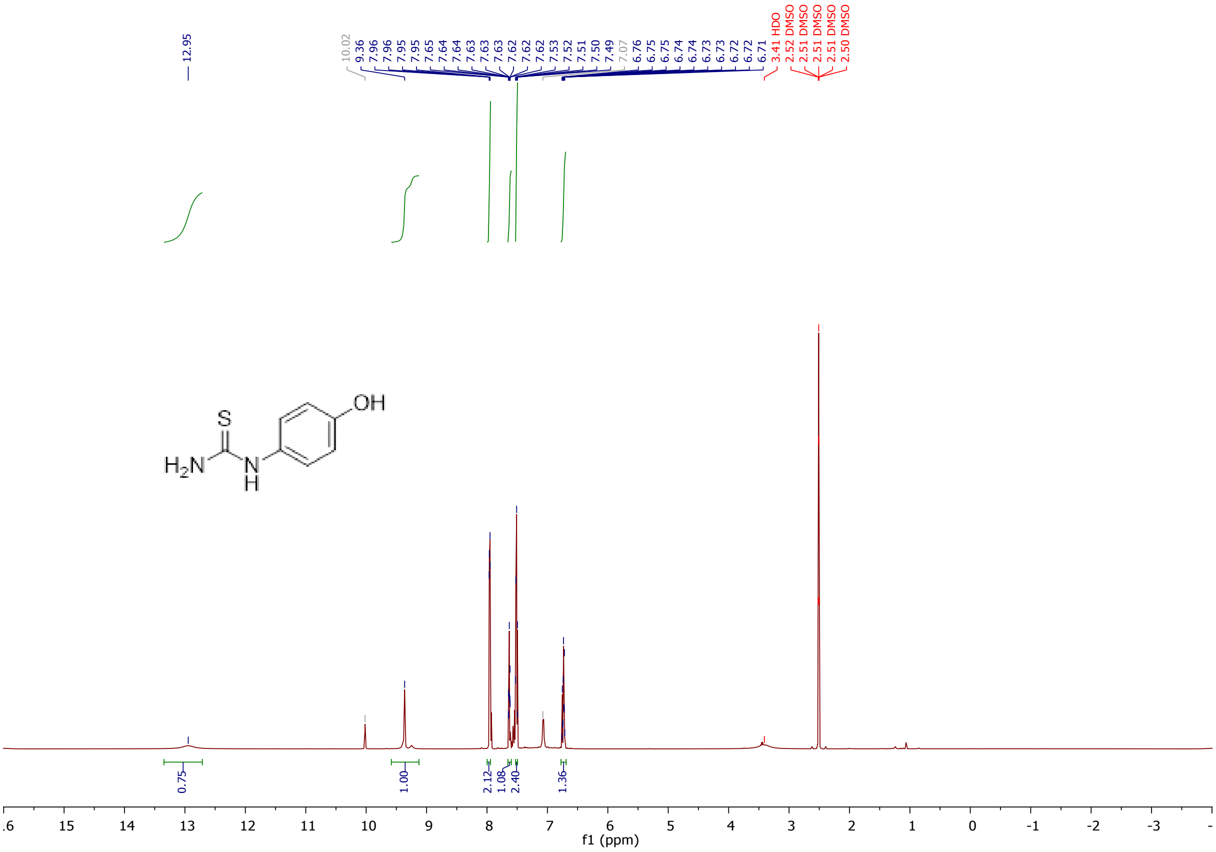

^{13}C NMR Spectrum of **VI** (151 MHz, DMSO-d_6):

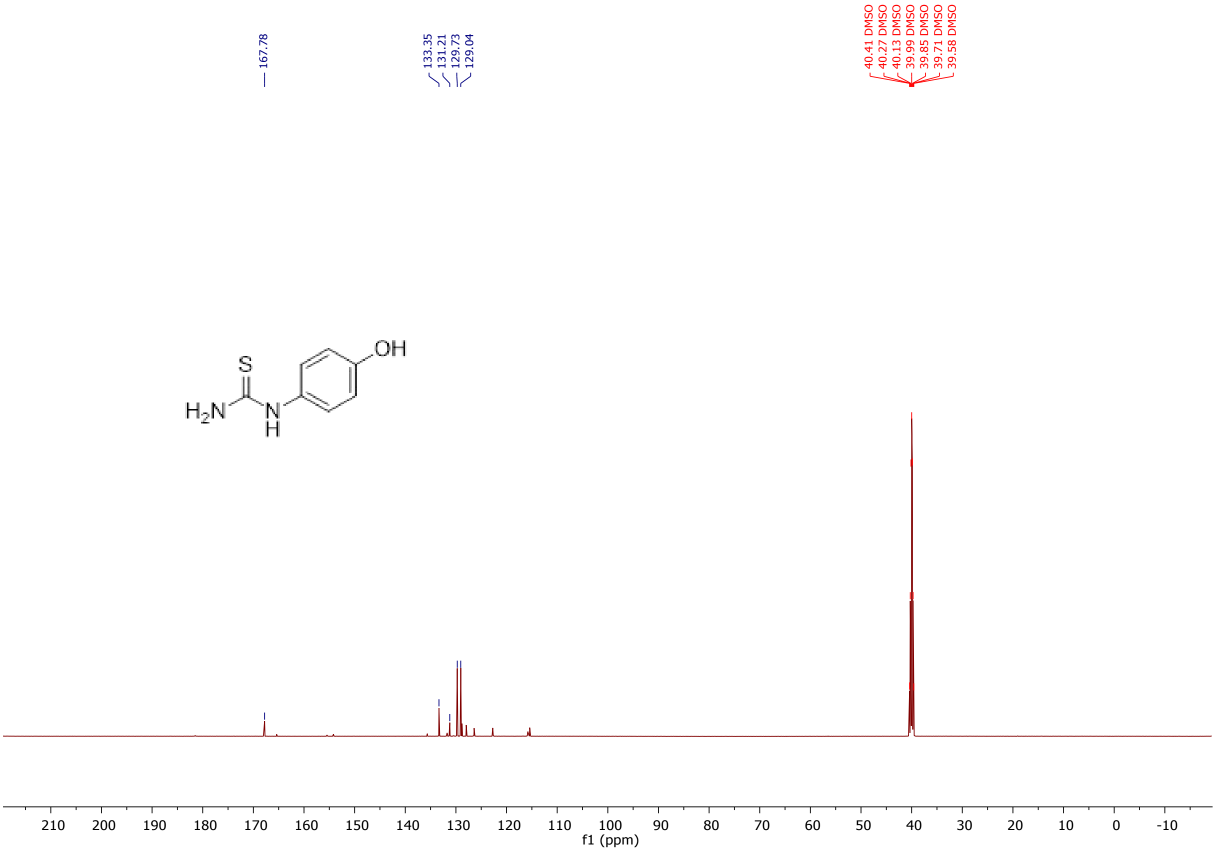

^{1}H NMR Spectrum of **1** (700 MHz, DMSO-d_6):

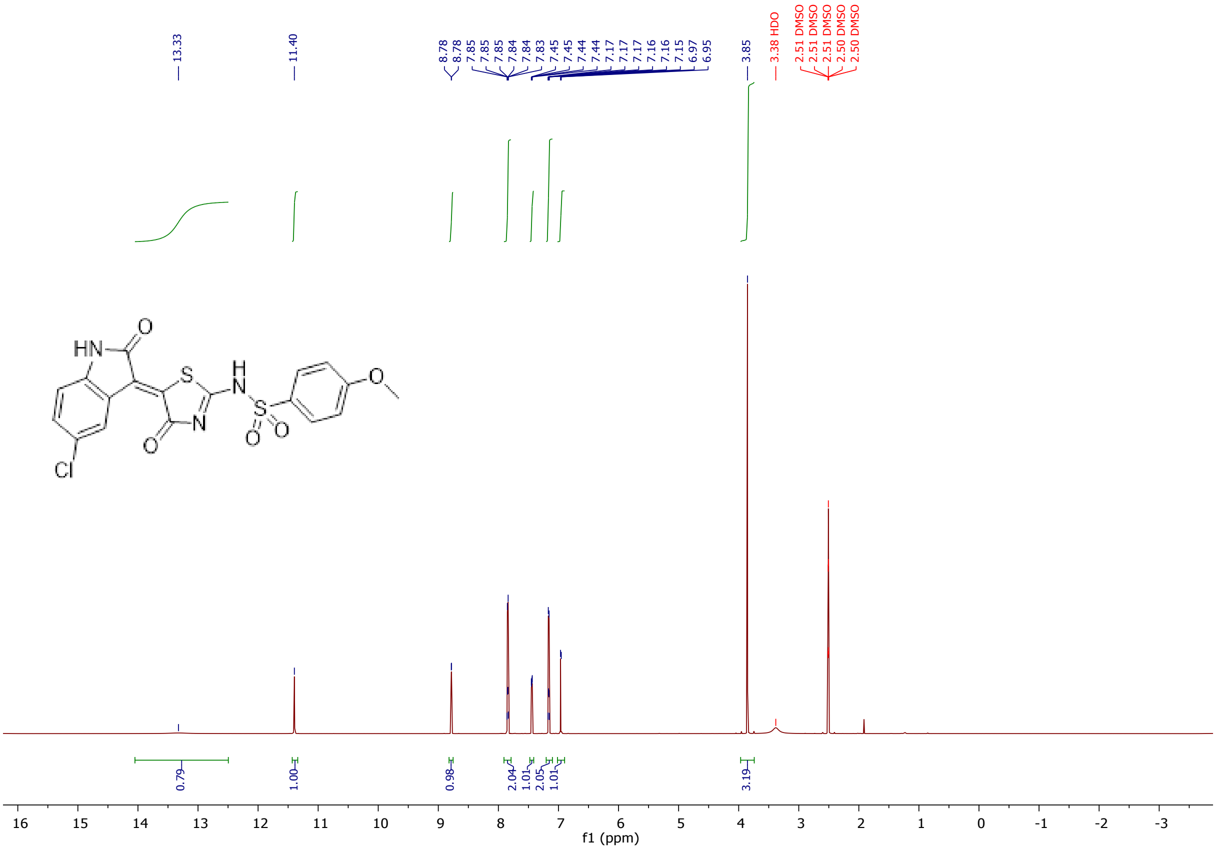

^{13}C NMR Spectrum of **1** (174 MHz, DMSO-d_6):

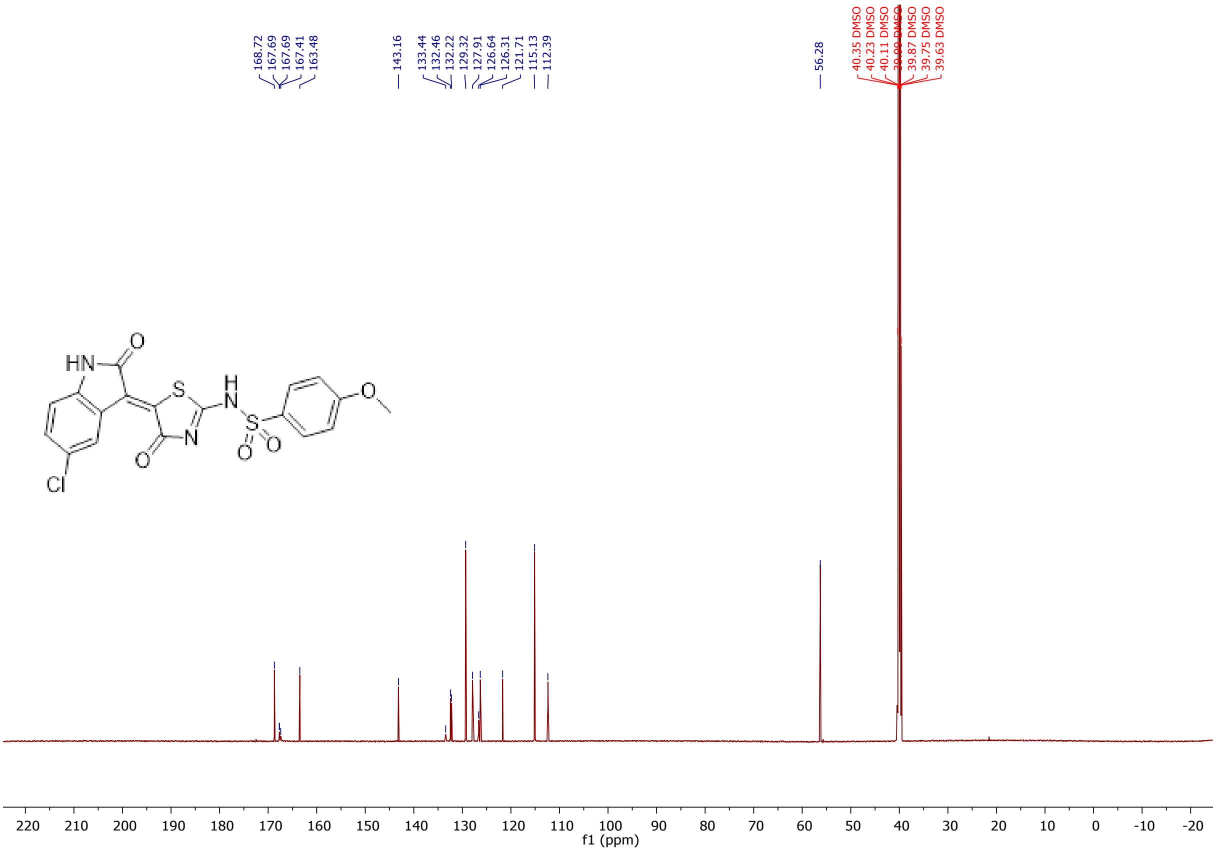

^{1}H NMR Spectrum of **2** (600 MHz, DMSO-d_6):

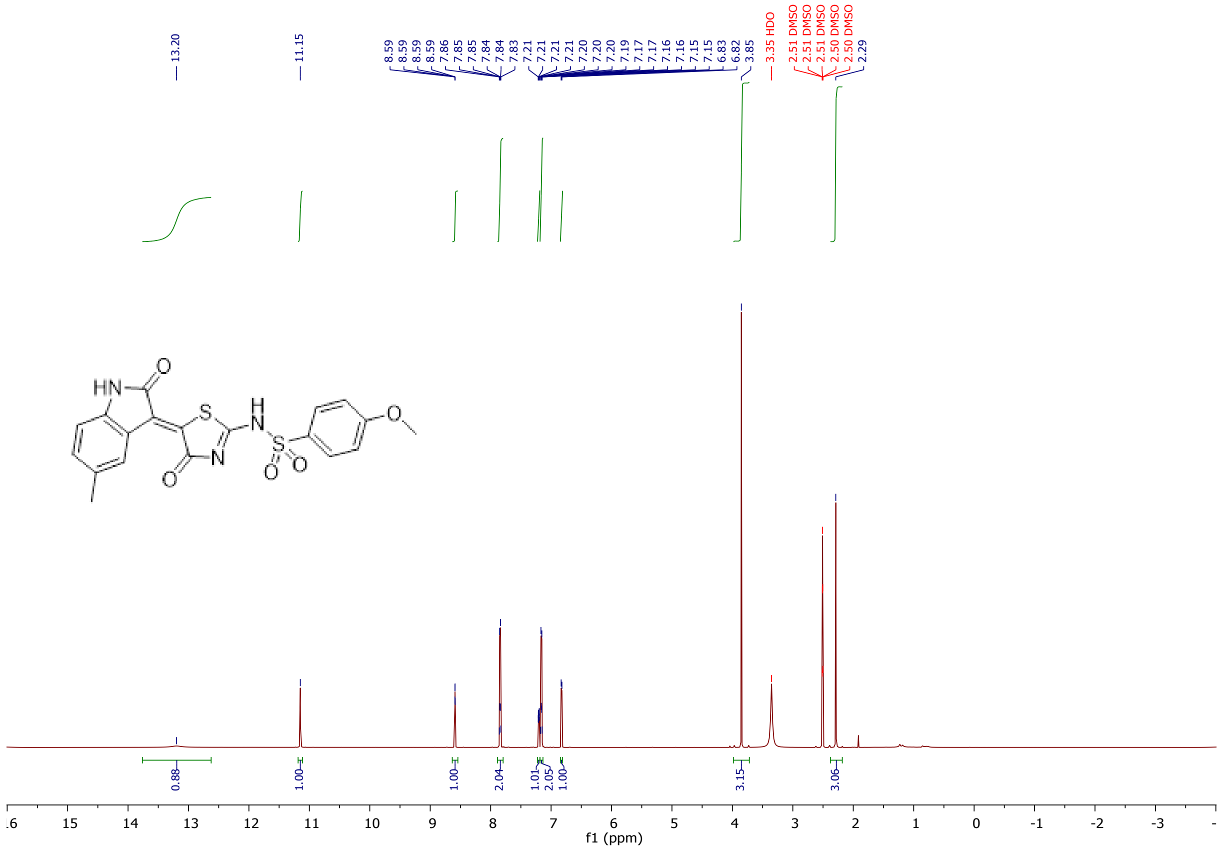

^{13}C NMR Spectrum of **2** (151 MHz, DMSO-d_6):

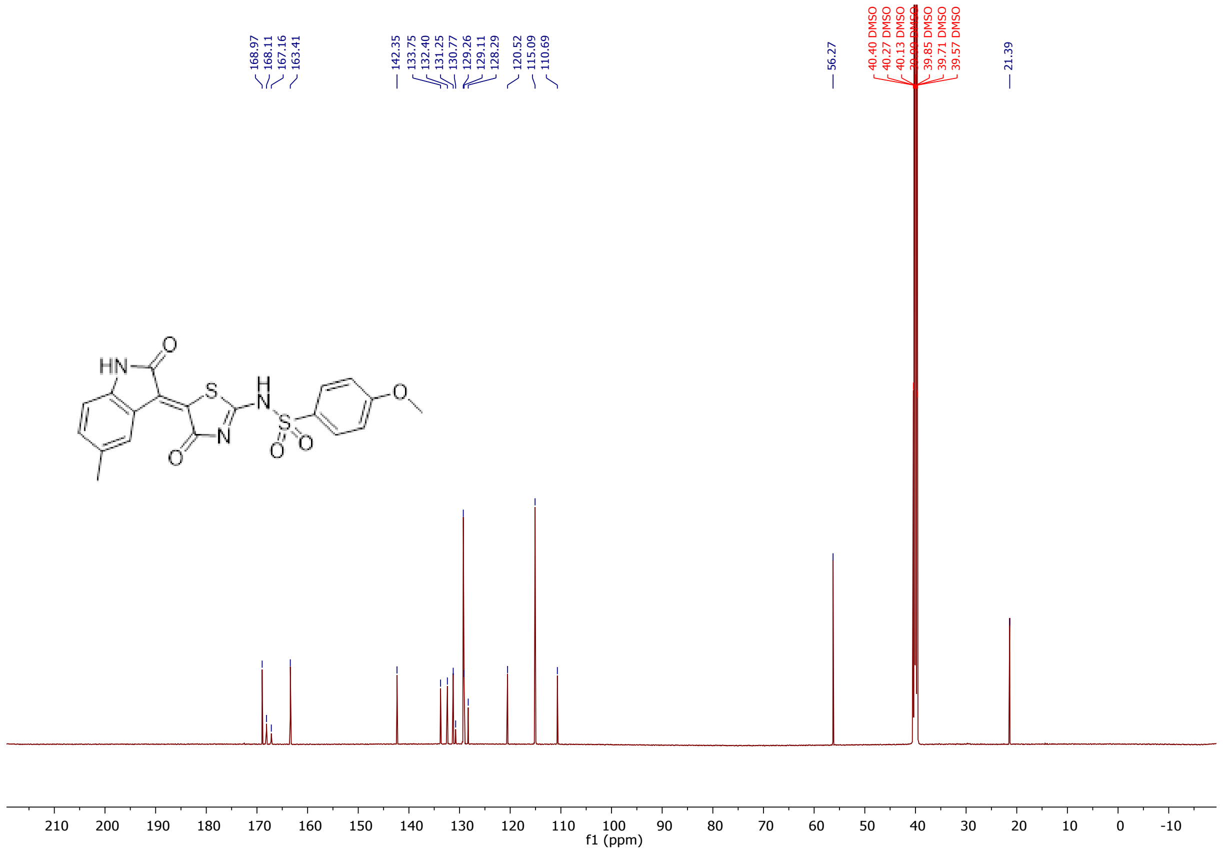

^{1}H NMR Spectrum of **3** (600 MHz, DMSO-d_6):

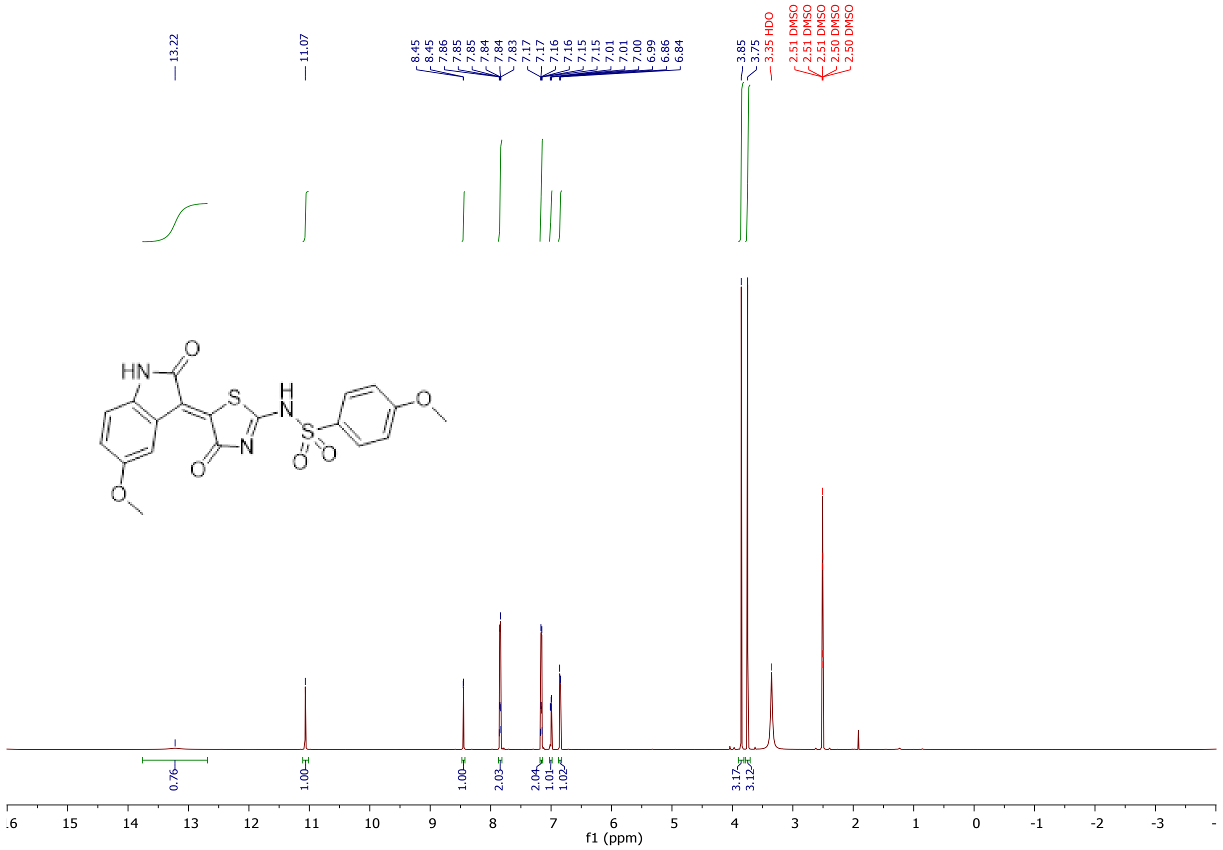

^{13}C NMR Spectrum of **3** (151 MHz, DMSO-d_6):

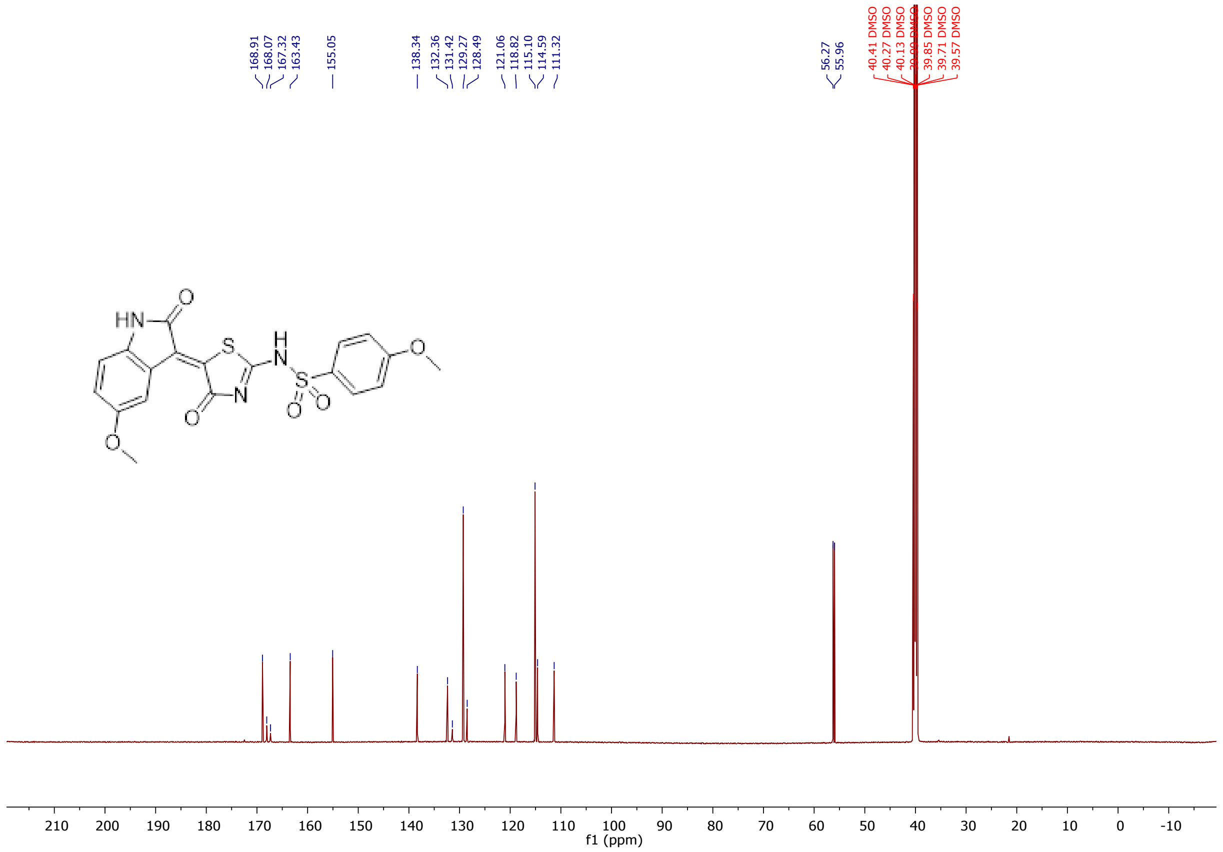

^{1}H NMR Spectrum of **4** (600 MHz, DMSO-d_6):

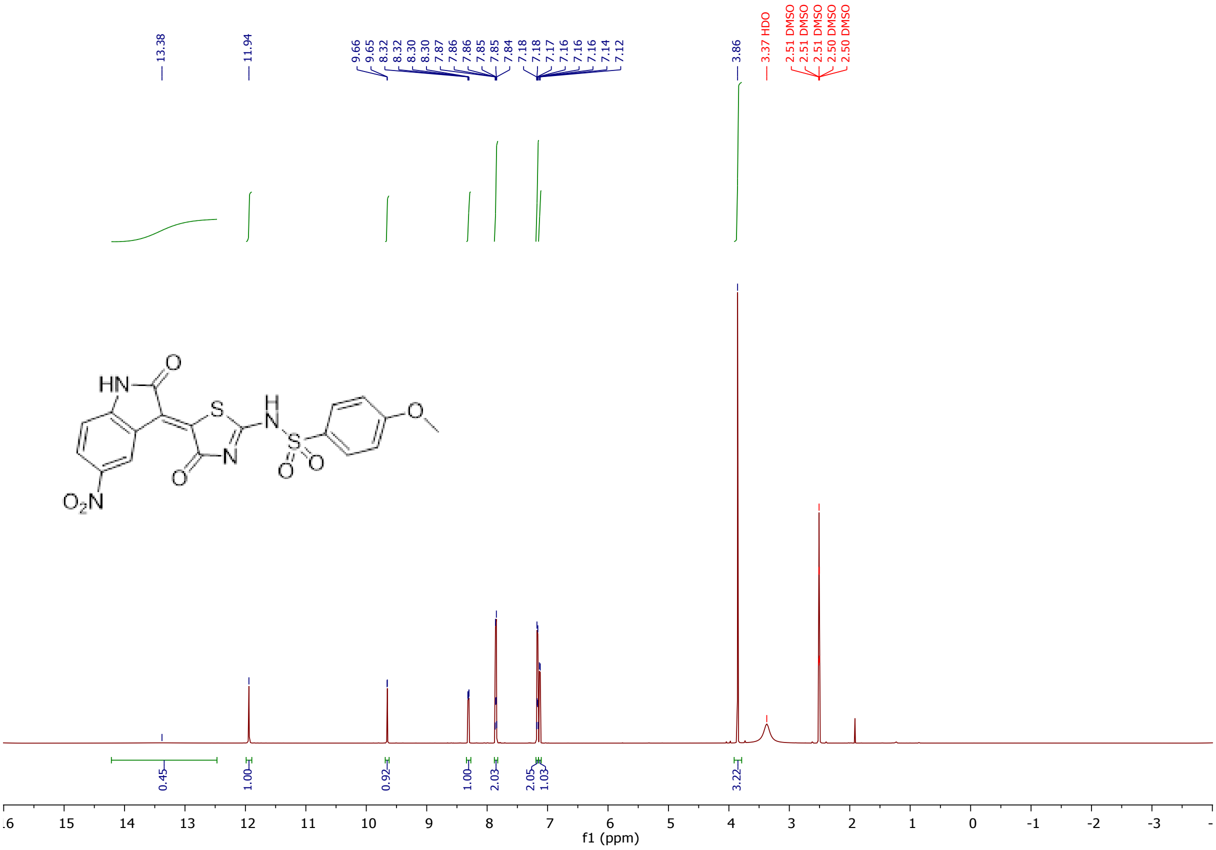

^{13}**C NMR** Spectrum of **4** (151 MHz, DMSO-d_6):

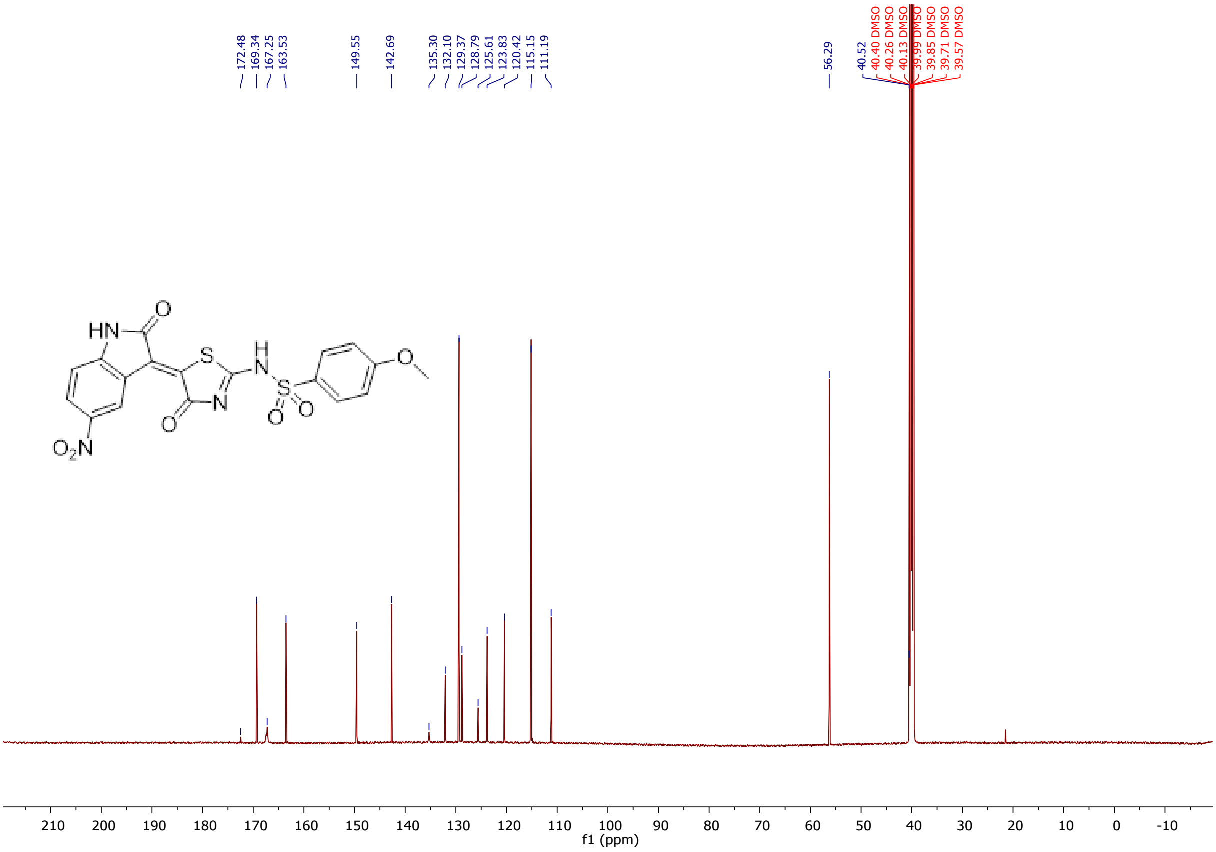

^{1}**H NMR** Spectrum of **5** (600 MHz, DMSO-d_6):

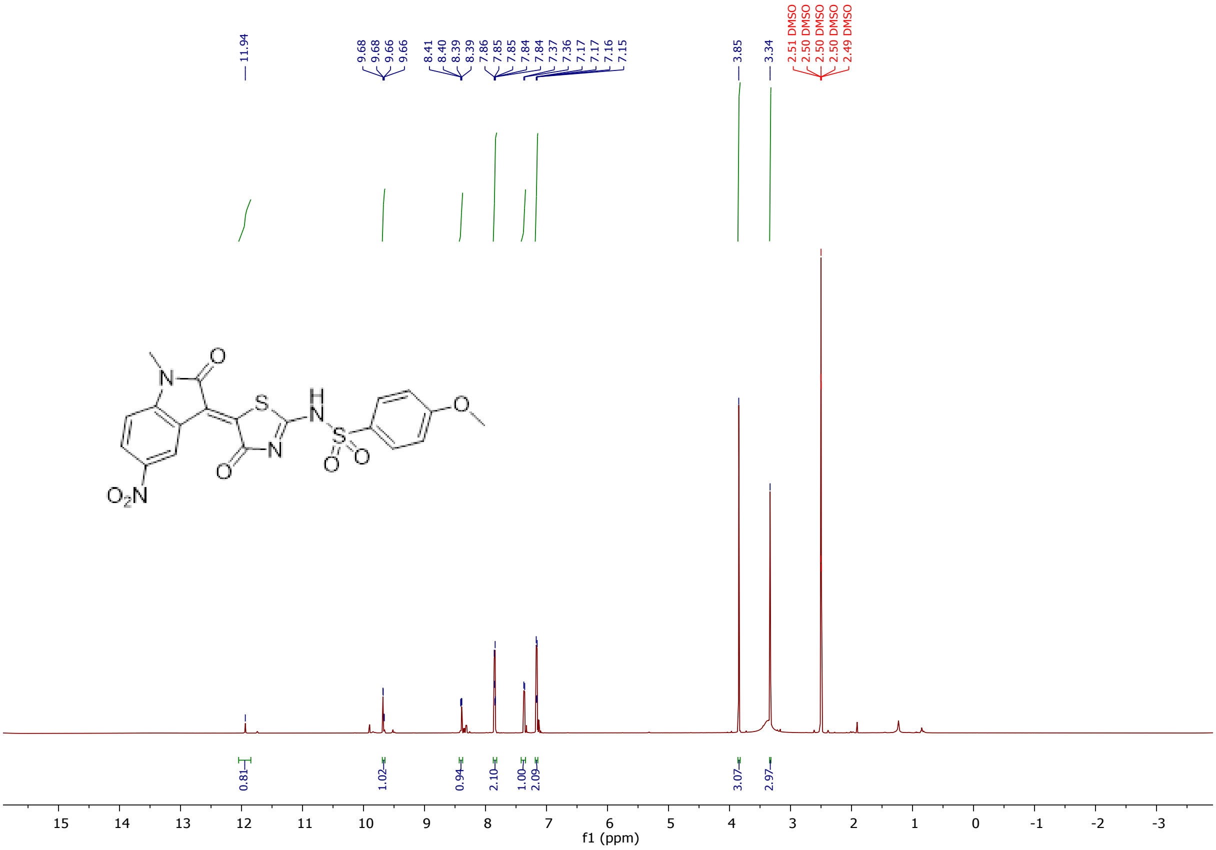

^{13}C NMR Spectrum of **5** (151 MHz, DMSO-d_6):

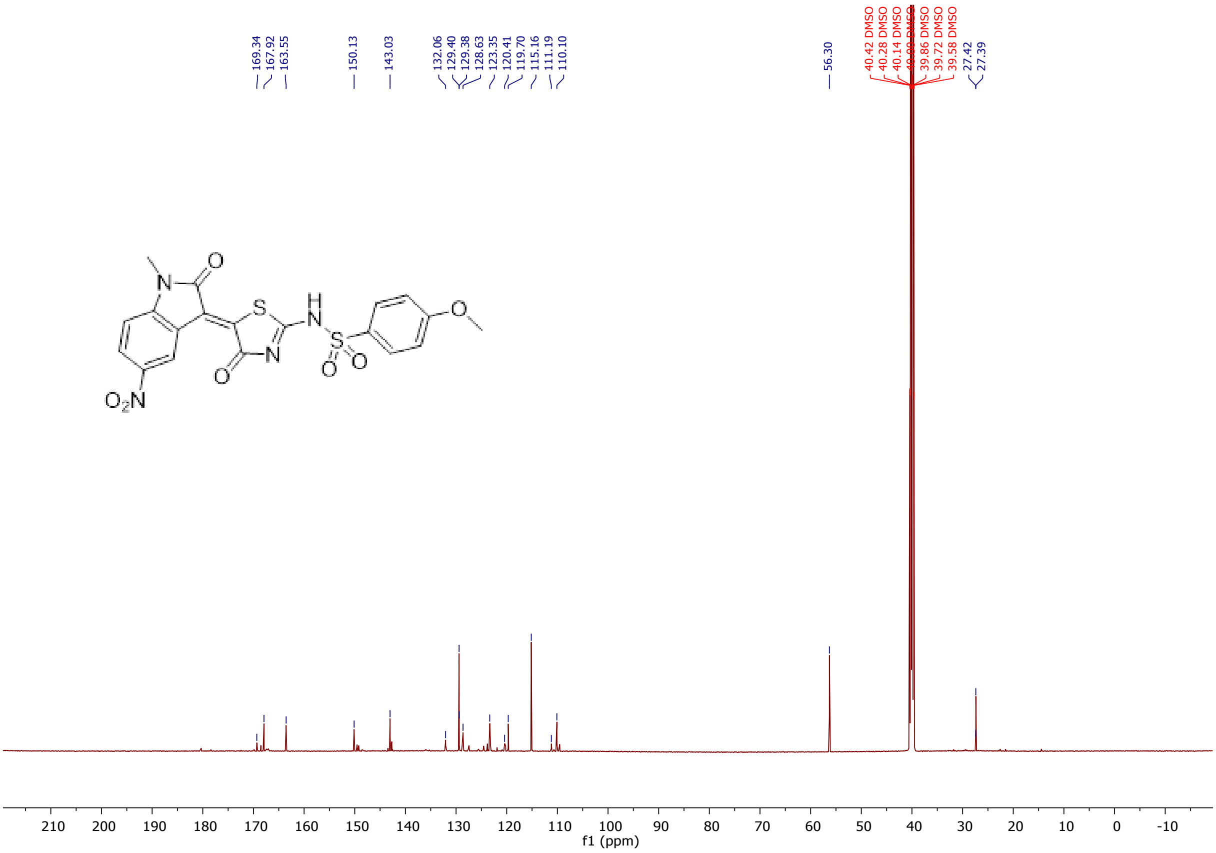

^{1}H NMR Spectrum of **6** (600 MHz, DMSO-d_6):

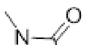

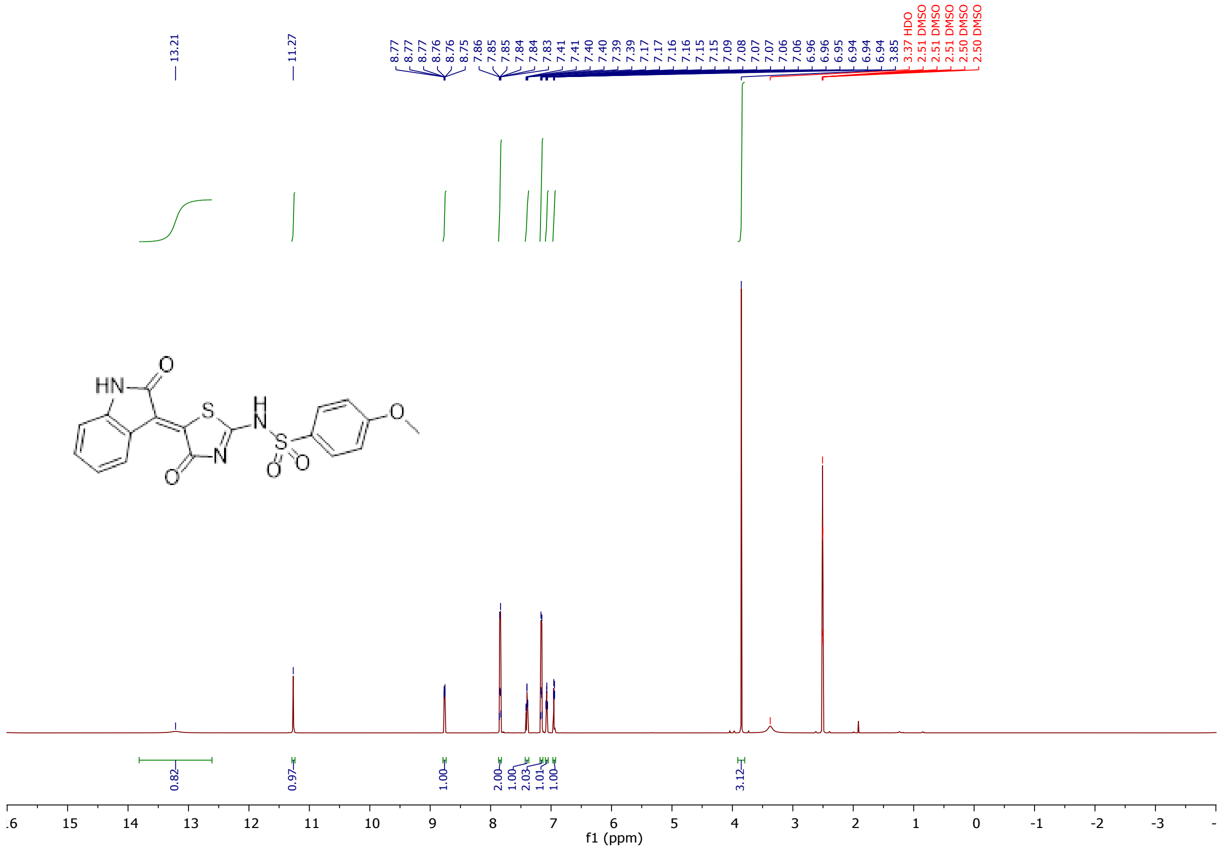

^{13}C NMR Spectrum of **6** (151 MHz, DMSO-d_6):

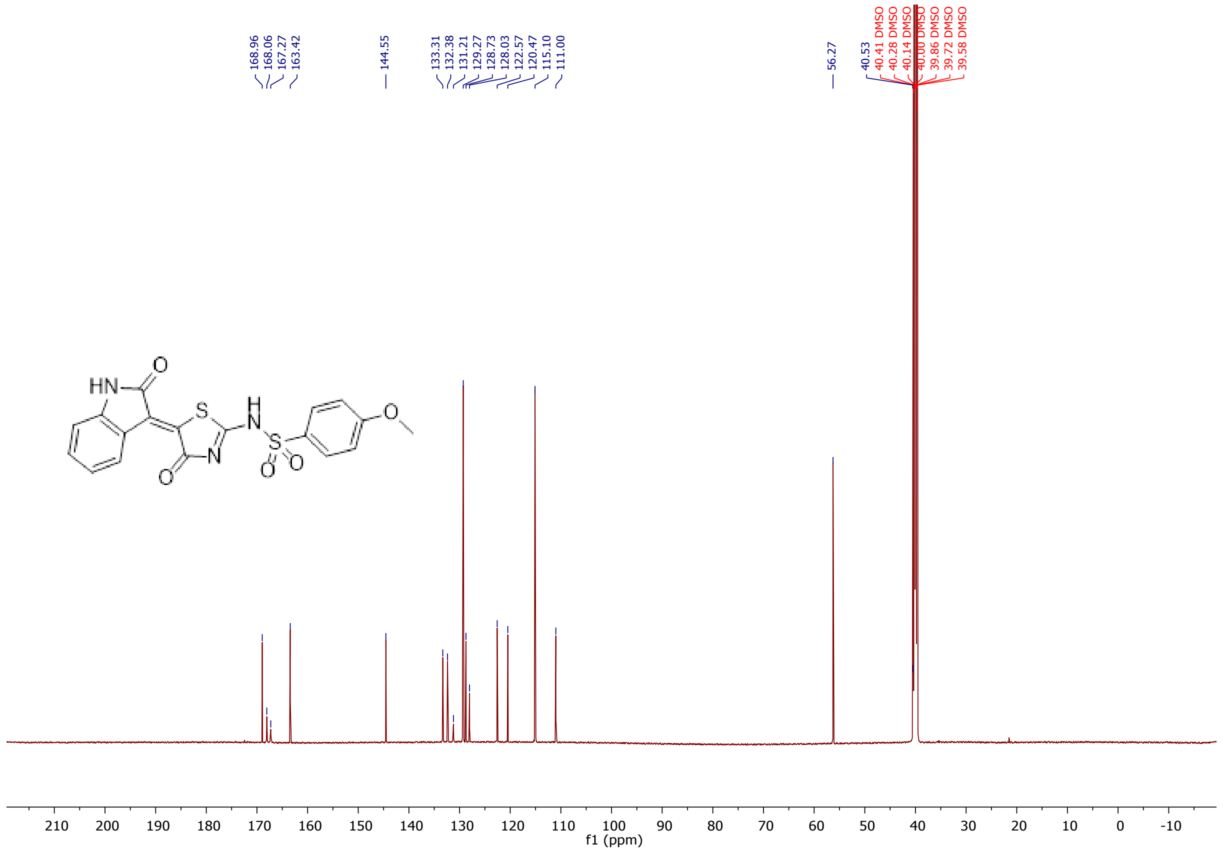

^{1}H NMR Spectrum of **7** (600 MHz, DMSO-d_6):

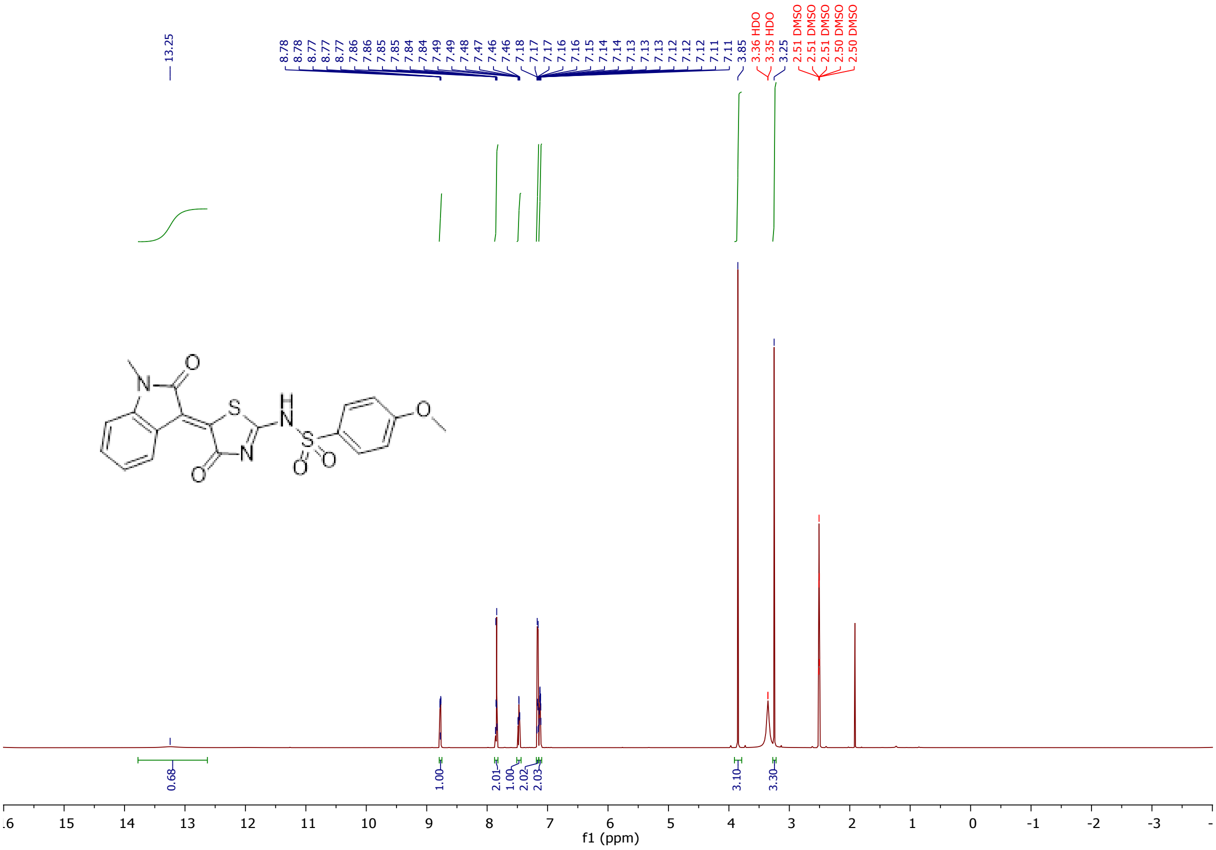

^{13}C NMR Spectrum of **7** (151 MHz, DMSO-d_6):

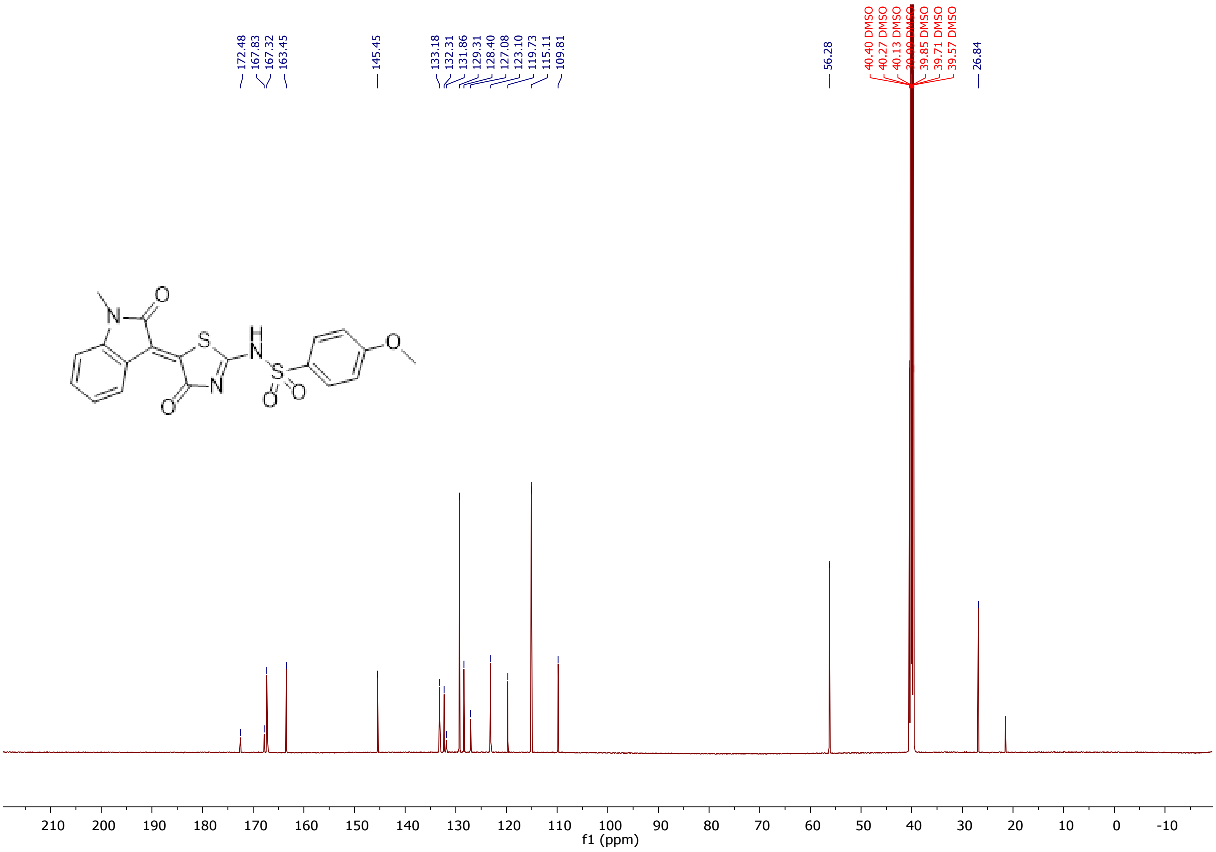

^{1}H NMR Spectrum of **8** (600 MHz, DMSO-d_6):

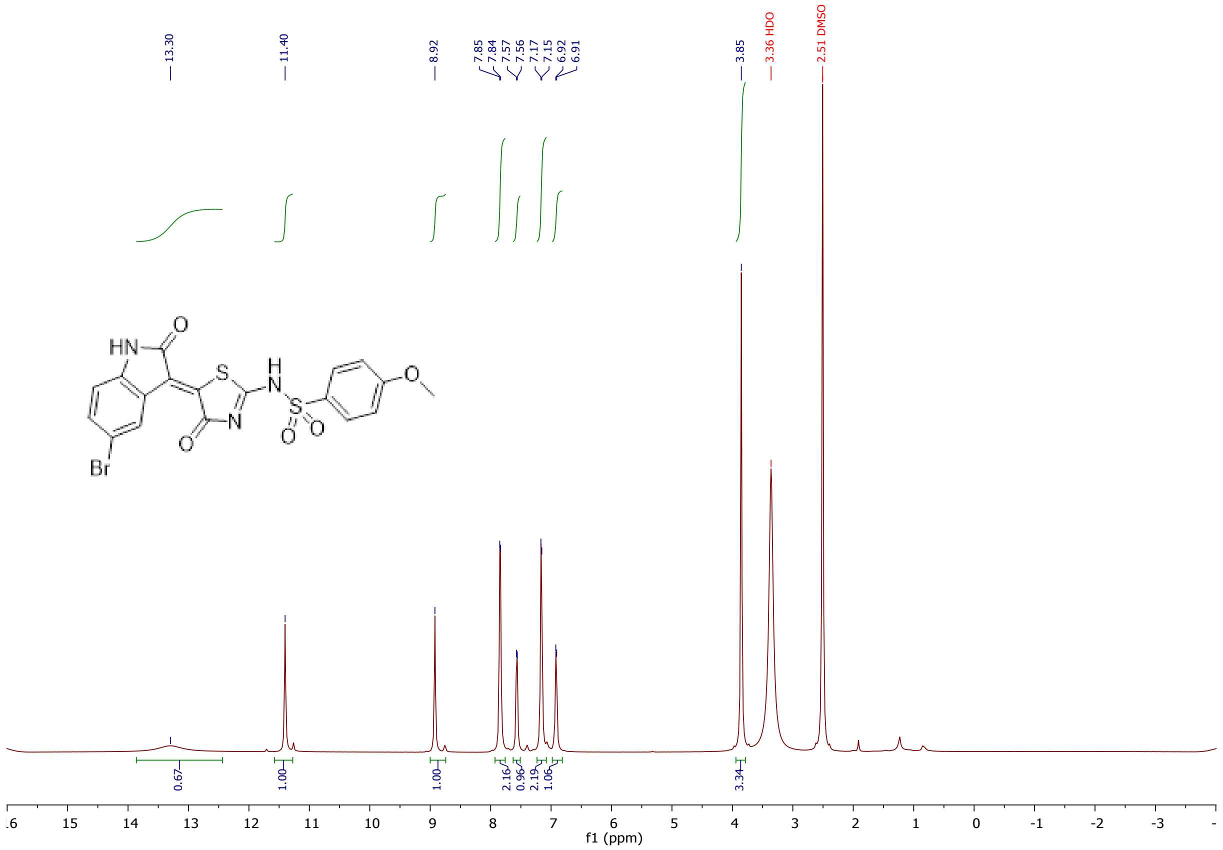

^{13}**C NMR** Spectrum of **8** (151 MHz, DMSO-d_6):

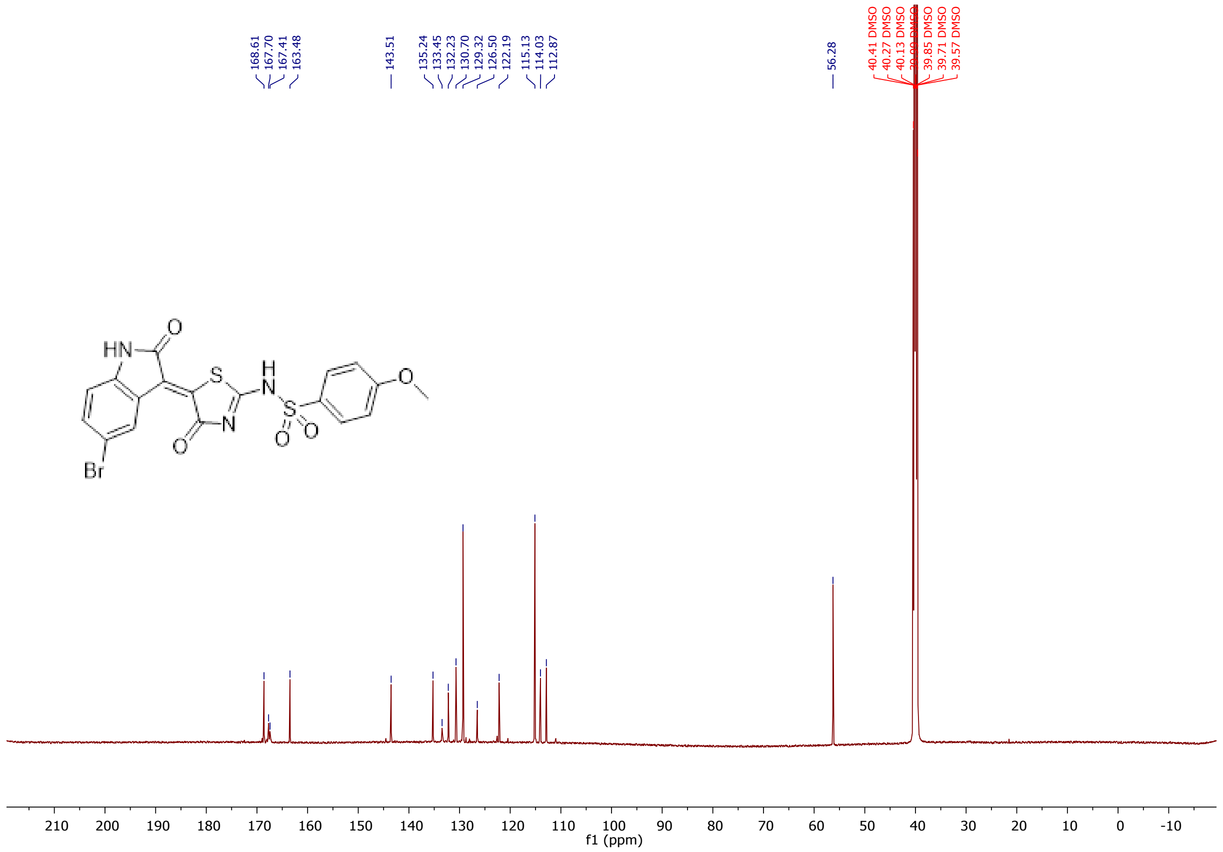

^{1}**H NMR** Spectrum of **9** (600 MHz, DMSO-d_6):

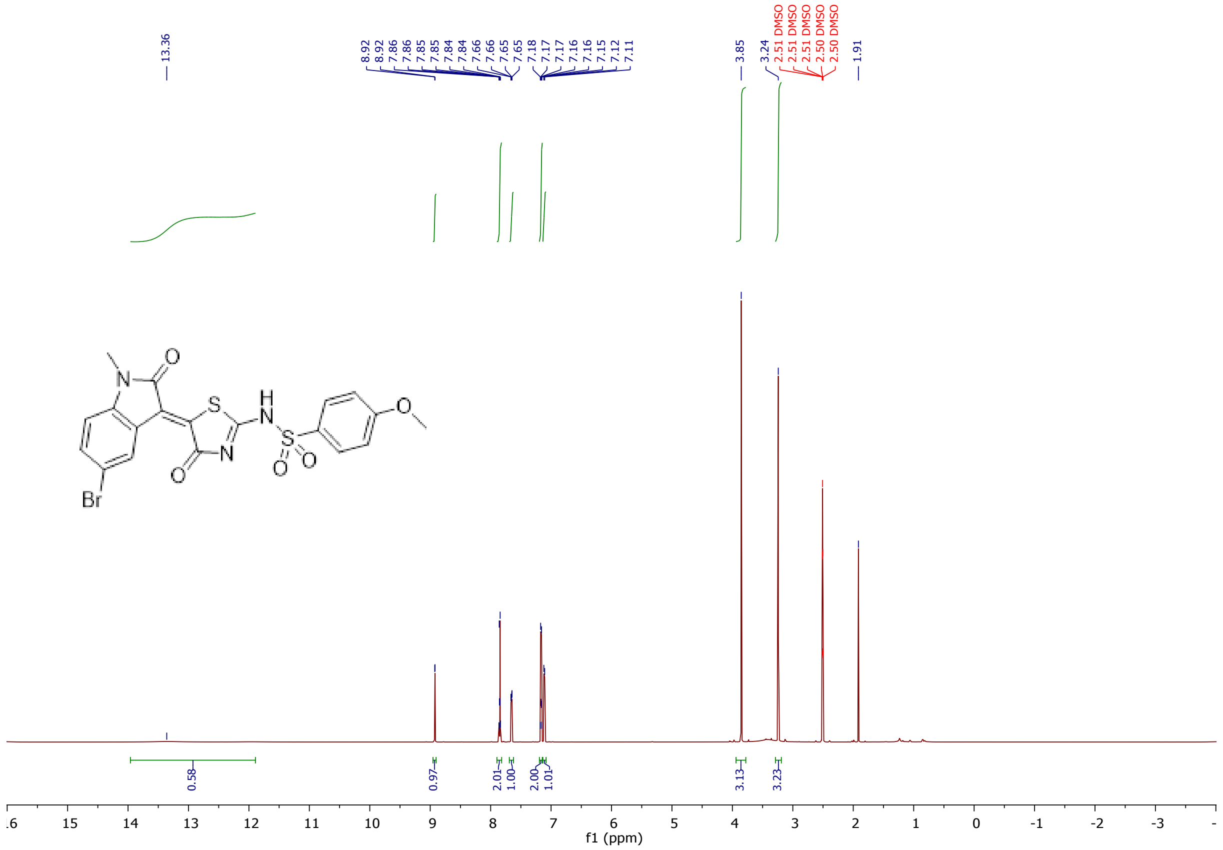

^{13}C NMR Spectrum of **9** (151 MHz, DMSO-d_6):

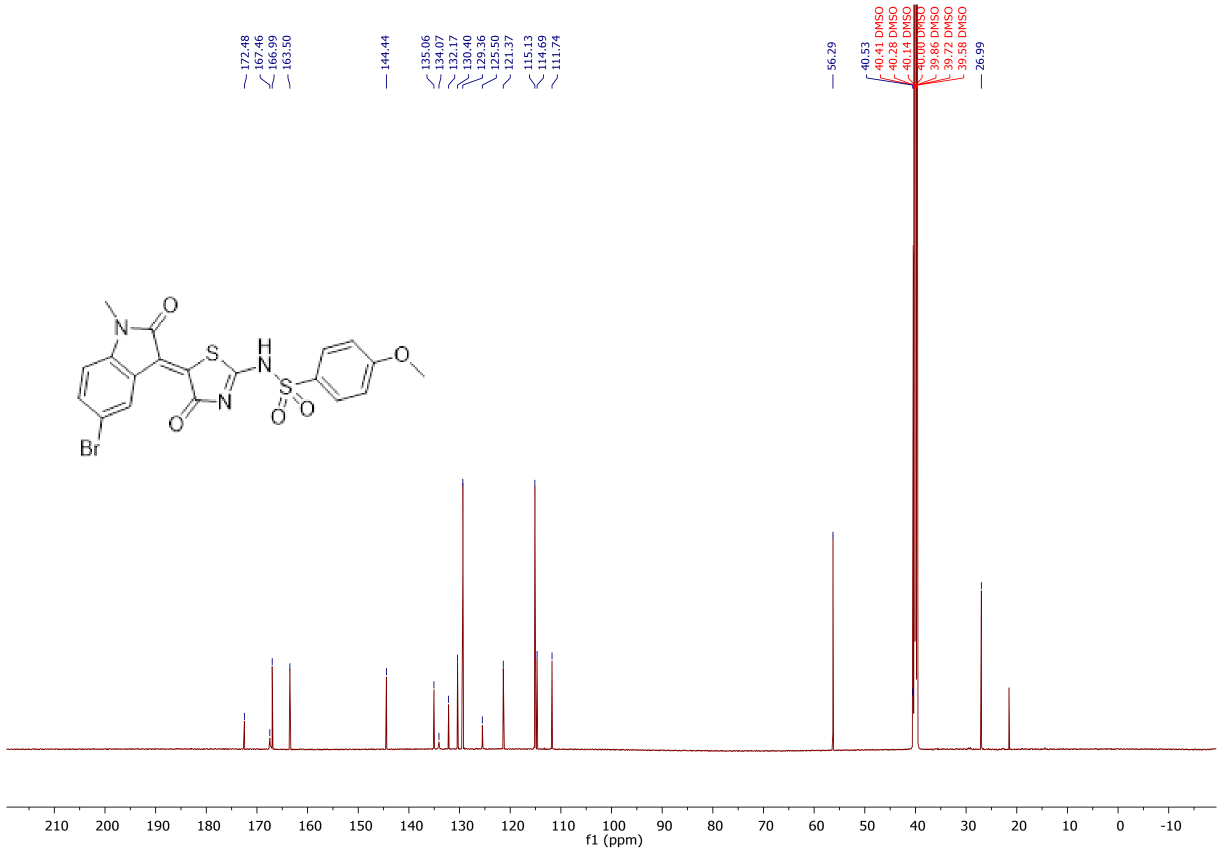

^{1}H NMR Spectrum of **10** (600 MHz, DMSO-d_6):

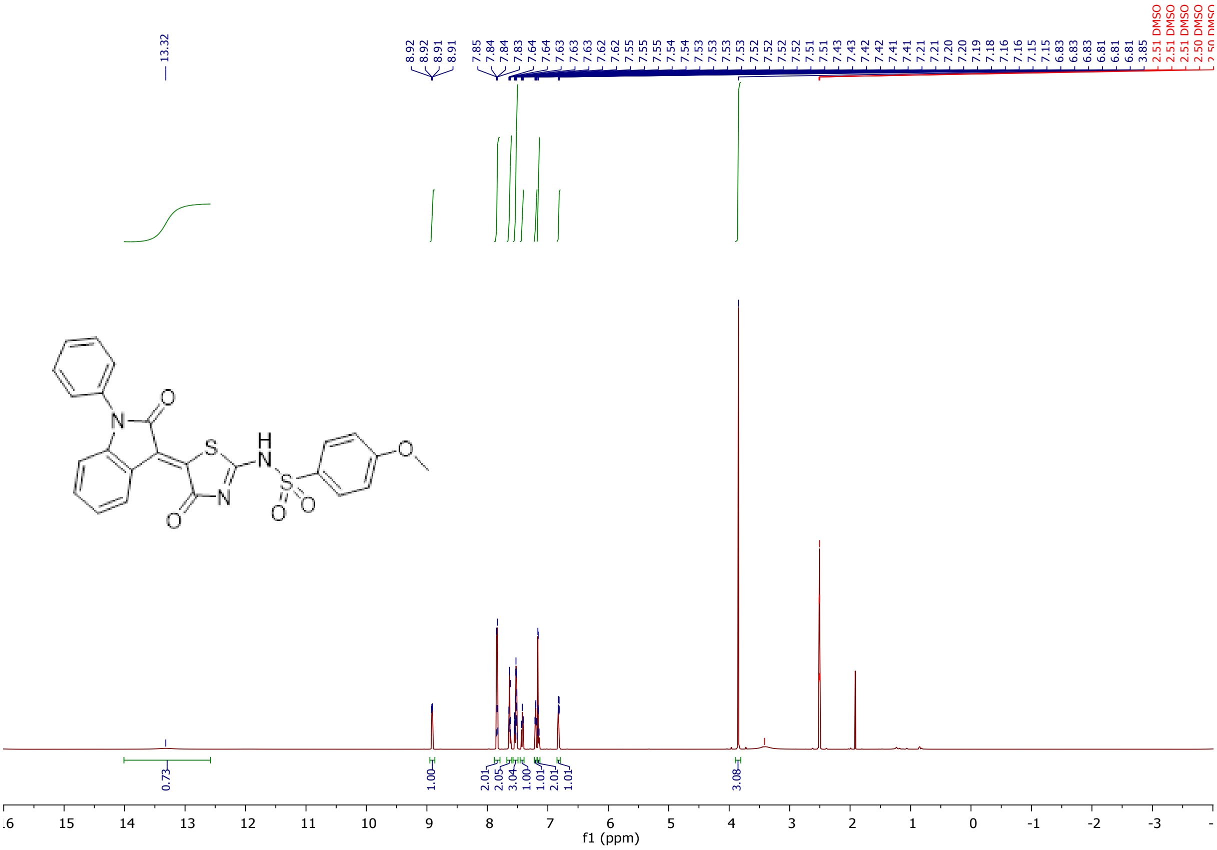

^{13}C **NMR** Spectrum of **10** (151 MHz, DMSO-d_6):

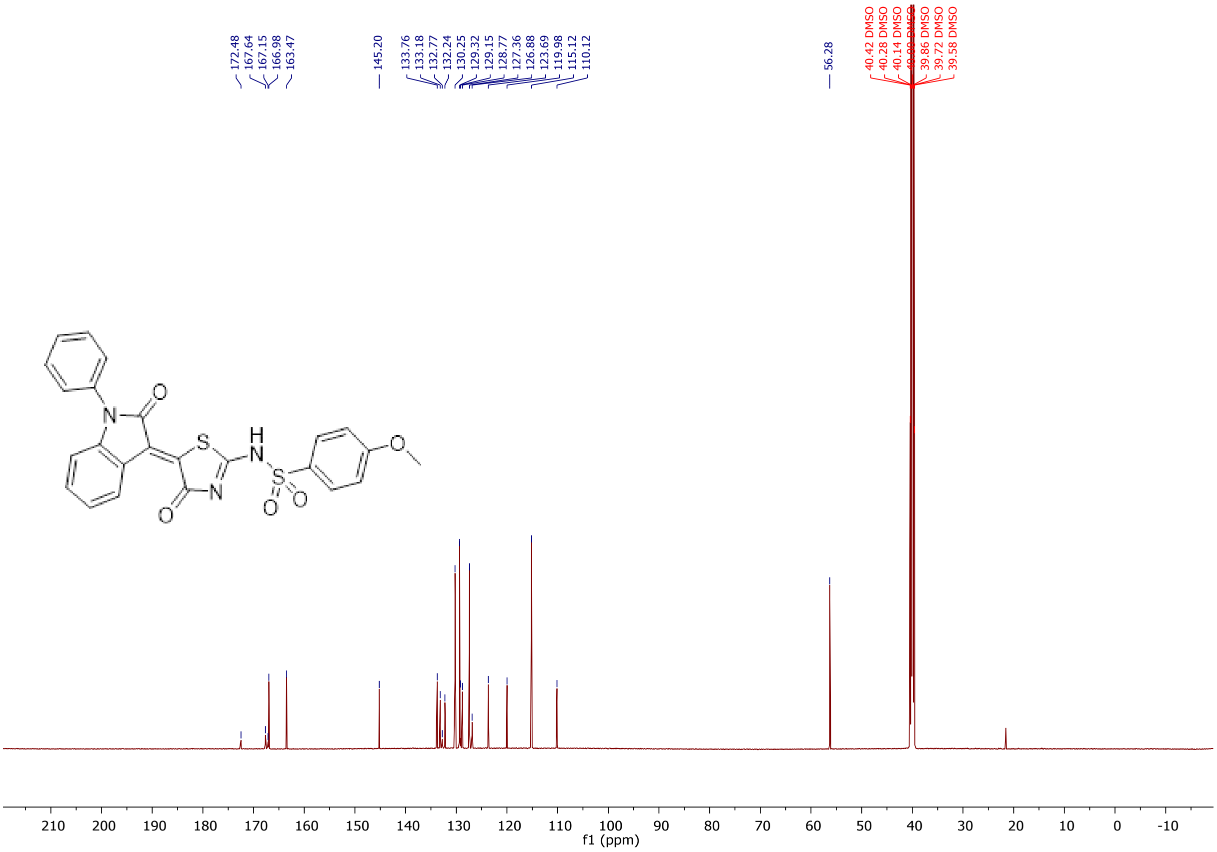

^{1}H **NMR** Spectrum of **11** (600 MHz, DMSO-d_6):

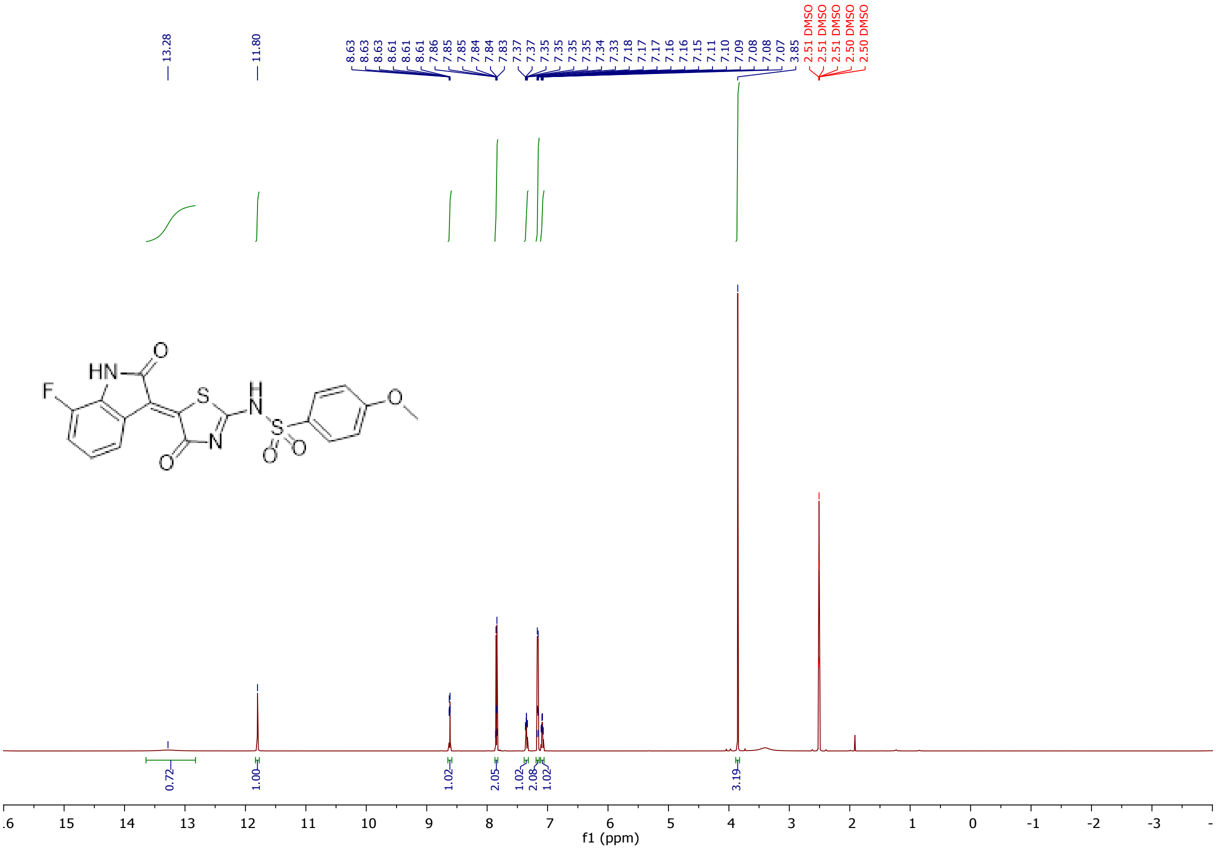

^{13}C NMR Spectrum of **11** (151 MHz, DMSO-d_6):

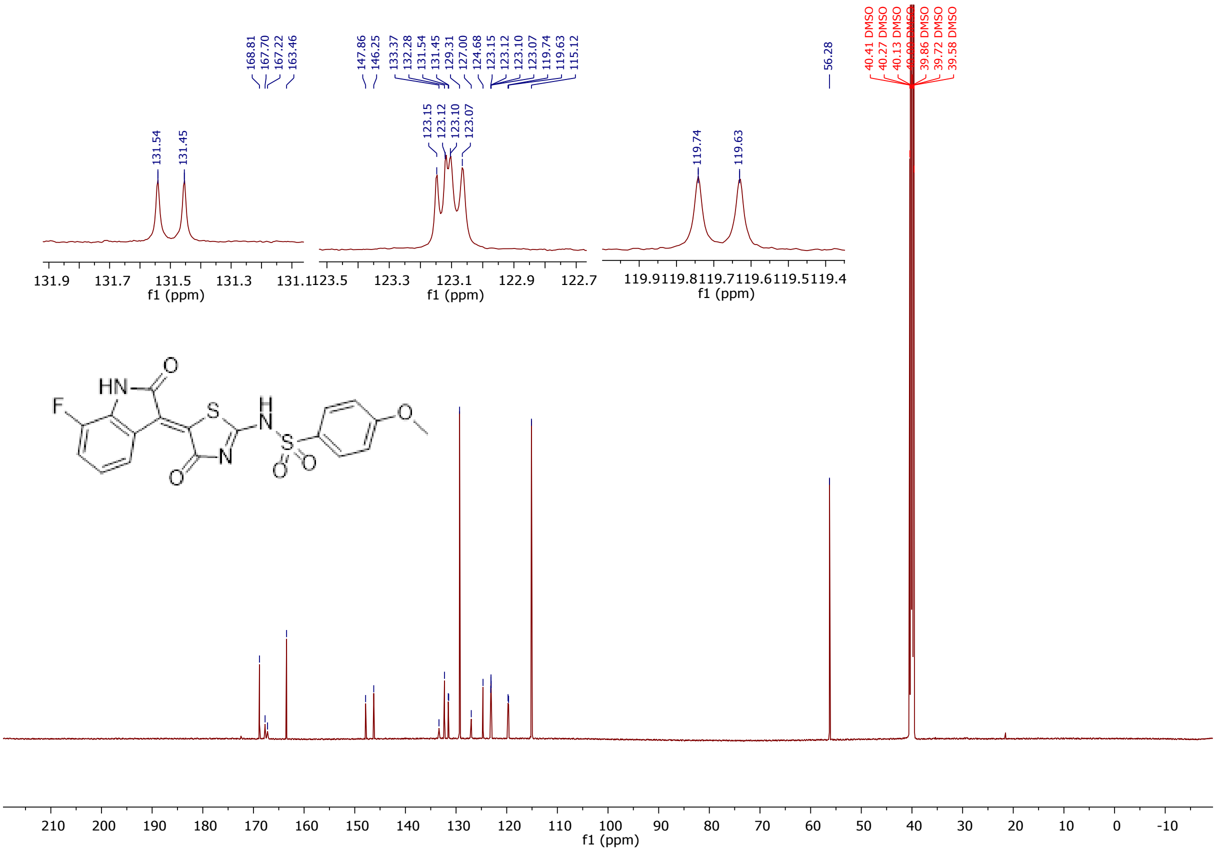

^{19}F NMR Spectrum of **11** (470 MHz, DMSO-d_6):

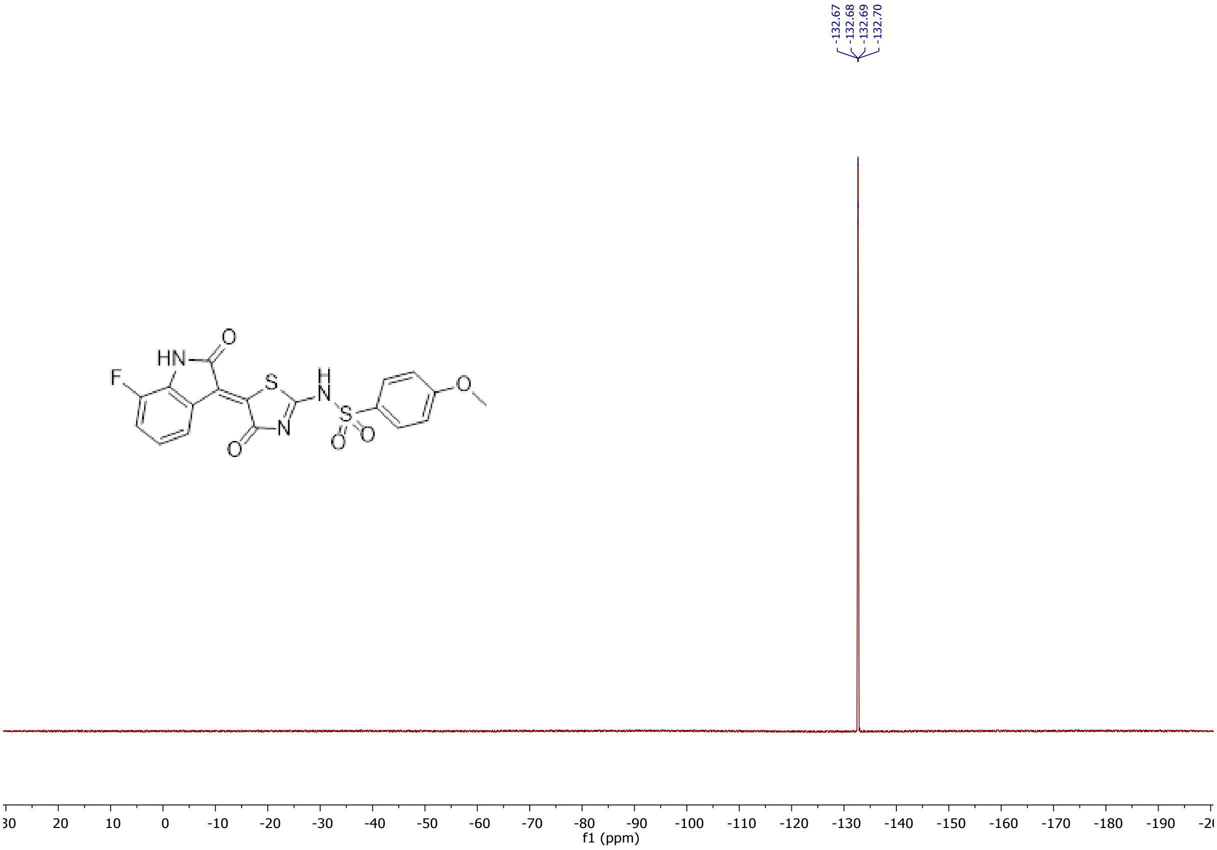

^{1}H NMR Spectrum of **12** (600 MHz, DMSO-d_6):

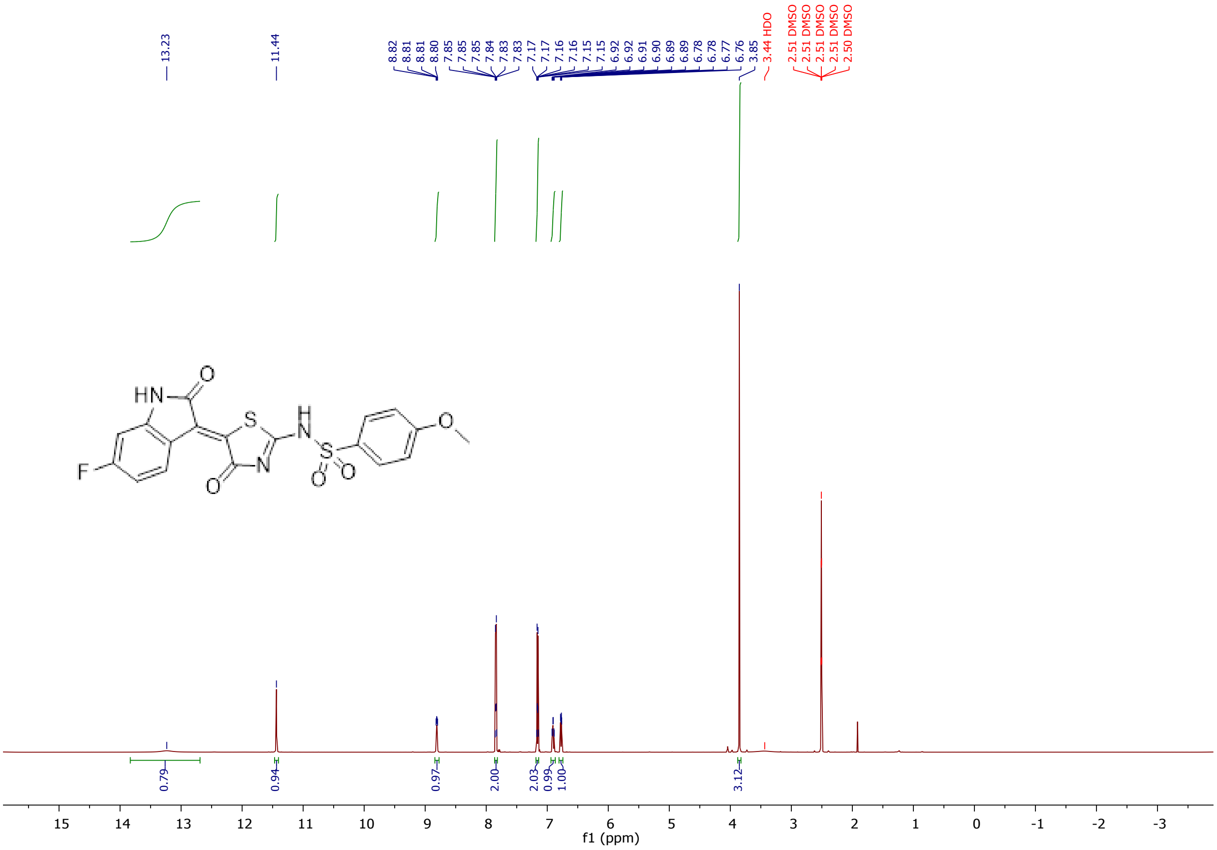

^{13}C NMR Spectrum of **12** (151 MHz, DMSO-d_6):

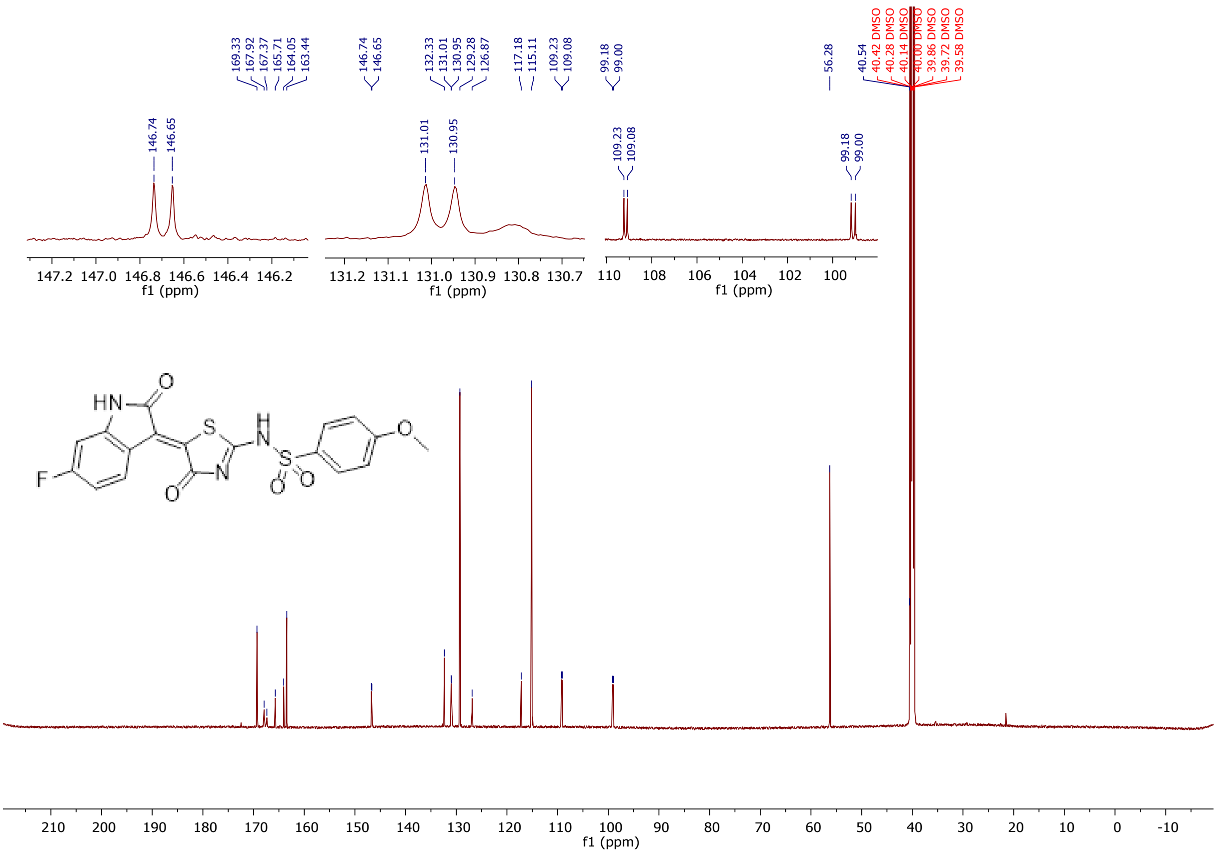

^{19}F NMR Spectrum of **12** (470 MHz, DMSO-d_6):

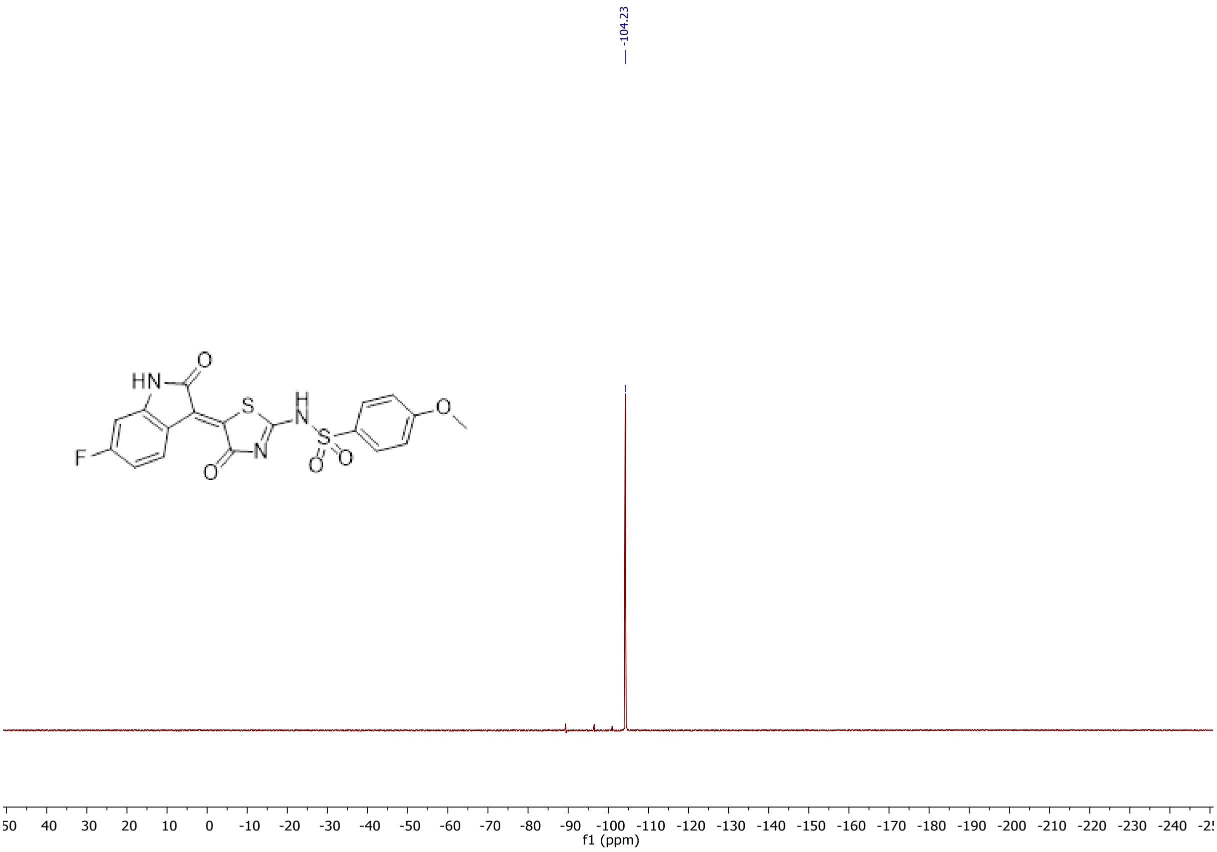

^{1}H NMR Spectrum of **13** (600 MHz, DMSO-d_6):

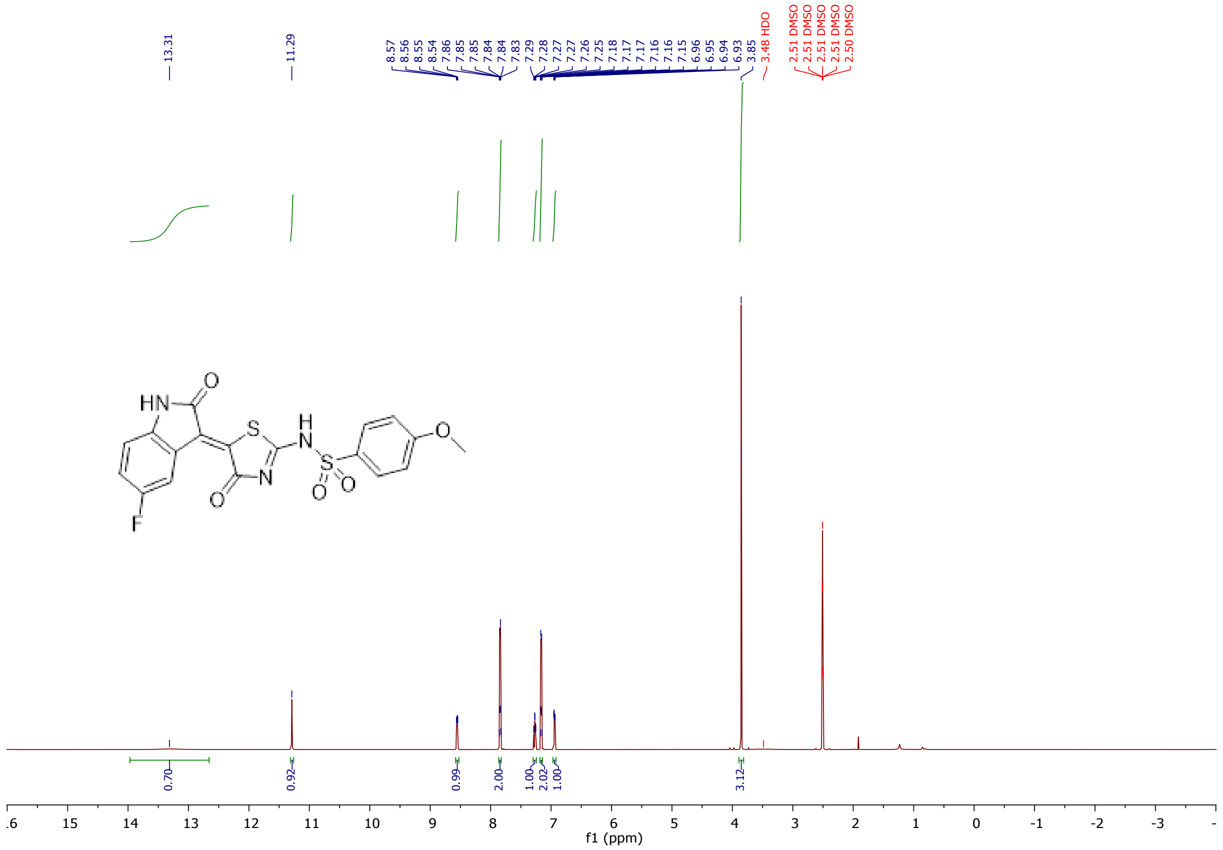

^{13}C NMR Spectrum of **13** (151 MHz, DMSO-d_6):

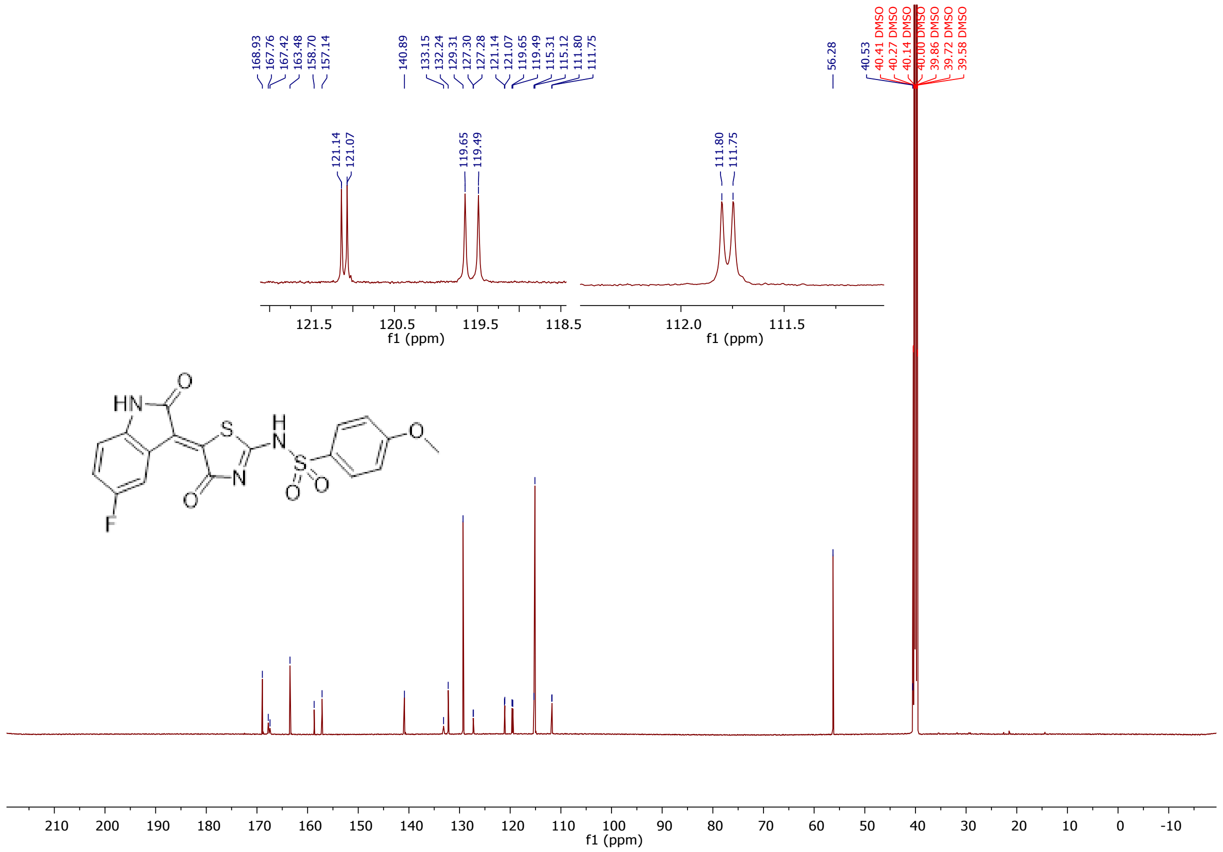

^{19}F NMR Spectrum of **13** (470 MHz, DMSO-d_6):

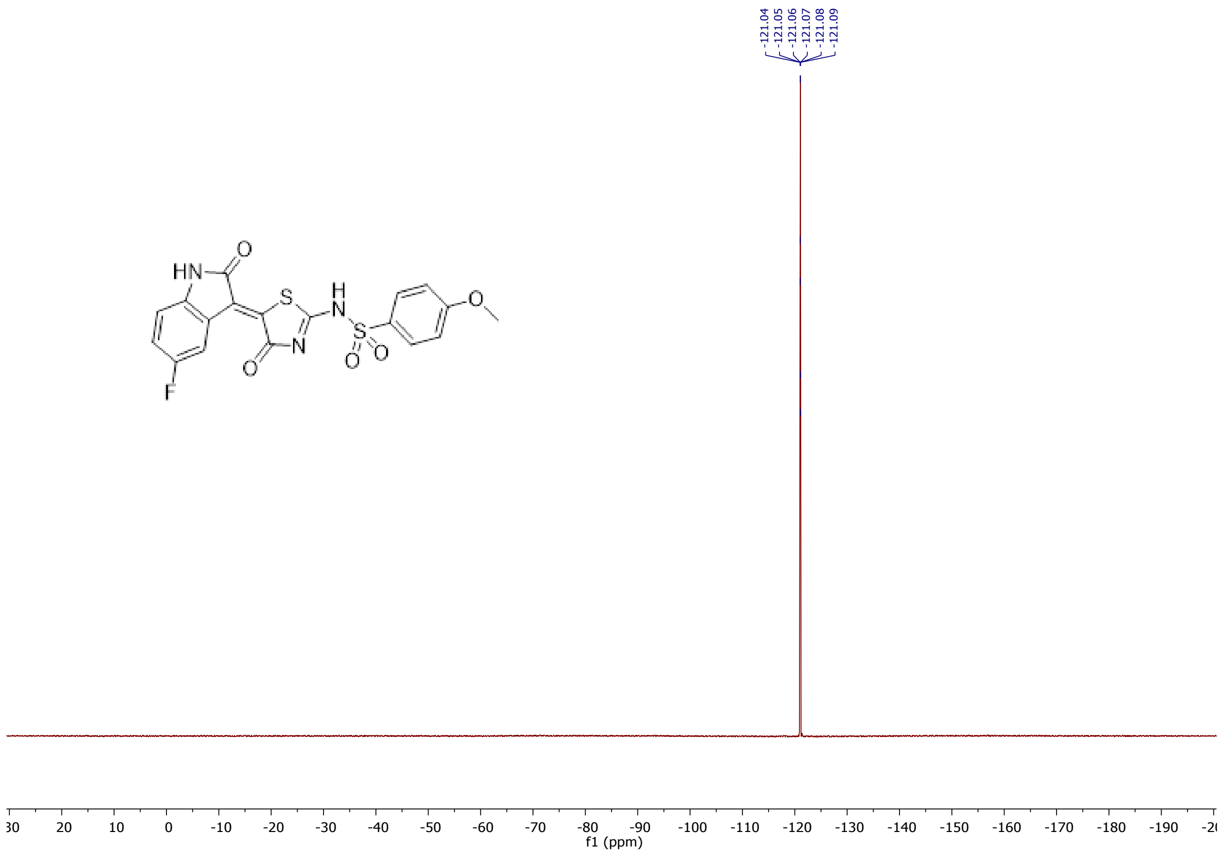

^{1}H NMR Spectrum of **14** (600 MHz, DMSO-d_6):

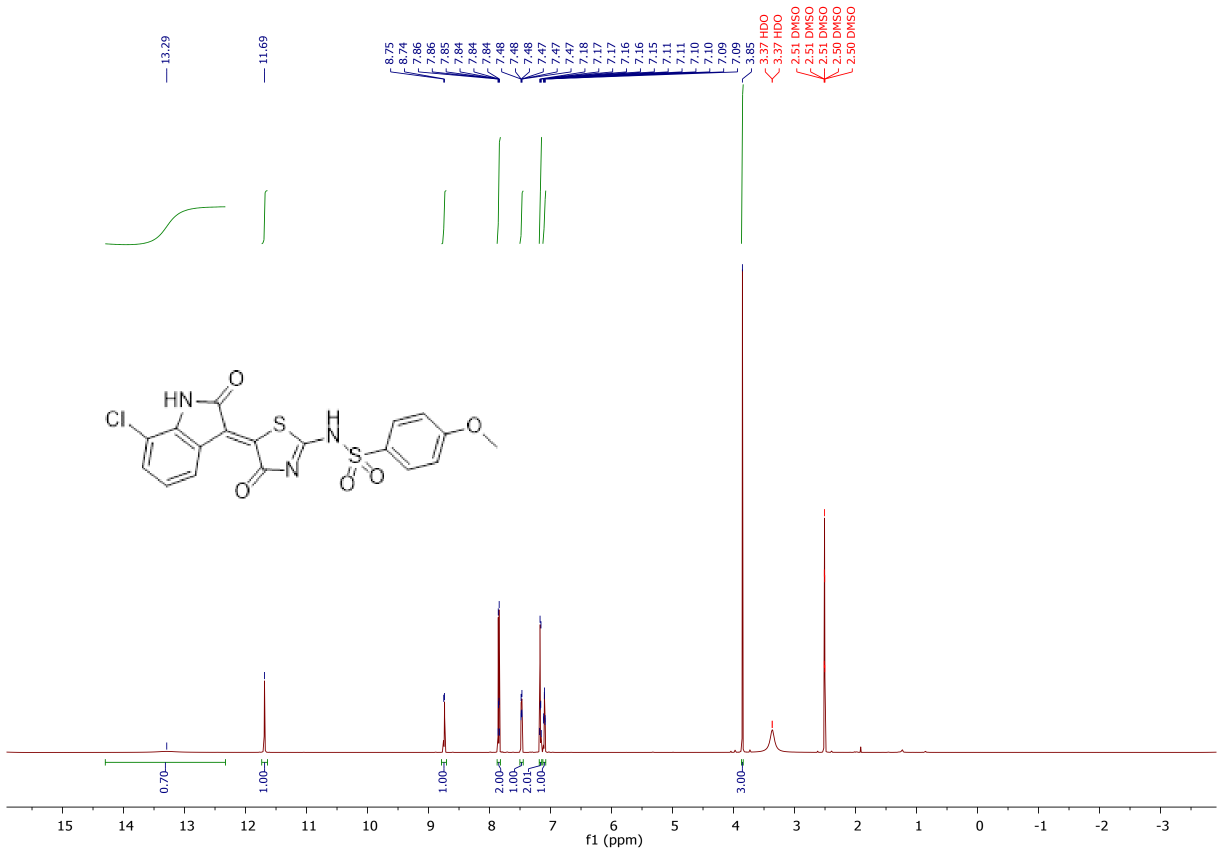

^{13}C NMR Spectrum of **14** (151 MHz, DMSO-d_6):

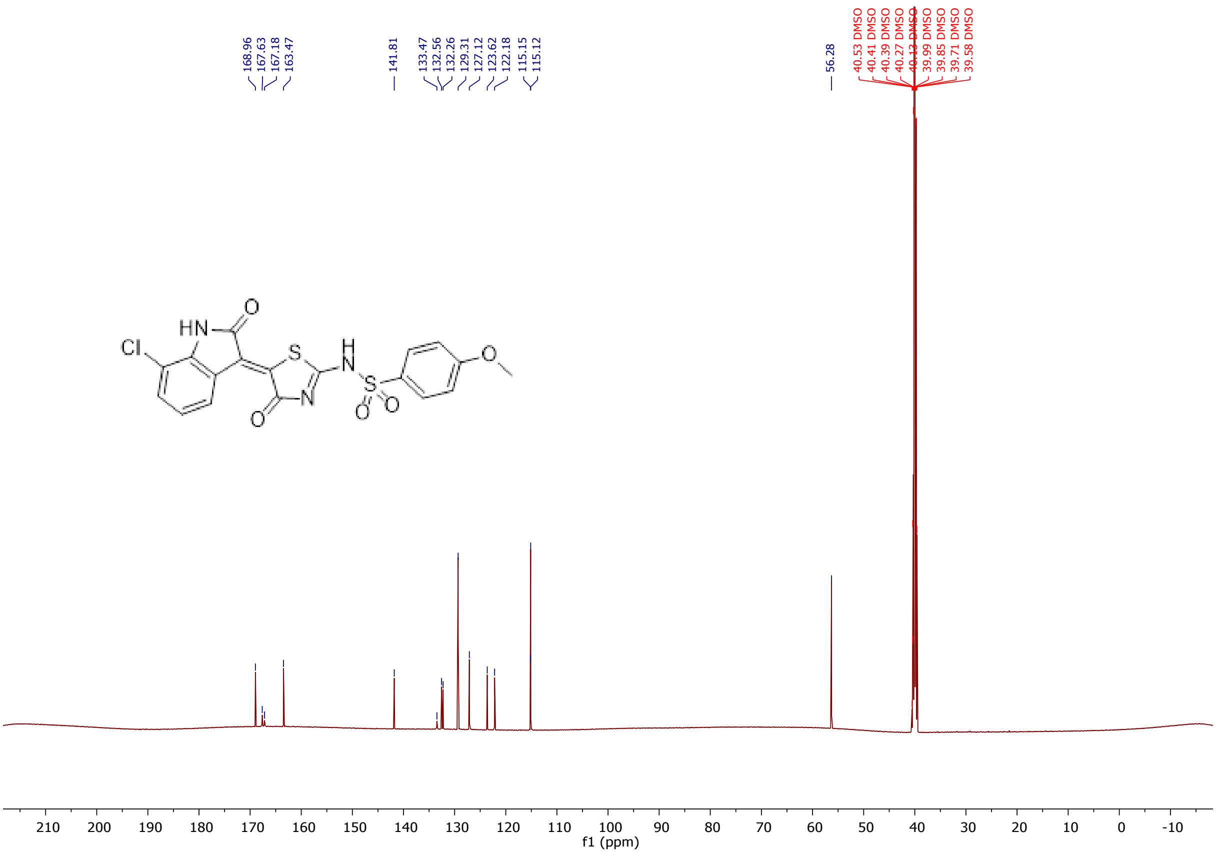

^{1}H NMR Spectrum of **15** (600 MHz, DMSO-d_6):

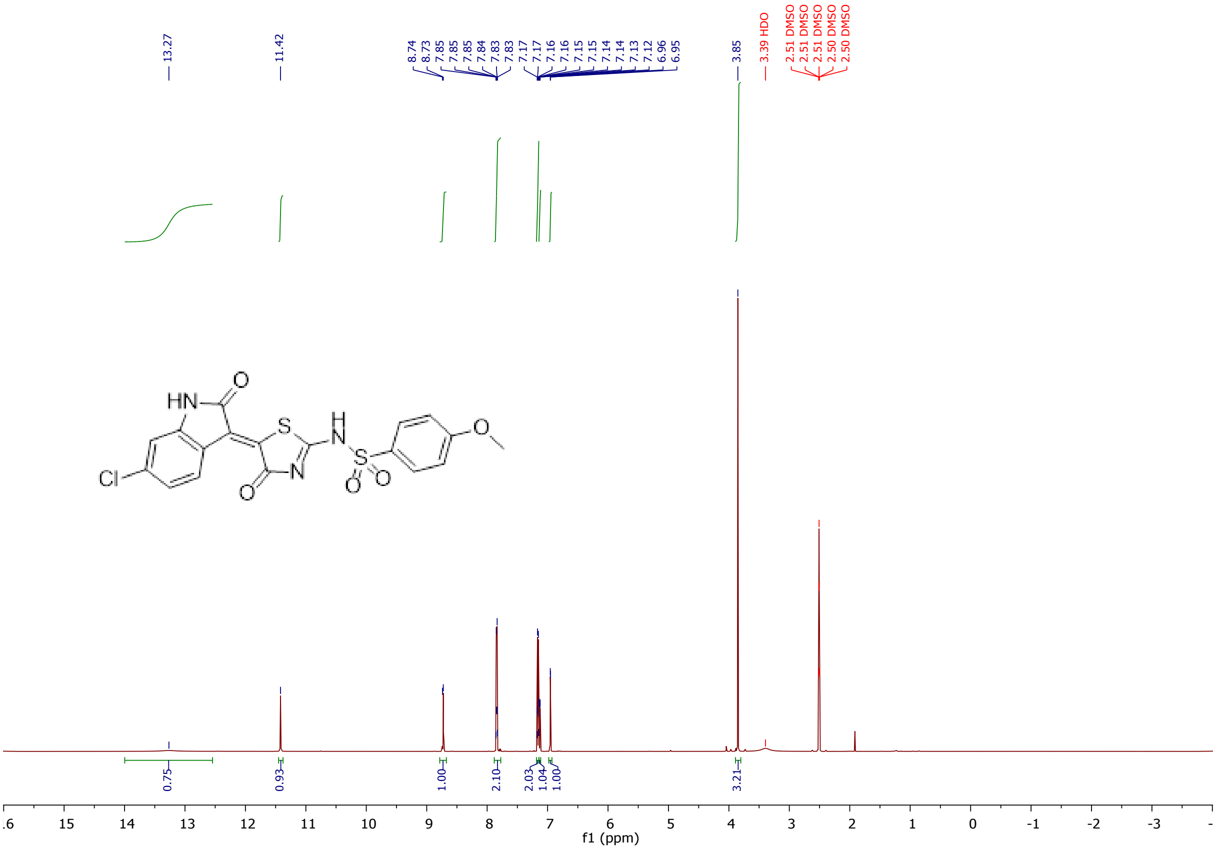

^{13}C NMR Spectrum of **15** (151 MHz, DMSO-d_6):

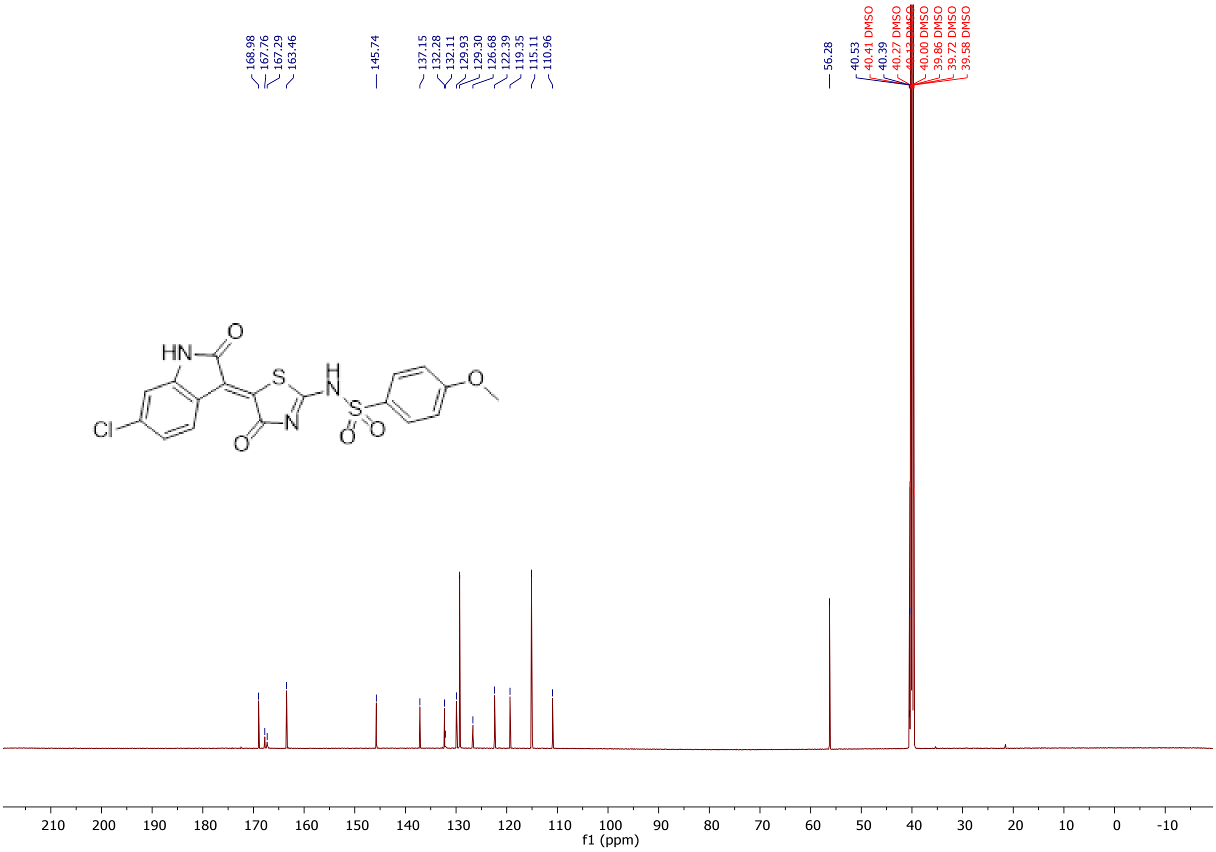

^{1}H NMR Spectrum of **16** (600 MHz, DMSO-d_6):

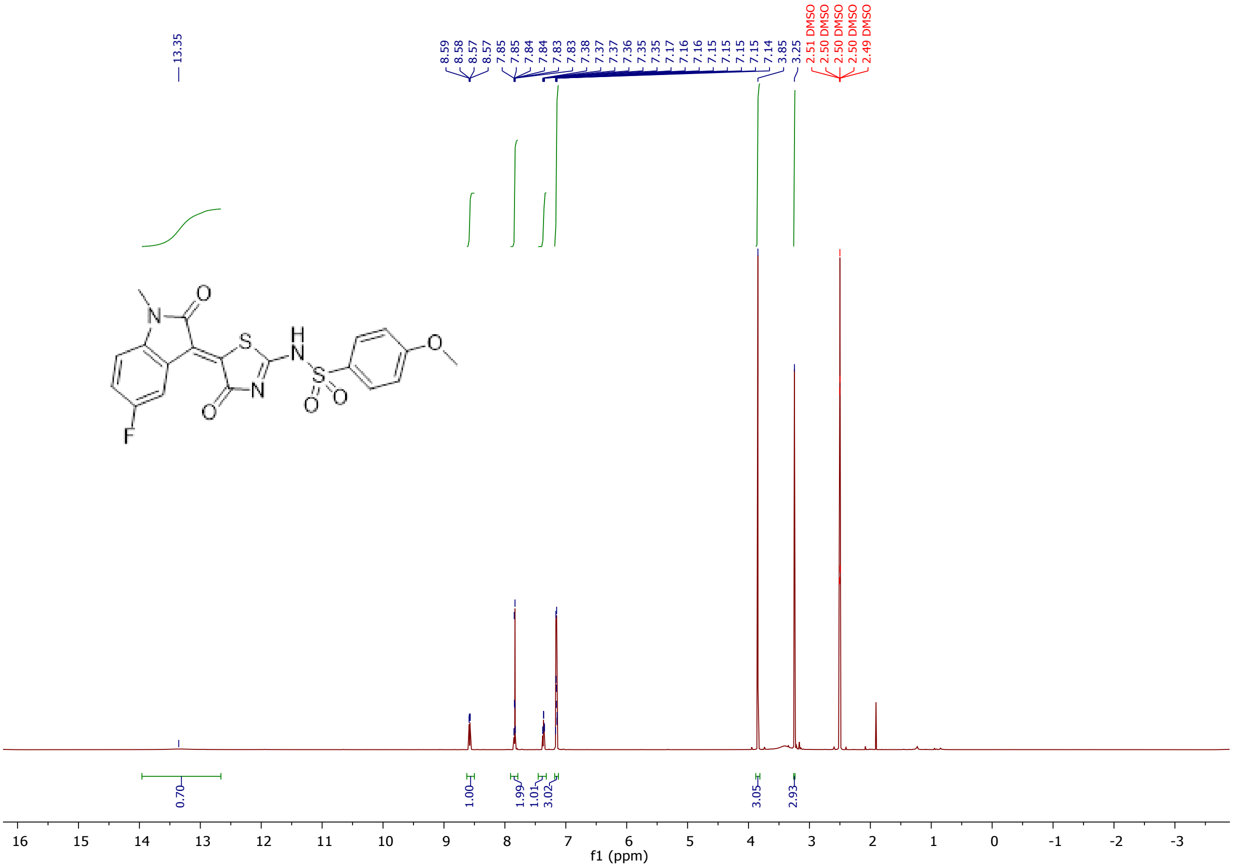

^{13}C NMR Spectrum of **16** (151 MHz, DMSO-d_6):

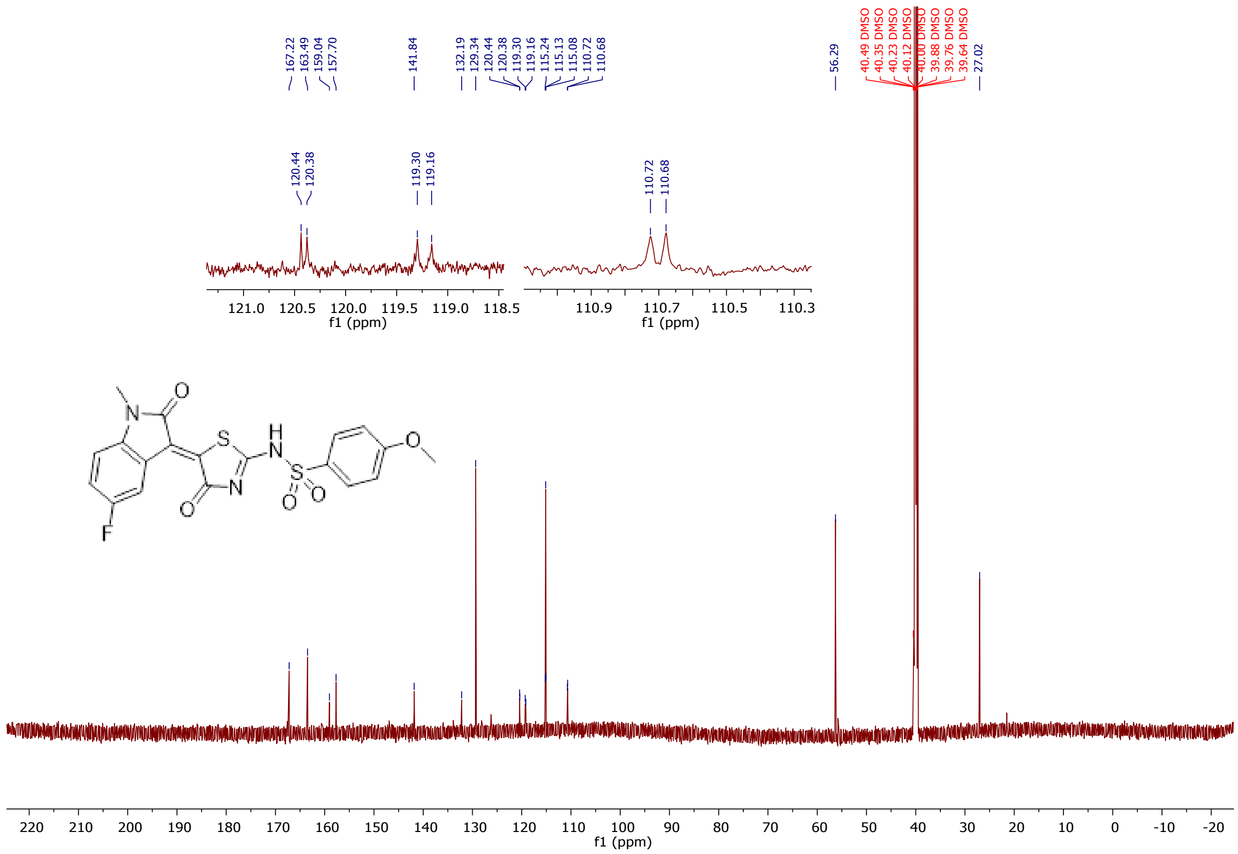

^{19}F NMR Spectrum of **16** (470 MHz, DMSO-d_6):

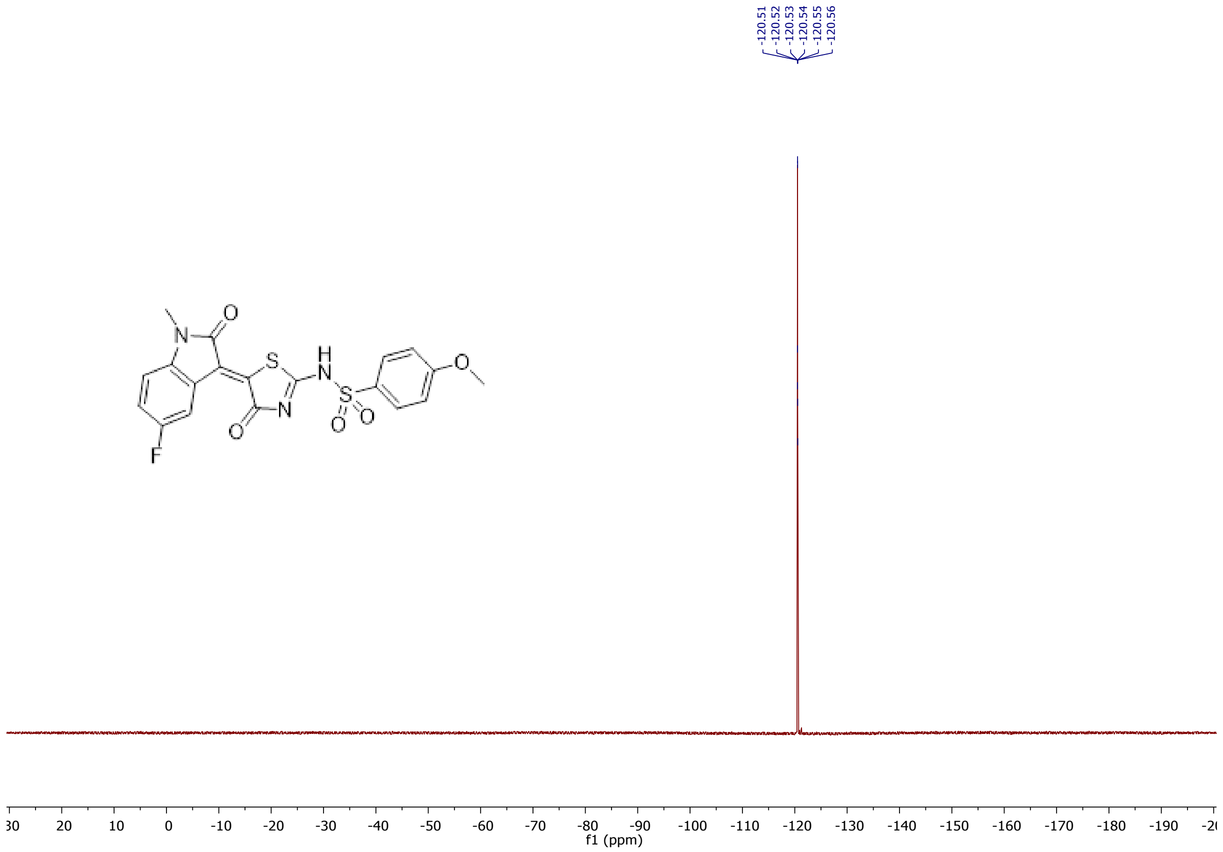

^{1}H NMR Spectrum of **17** (600 MHz, DMSO-d_6):

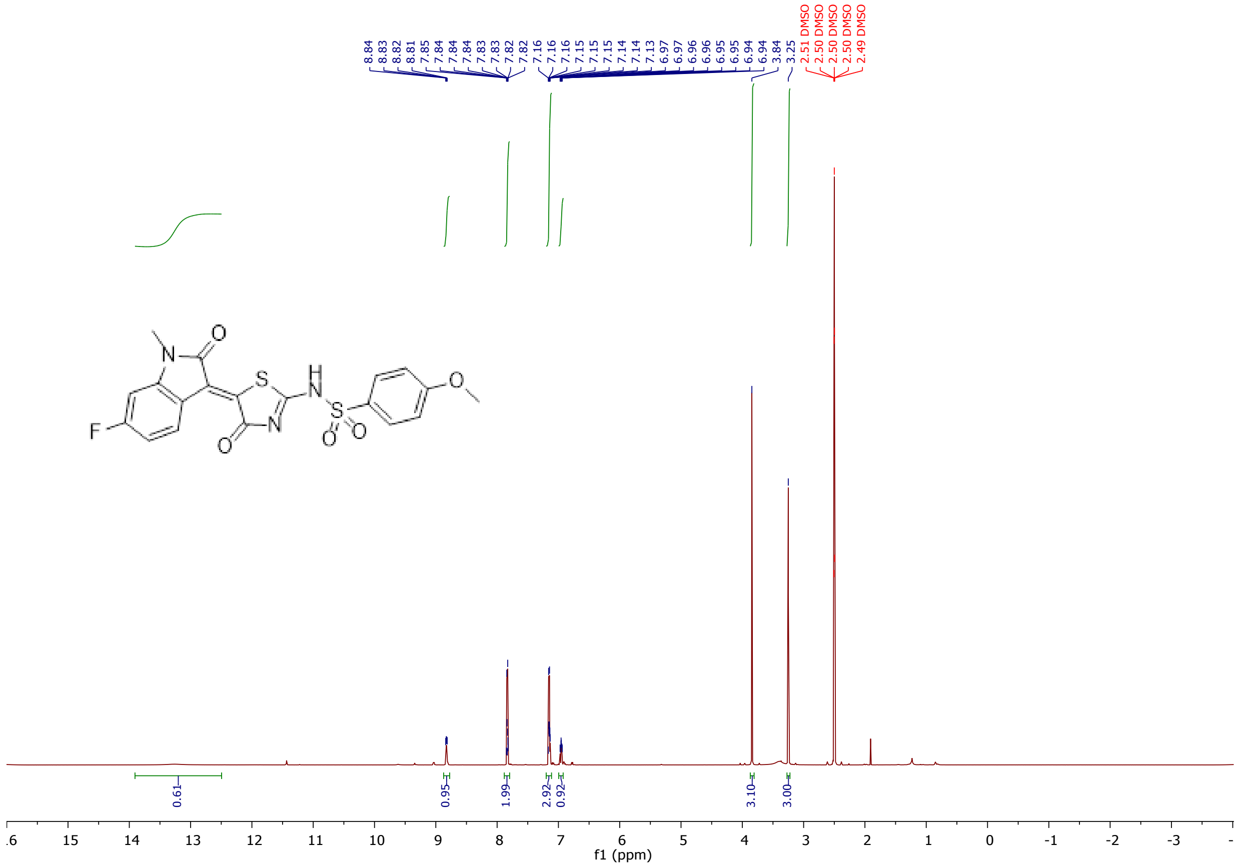

^{13}C NMR Spectrum of 17 (151 MHz, DMSO-d_6):

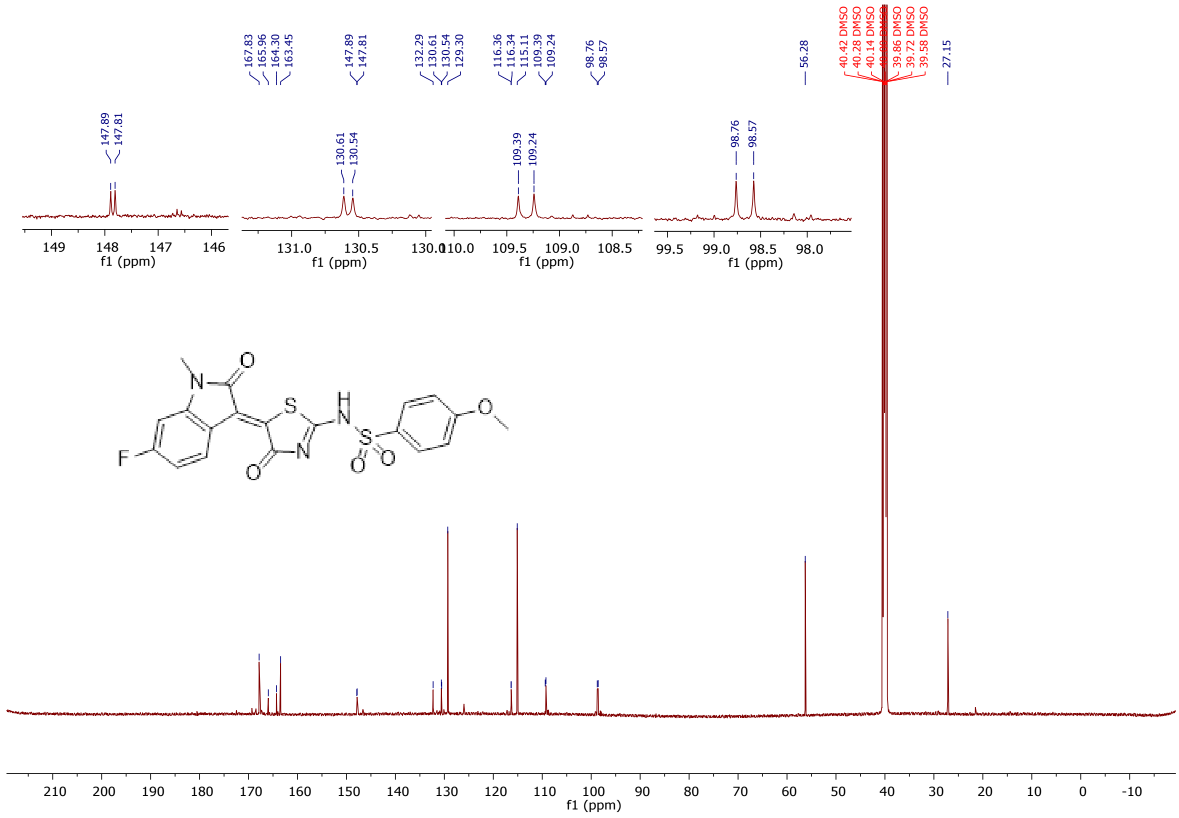

^{19}F NMR Spectrum of 17 (470 MHz, DMSO-d_6):

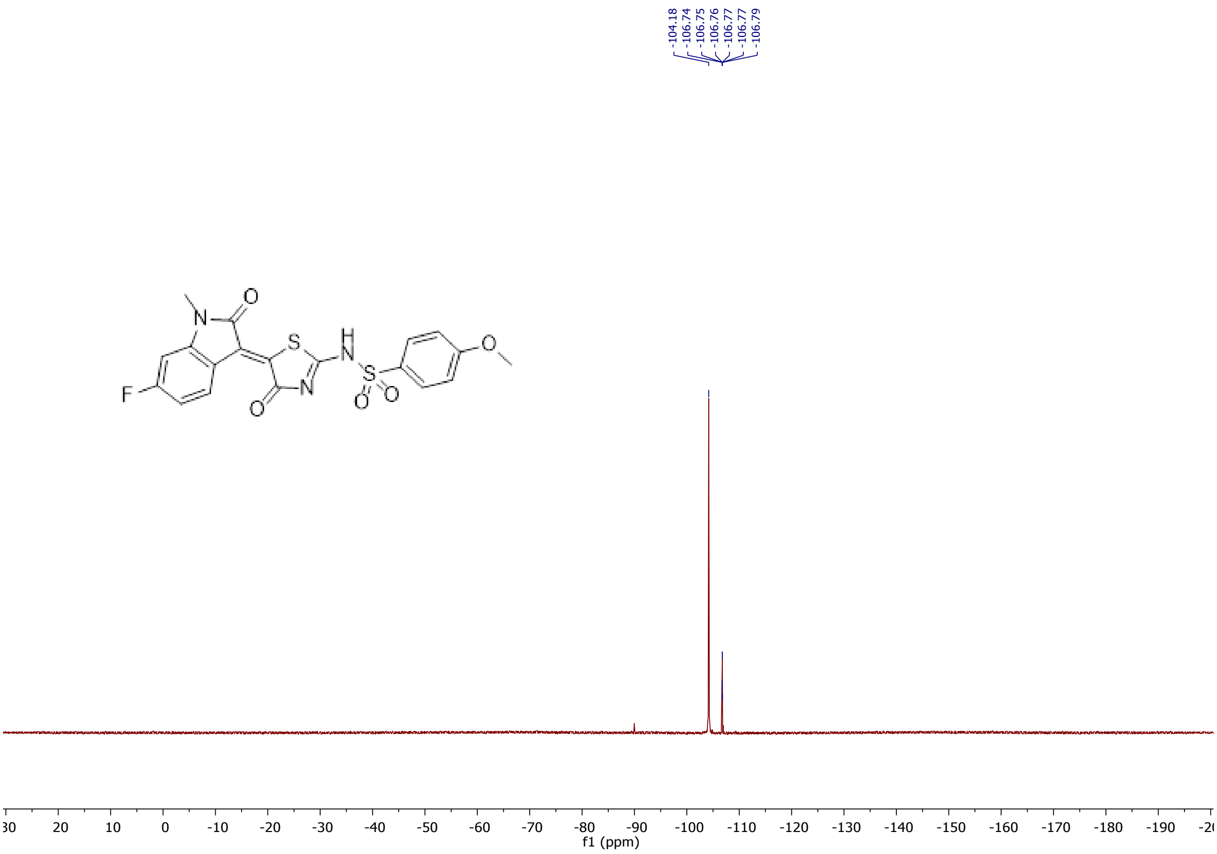
-104.18
-106.74
-106.75
-106.76
-106.77
-106.77
-106.79
F
N
O
S
H
N
S
O
O
O
N
O
30 20 10 0 -10 -20 -30 -40 -50 -60 -70 -80 -90 -100 -110 -120 -130 -140 -150 -160 -170 -180 -190 -20
f1 (ppm)

^{1}H NMR Spectrum of **18** (700 MHz, DMSO-d_6):

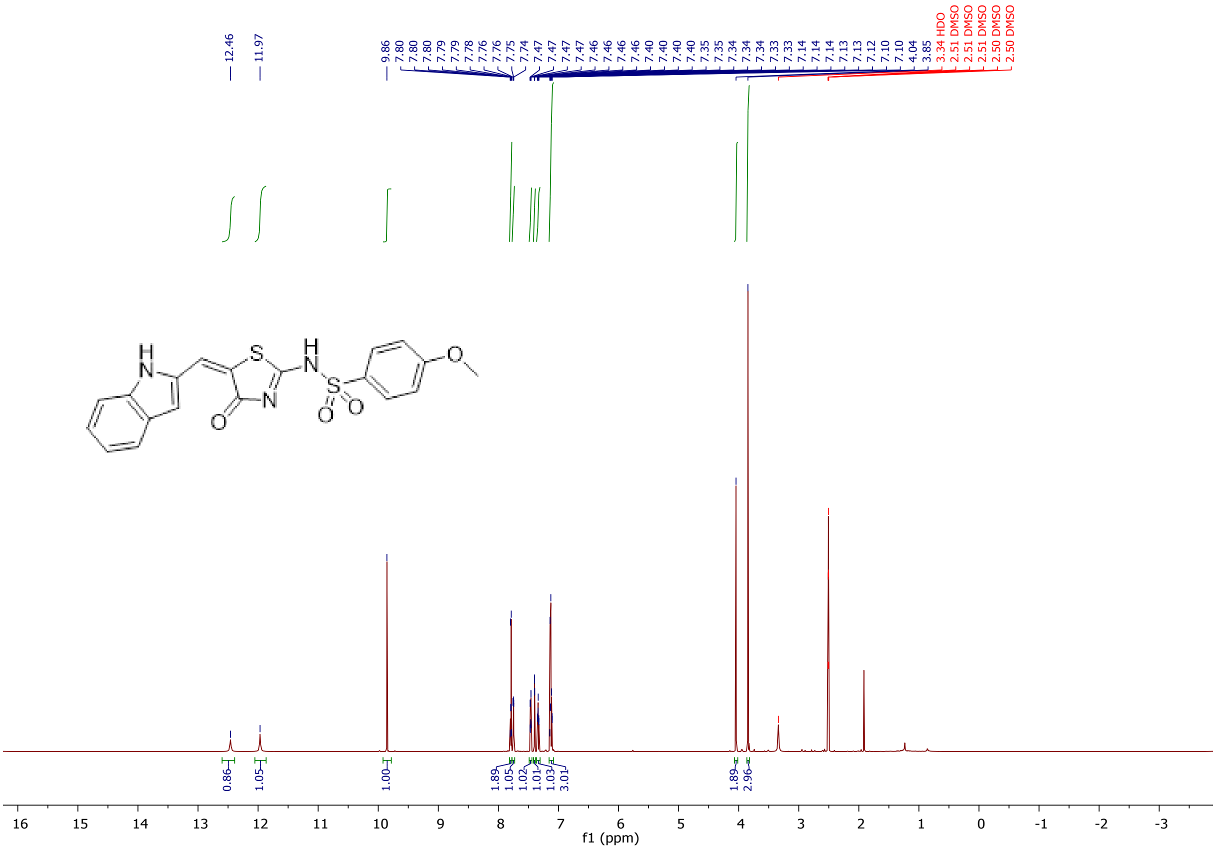

^{13}C NMR Spectrum of **18** (174 MHz, DMSO-d_6):

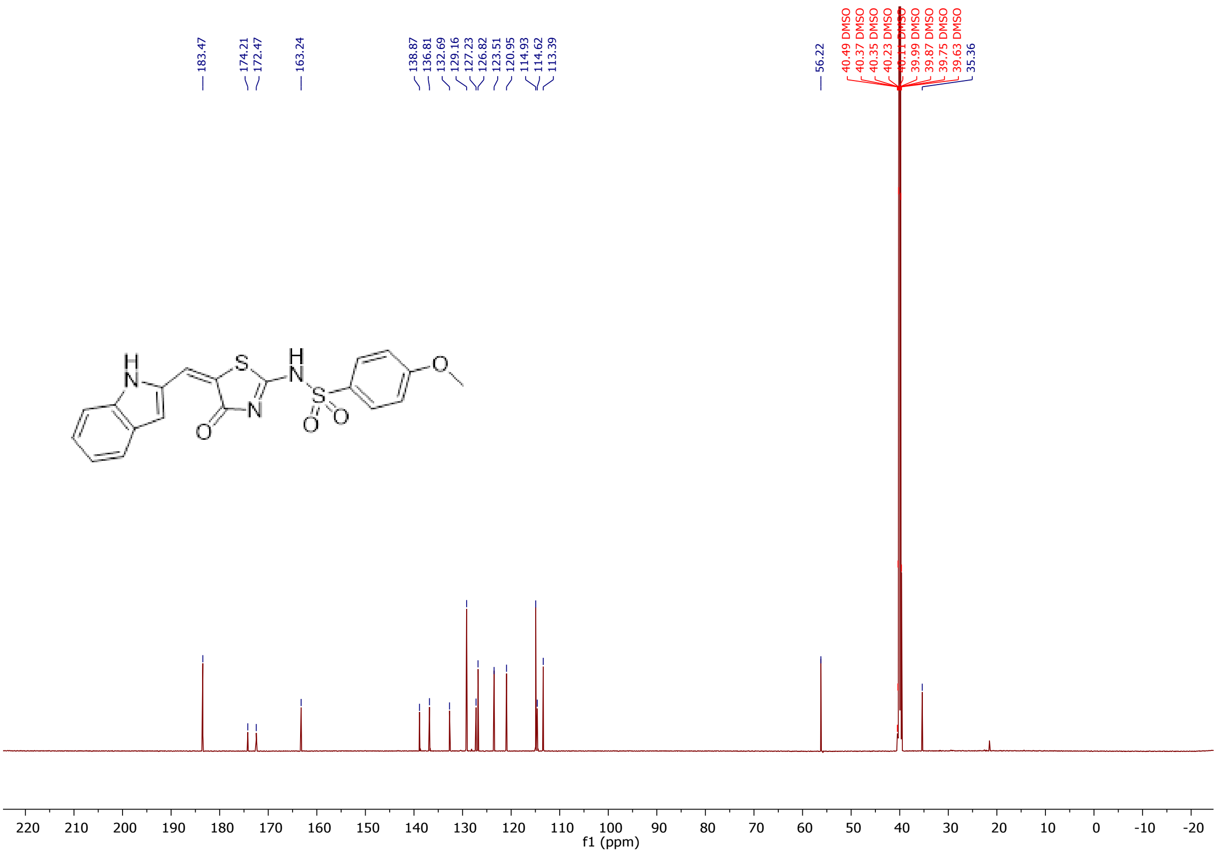

^{1}H NMR Spectrum of **19** (600 MHz, DMSO-d_6):

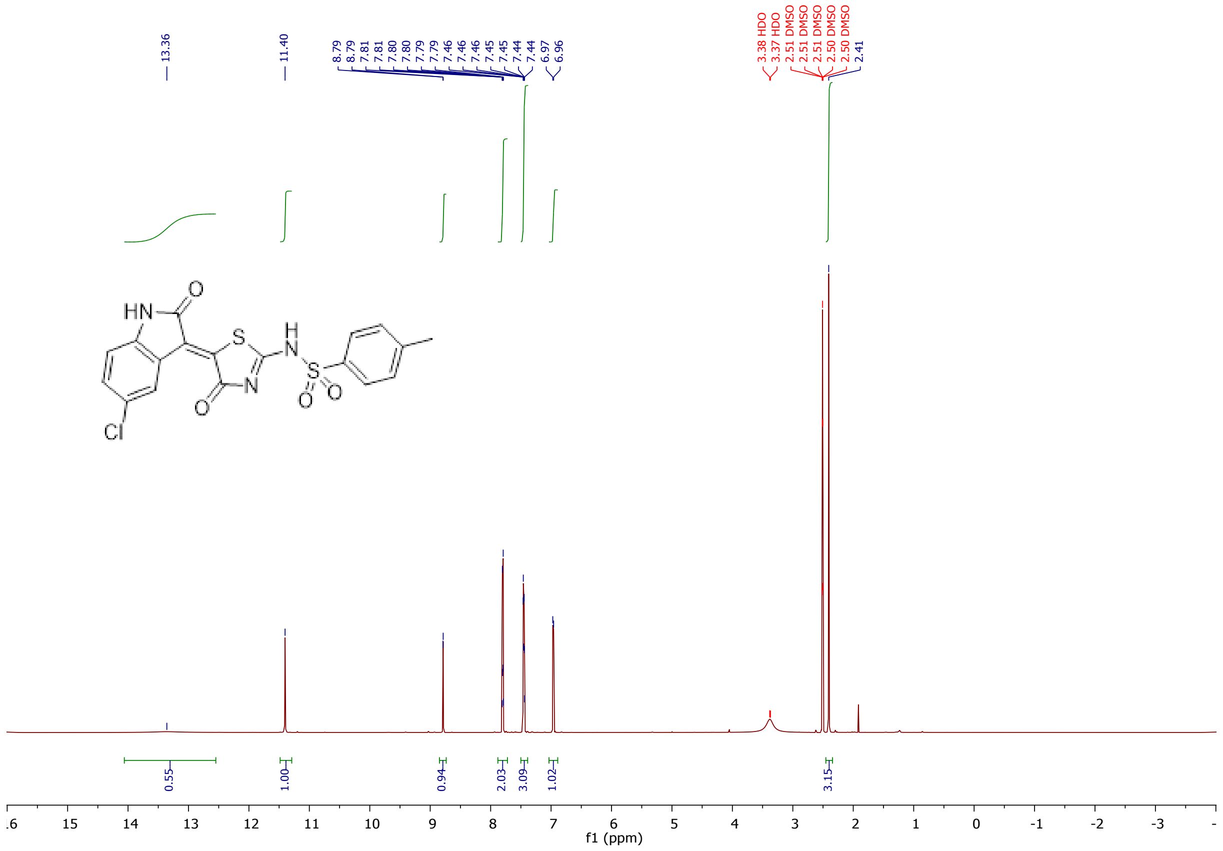

^{13}C NMR Spectrum of **19** (151 MHz, DMSO-d_6):

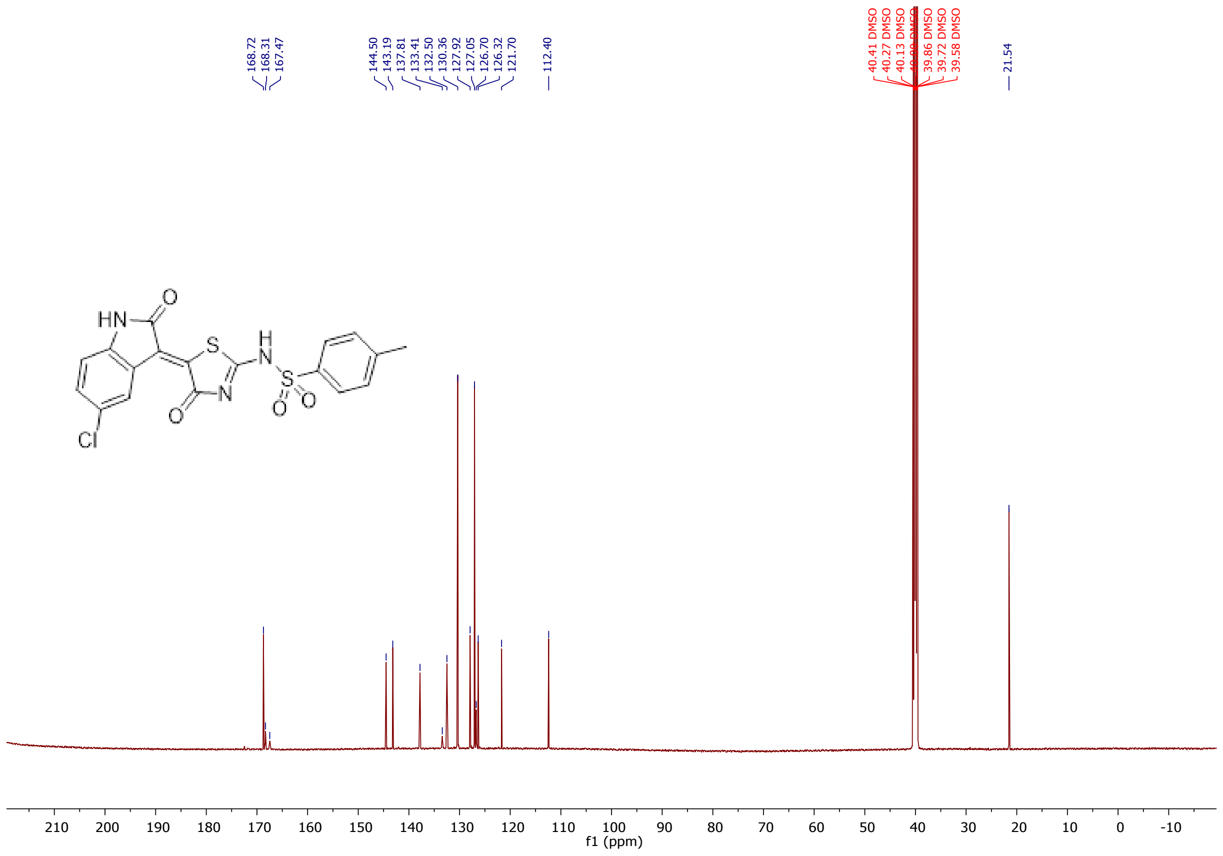

^{1}H NMR Spectrum of **20** (700 MHz, DMSO-d_6):

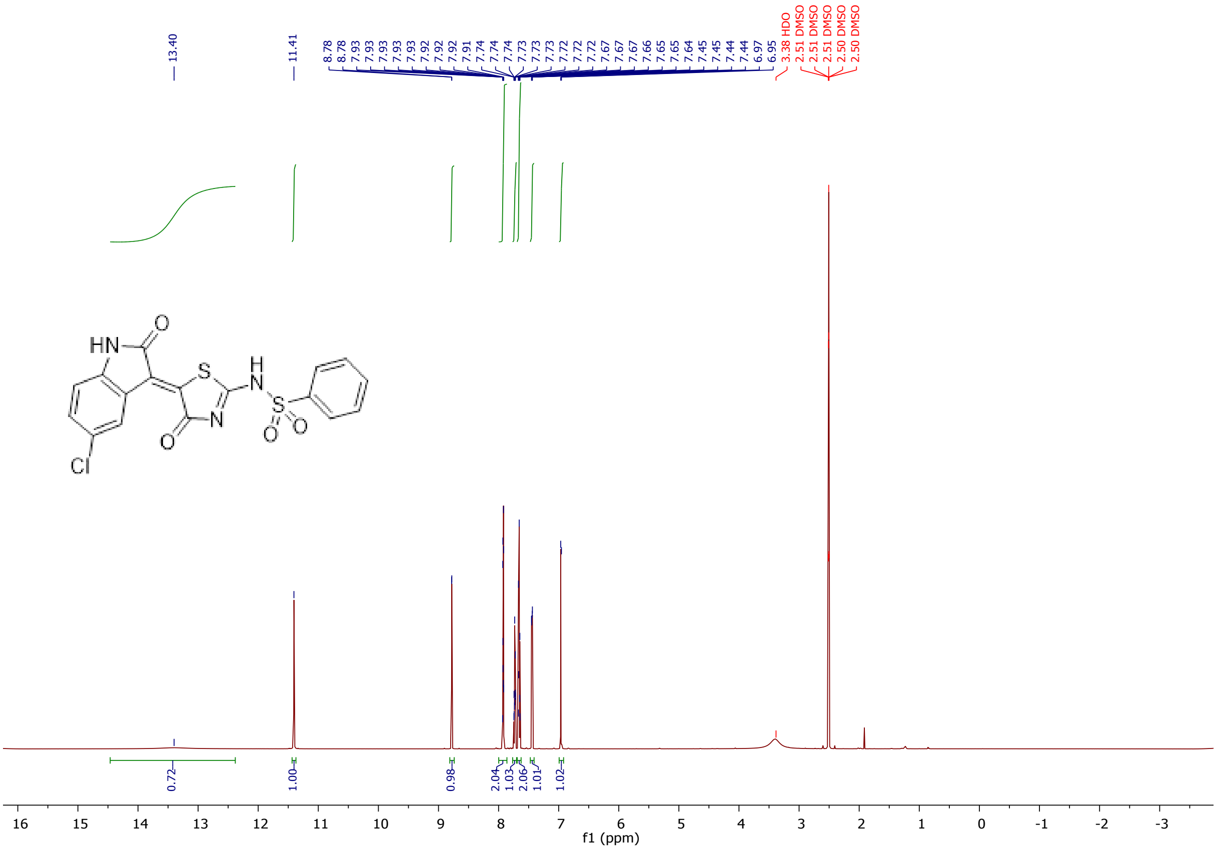

^{13}C NMR Spectrum of **20** (174 MHz, DMSO-d_6):

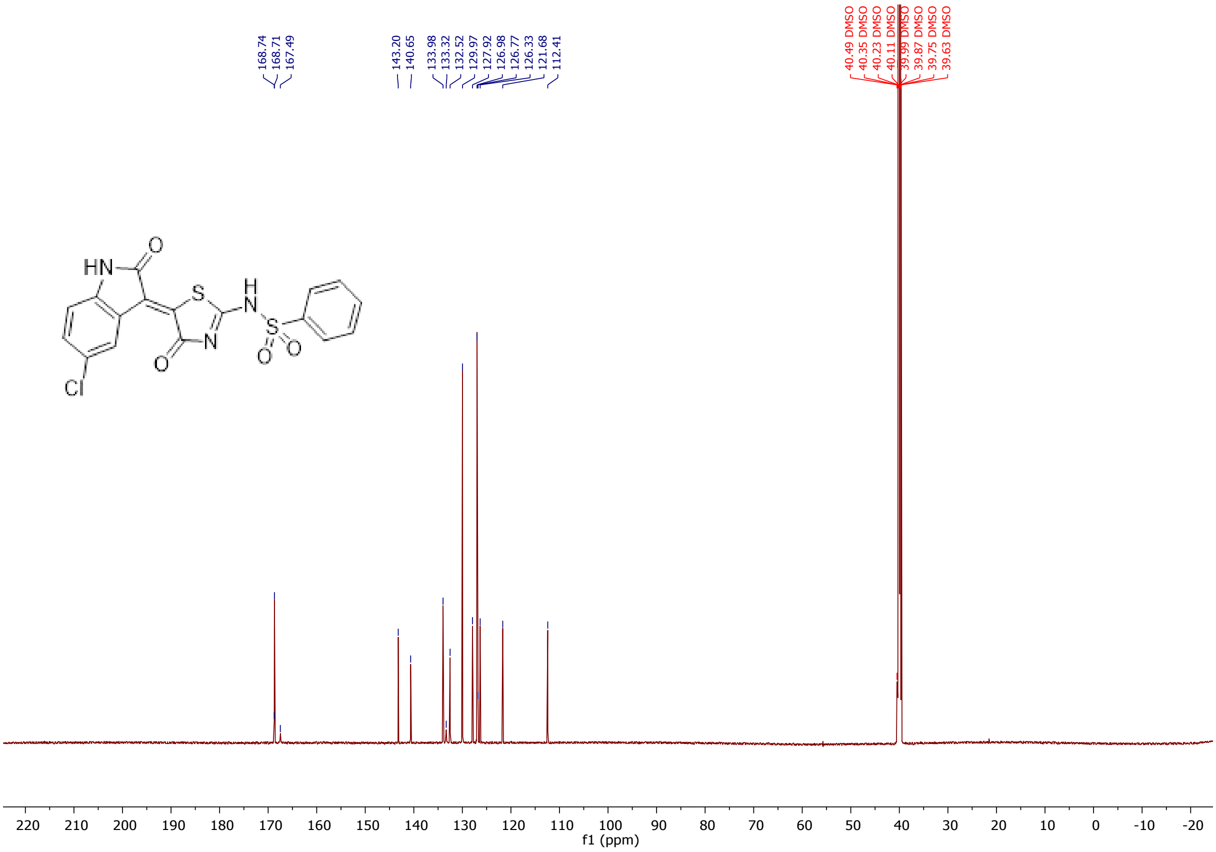

^{1}H NMR Spectrum of **21** (600 MHz, DMSO-d_6):

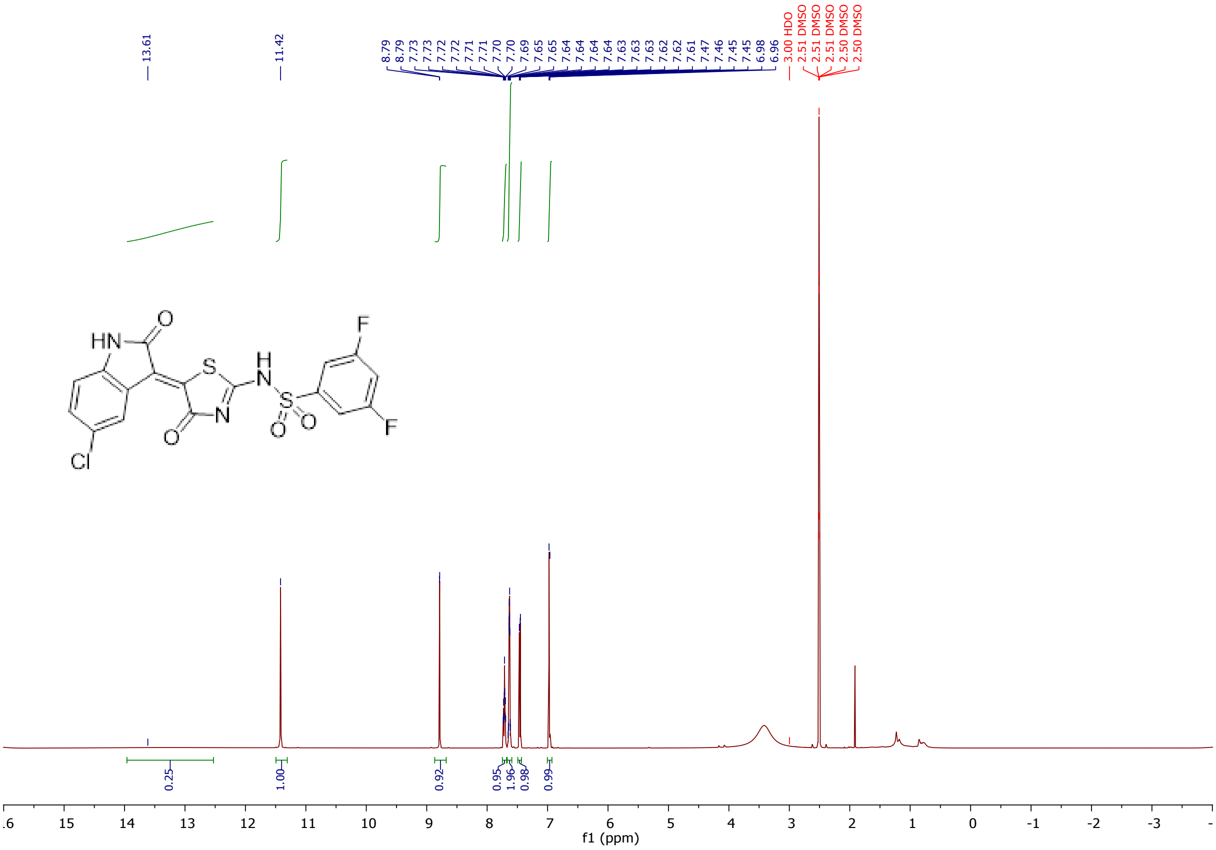

^{13}C NMR Spectrum of **21** (151 MHz, DMSO-d_6):

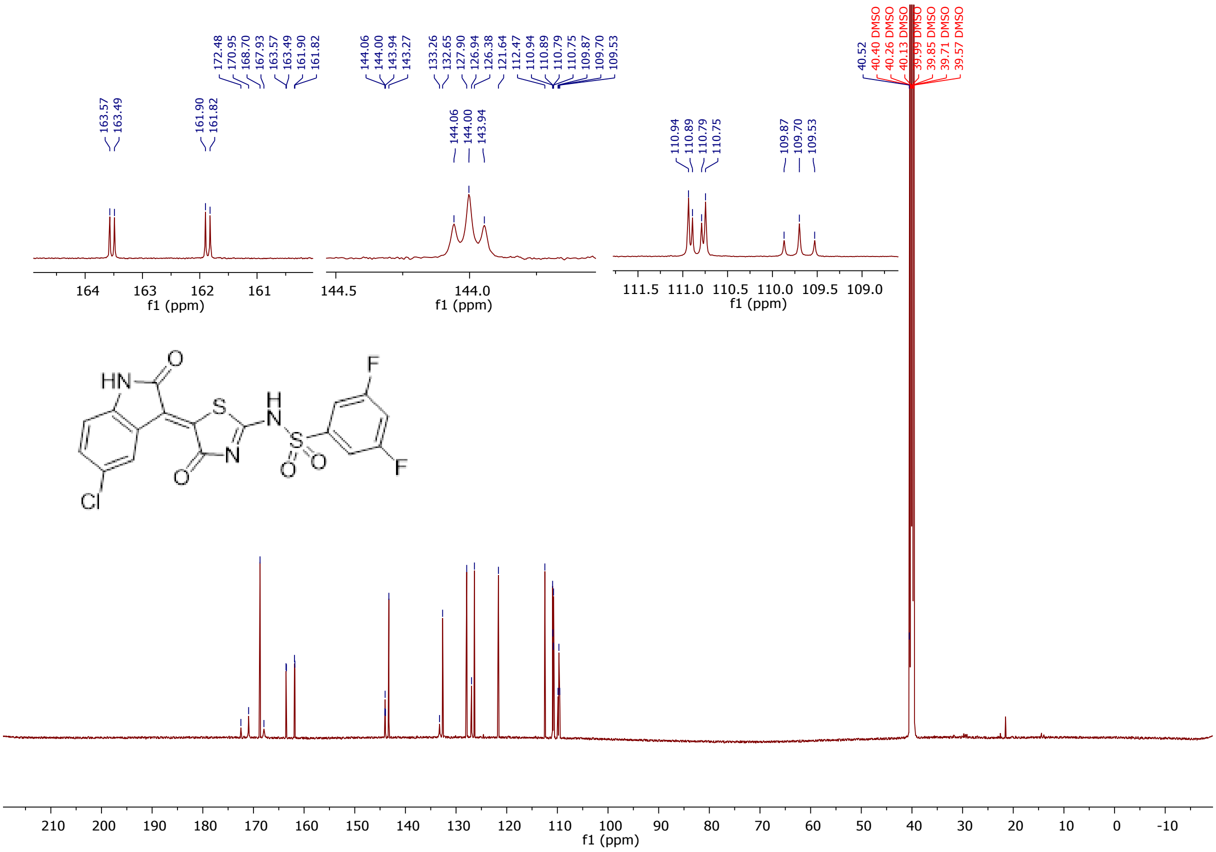

^{19}F NMR Spectrum of **21** (470 MHz, DMSO-d_6):

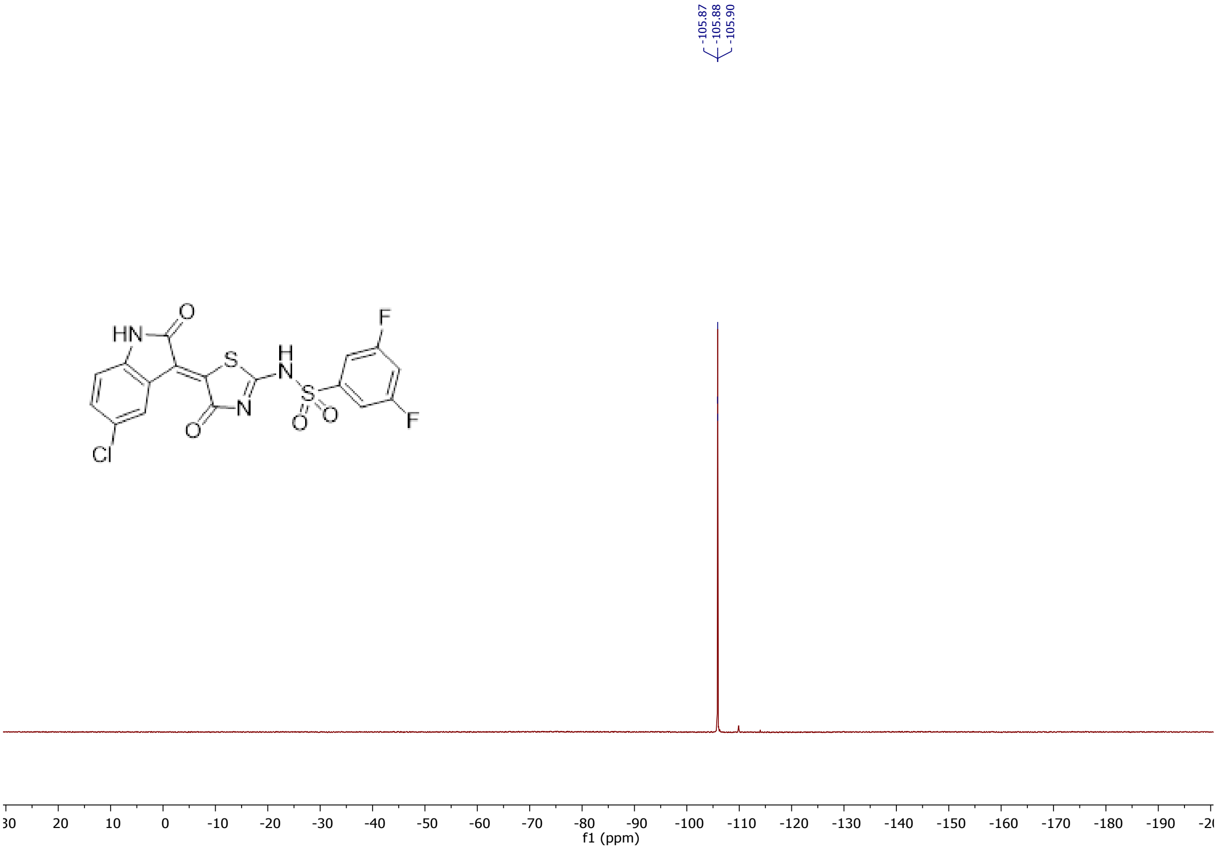

^{1}H NMR Spectrum of **22** (600 MHz, DMSO-d_6):

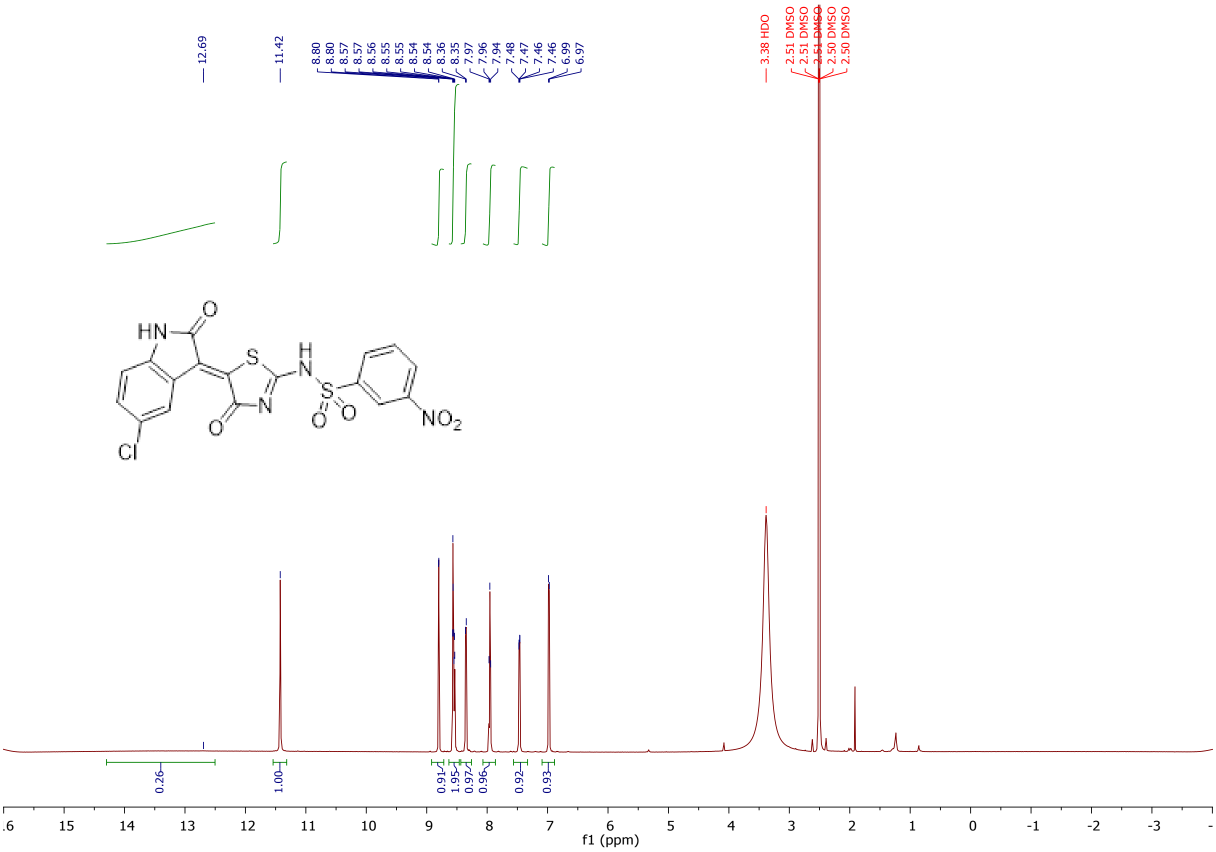

^{13}C NMR Spectrum of **22** (151 MHz, DMSO-d_6):

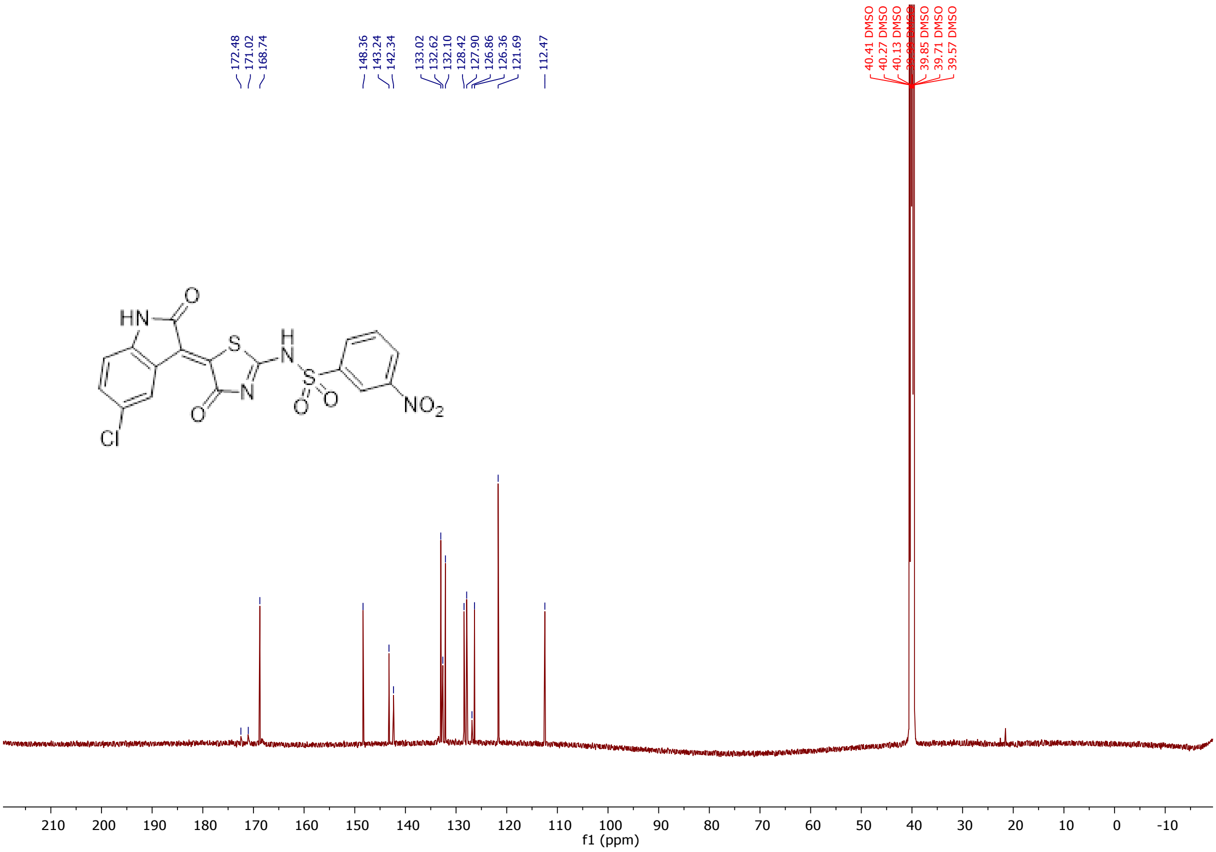

^{1}H NMR Spectrum of **23** (600 MHz, DMSO-d_6):

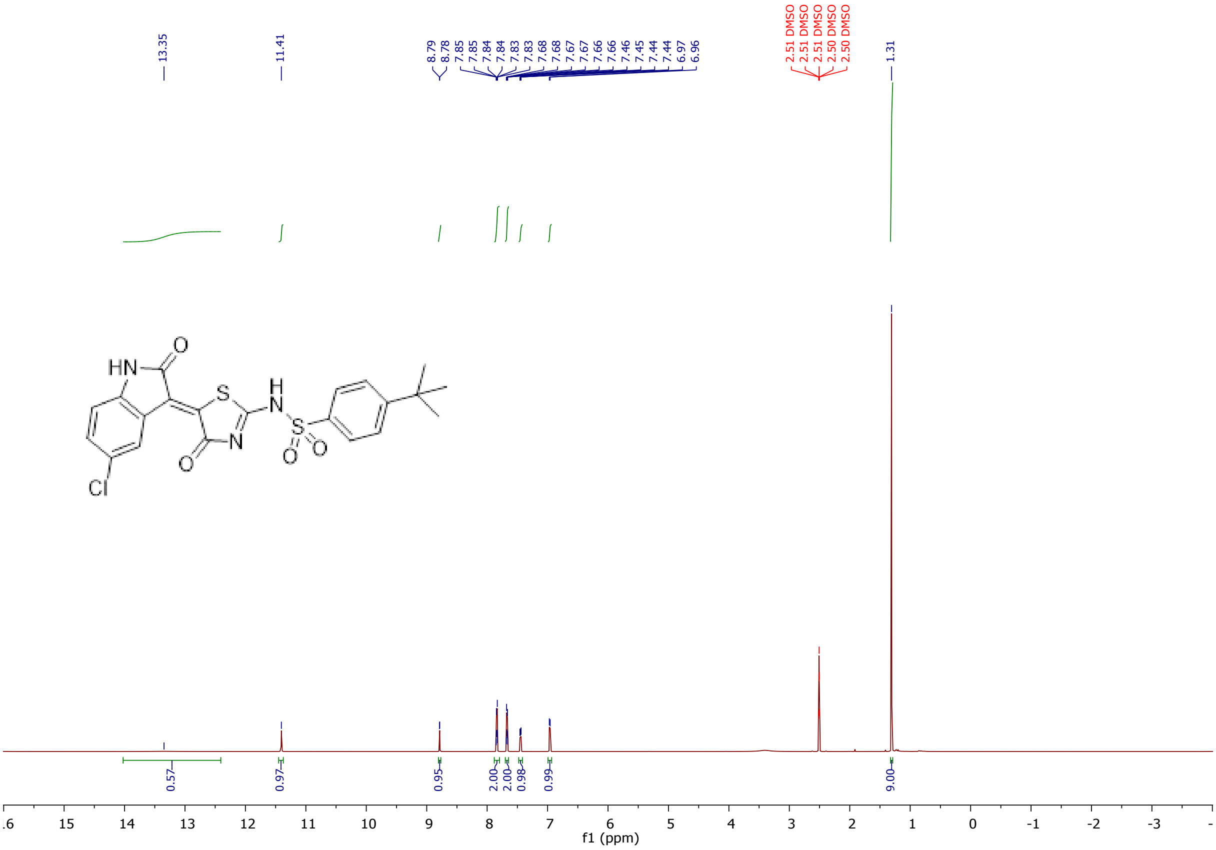

^{13}C NMR Spectrum of **23** (151 MHz, DMSO-d_6):

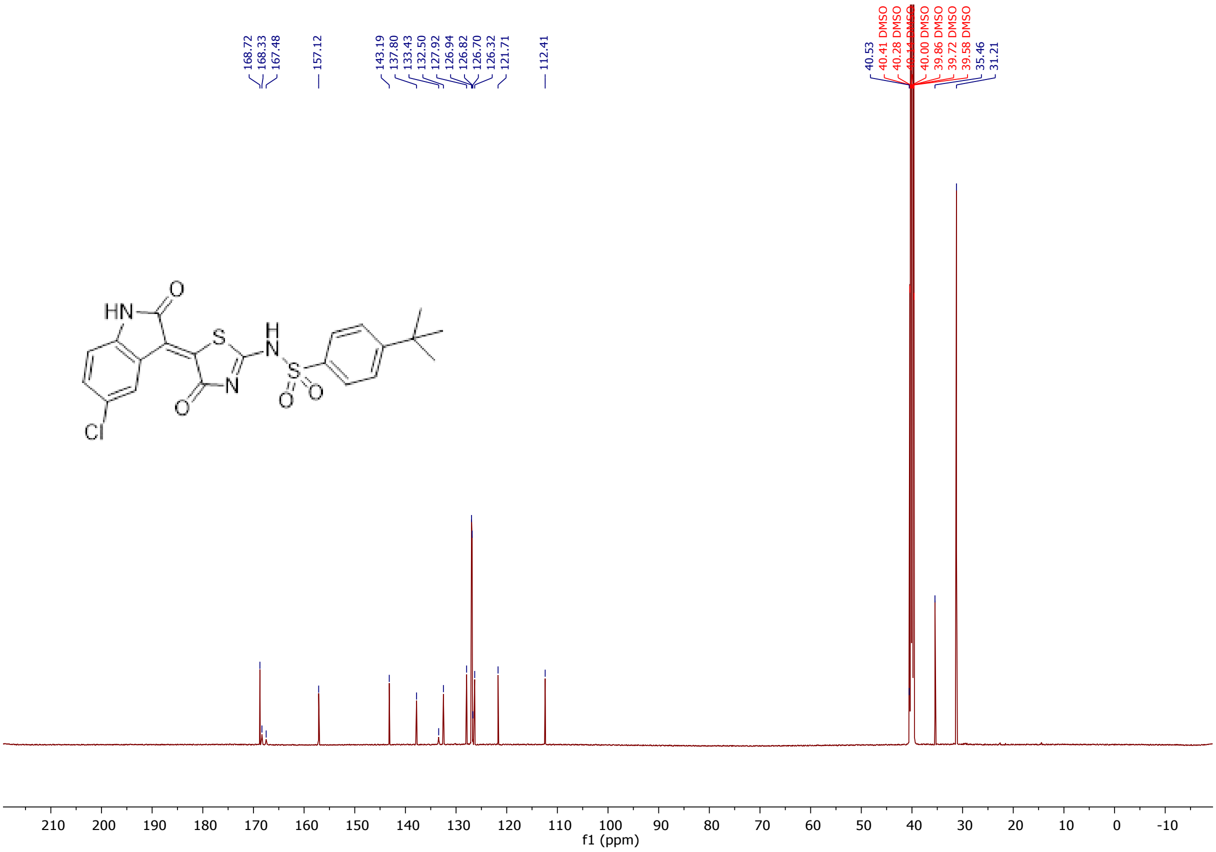

^{1}H NMR Spectrum of **24** (600 MHz, DMSO-d_6):

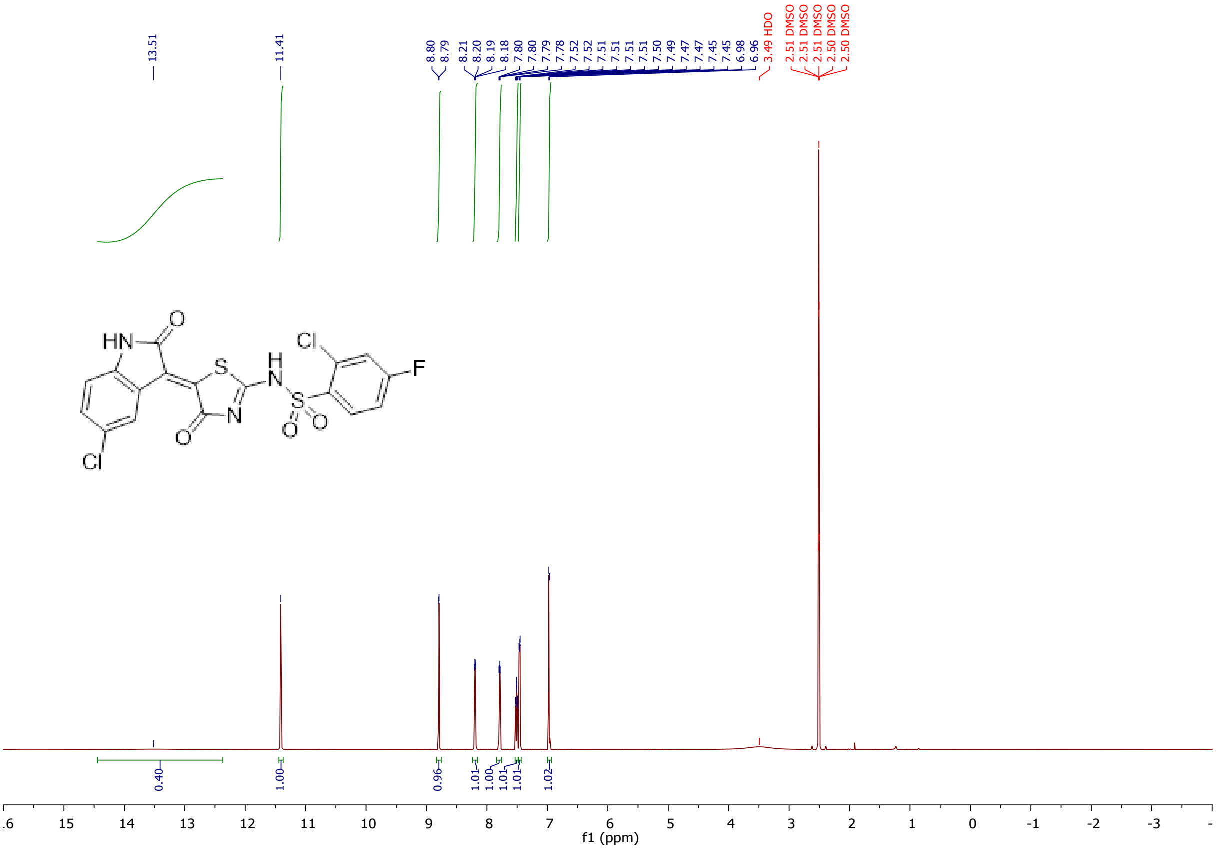

^{13}C NMR Spectrum of **24** (151 MHz, DMSO-d_6):

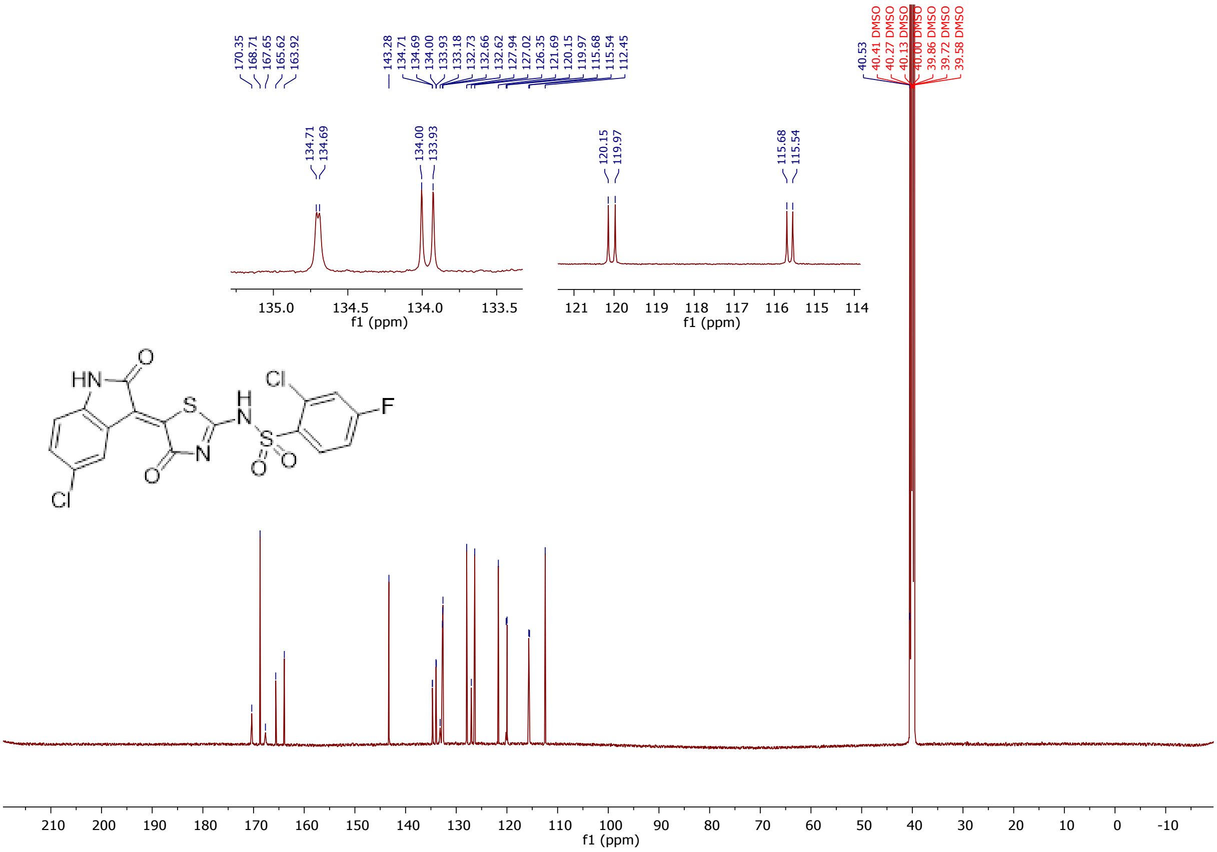

^{19}**F NMR** Spectrum of **24** (470 MHz, DMSO-d_6):

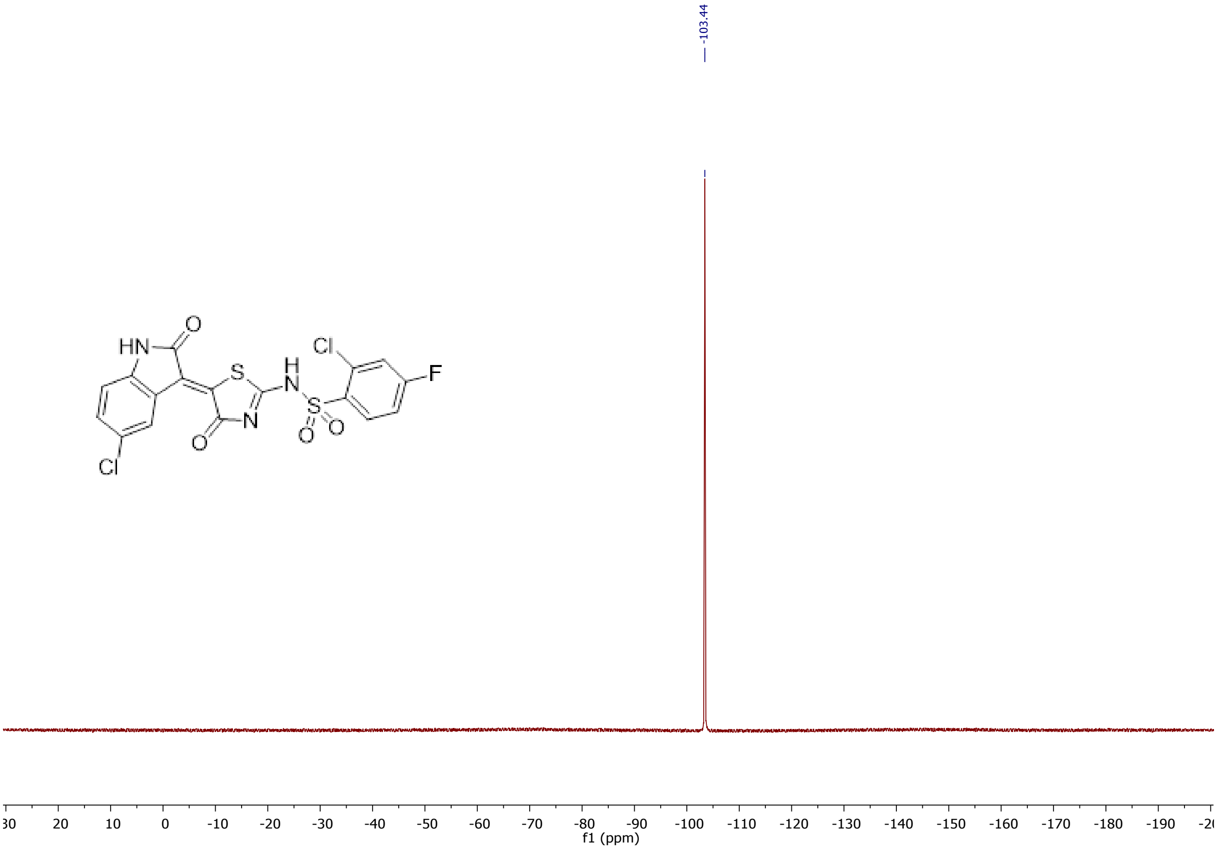

^{1}**H NMR** Spectrum of **25** (600 MHz, DMSO-d_6):

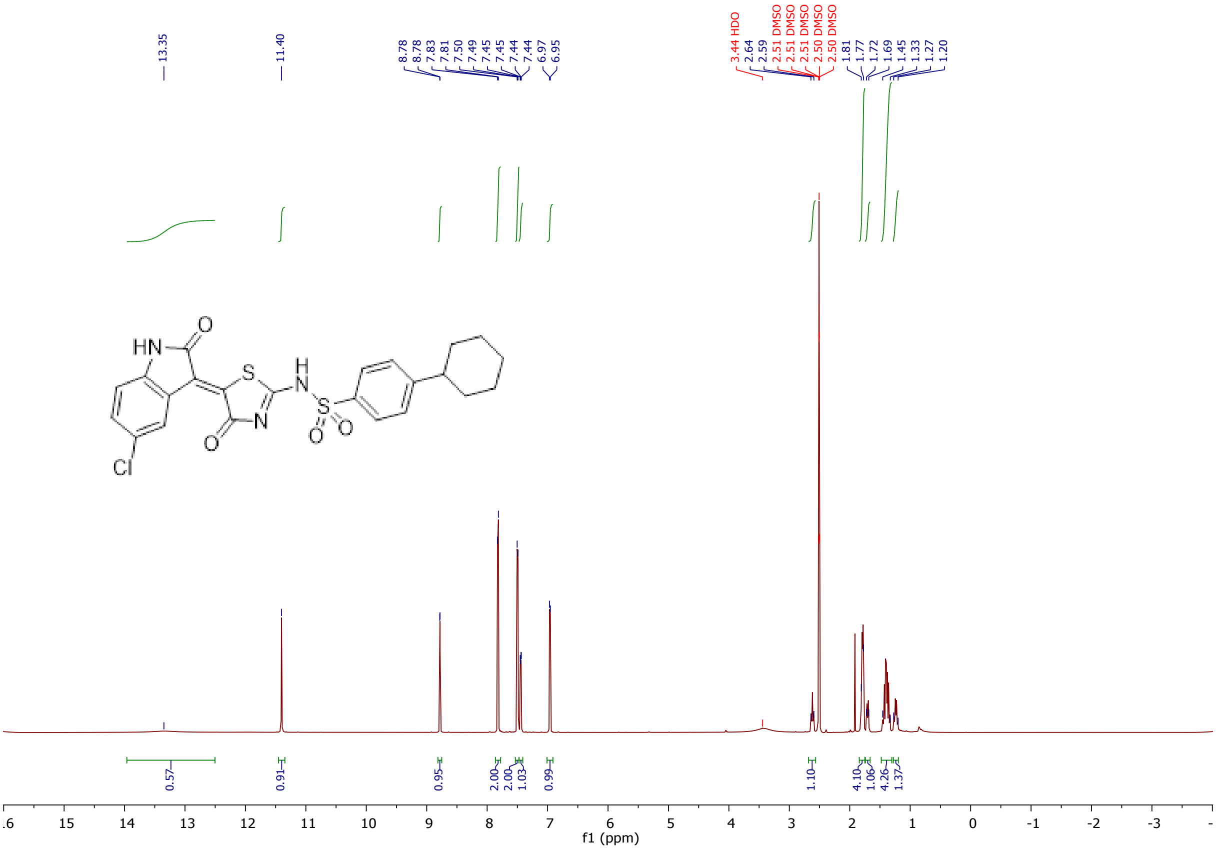

^{13}**C NMR** Spectrum of **25** (151 MHz, DMSO-d_6):

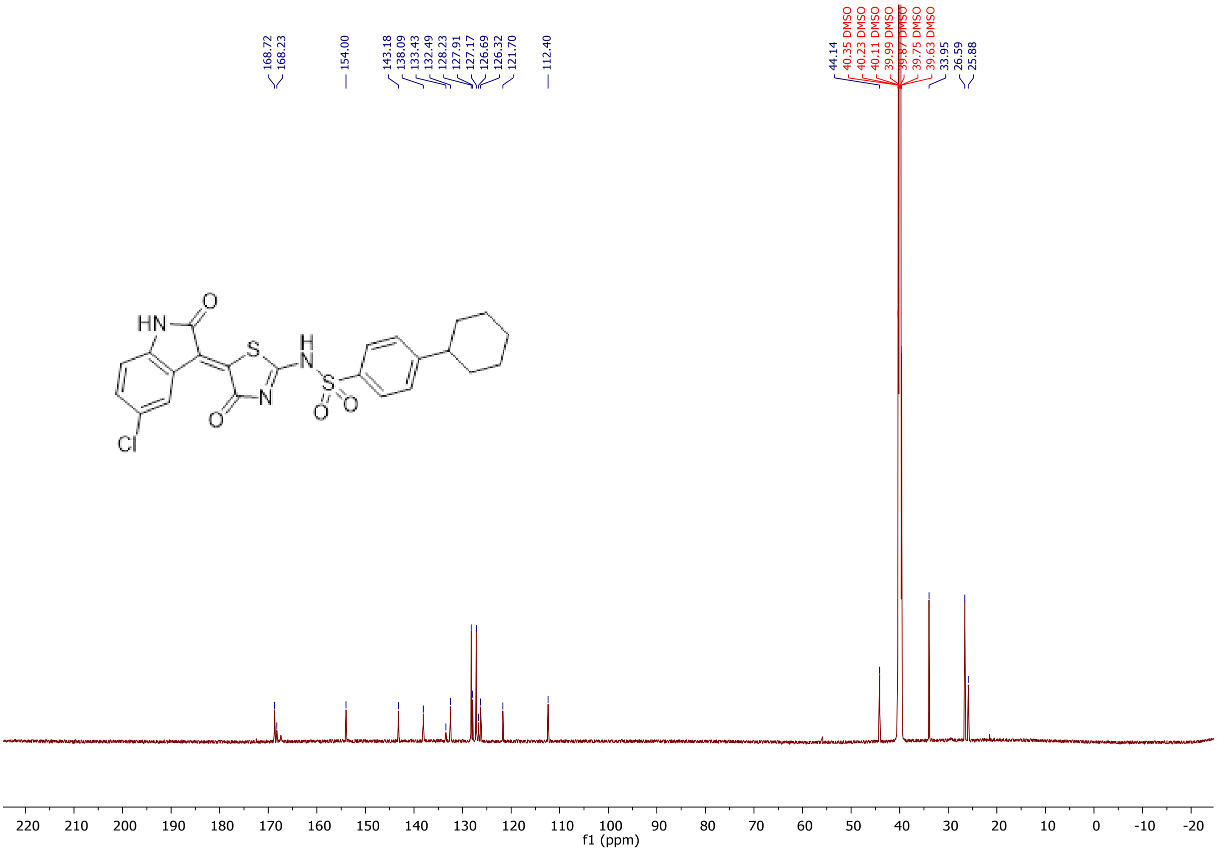

^{1}**H NMR** Spectrum of **26** (600 MHz, DMSO-d_6):

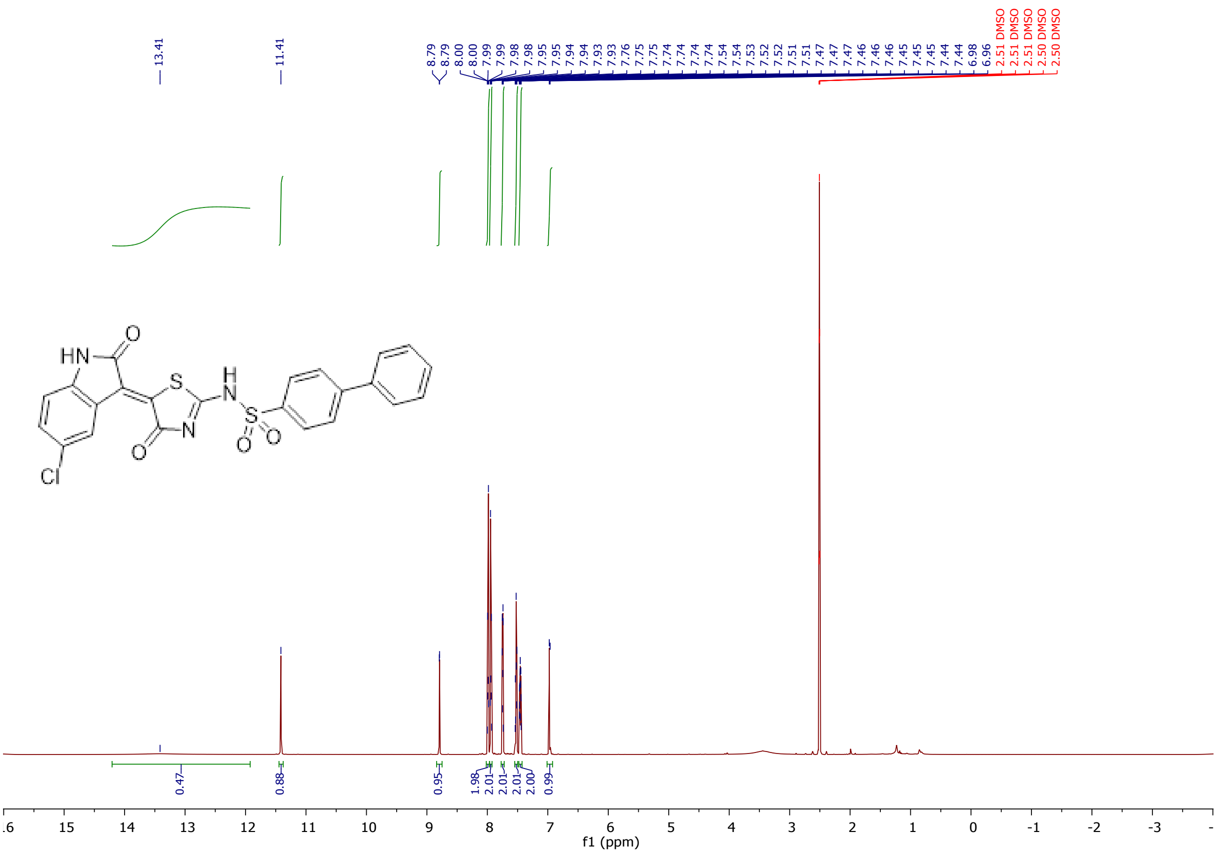

^{13}C NMR Spectrum of **26** (151 MHz, DMSO-d_6):

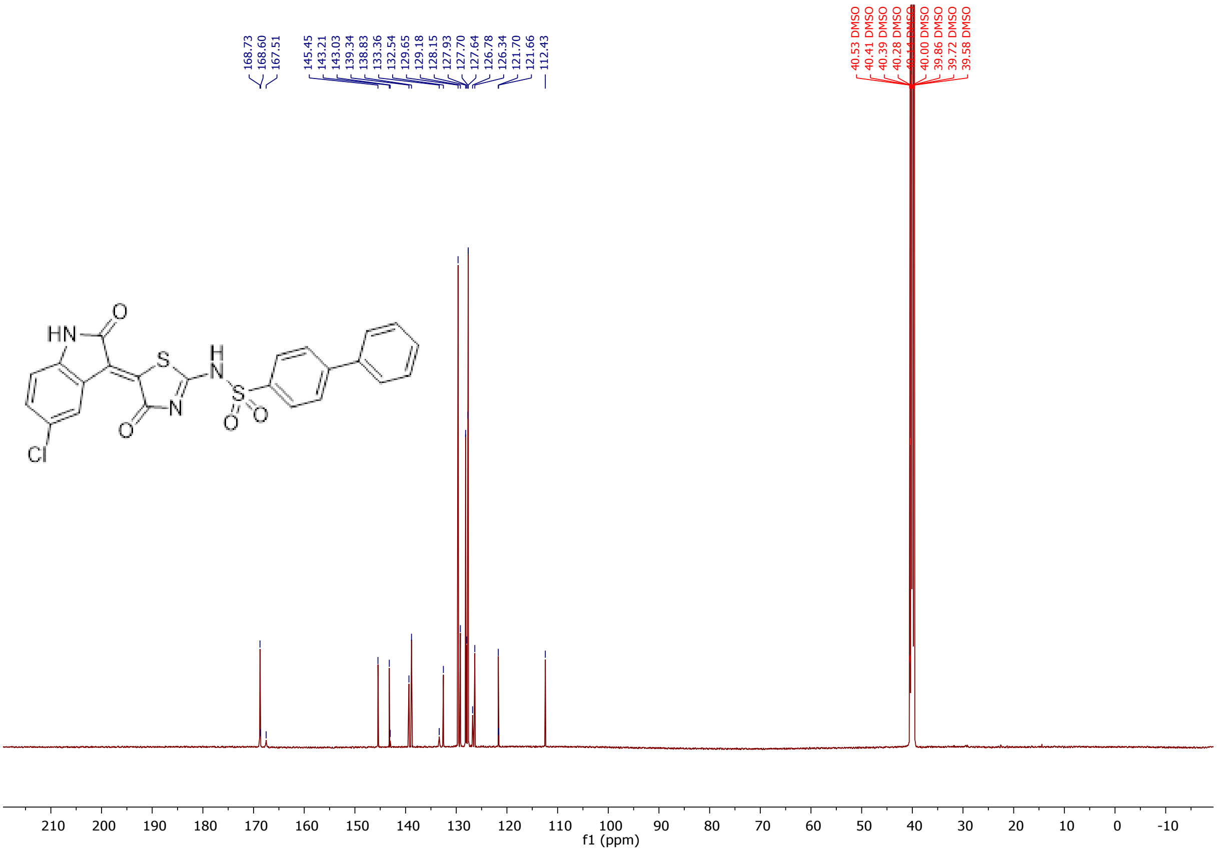

^{1}H NMR Spectrum of **27** (600 MHz, DMSO-d_6)(d.r. = 1:1):

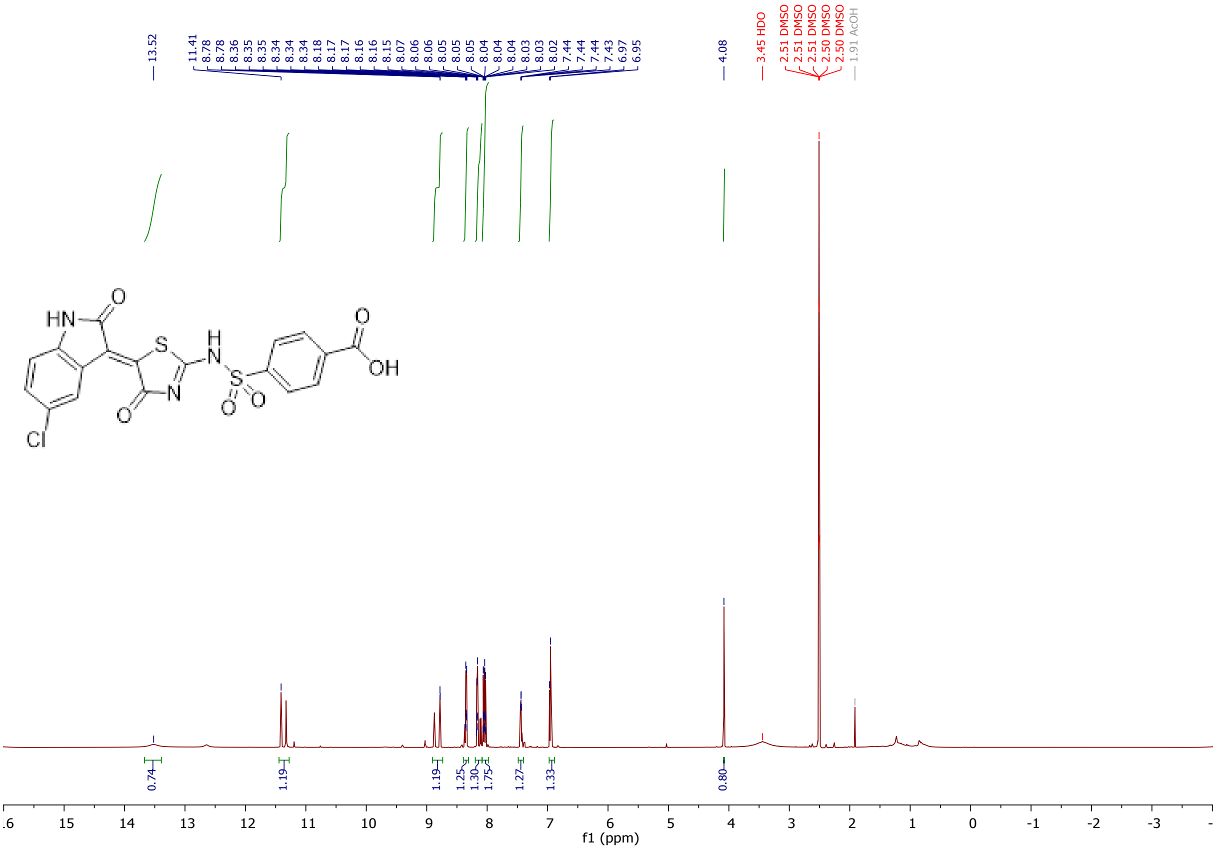

^{13}C NMR Spectrum of **27** (151 MHz, DMSO-d_6):

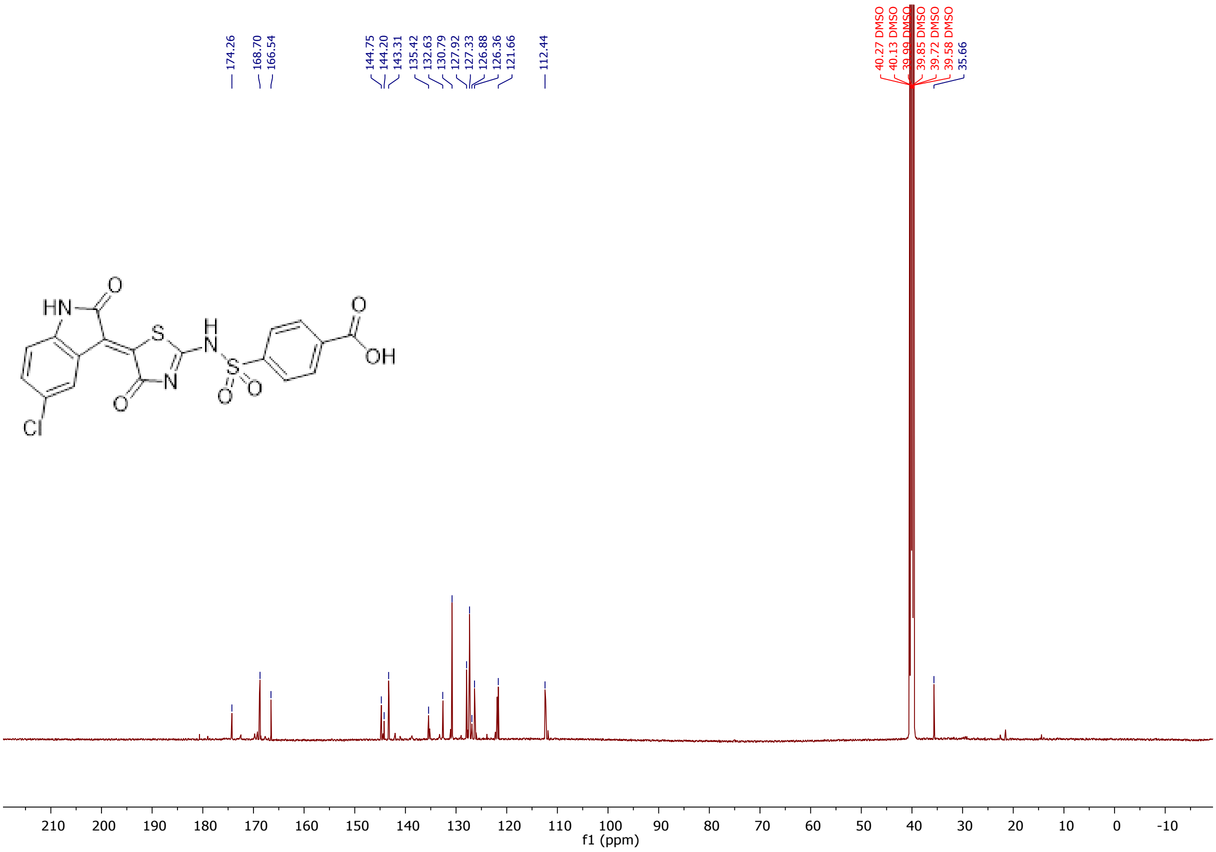

^{1}H NMR Spectrum of **28** (600 MHz, DMSO-d_6):

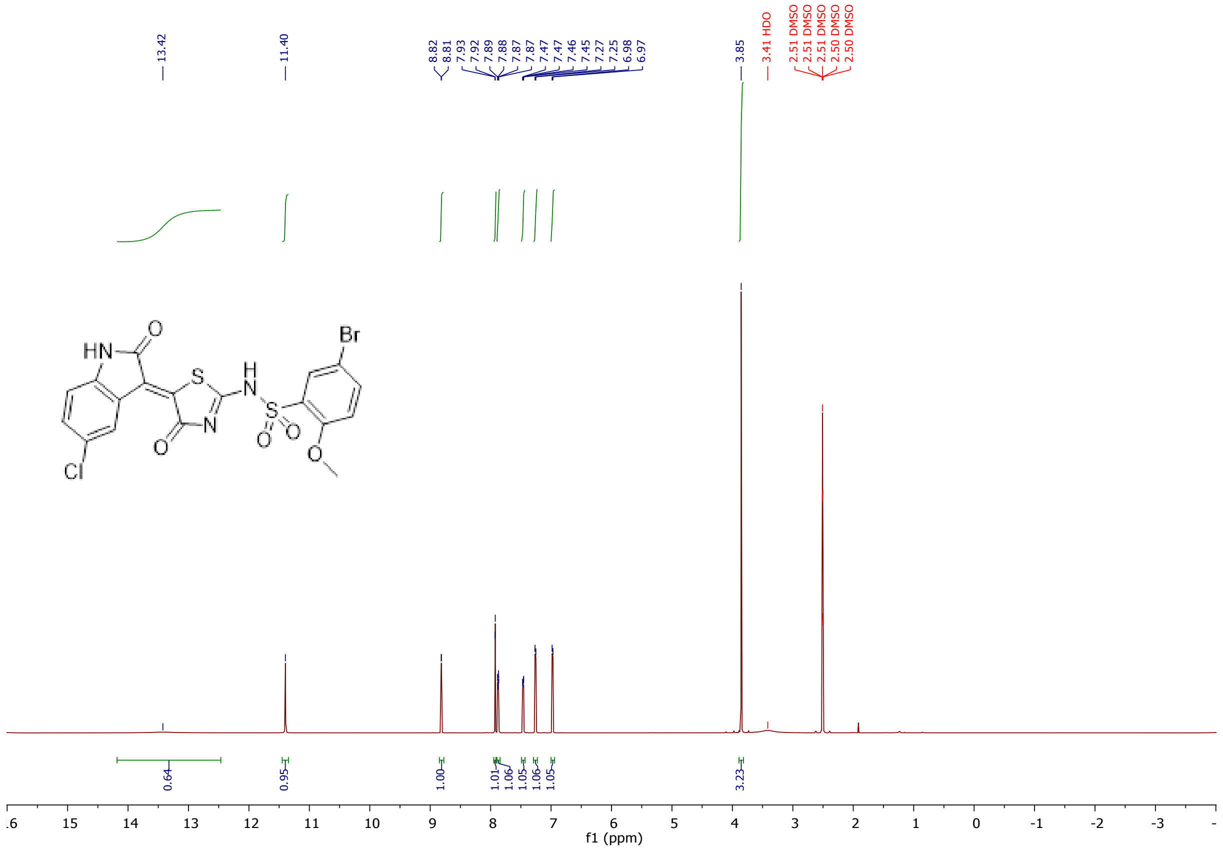

^{13}C NMR Spectrum of **28** (151 MHz, DMSO-d_6):

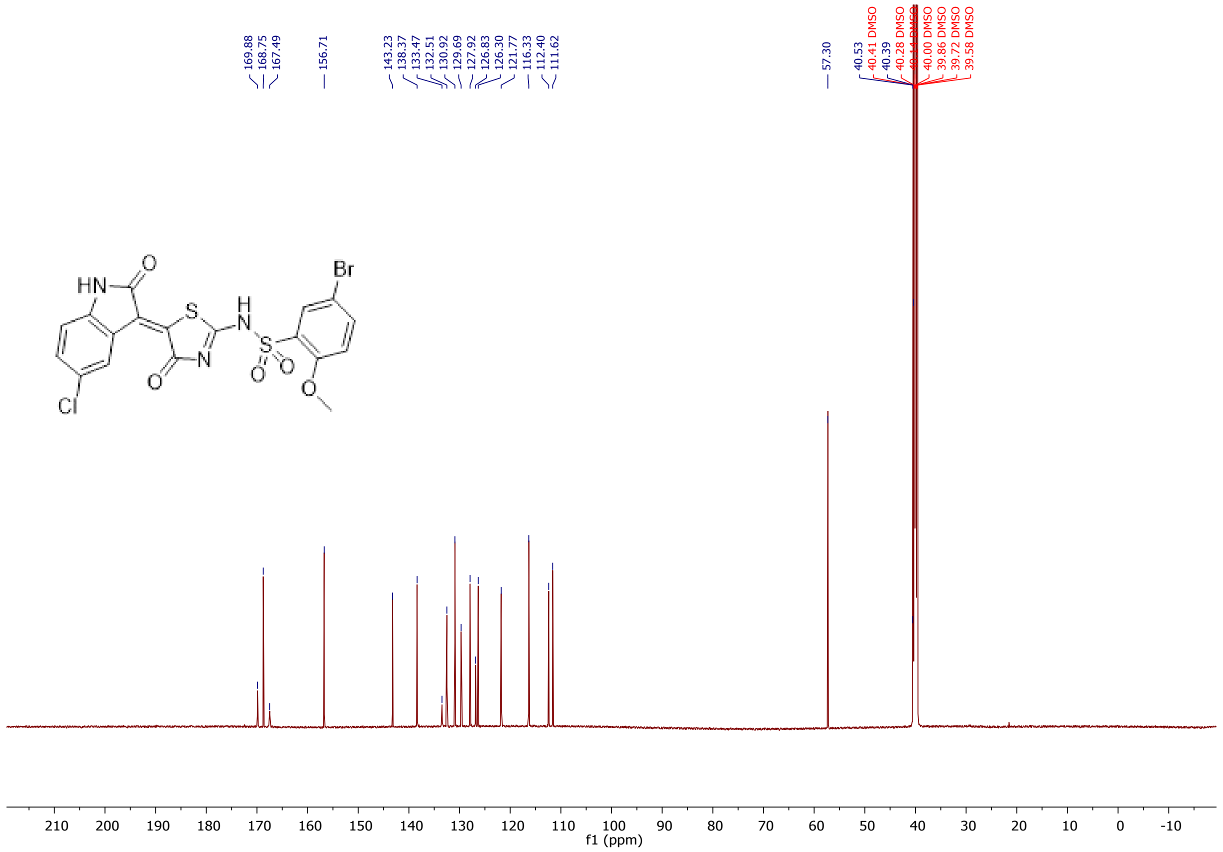

^{1}H NMR Spectrum of **29** (700 MHz, DMSO-d_6):

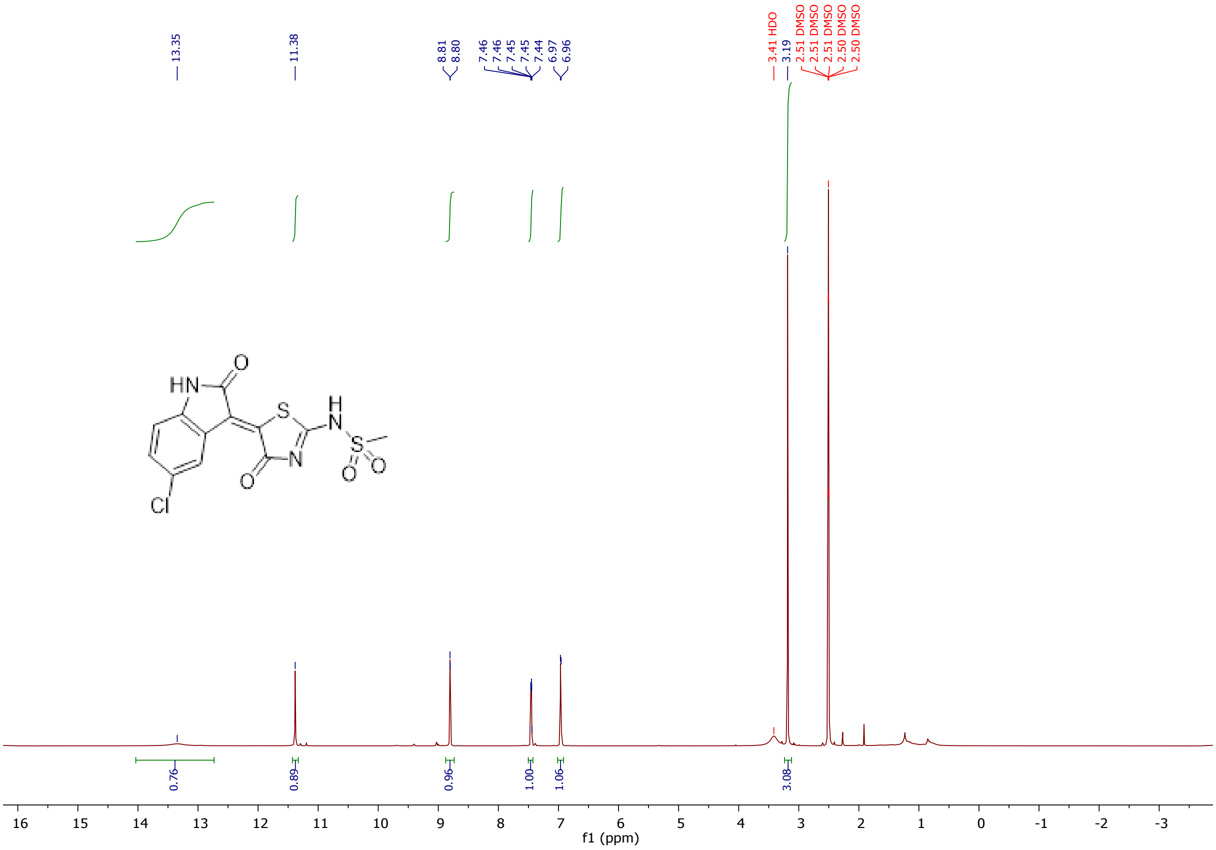

^{13}C NMR Spectrum of **29** (151 MHz, DMSO-d_6):

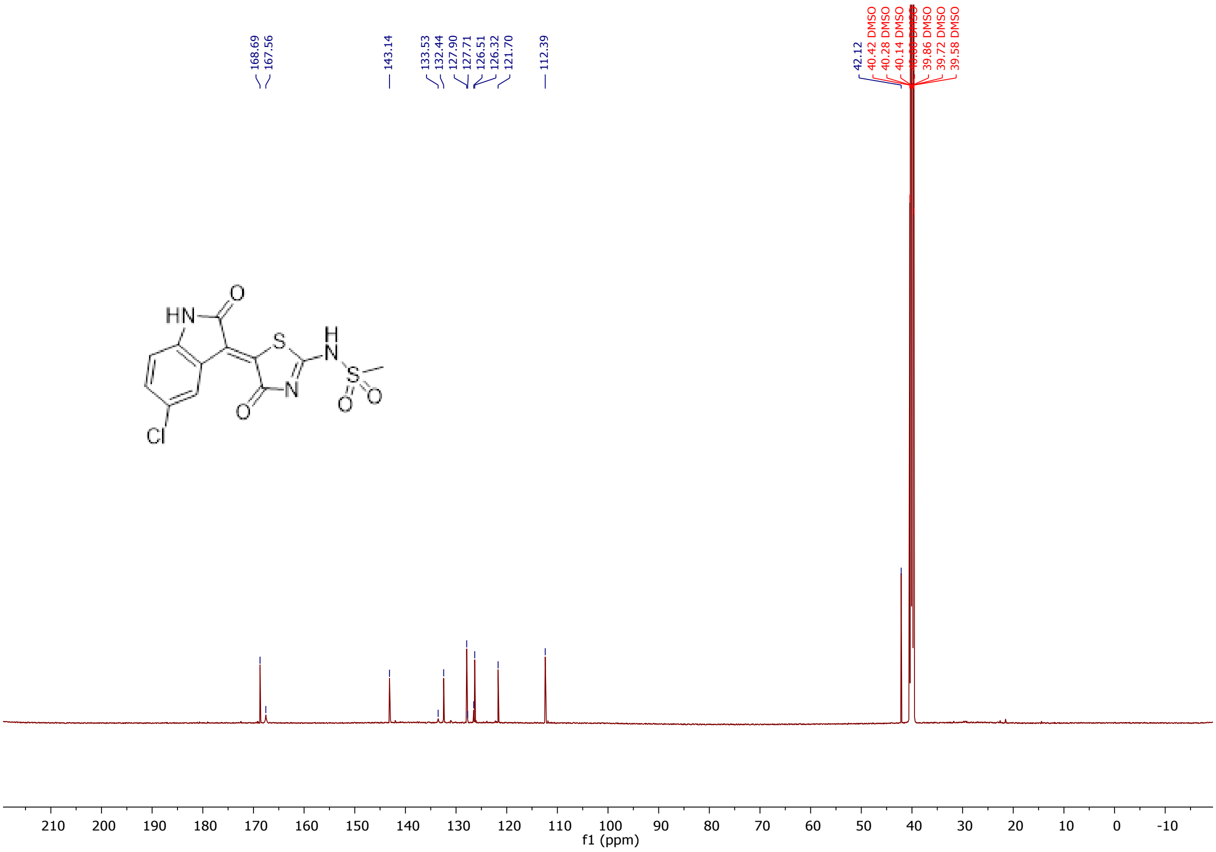

^{1}H NMR Spectrum of **30** (700 MHz, DMSO-d_6):

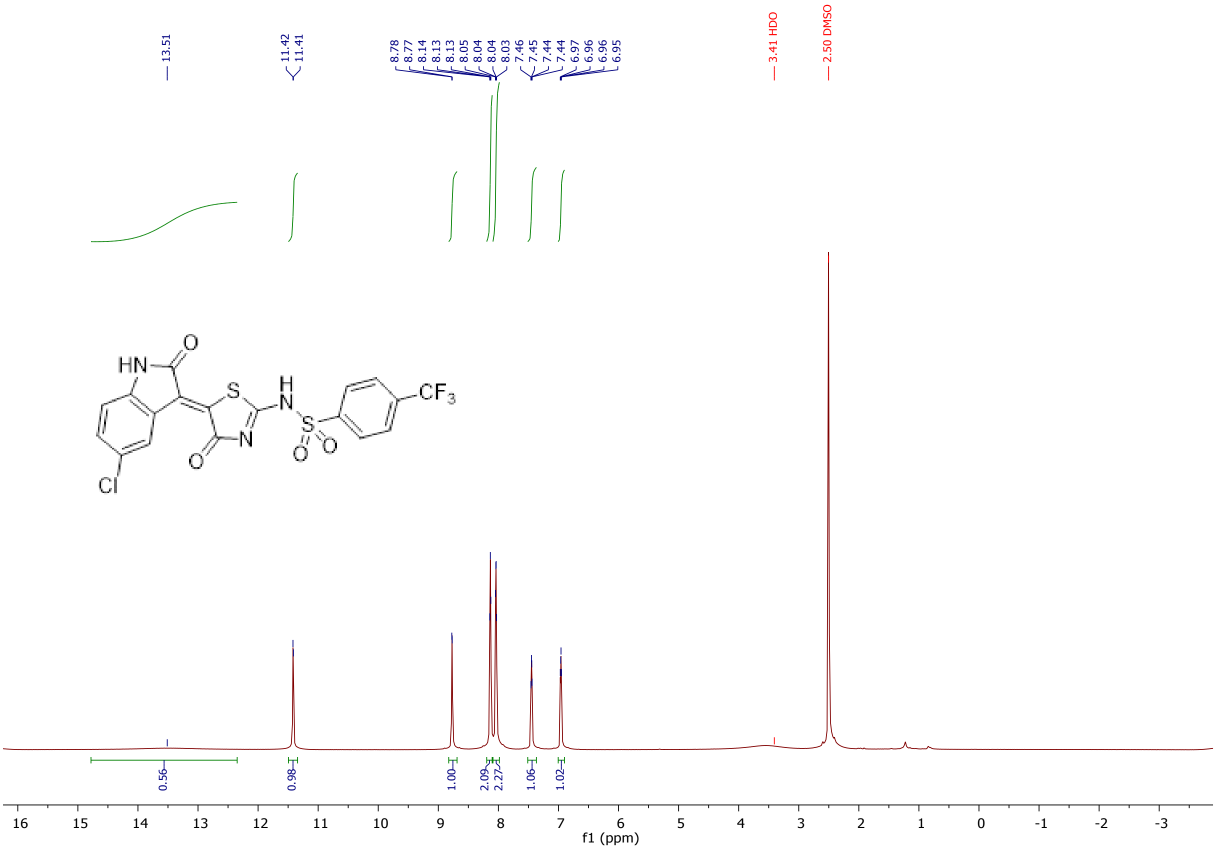

^{13}C **NMR** Spectrum of **30** (174 MHz, DMSO-d_6):

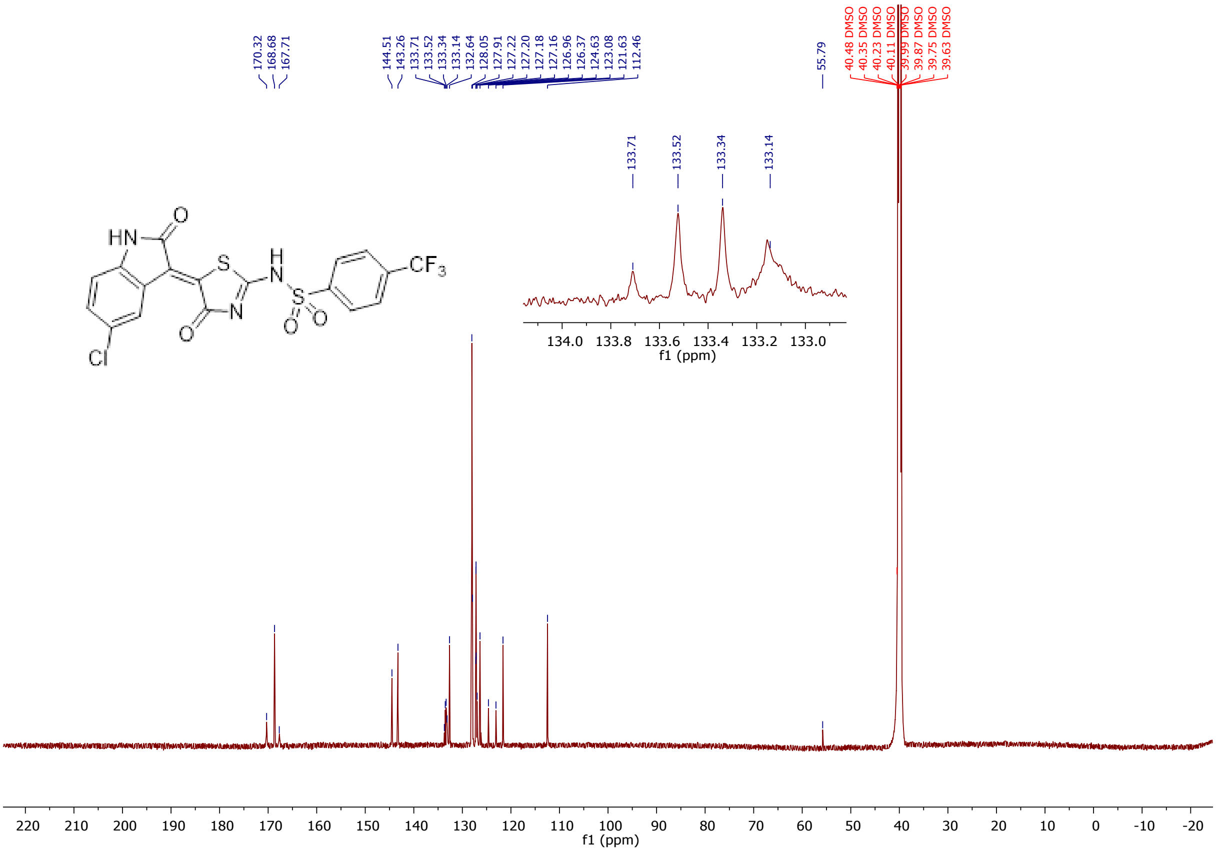

^{19}F **NMR** Spectrum of **30** (470 MHz, DMSO-d_6):

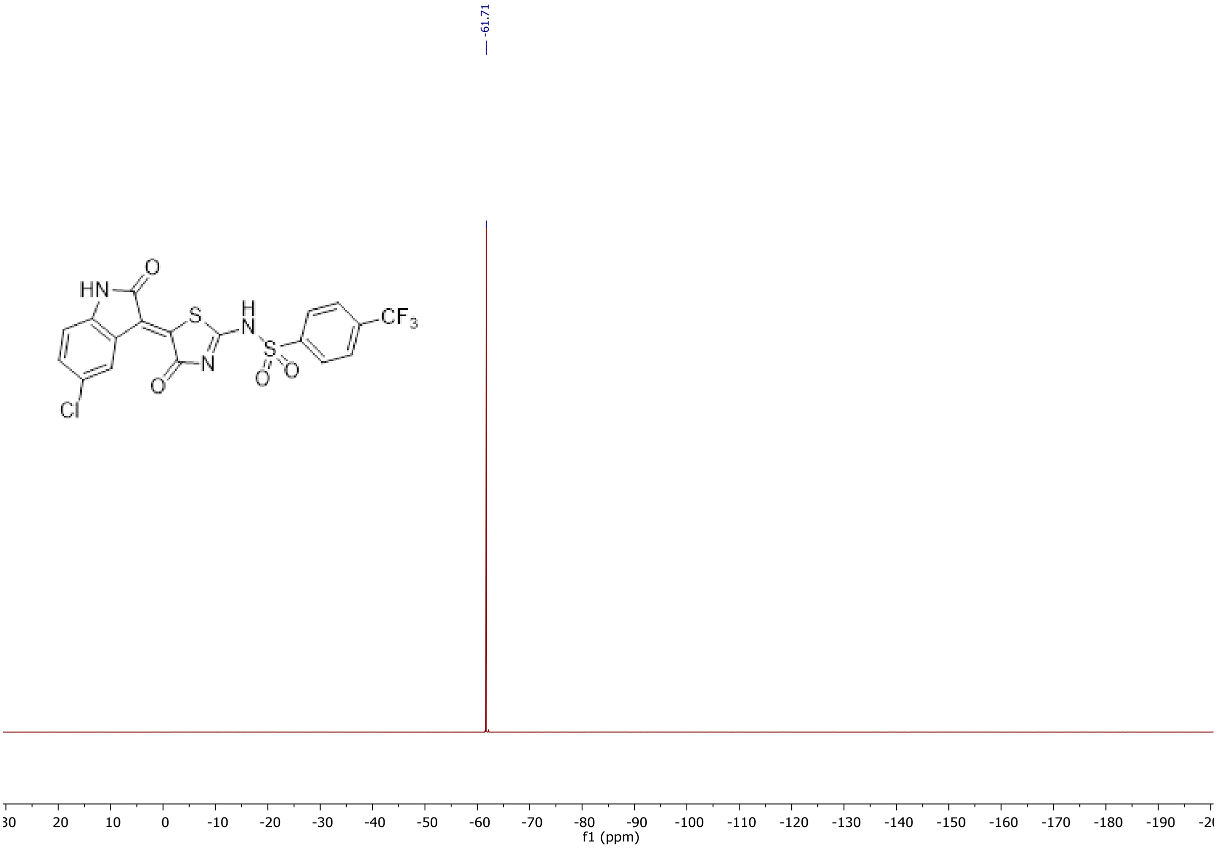

^{1}**H NMR** Spectrum of **31** (600 MHz, DMSO-d_6):

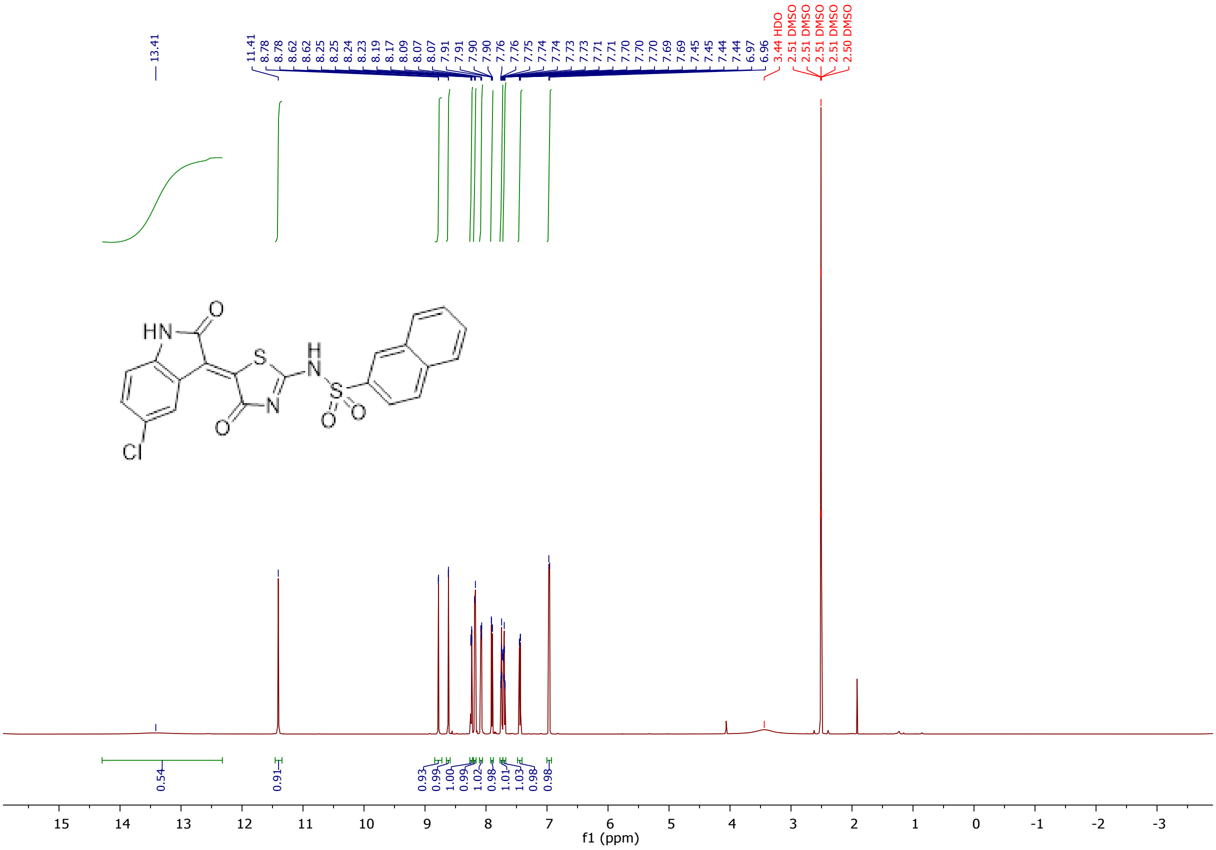

^{13}**C NMR** Spectrum of **31** (151 MHz, DMSO-d_6):

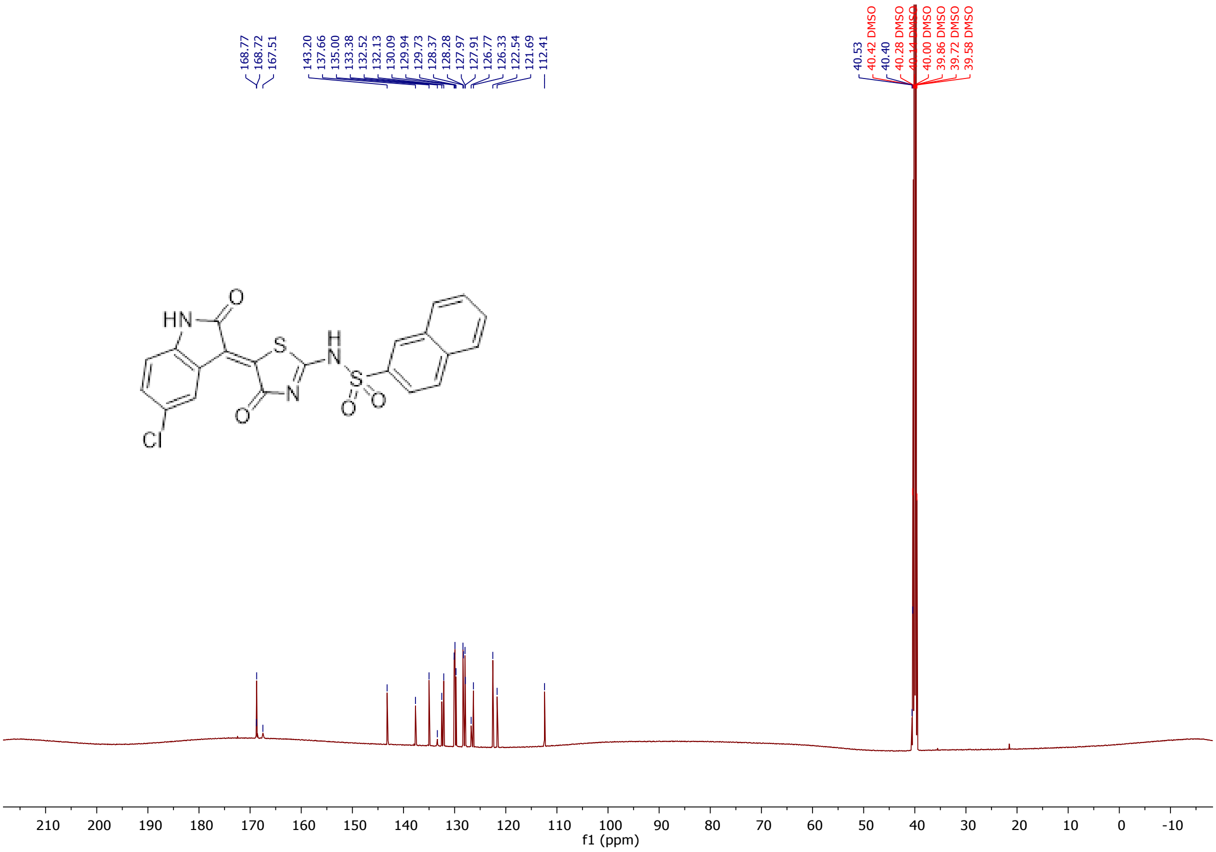

^{1}H NMR Spectrum of **32** (600 MHz, DMSO-d_6):

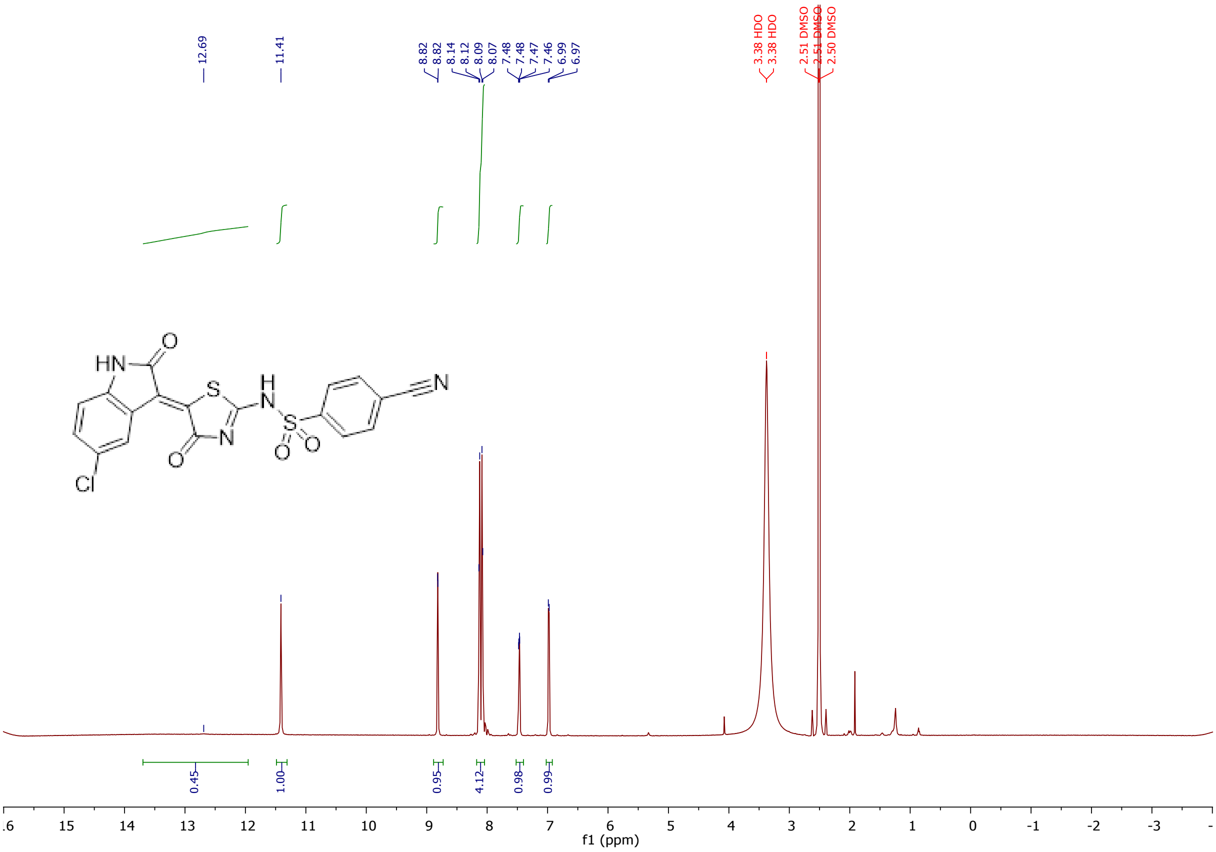

^{13}C NMR Spectrum of **32** (151 MHz, DMSO-d_6):

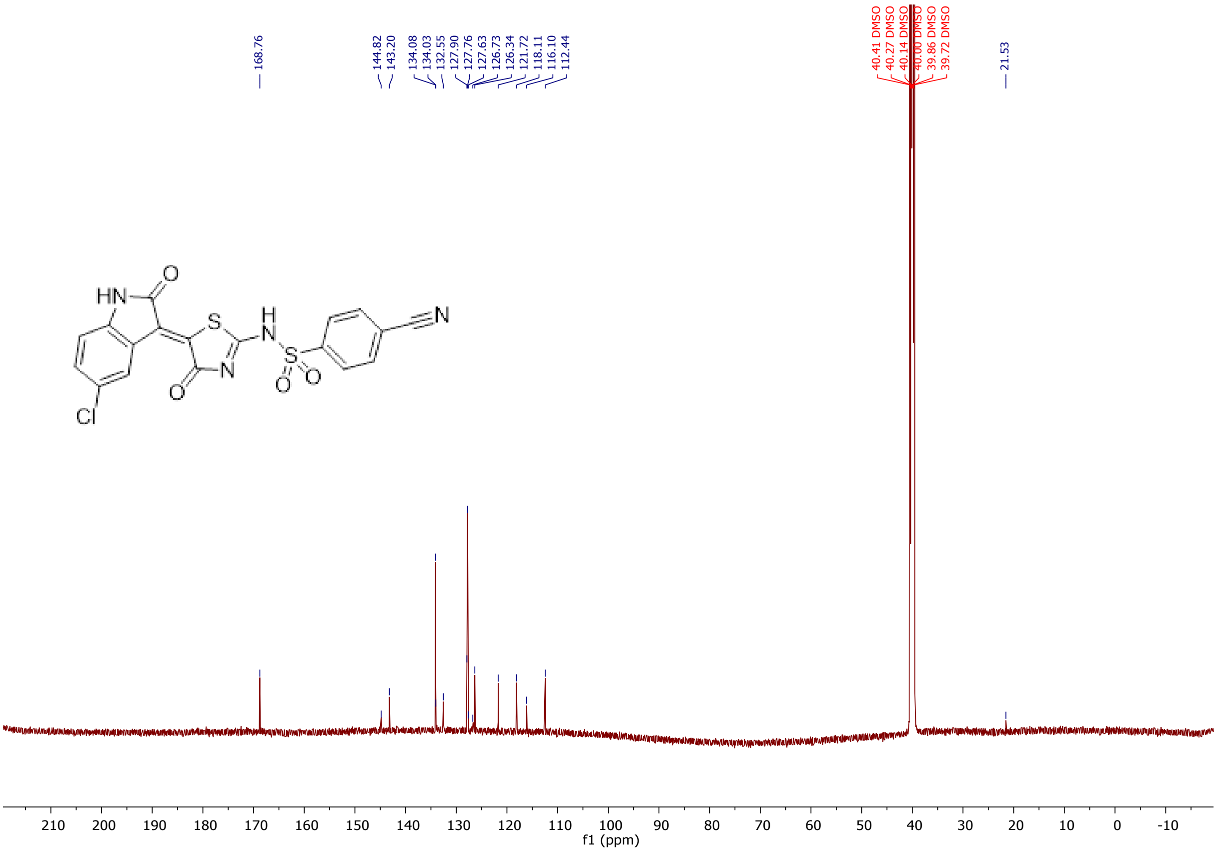

^{1}H NMR Spectrum of **33** (700 MHz, DMSO-d_6):

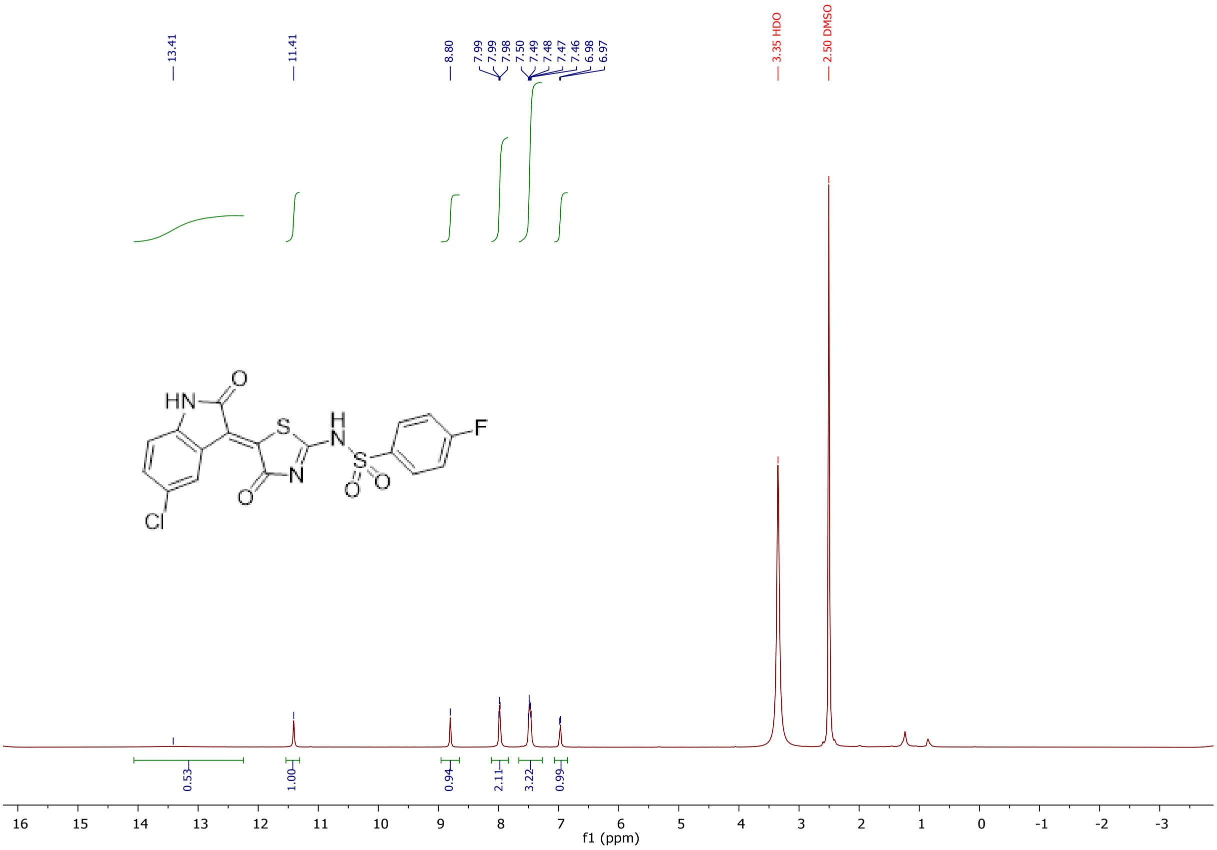

^{13}C NMR Spectrum of **33** (174 MHz, DMSO-d_6):

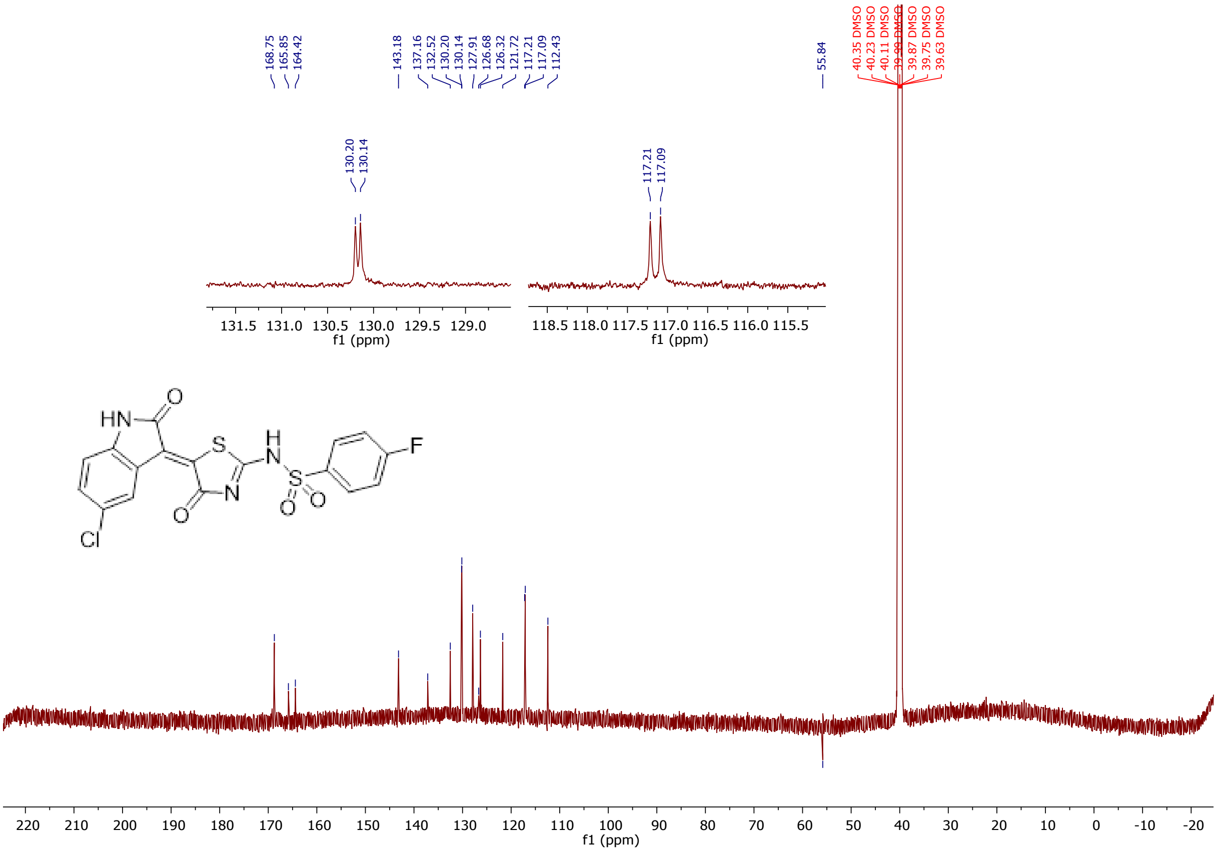

^{19}F NMR Spectrum of **33** (470 MHz, DMSO-d_6):

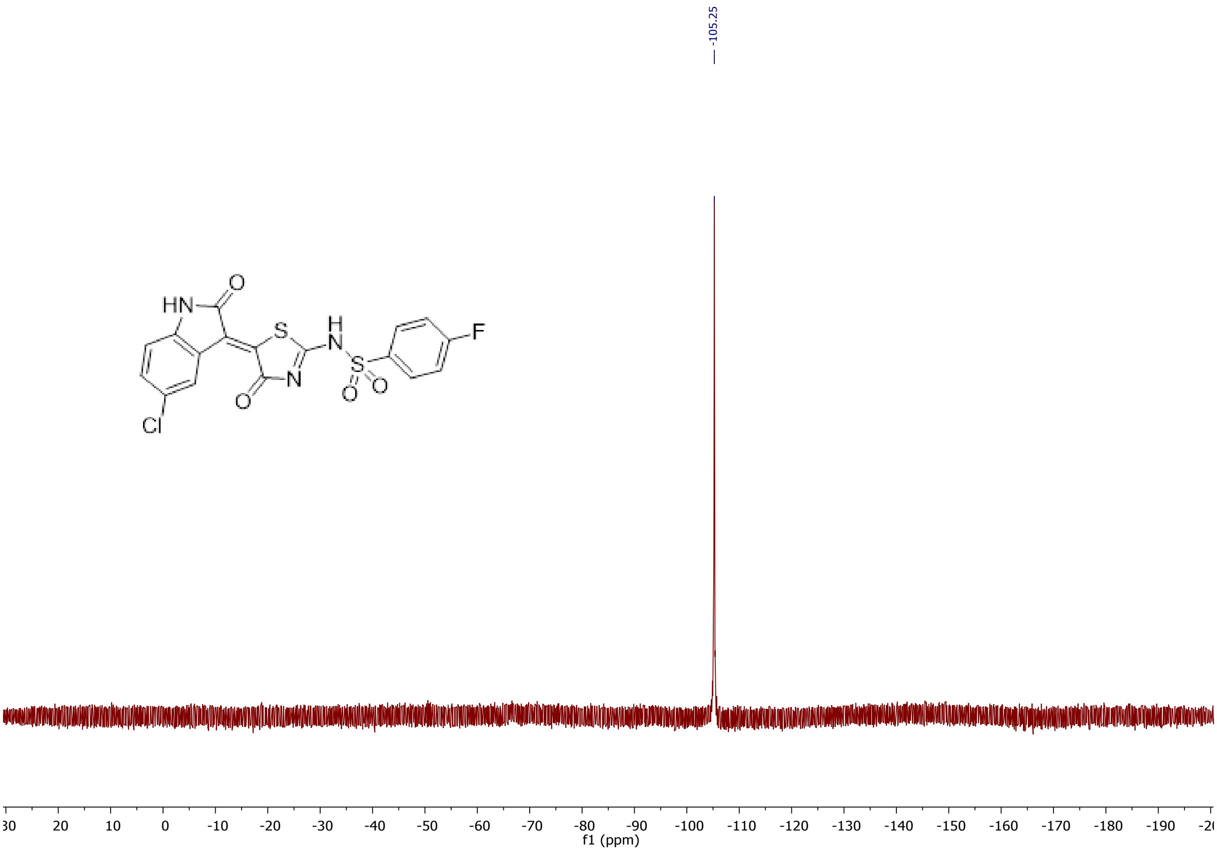

^{1}H NMR Spectrum of **34** (600 MHz, DMSO-d_6):

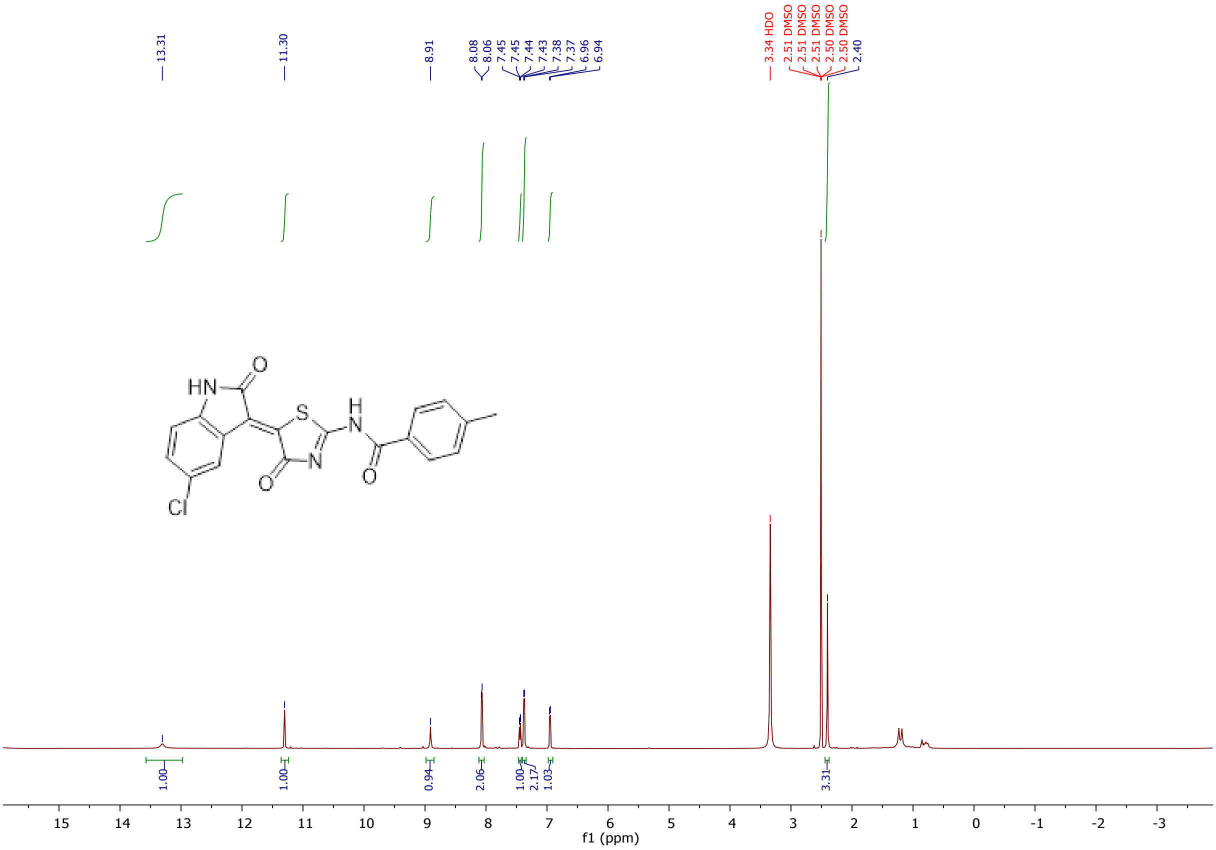

^{13}**C NMR** Spectrum of **34** (151 MHz, DMSO-d_6):

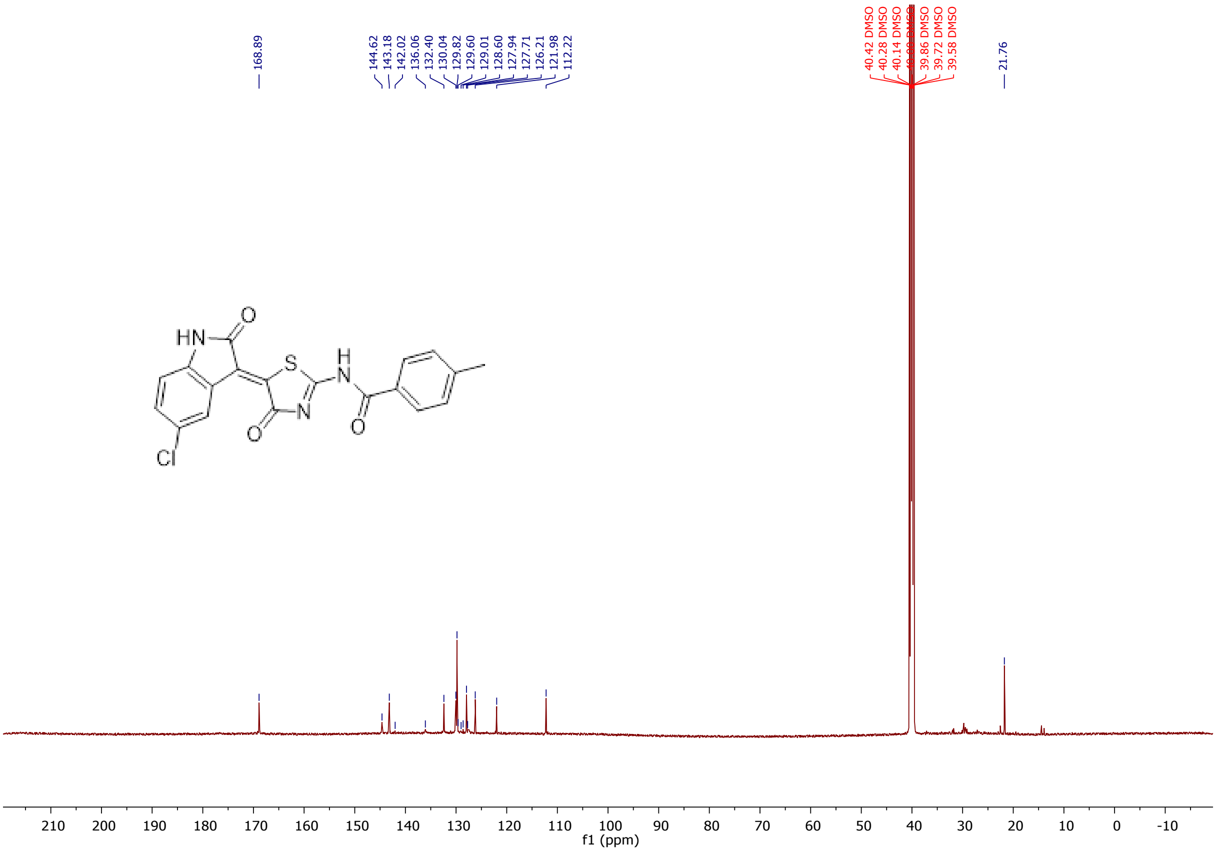

^{1}**H NMR** Spectrum of **35** (600 MHz, DMSO-d_6):

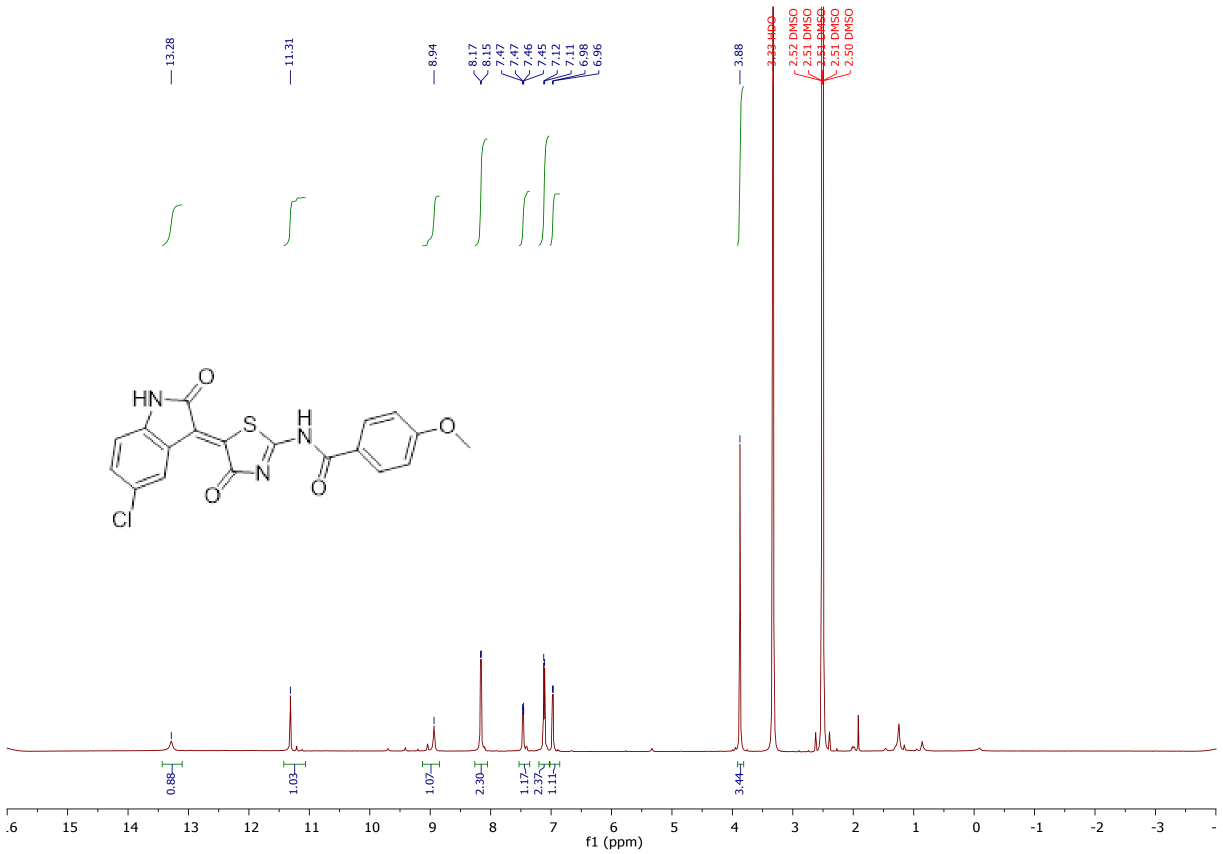

^{13}**C NMR** Spectrum of **35** (151 MHz, DMSO-d_6):

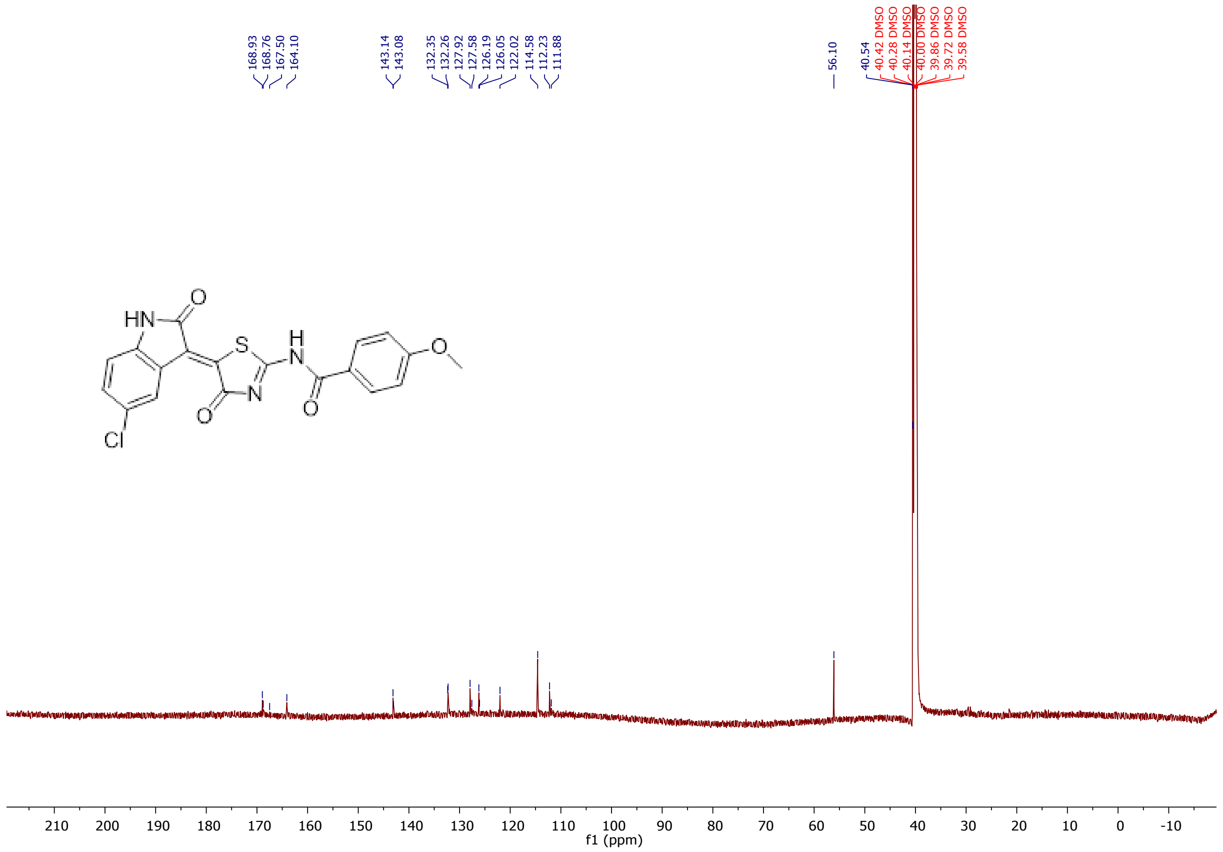

^{1}**H NMR** Spectrum of **36** (600 MHz, DMSO-d_6):

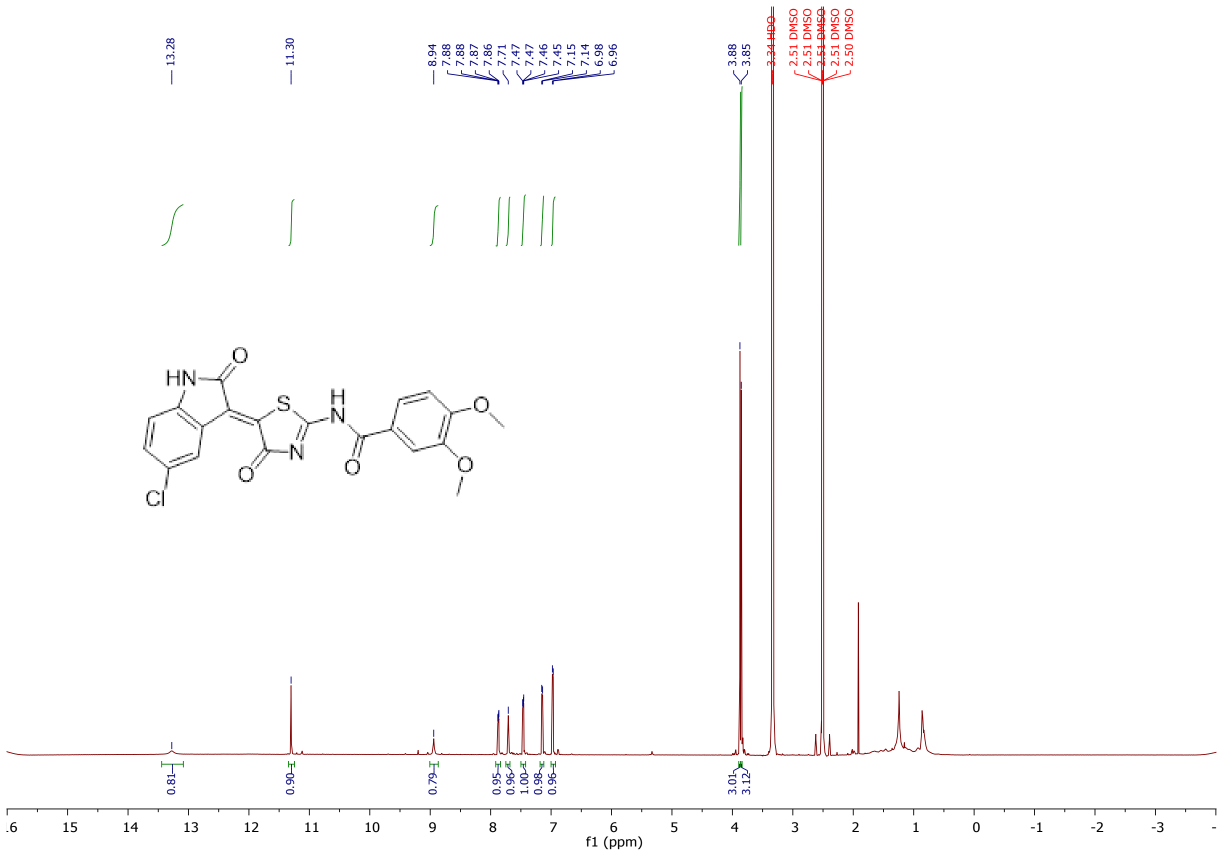

^{13}C NMR Spectrum of **36** (151 MHz, DMSO-d_6):

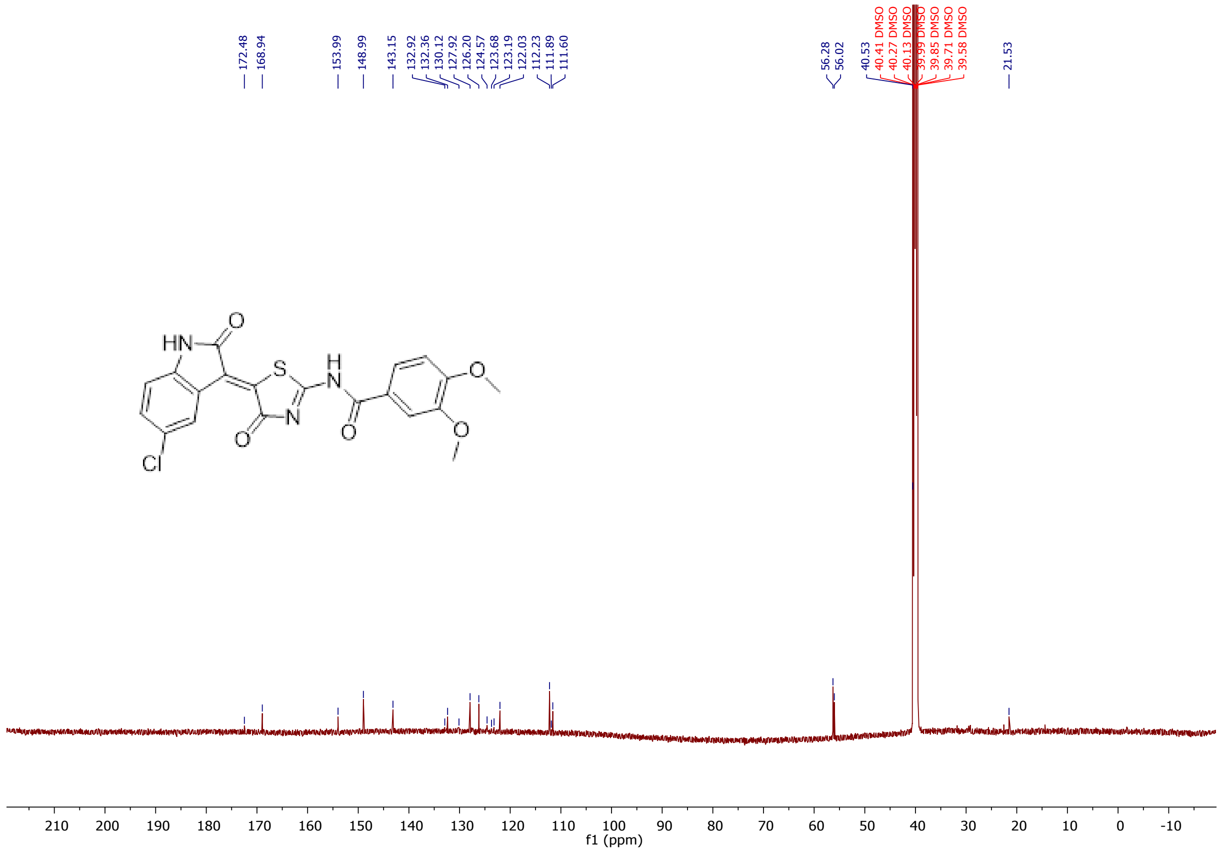

^{1}H NMR Spectrum of **37** (600 MHz, DMSO-d_6):

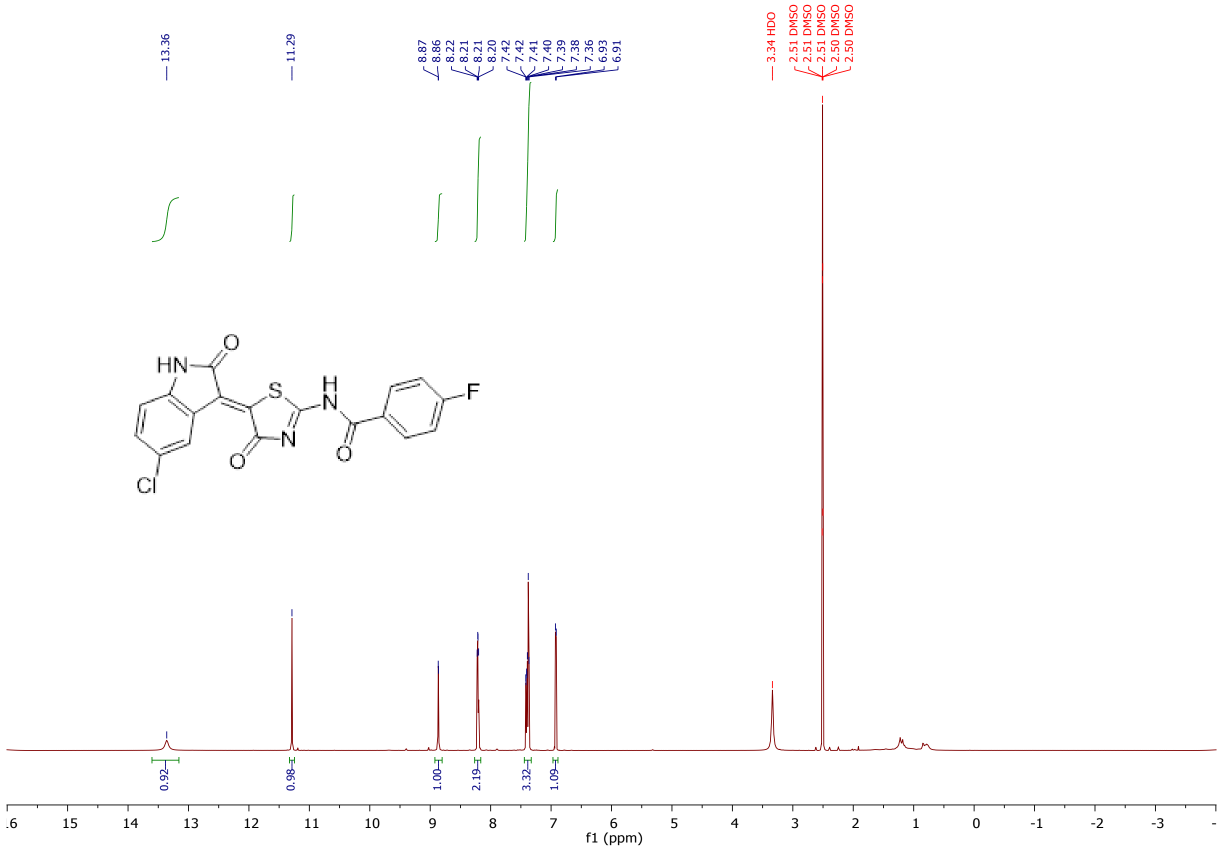

^{13}C NMR Spectrum of **37** (151 MHz, DMSO-d_6):

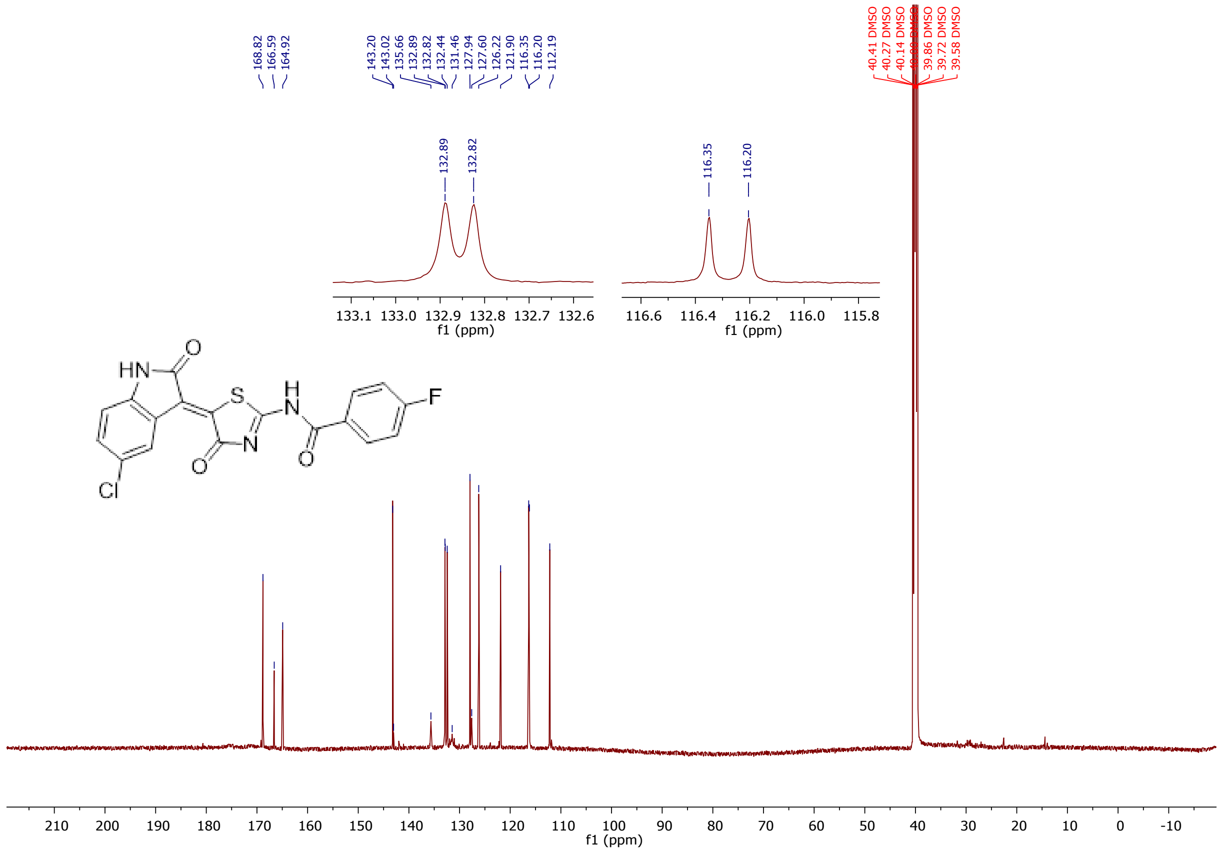

^{19}F NMR Spectrum of **37** (470 MHz, DMSO-d_6):

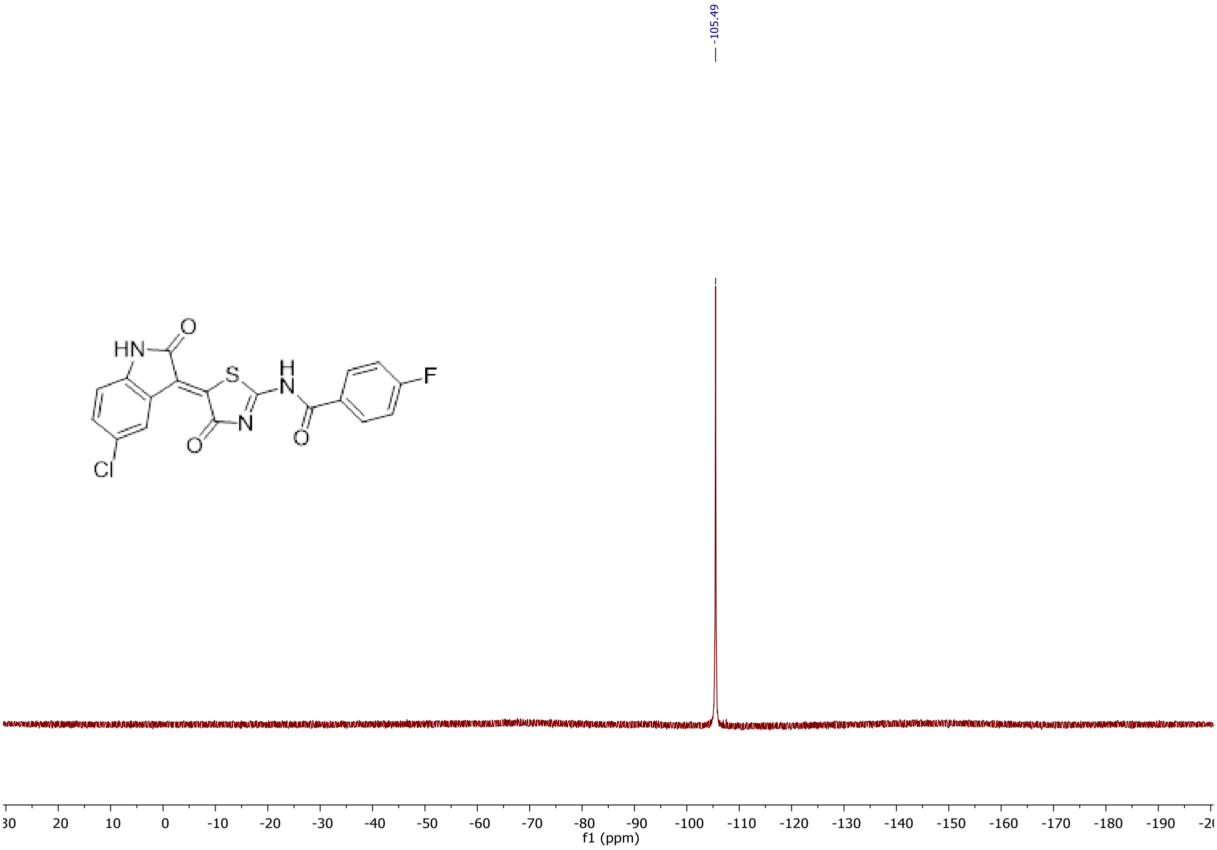

^{1}H NMR Spectrum of **38** (600 MHz, DMSO-d_6):

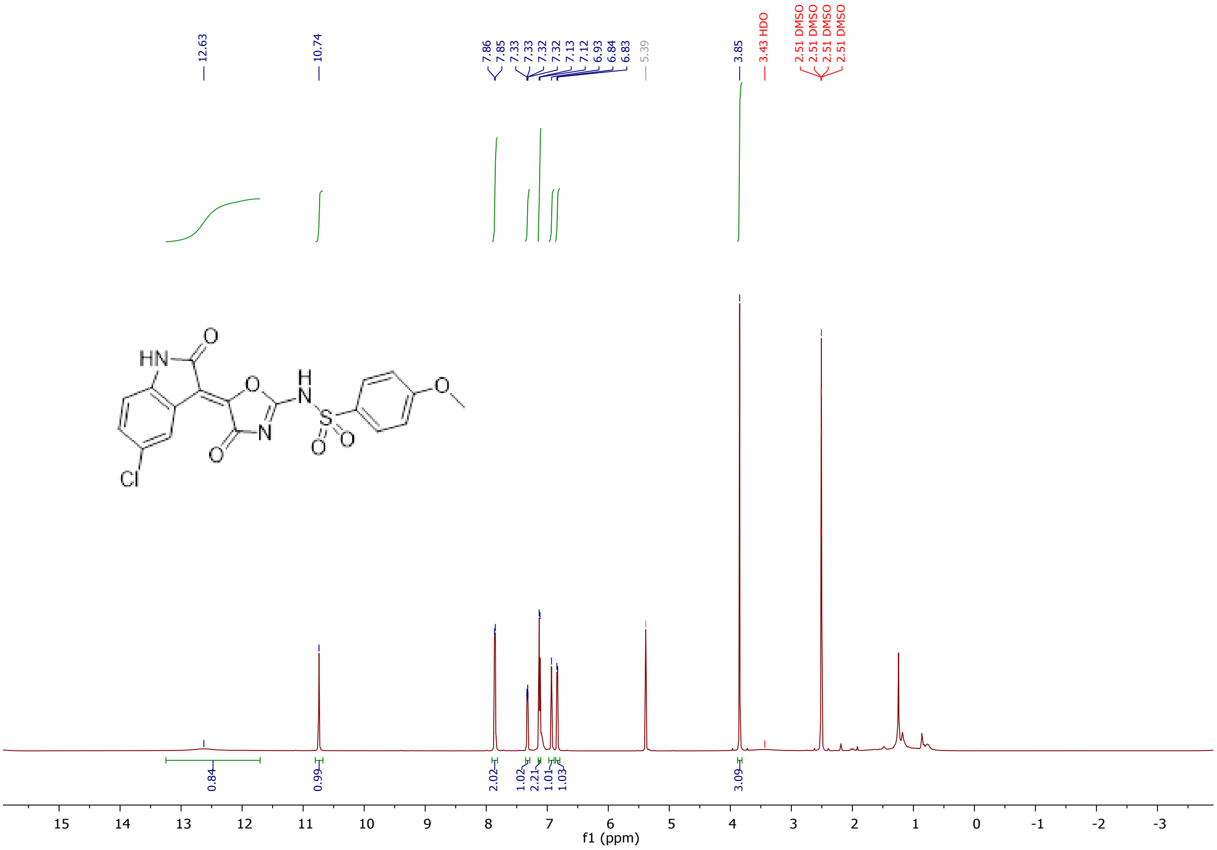

^{13}C NMR Spectrum of **38** (151 MHz, DMSO-d_6):

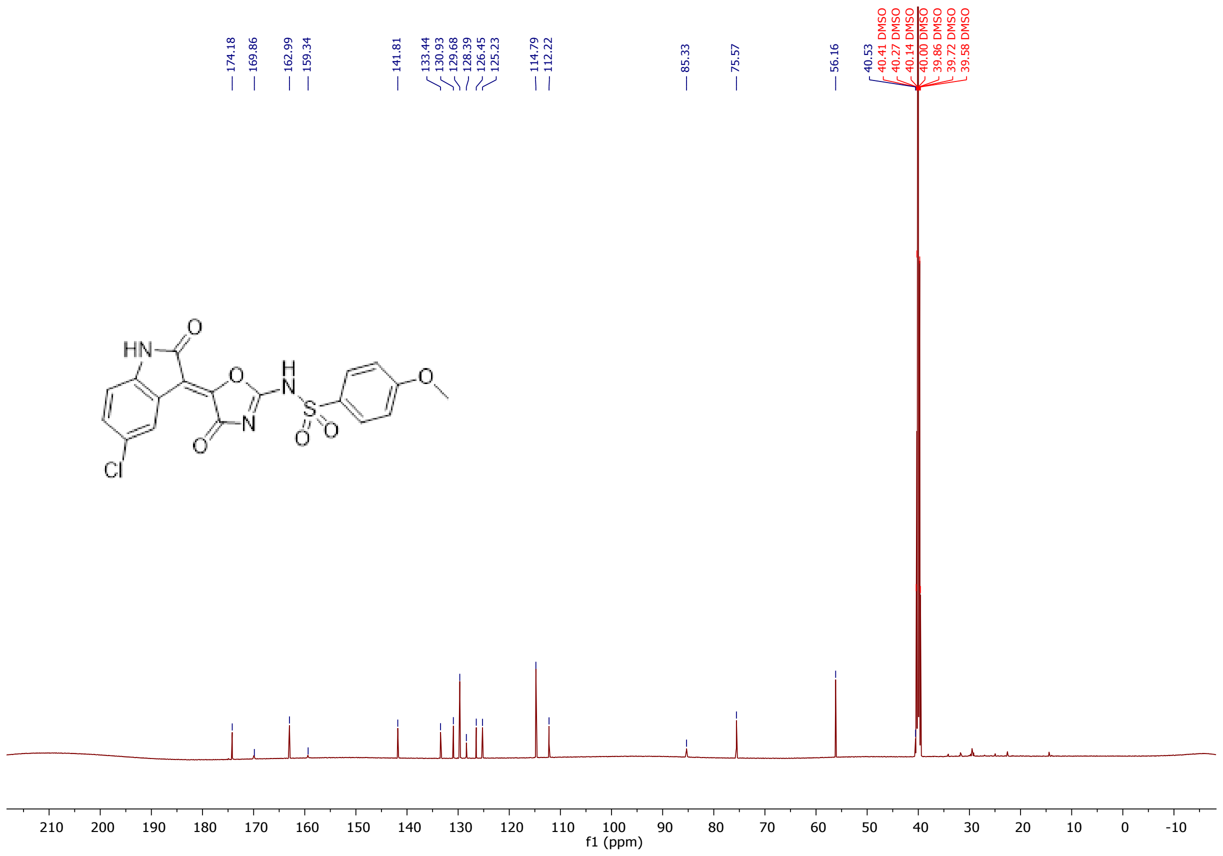

^{1}H NMR Spectrum of **39** (700 MHz, DMSO-d_6):

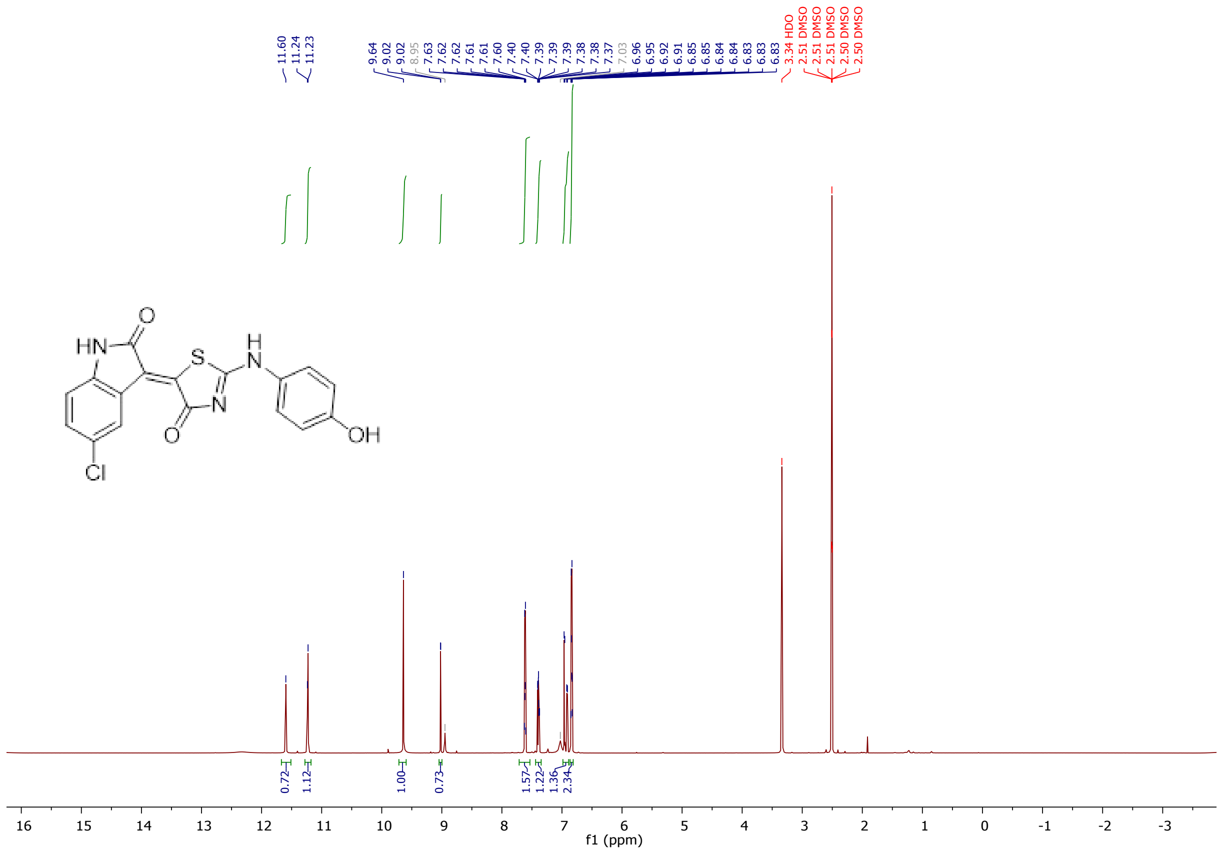

^{13}C NMR Spectrum of **39** (174 MHz, DMSO-d_6):

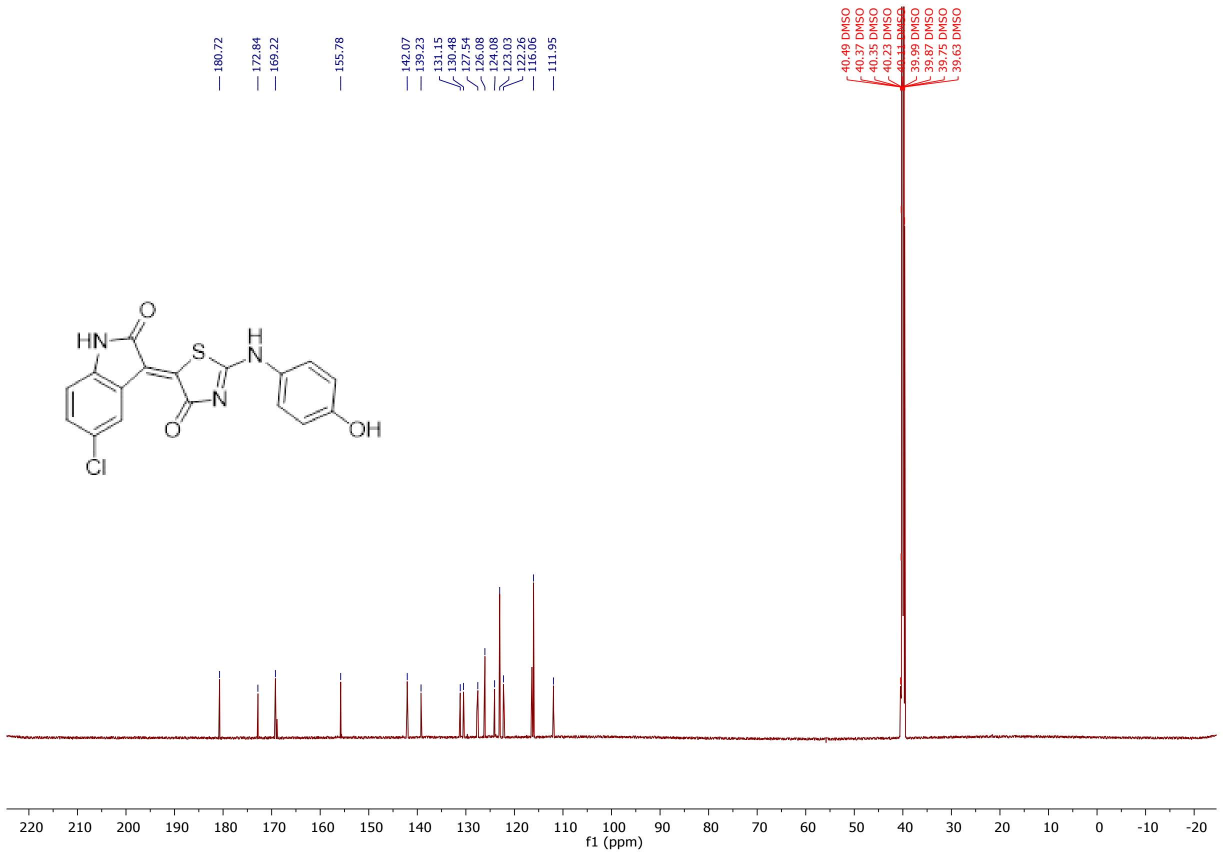

^{1}H NMR Spectrum of **40** (*E/Z* mixture = 0.13/1) (700 MHz, DMSO-d_6):

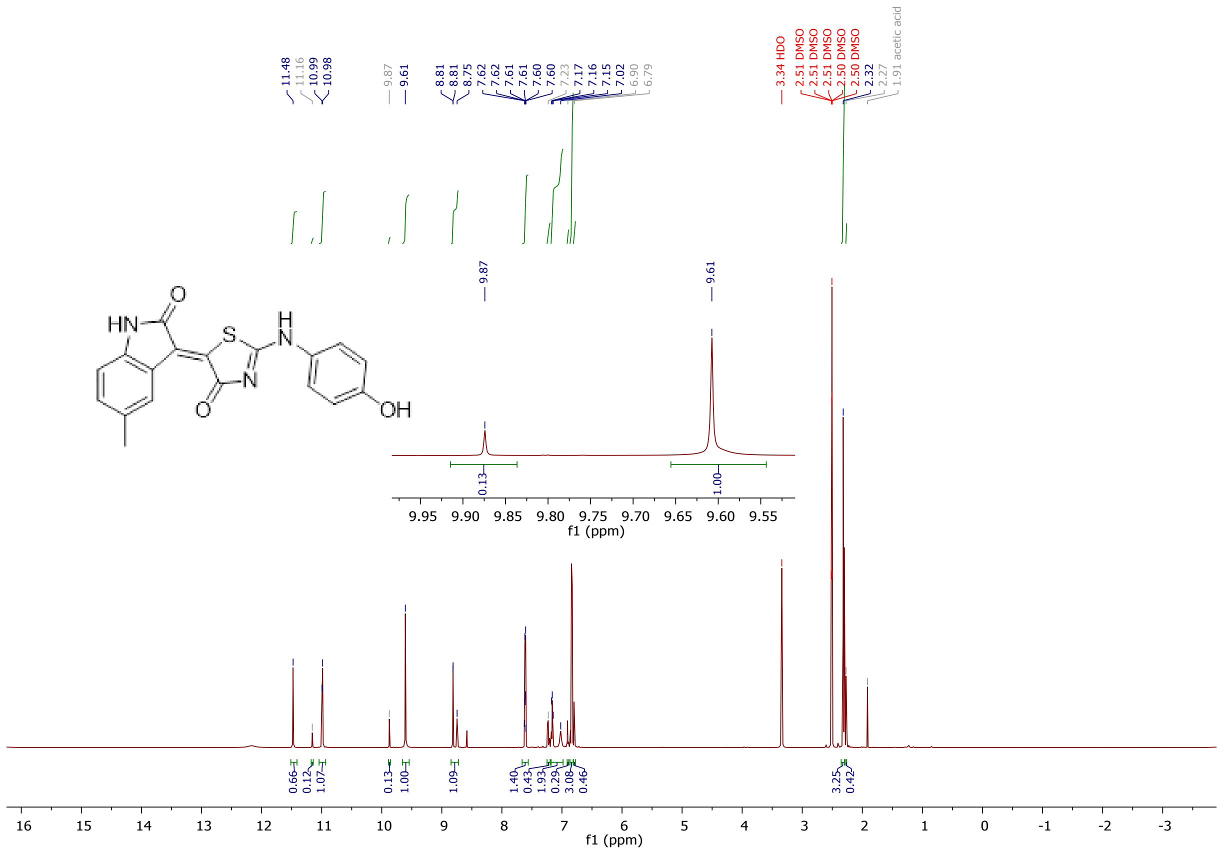

^{13}C NMR Spectrum of **40** (174 MHz, DMSO-d_6):

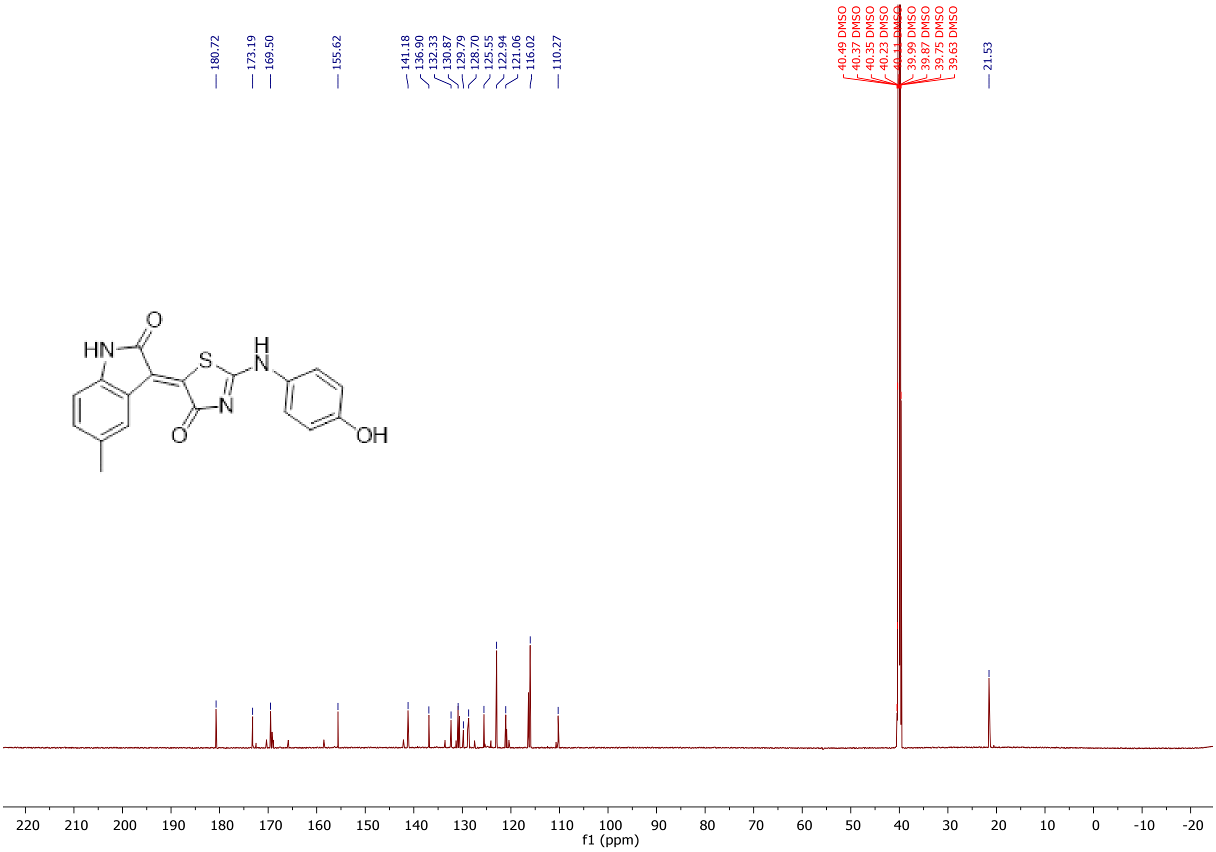

^{1}H NMR Spectrum of **41** (*E/Z* mixture = 0.13/1) (700 MHz, DMSO-d_6):

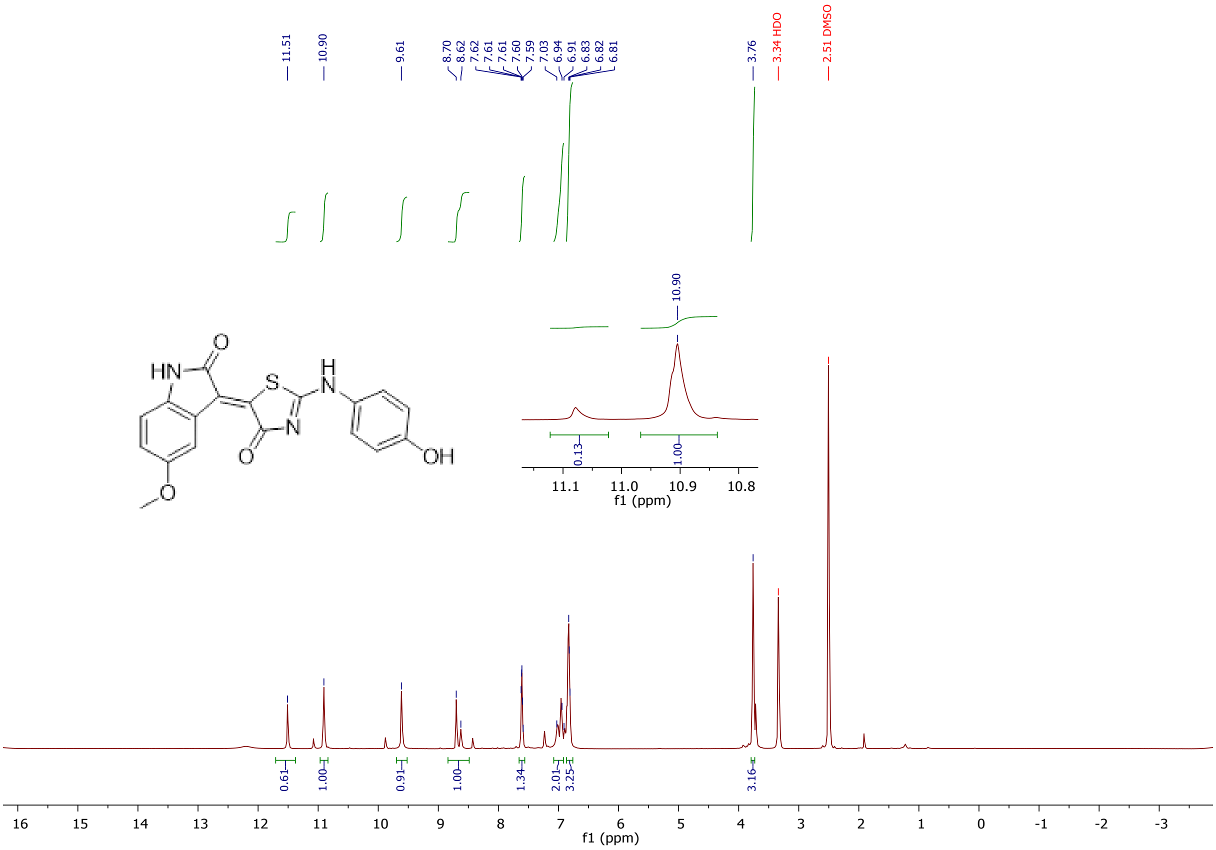

^{13}C NMR Spectrum of **41** (174 MHz, DMSO-d_6):

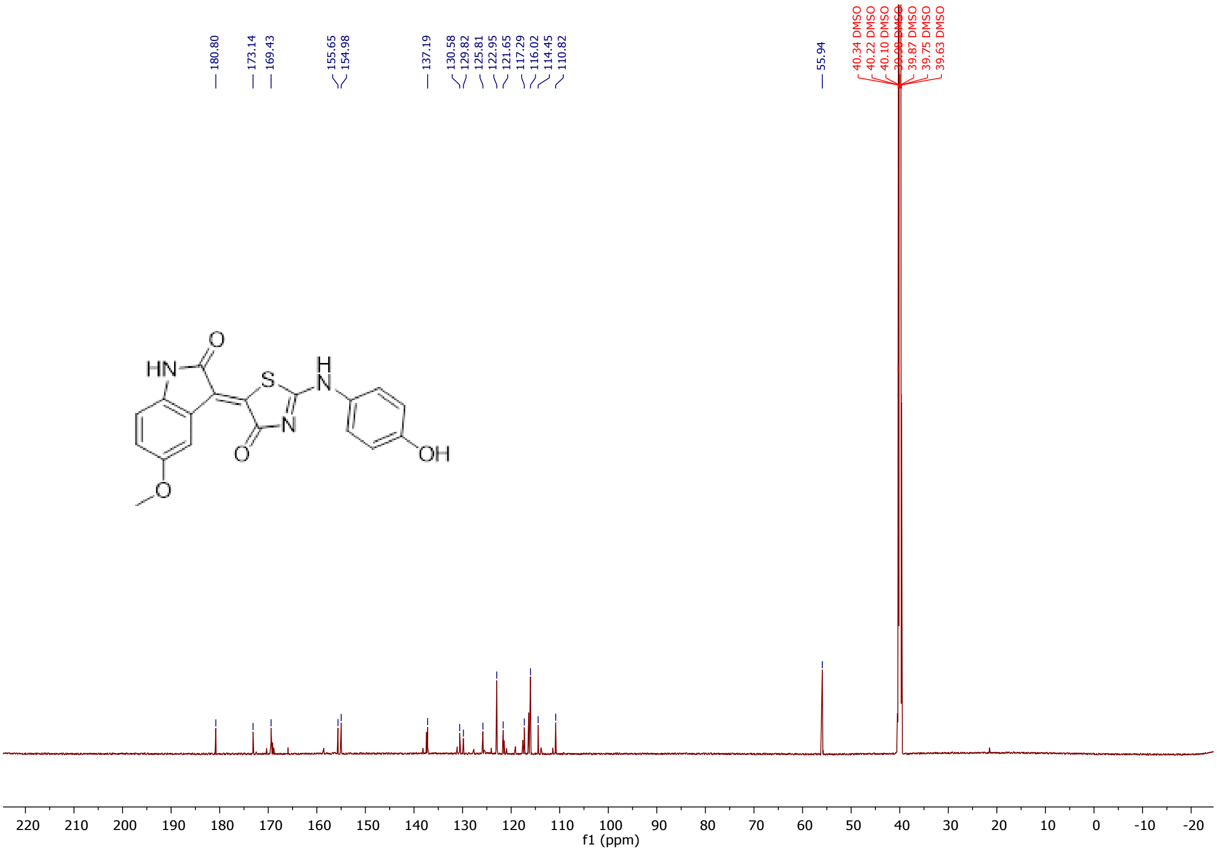

^{1}H NMR Spectrum of **42** (700 MHz, DMSO-d_6):

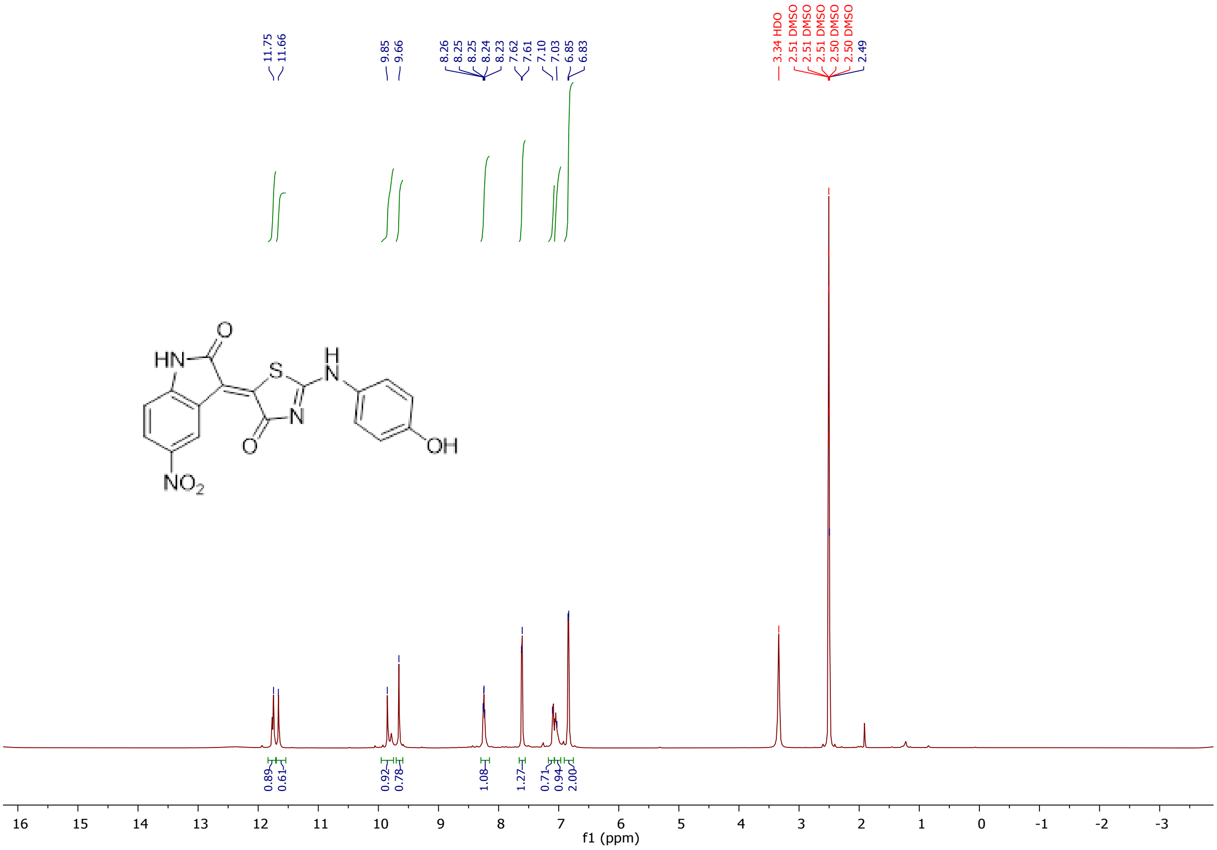

^{13}C NMR Spectrum of **42** (174 MHz, DMSO-d_6):

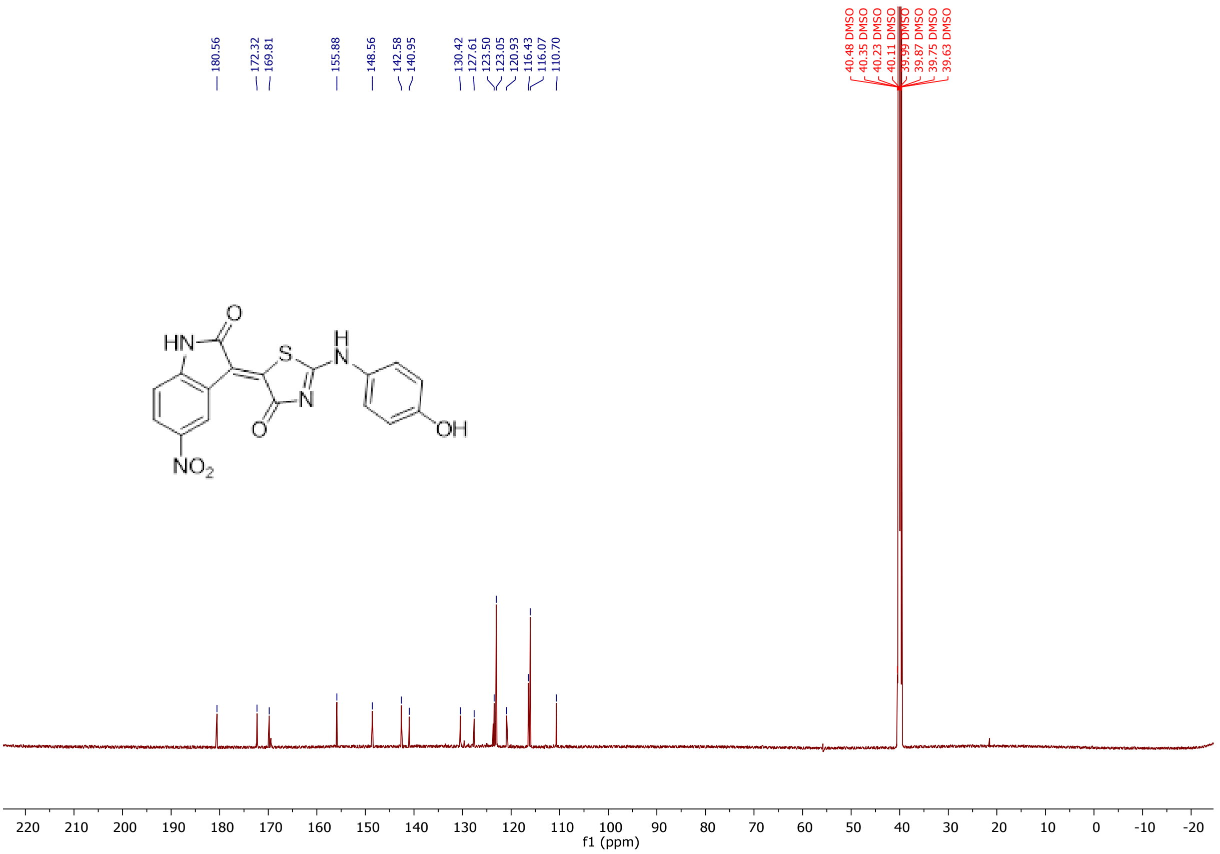

^{1}H NMR Spectrum of **43** (700 MHz, DMSO-d_6):

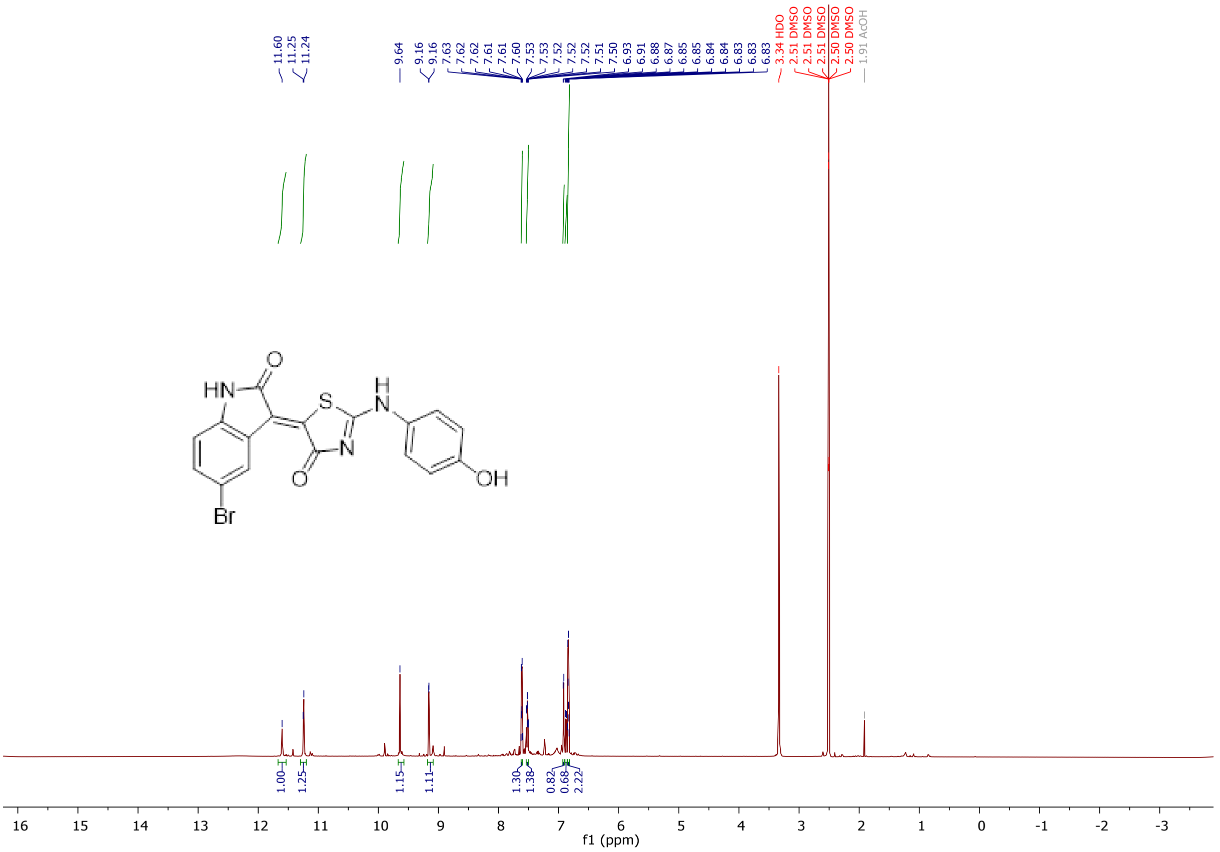

^{13}C NMR Spectrum of **43** (174 MHz, DMSO-d_6):

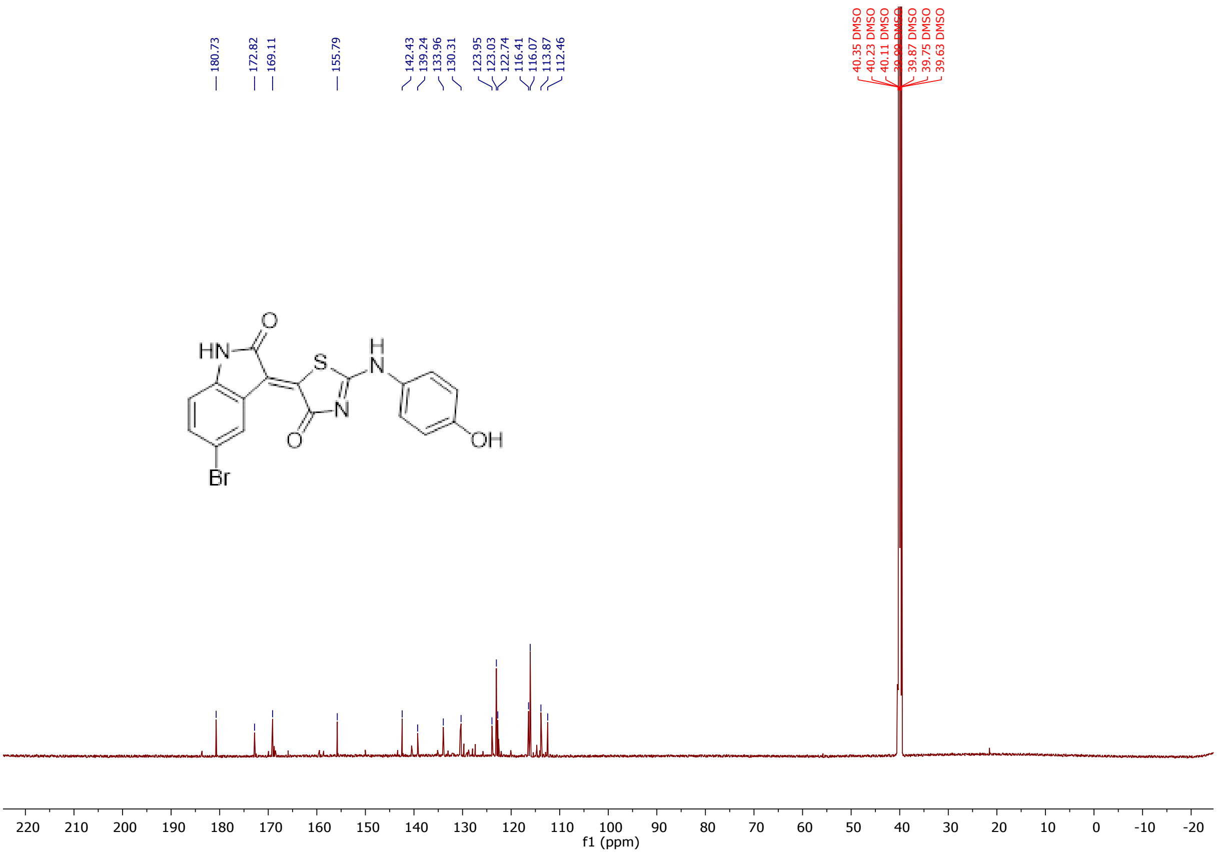

^{1}H NMR Spectrum of **44** (*E/Z* mixture = 0.14/1) (600 MHz, DMSO-d_6):

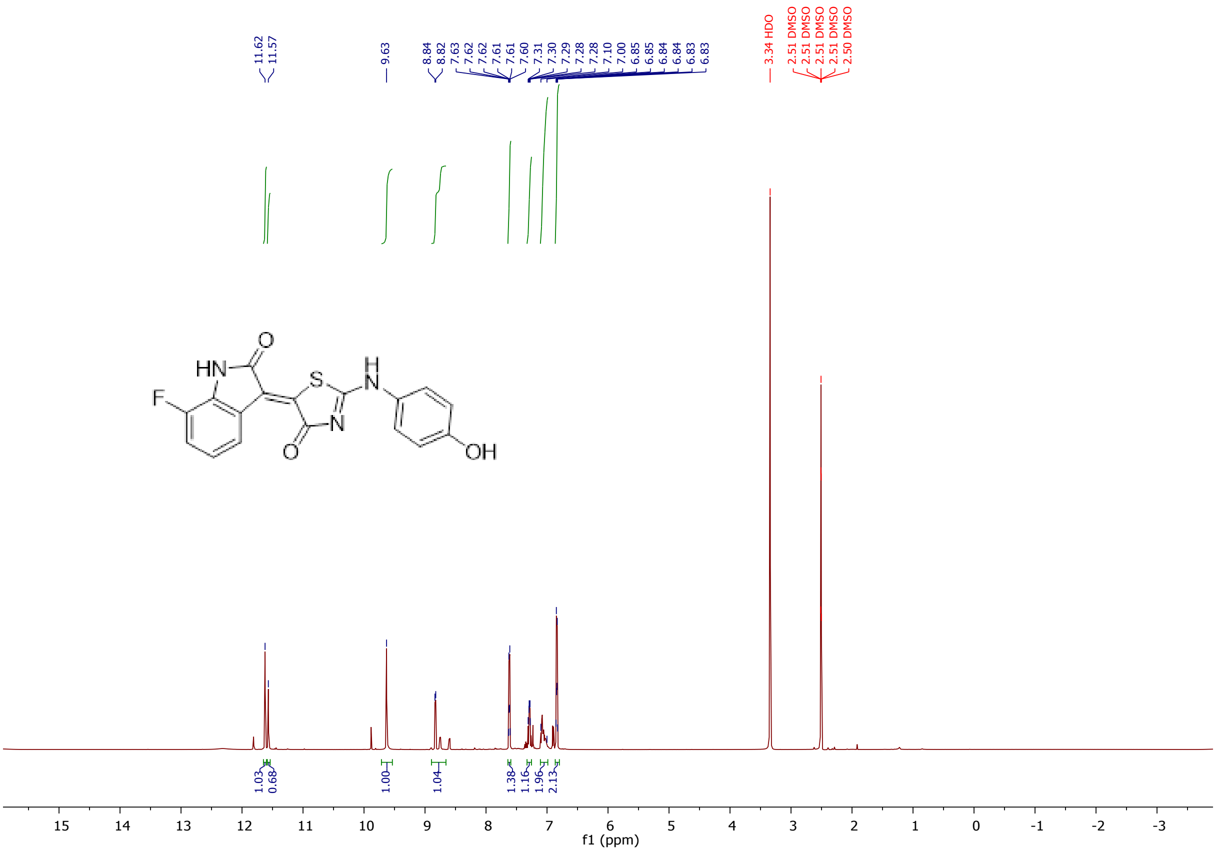

^{13}C NMR Spectrum of **44** (151 MHz, DMSO-d_6):

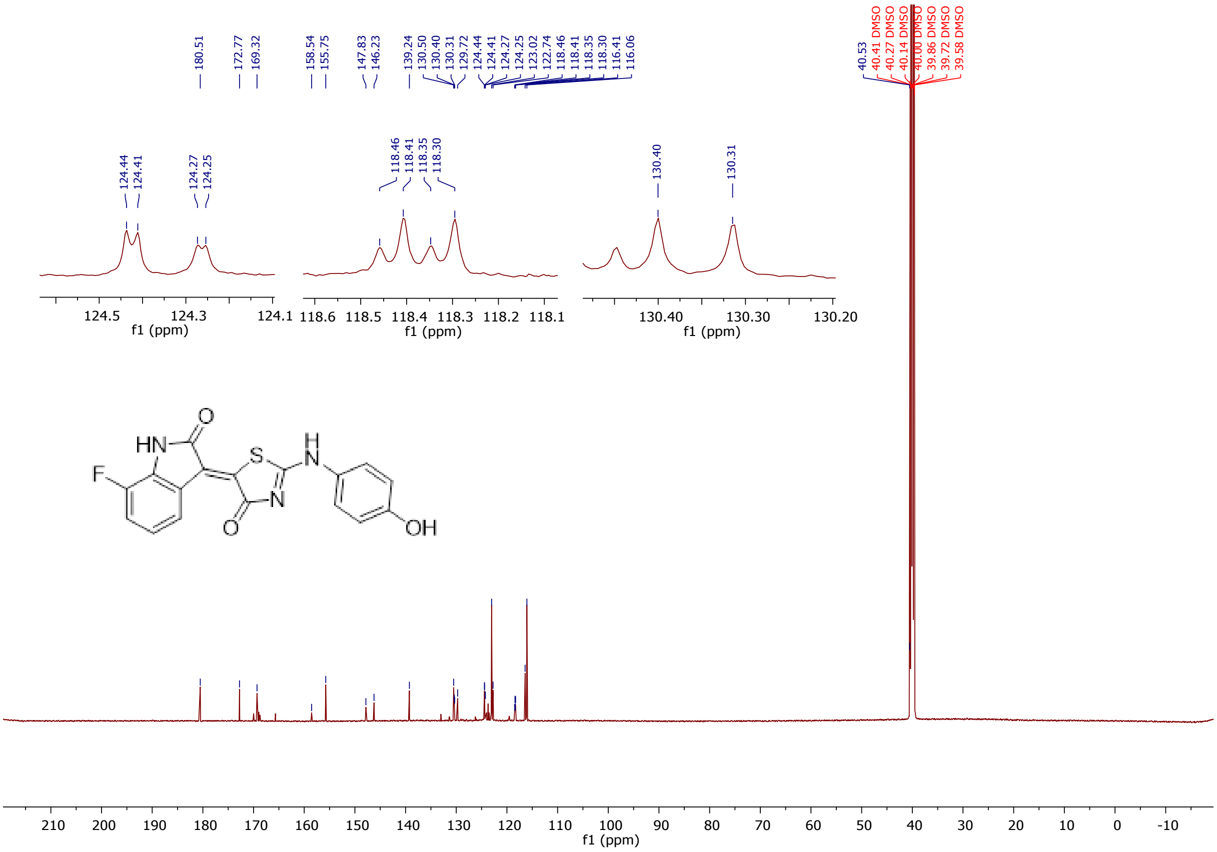

^{19}F NMR Spectrum of **44** (470 MHz, DMSO-d_6):

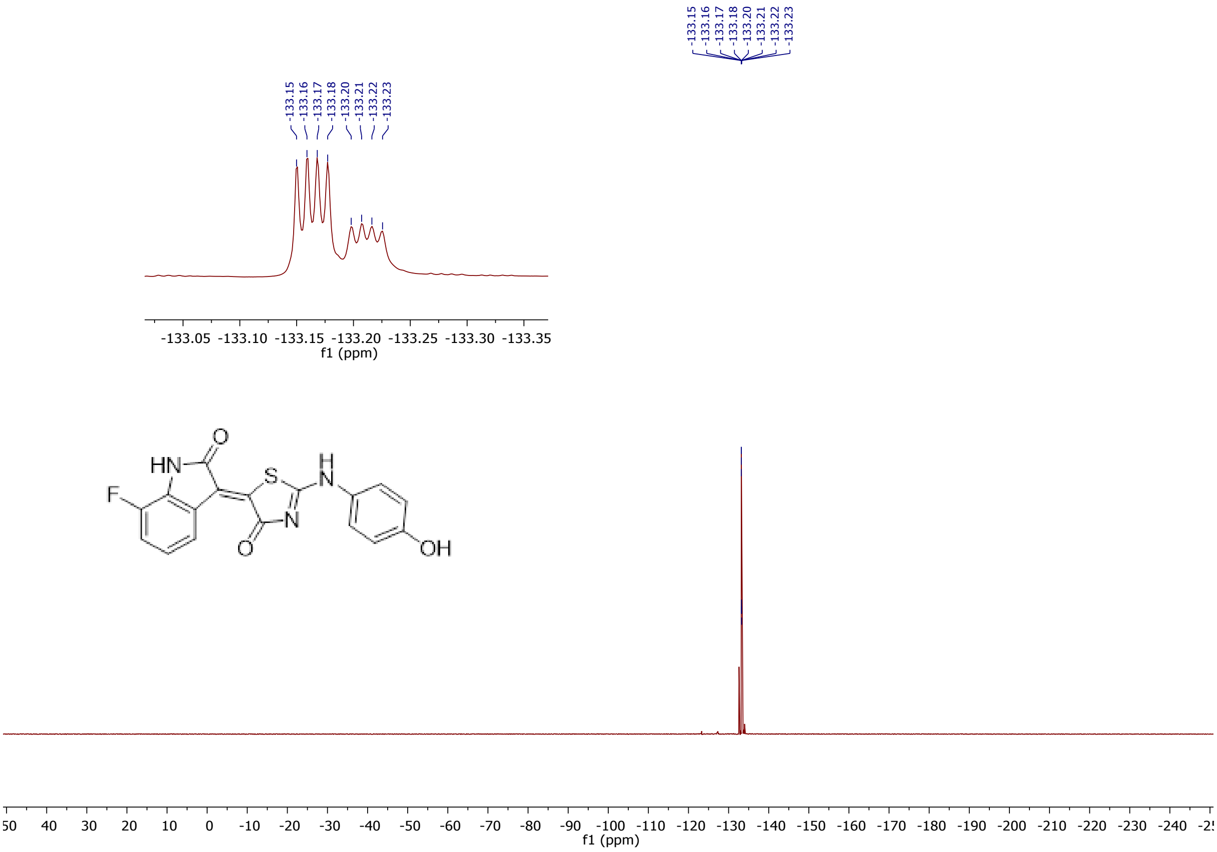

^{1}H NMR Spectrum of **45** (600 MHz, DMSO-d_6) (*E/Z* mixture = 4:1):

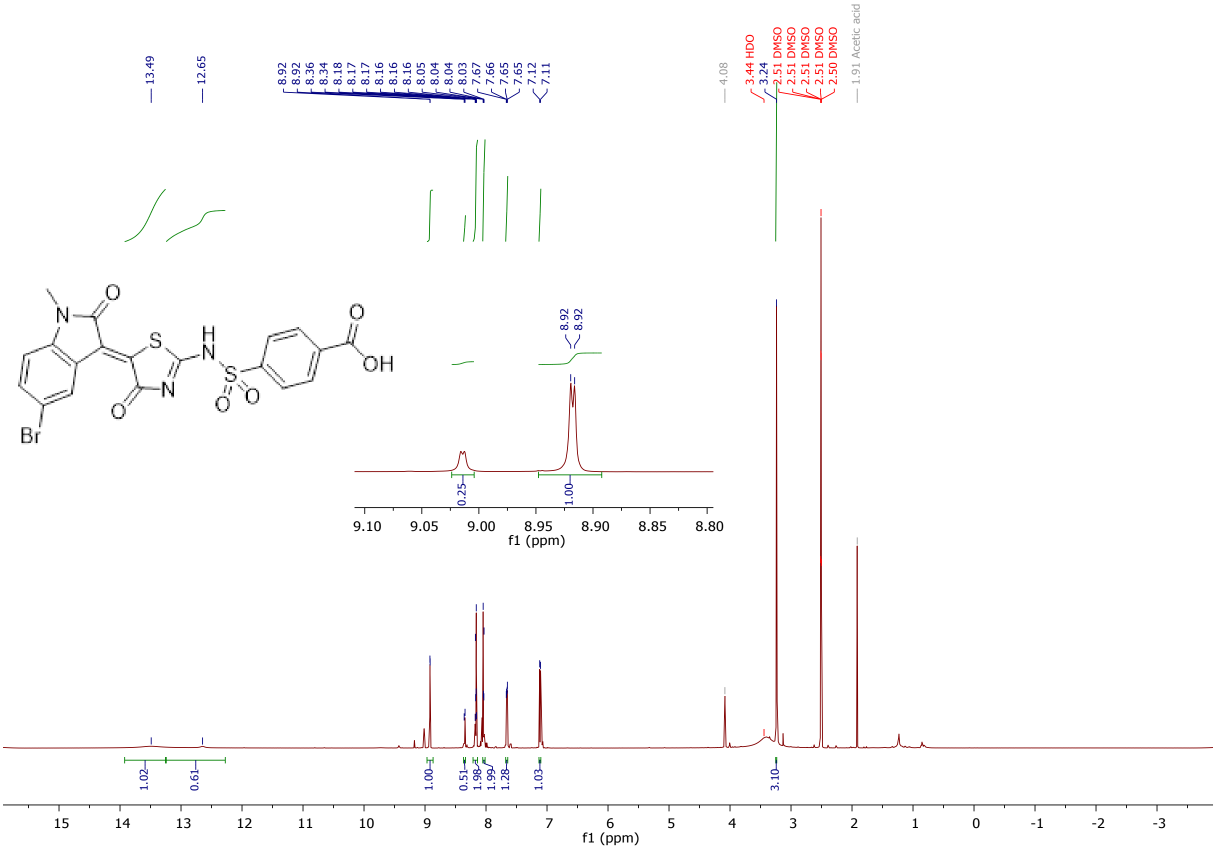

^{13}C NMR Spectrum of **45** (151 MHz, DMSO-d_6) (*E/Z* mixture = 4:1):

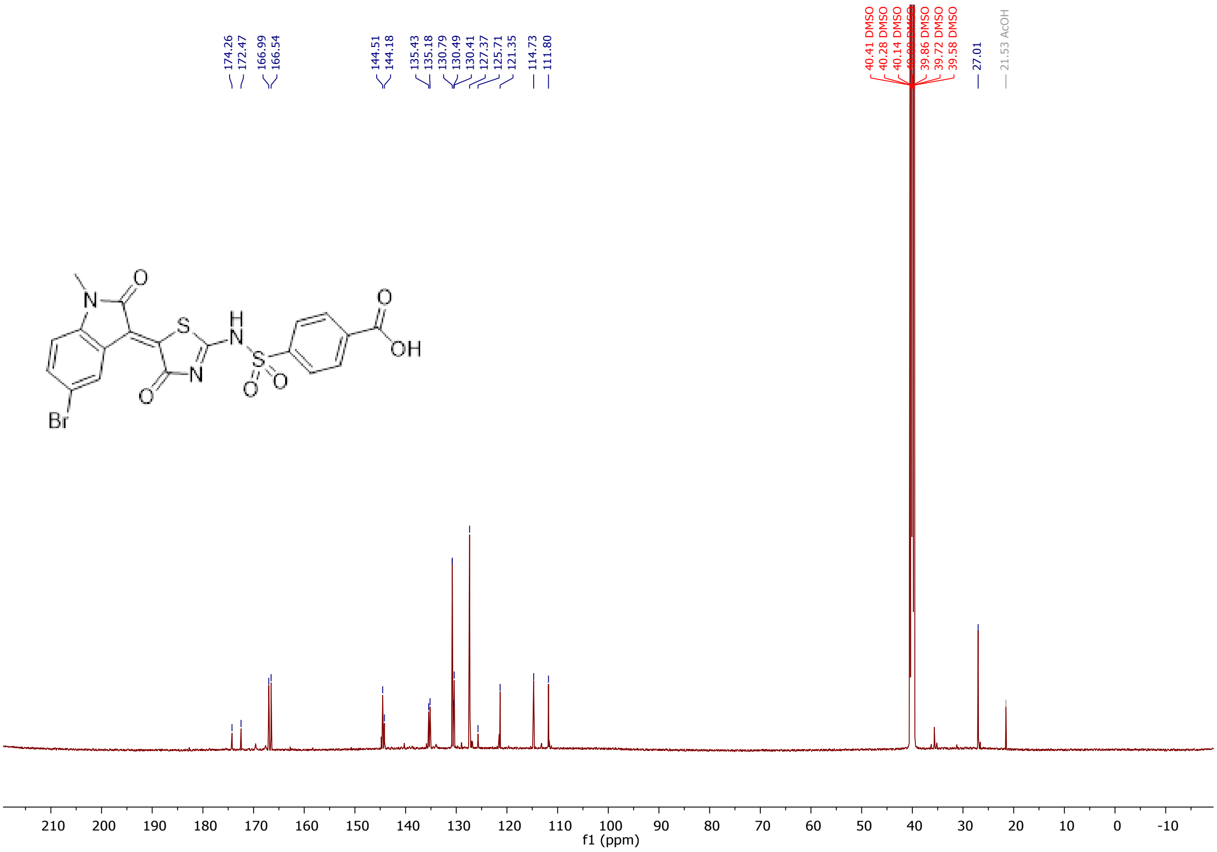

^{1}H NMR Spectrum of **46** (700 MHz, DMSO-d_6):

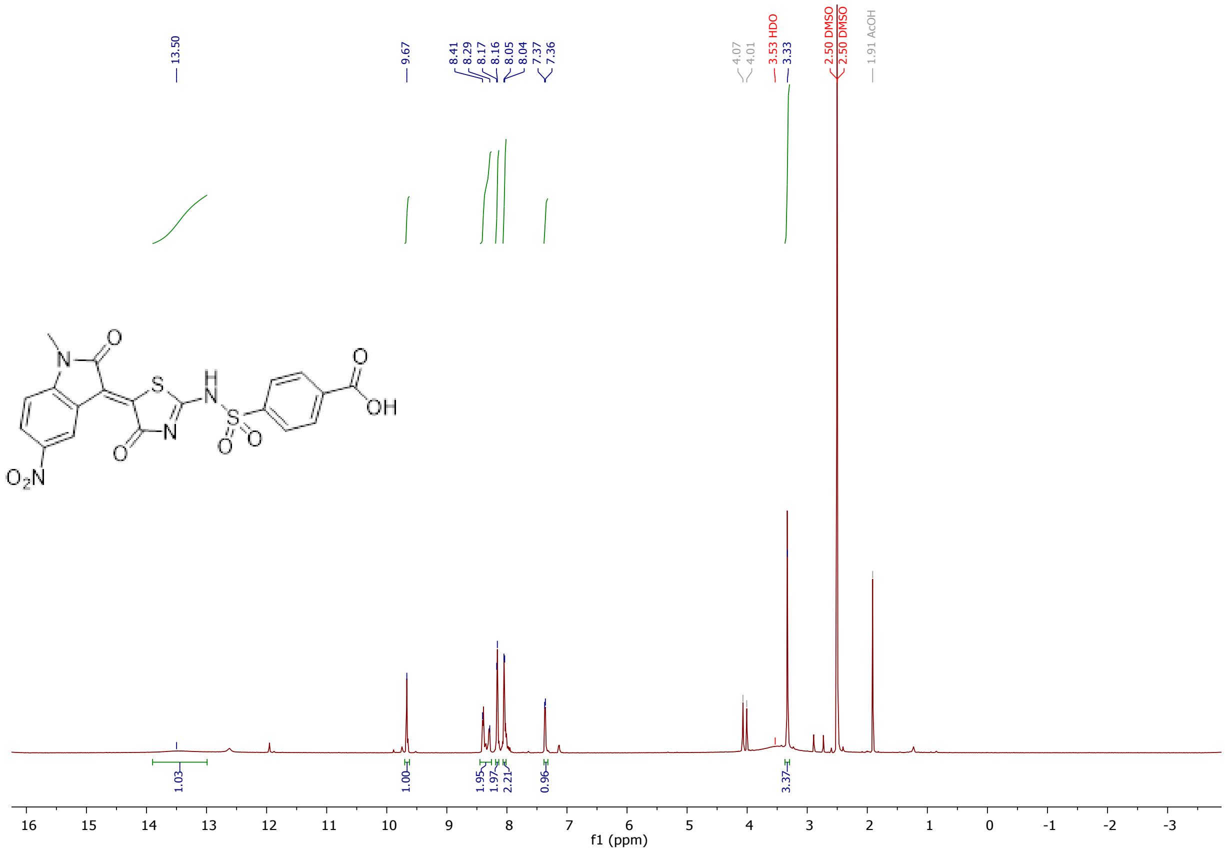

^{13}C NMR Spectrum of **46** (174 MHz, DMSO-d_6):

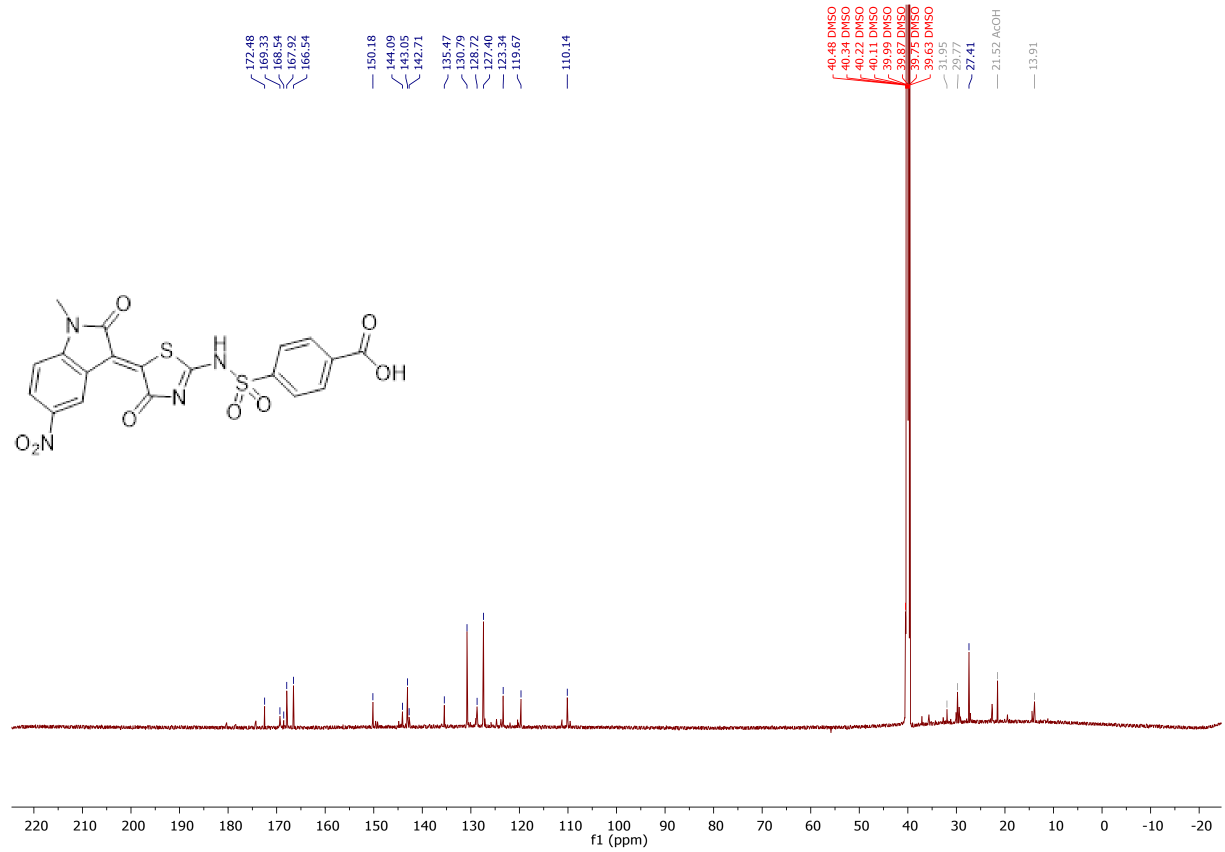

^{1}H NMR Spectrum of **47** (600 MHz, DMSO-d_6):

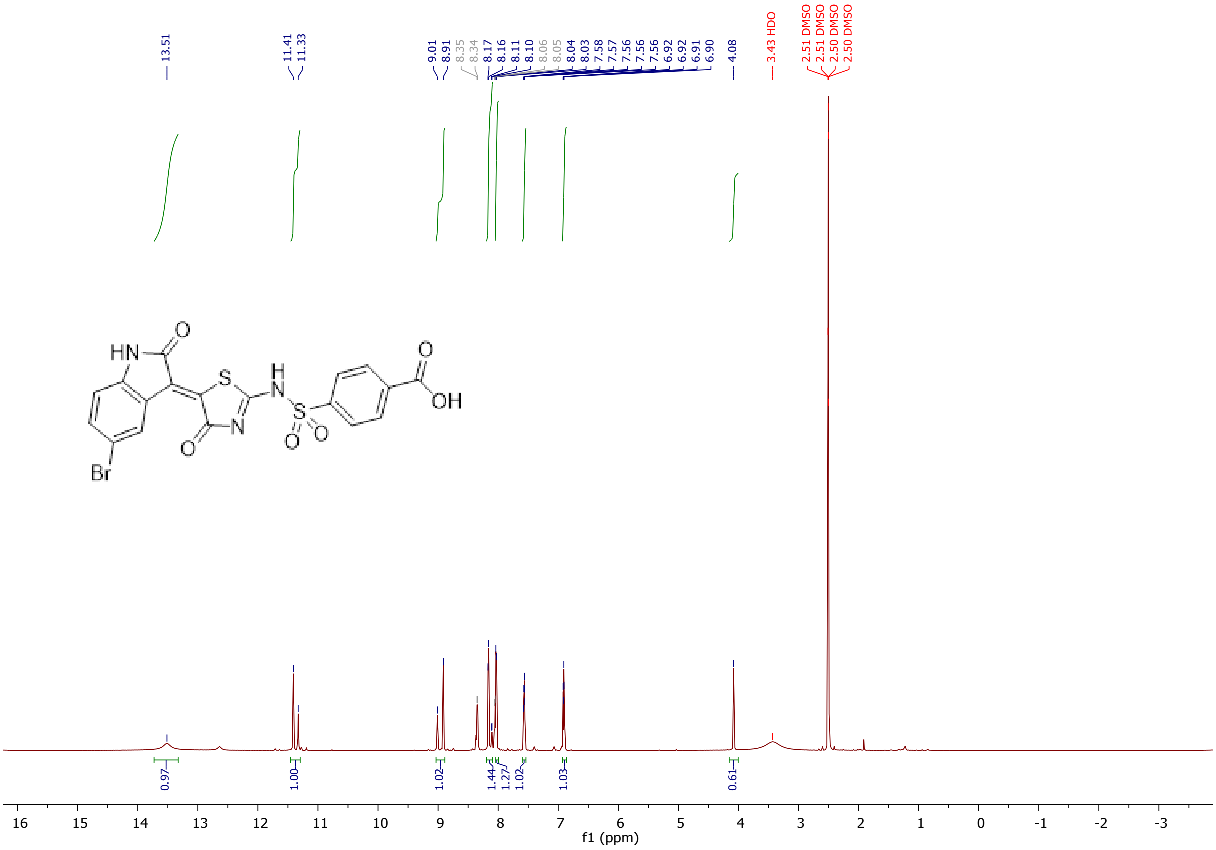

^{13}C NMR Spectrum of **47** (151 MHz, DMSO-d_6):

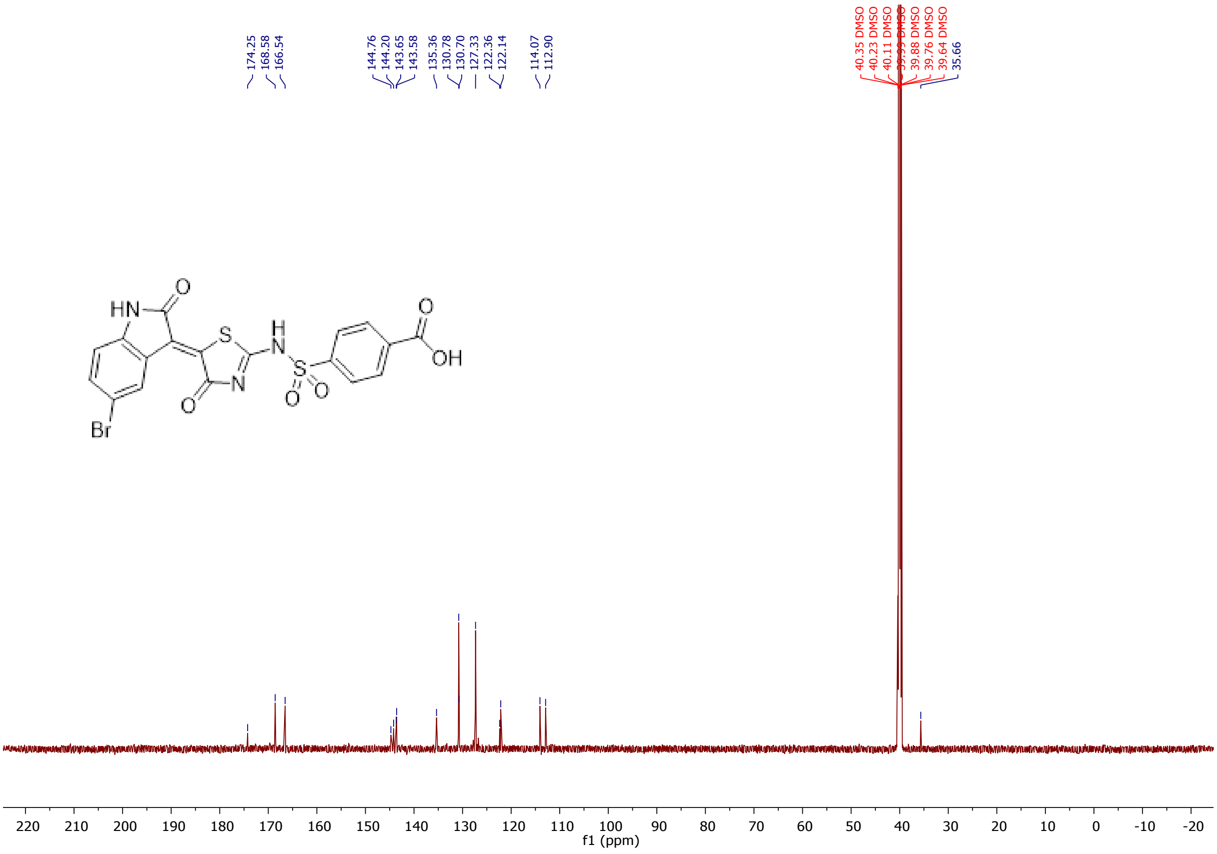

^{1}H NMR Spectrum of **48** (500 MHz, DMSO-d_6):

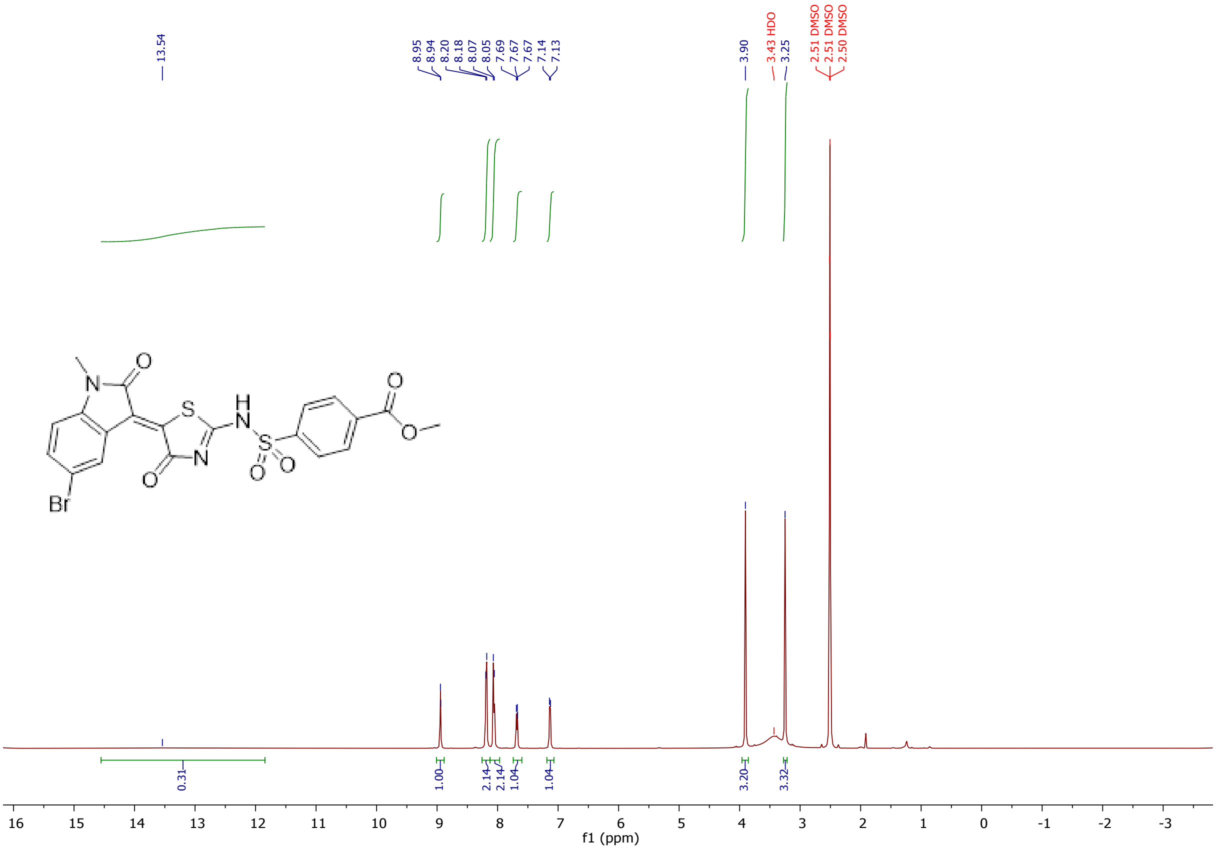

^{13}C NMR Spectrum of **48** (126 MHz, DMSO-d_6):

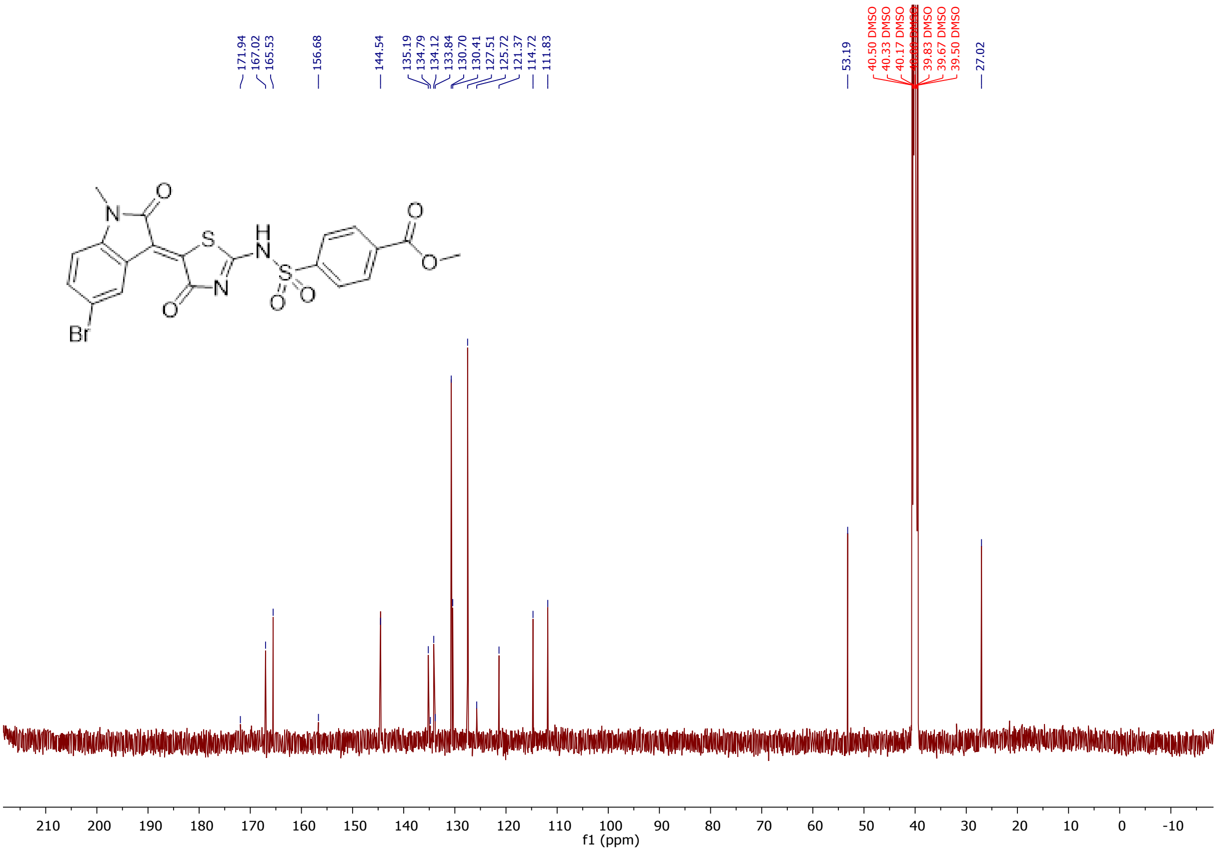

^{1}H NMR Spectrum of **49** (700 MHz, DMSO-d_6):

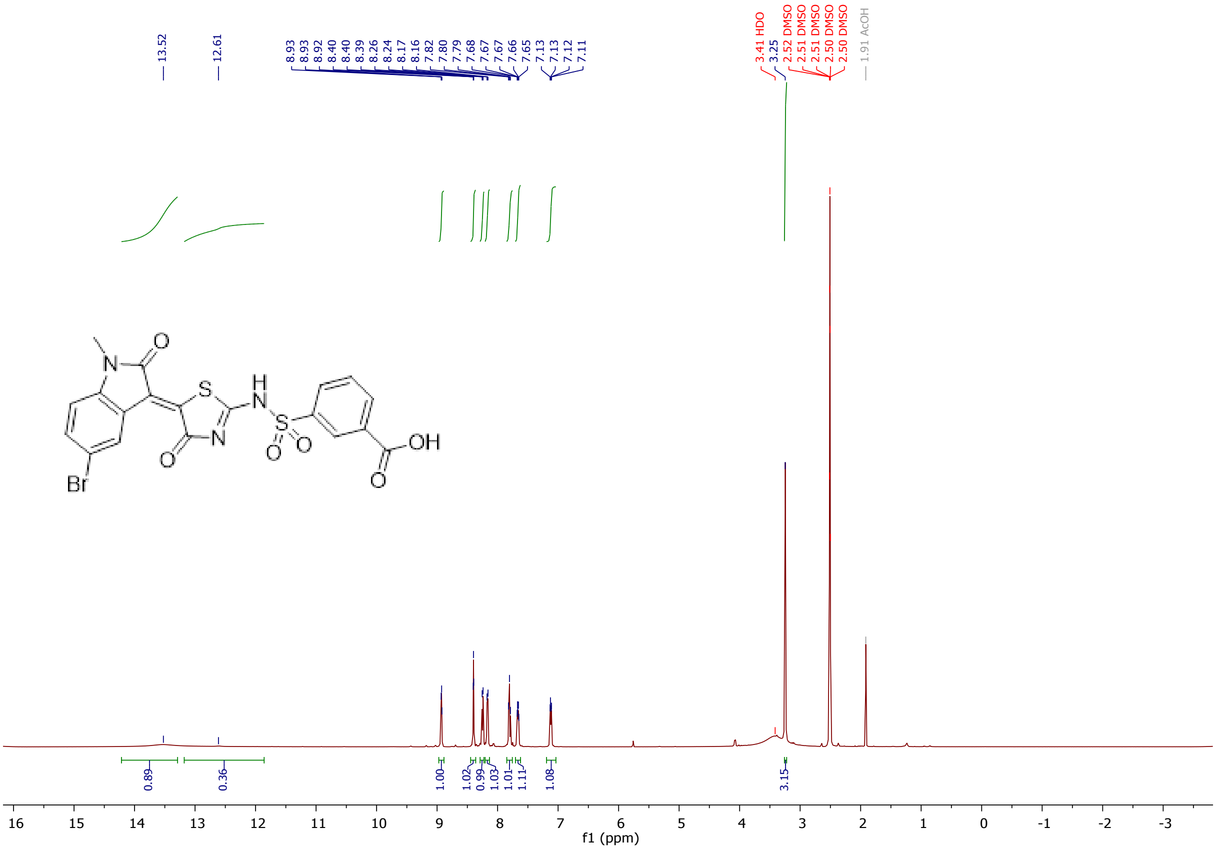

^{13}C NMR Spectrum of **49** (174 MHz, DMSO-d_6):

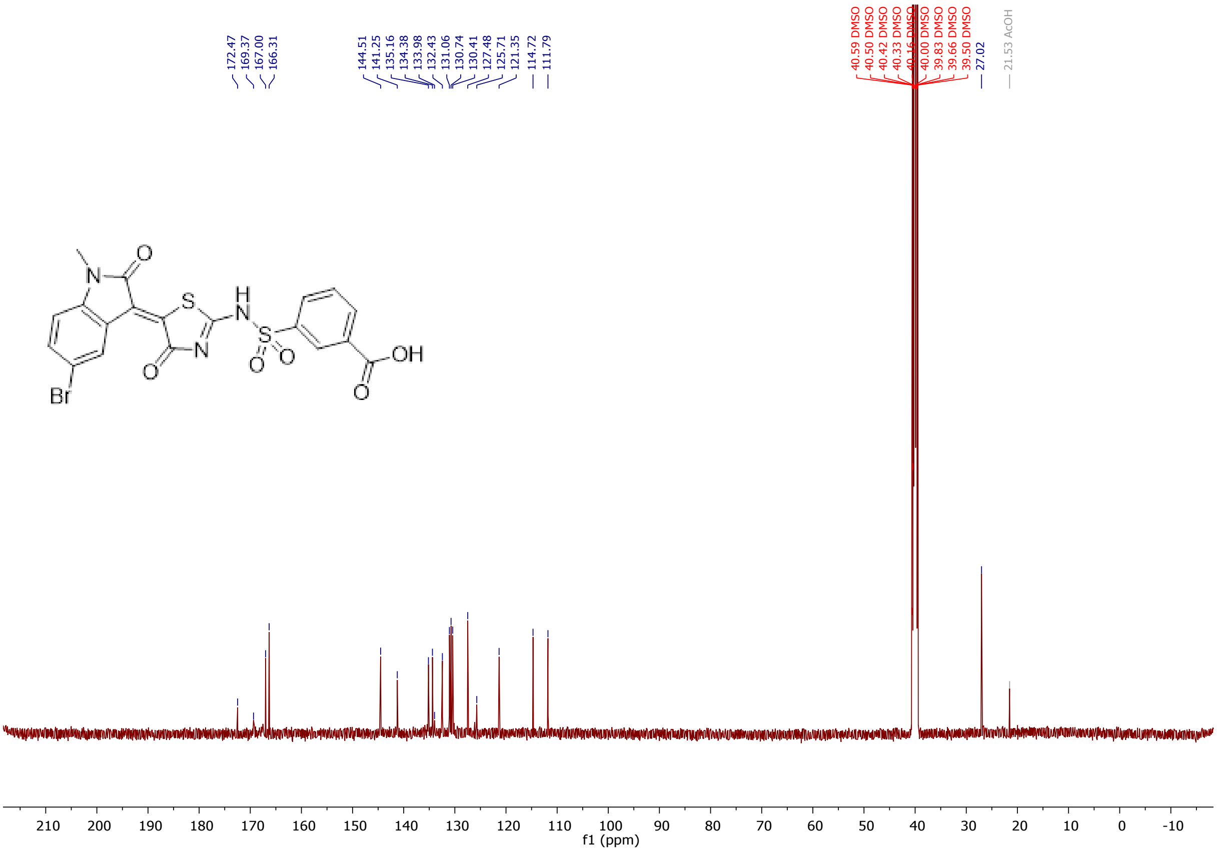

LC-MS Spectra of Aminothiazolones (1-49)

LC-MS Spectrum of **1**:

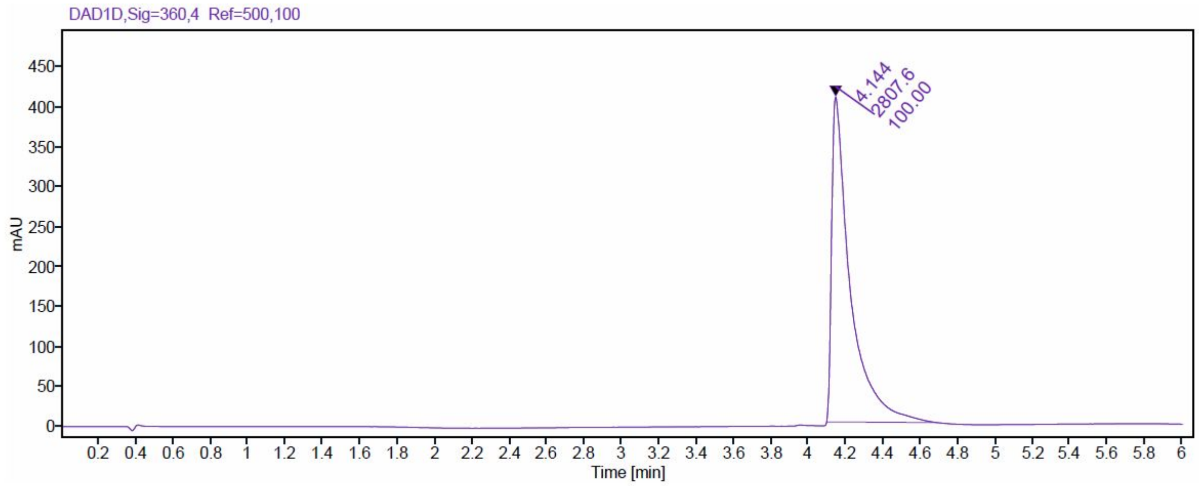

LC-MS Spectrum of **2**:

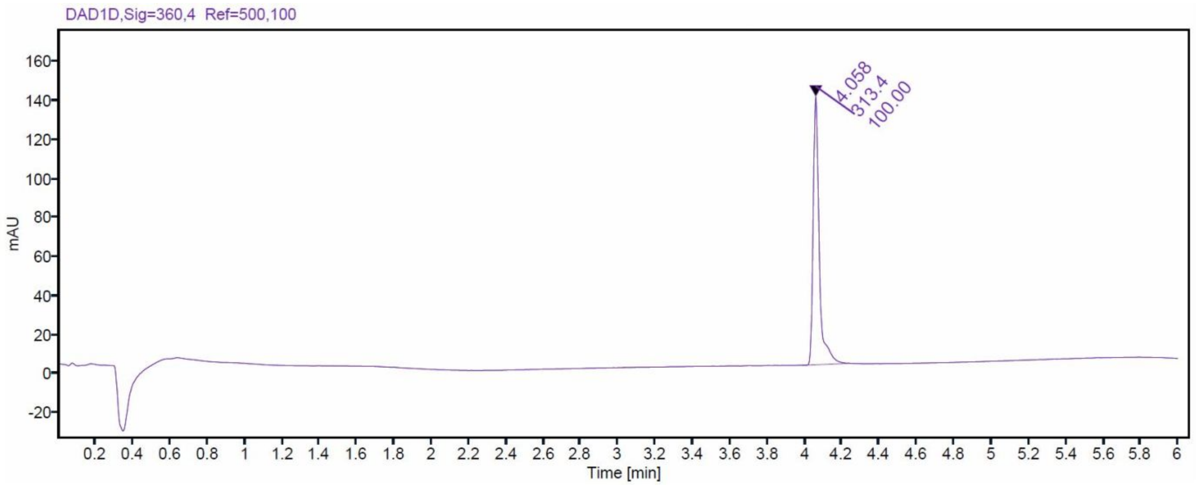

LC-MS Spectrum of **3**:

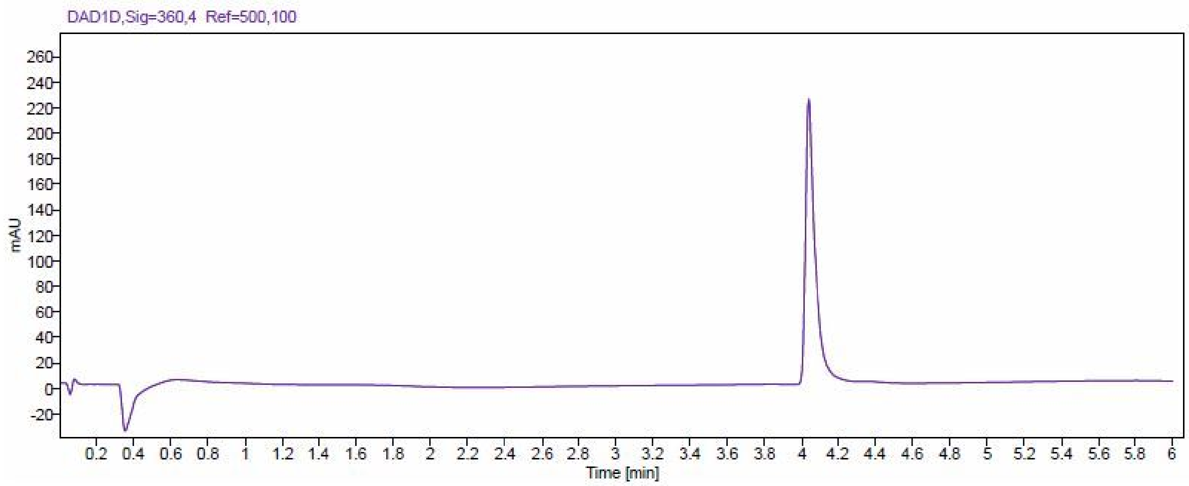

LC-MS Spectrum of **4**:

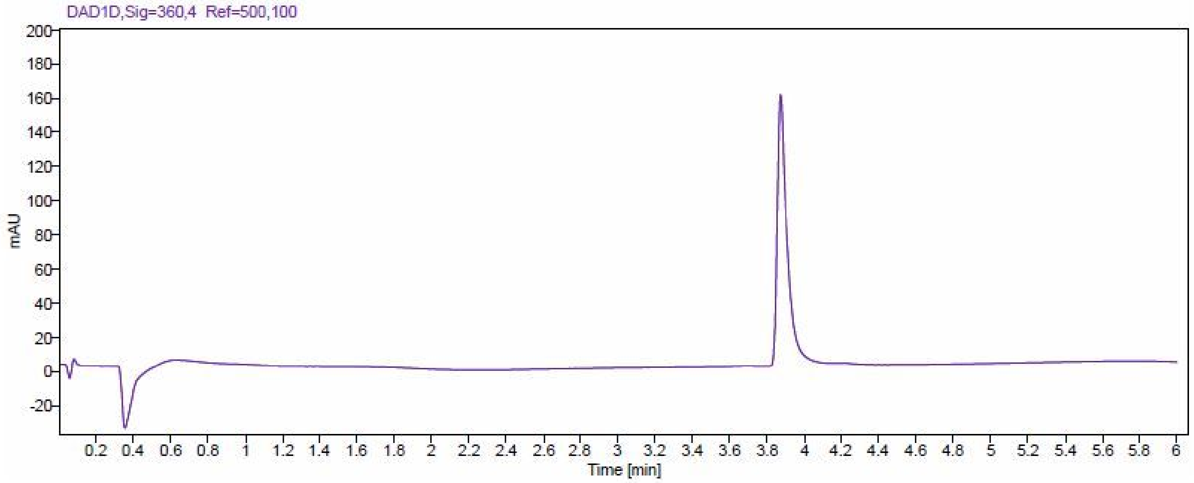

LC-MS Spectrum of **5**:

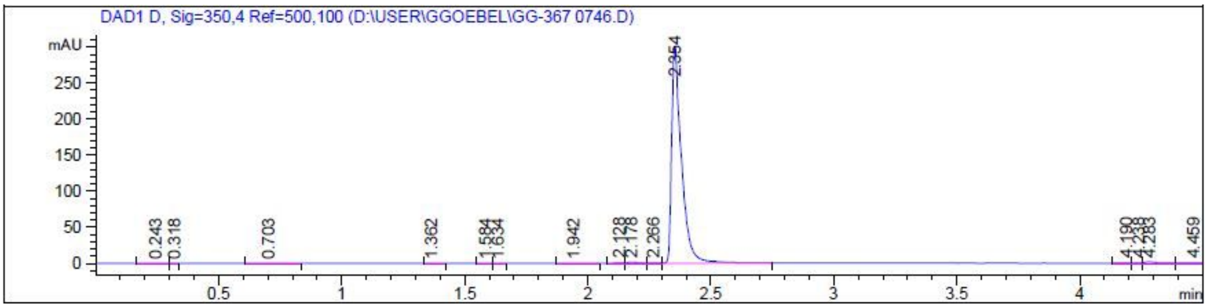

LC-MS Spectrum of **6**:

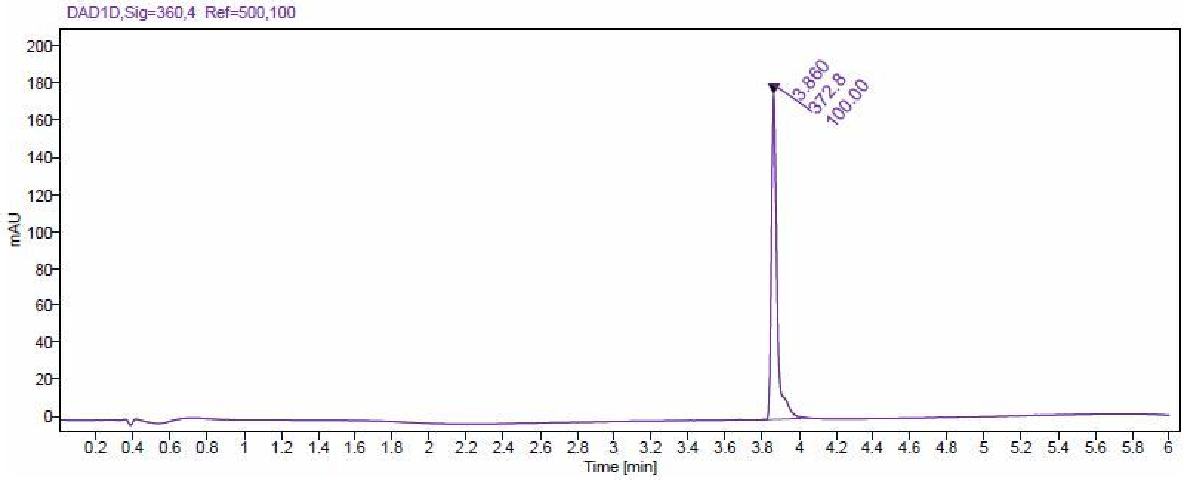

LC-MS Spectrum of **7**:

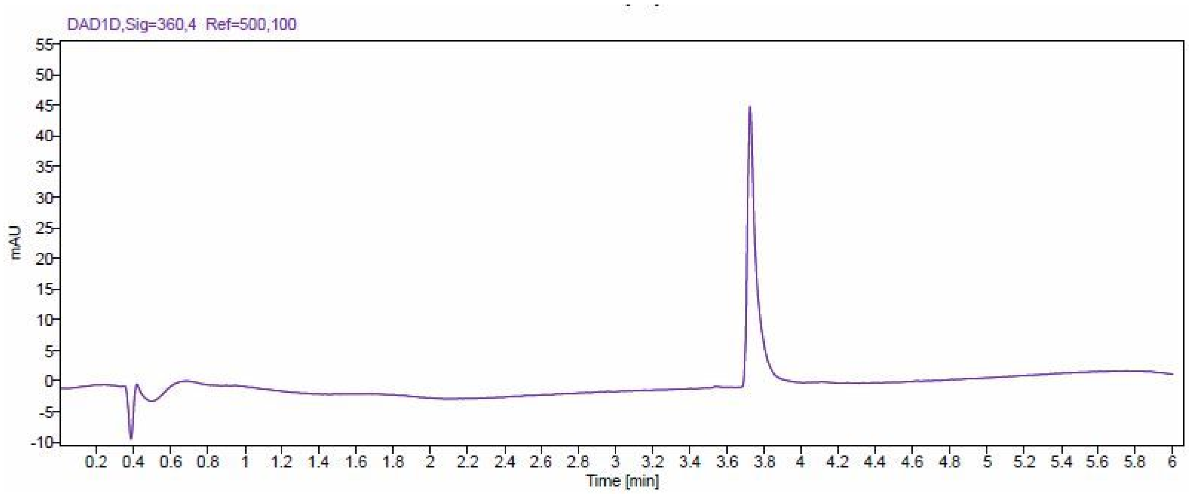

LC-MS Spectrum of **8**:

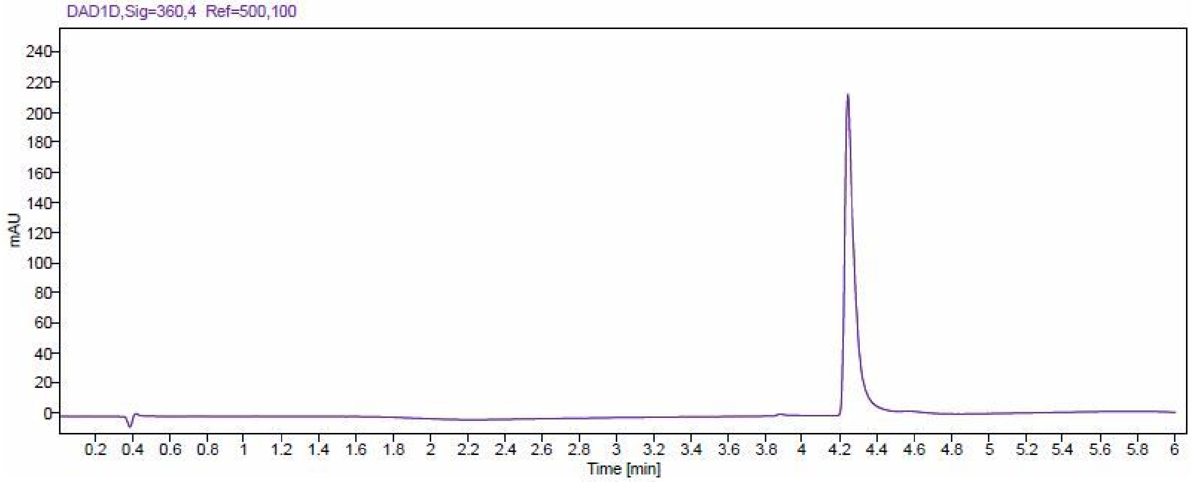

LC-MS Spectrum of **9**:

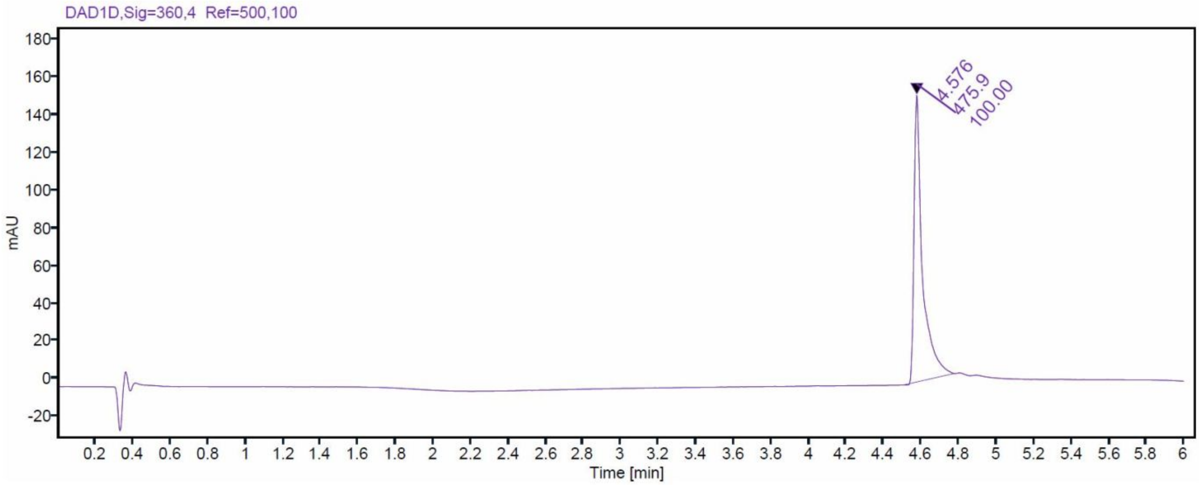

LC-MS Spectrum of **10**:

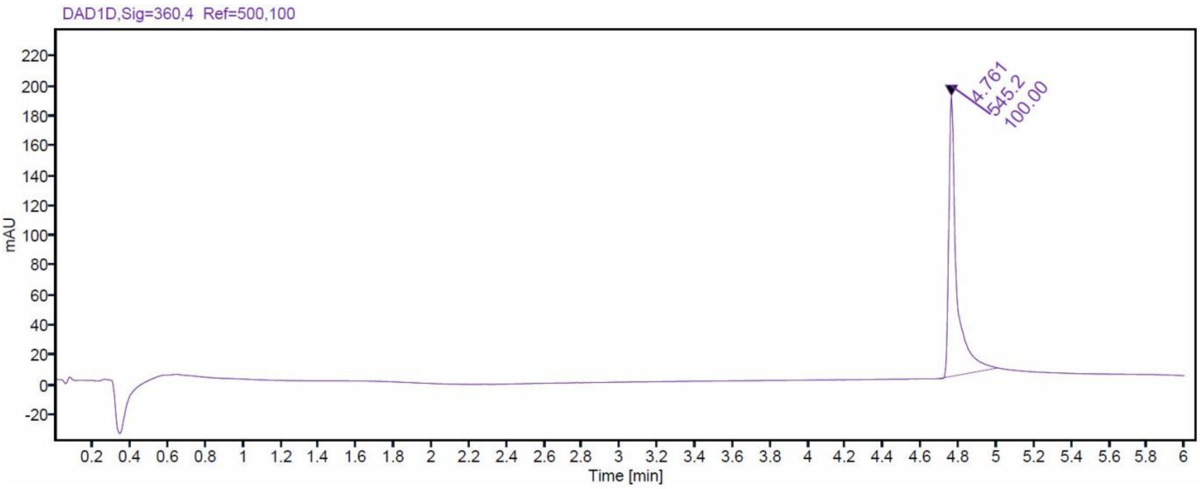

LC-MS Spectrum of **11**:

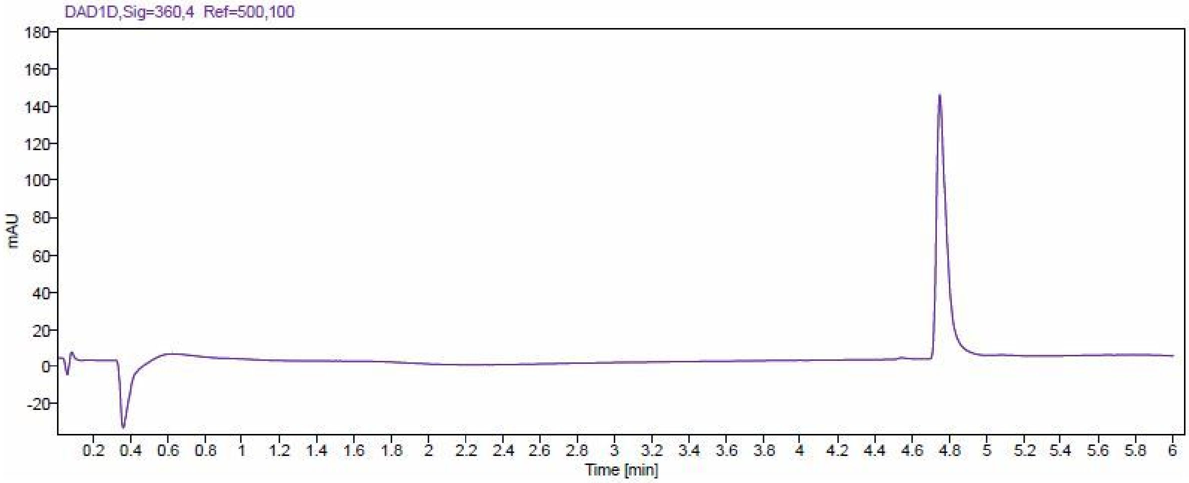

LC-MS Spectrum of **12**:

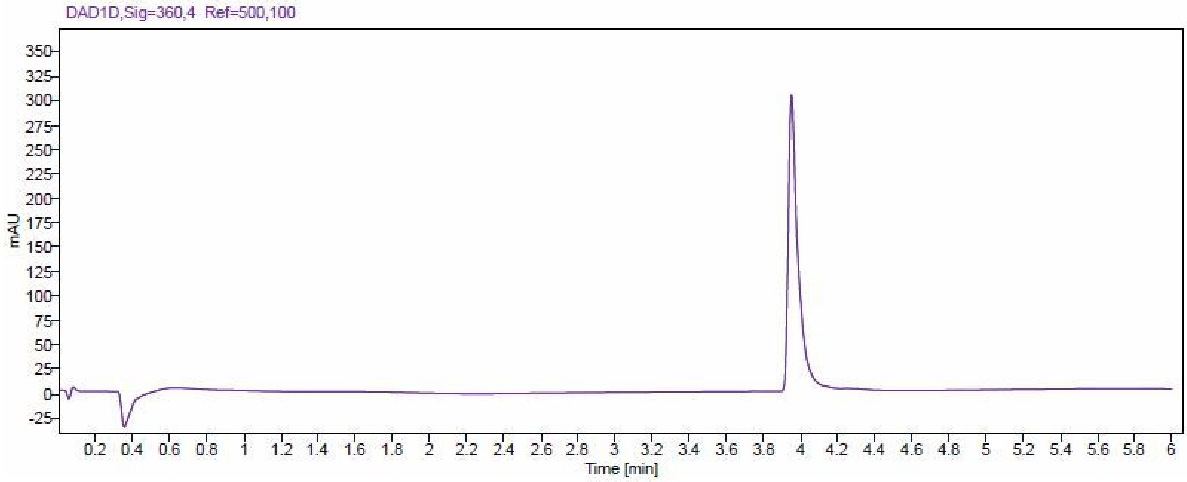

LC-MS Spectrum of **13**:

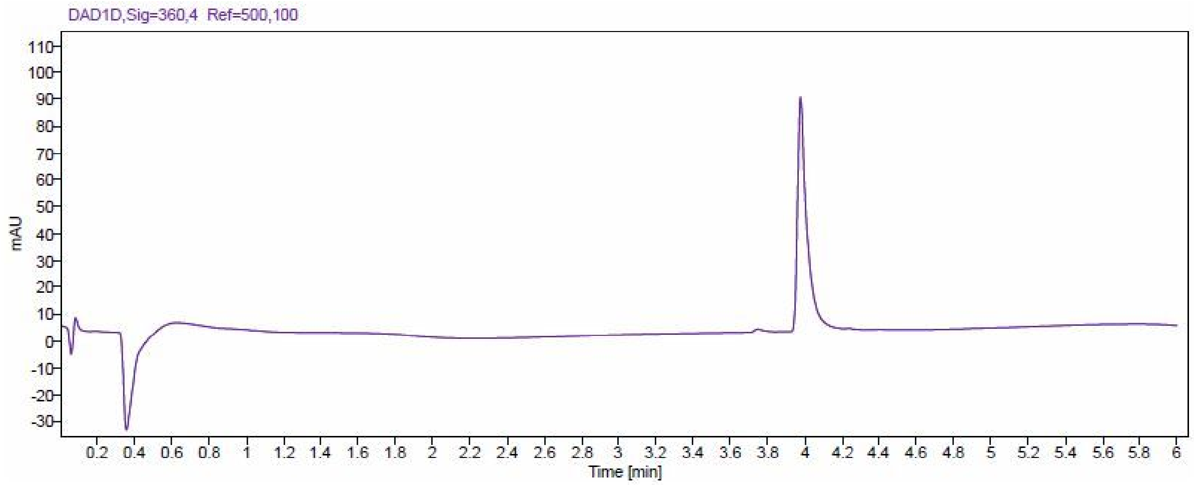

LC-MS Spectrum of **14**:

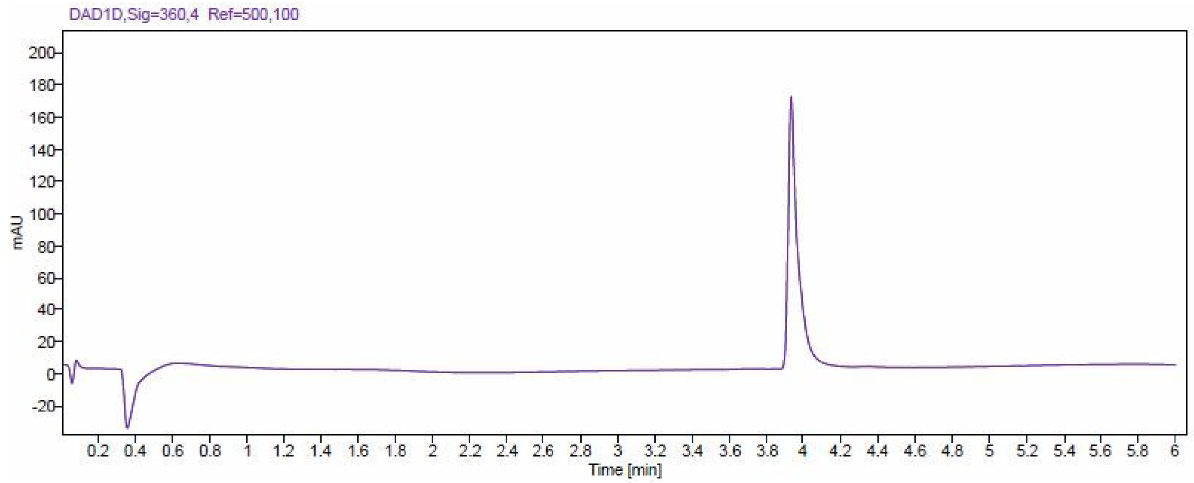

LC-MS Spectrum of **15**:

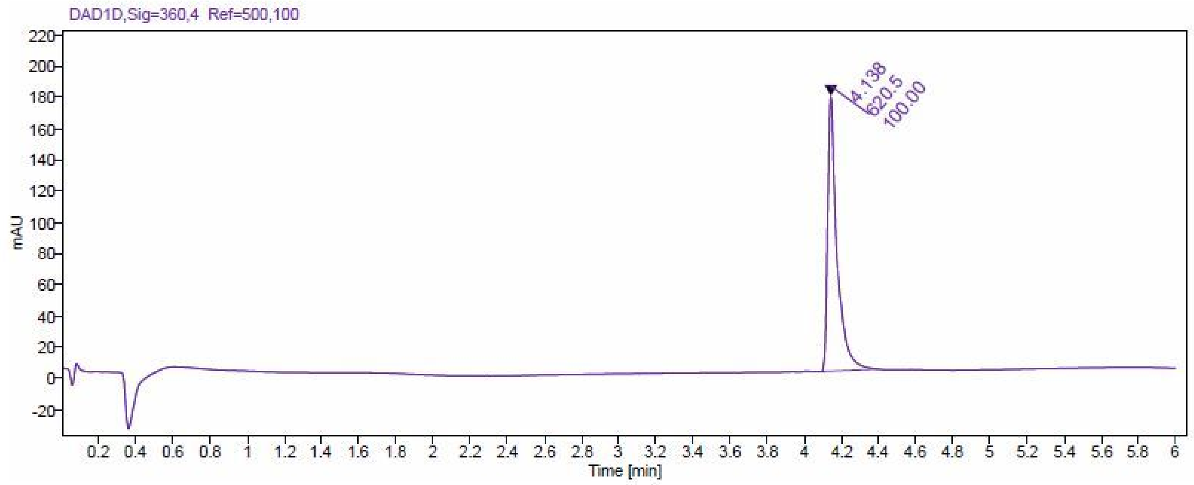

LC-MS Spectrum of **16**:

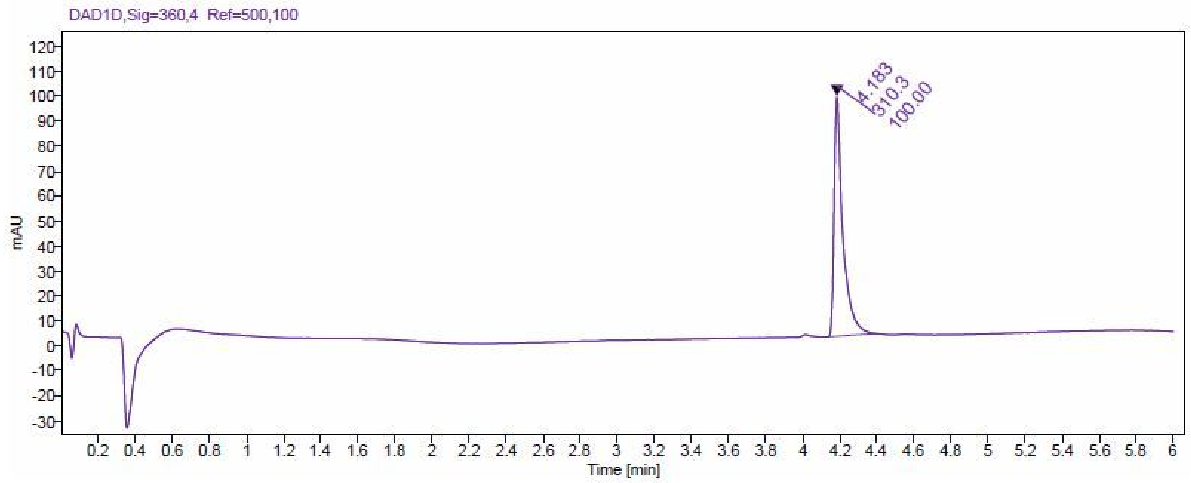

LC-MS Spectrum of **17**:

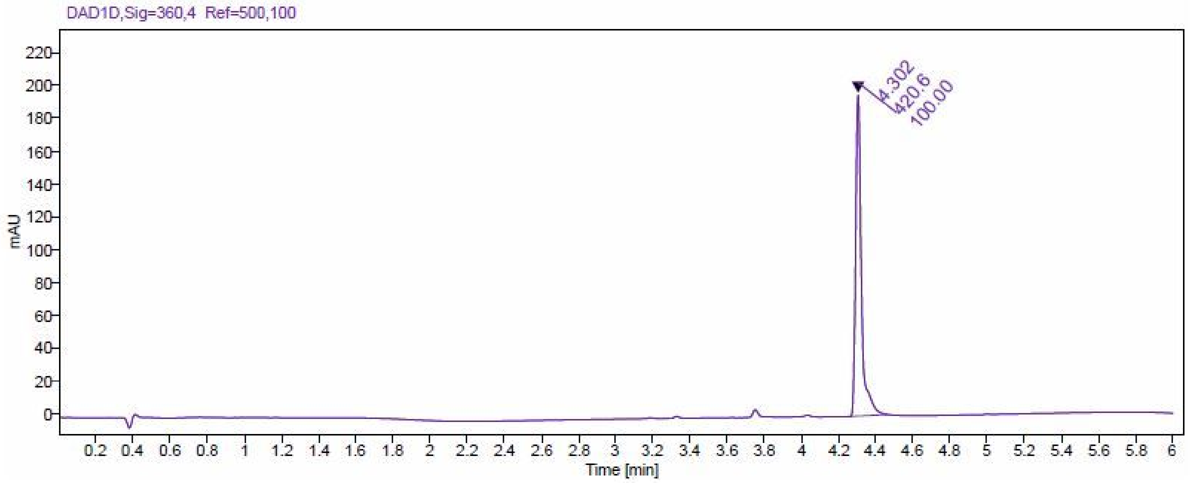

LC-MS Spectrum of **18**:

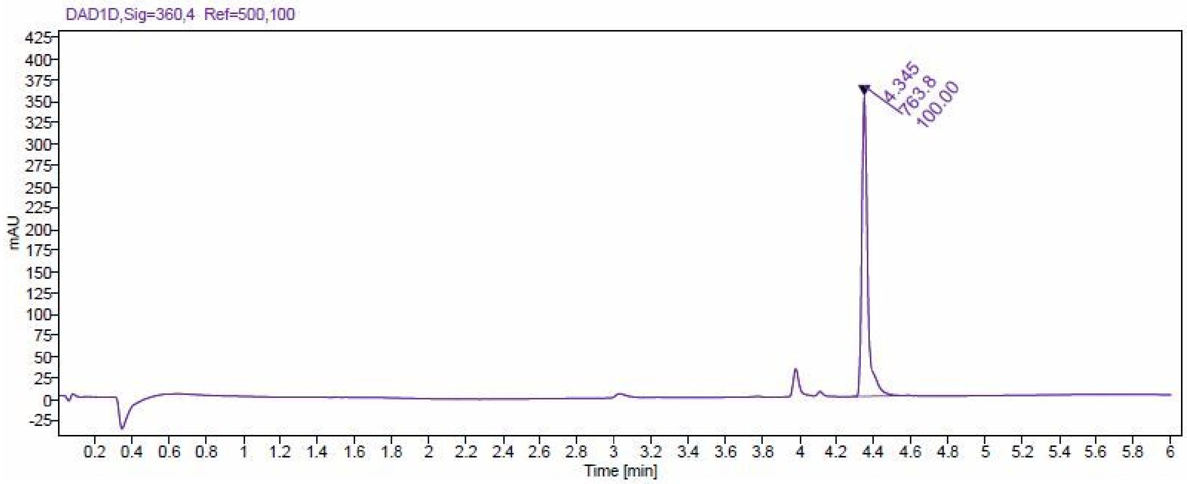

LC-MS Spectrum of **19**:

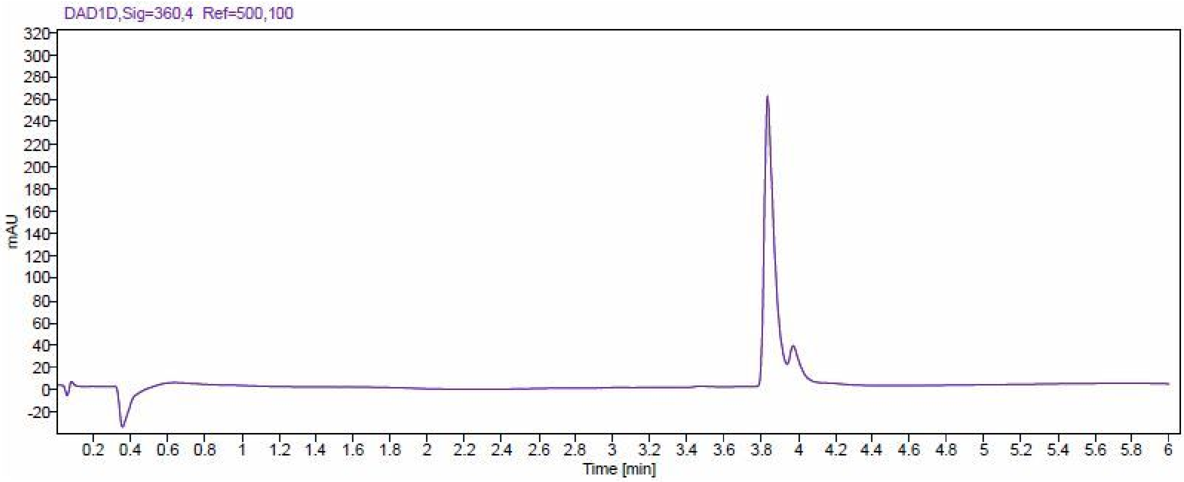

LC-MS Spectrum of **20**:

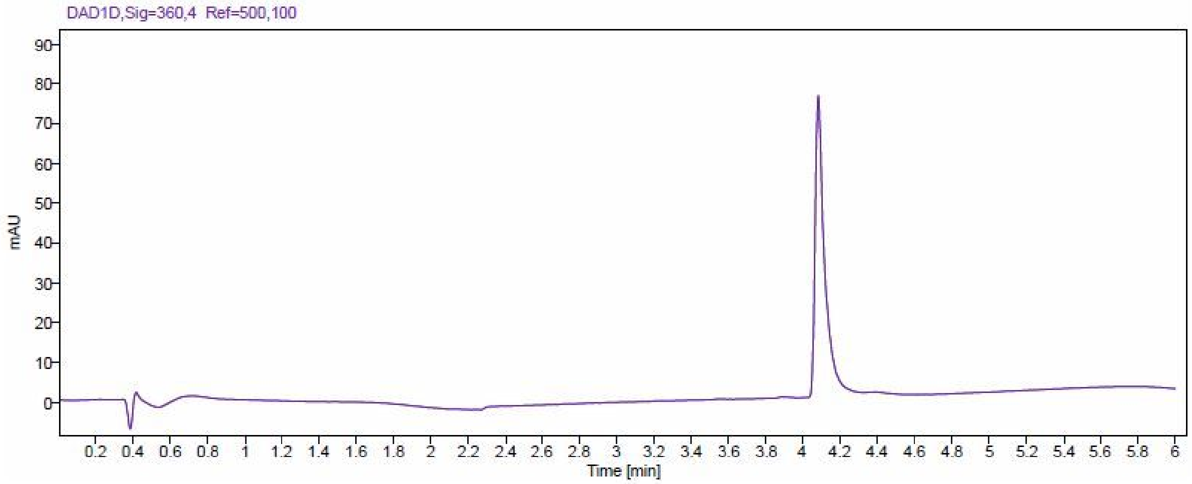

LC-MS Spectrum of **21**:

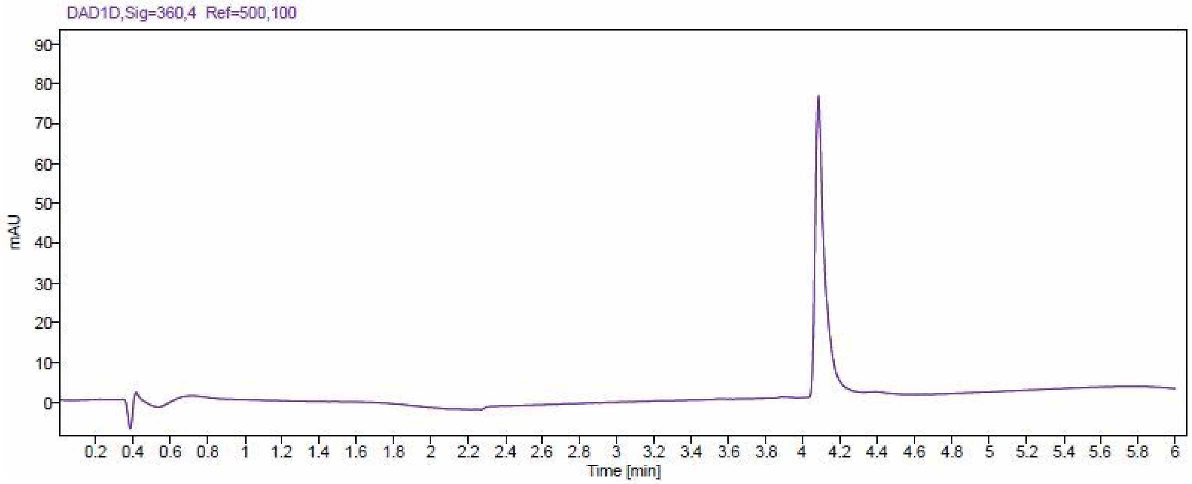

LC-MS Spectrum of **22**:

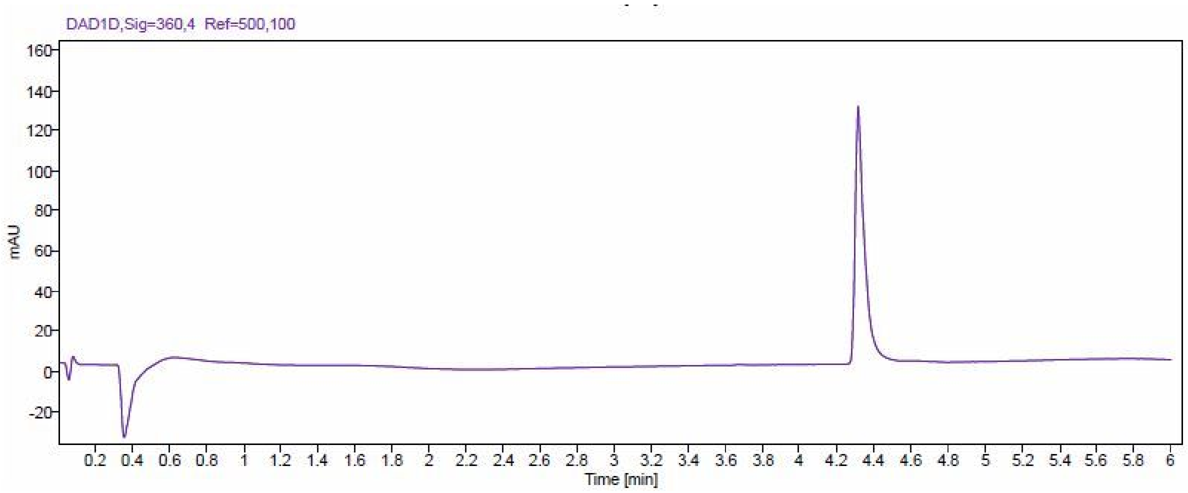

LC-MS Spectrum of **23**:

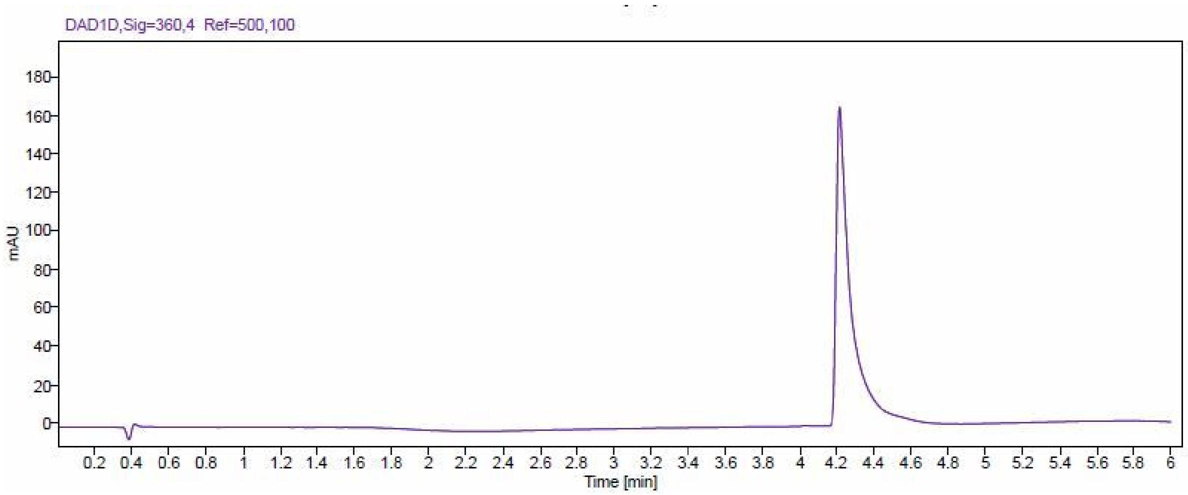

LC-MS Spectrum of **24**:

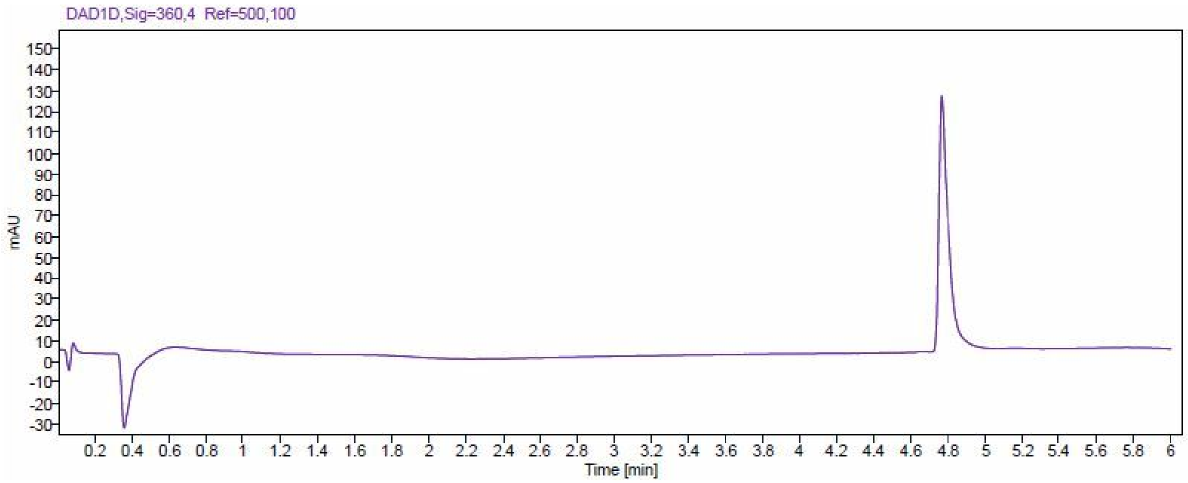

LC-MS Spectrum of **25**:

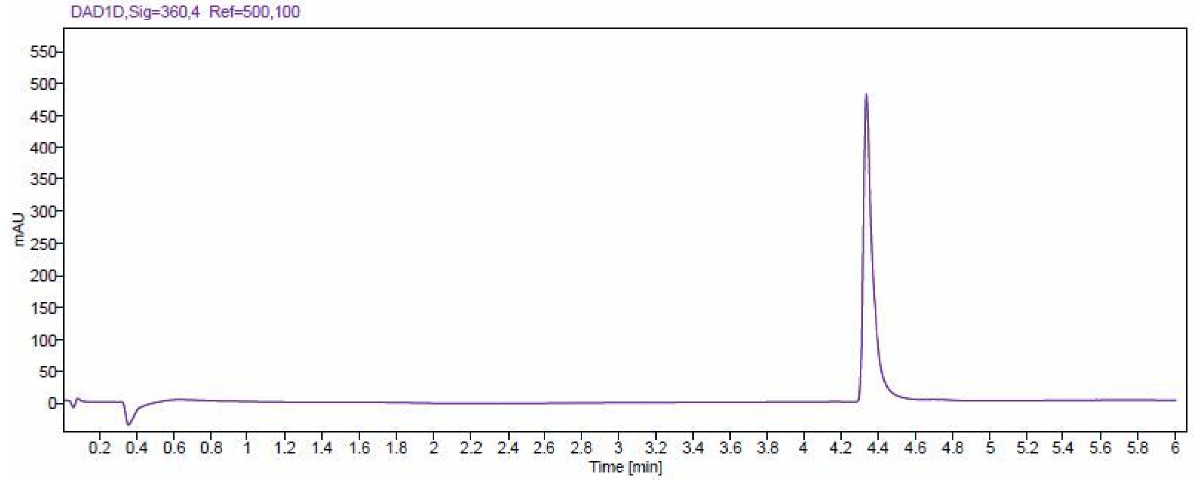

LC-MS Spectrum of **26**:

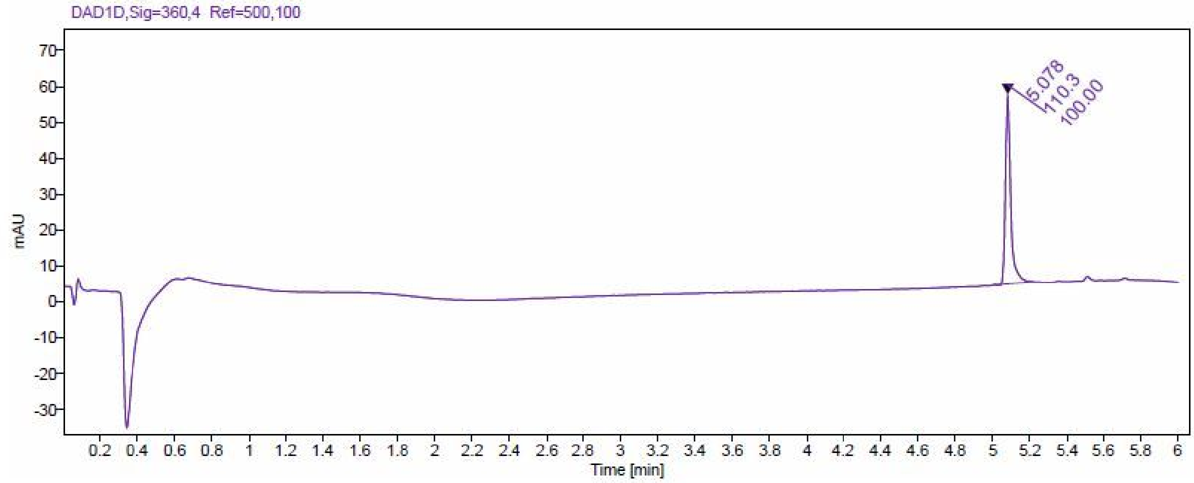

LC-MS Spectrum of **27**:

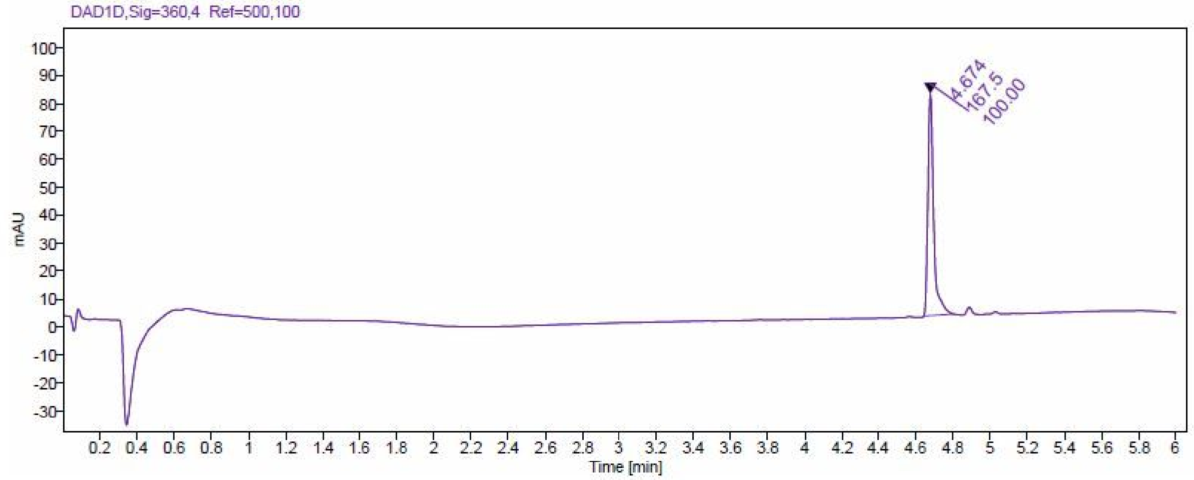

LC-MS Spectrum of **28**:

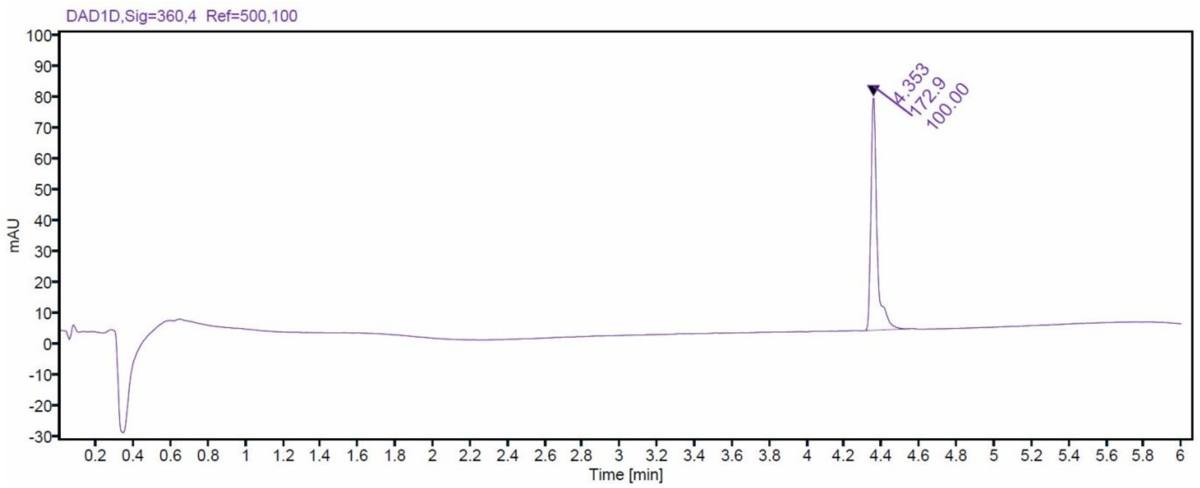

LC-MS Spectrum of **29**:

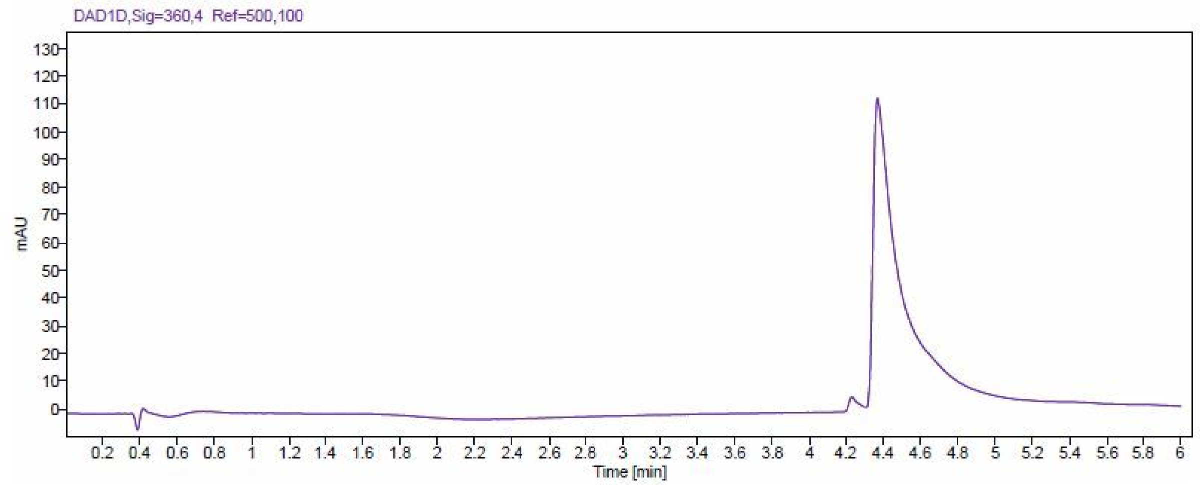

LC-MS Spectrum of **30**:

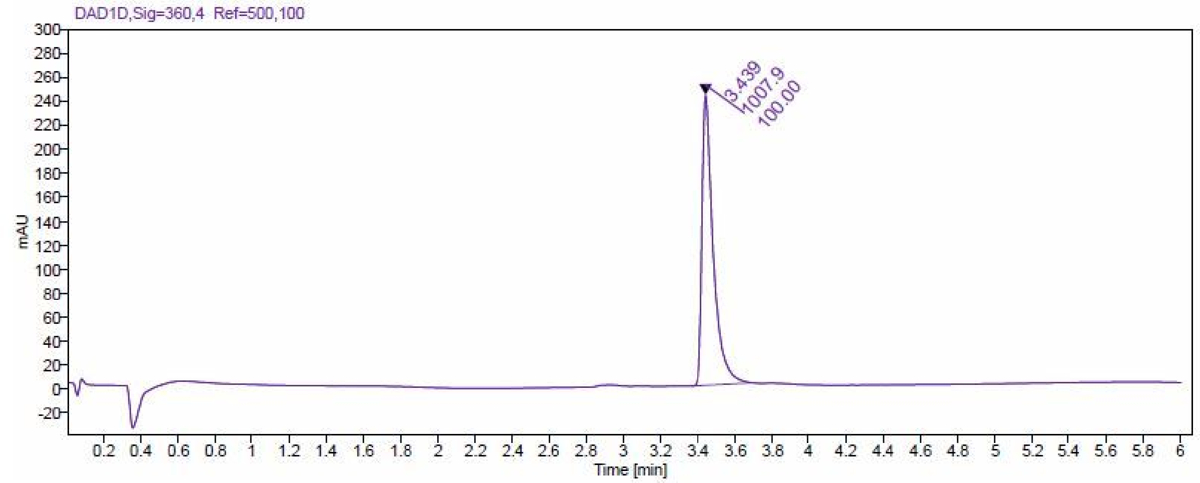

LC-MS Spectrum of **31**:

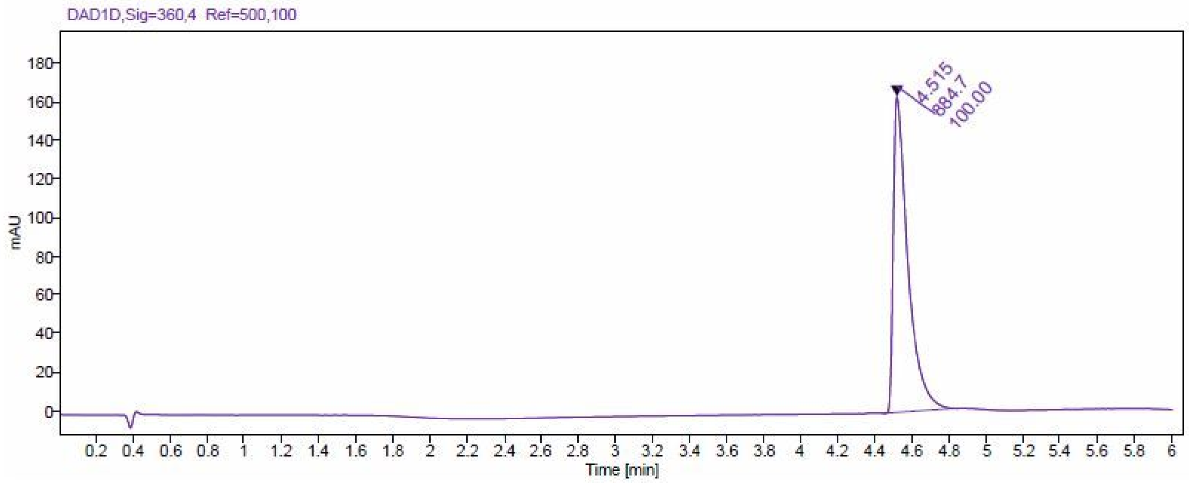

LC-MS Spectrum of **32**:

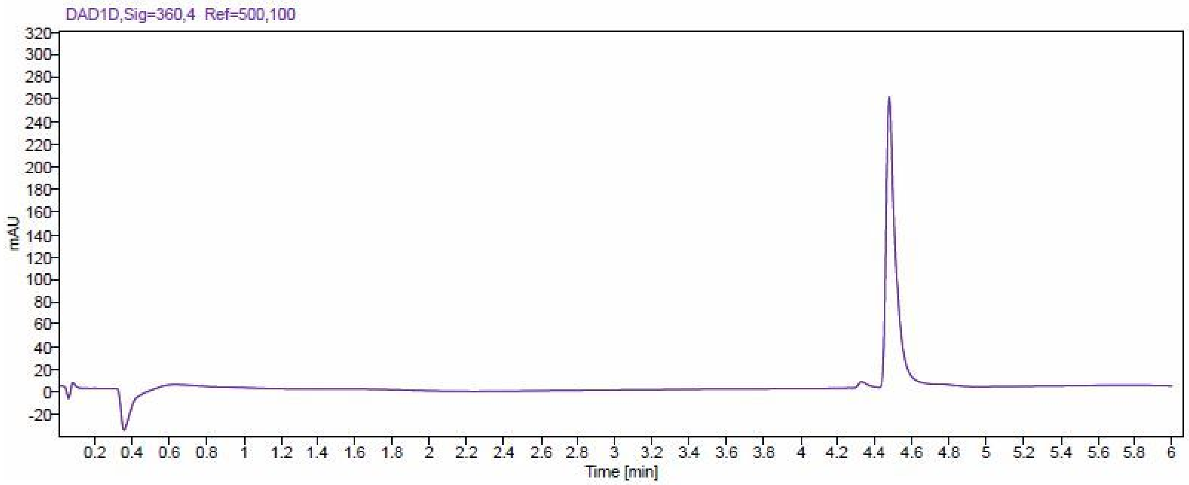

LC-MS Spectrum of **33**:

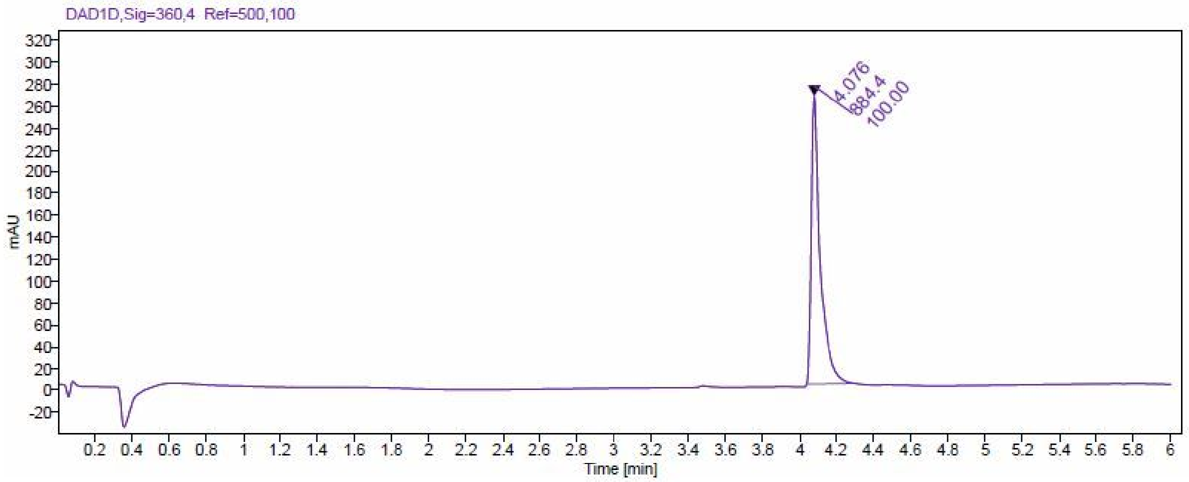

LC-MS Spectrum of **34**:

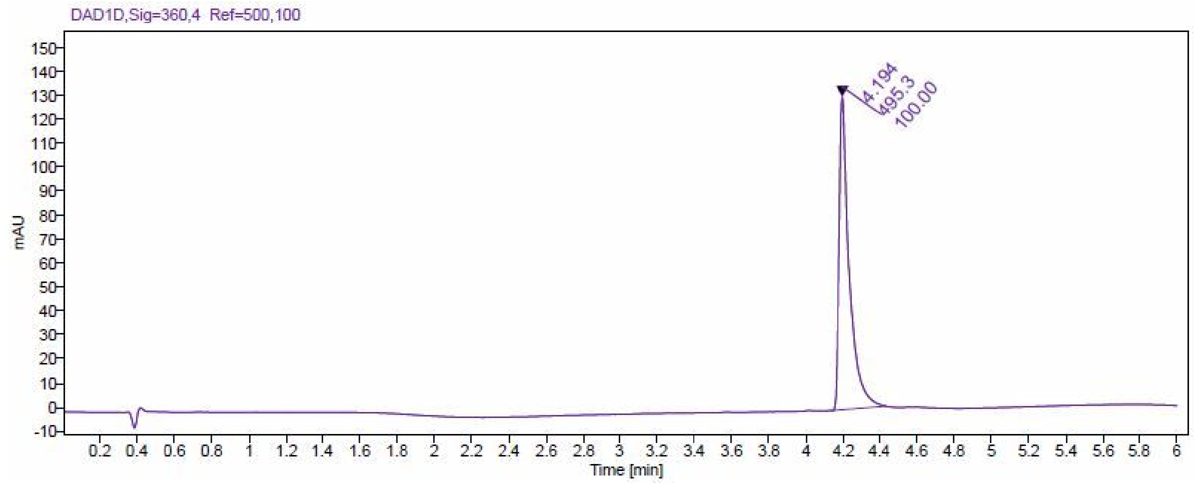

LC-MS Spectrum of **35**:

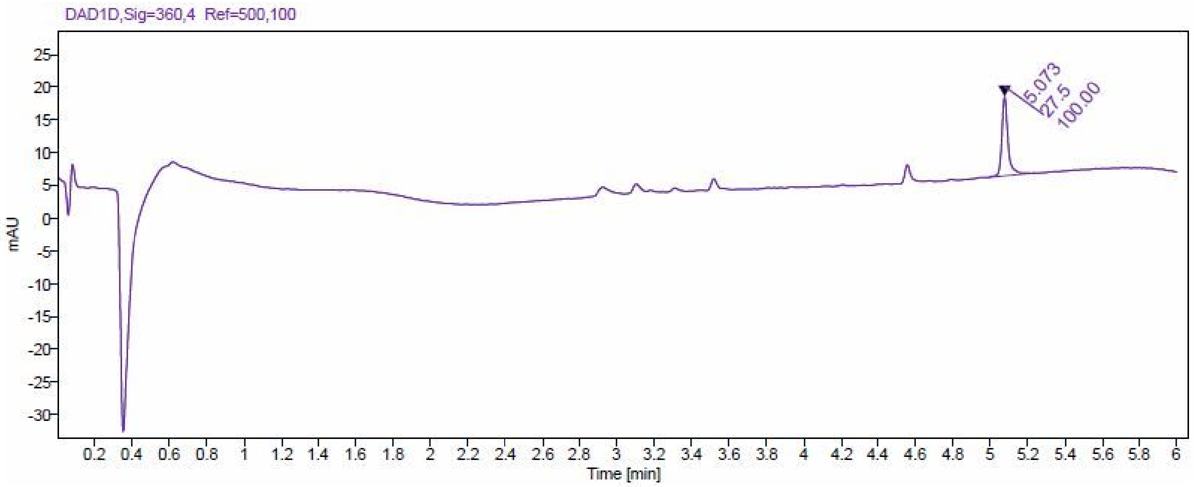

LC-MS Spectrum of **36**:

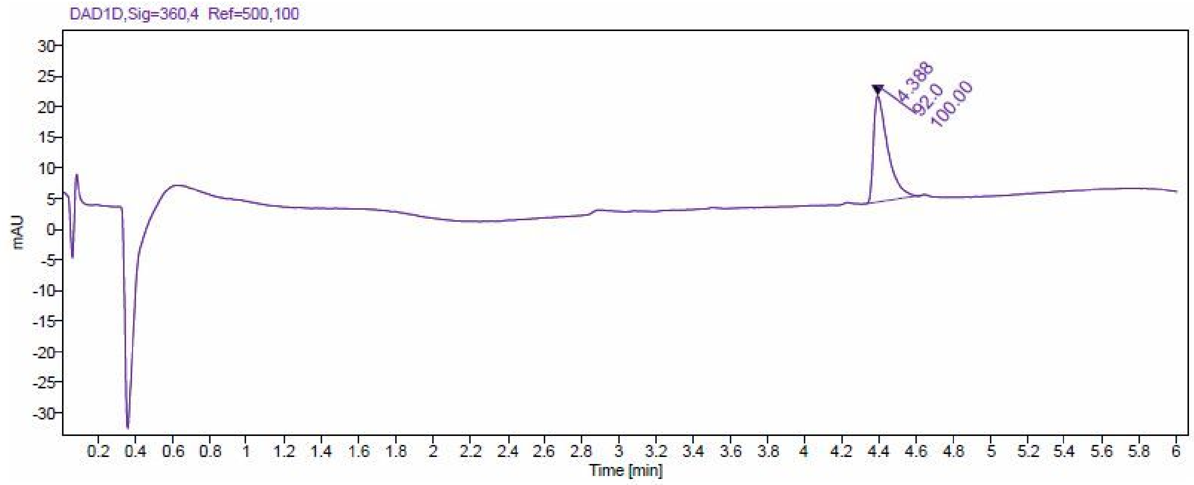

LC-MS Spectrum of **37**:

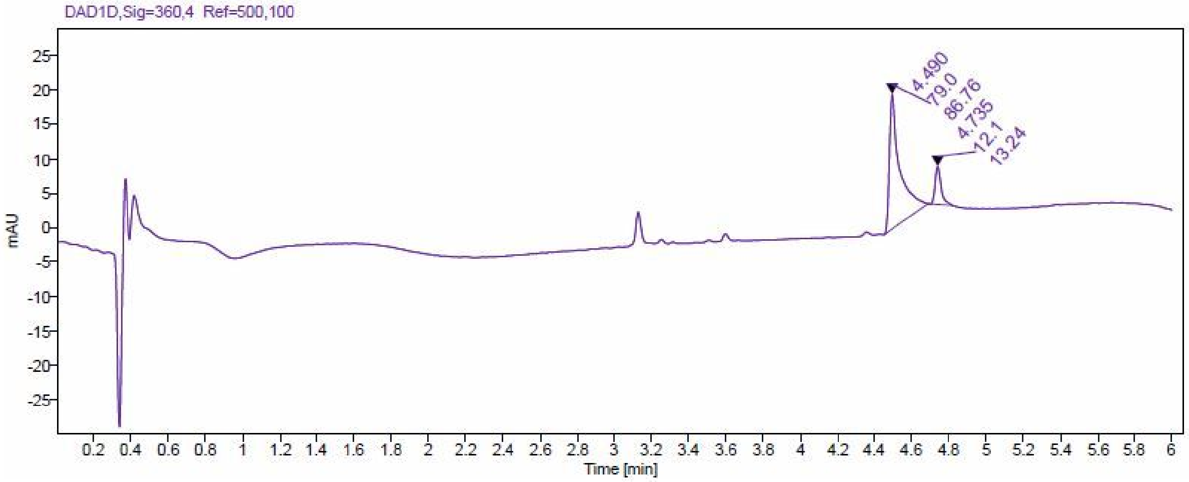

LC-MS Spectrum of **38**:

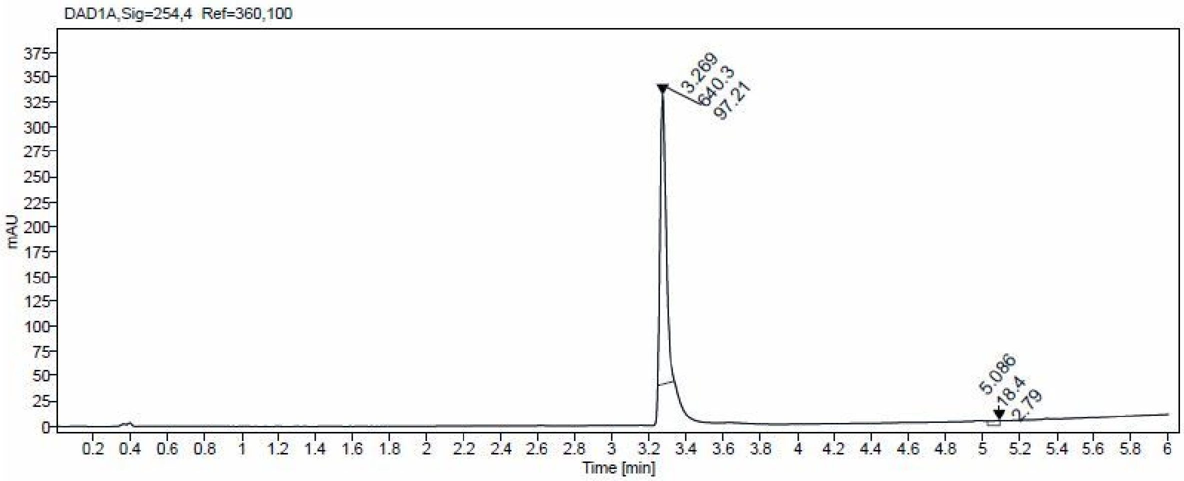

LC-MS Spectrum of **39**:

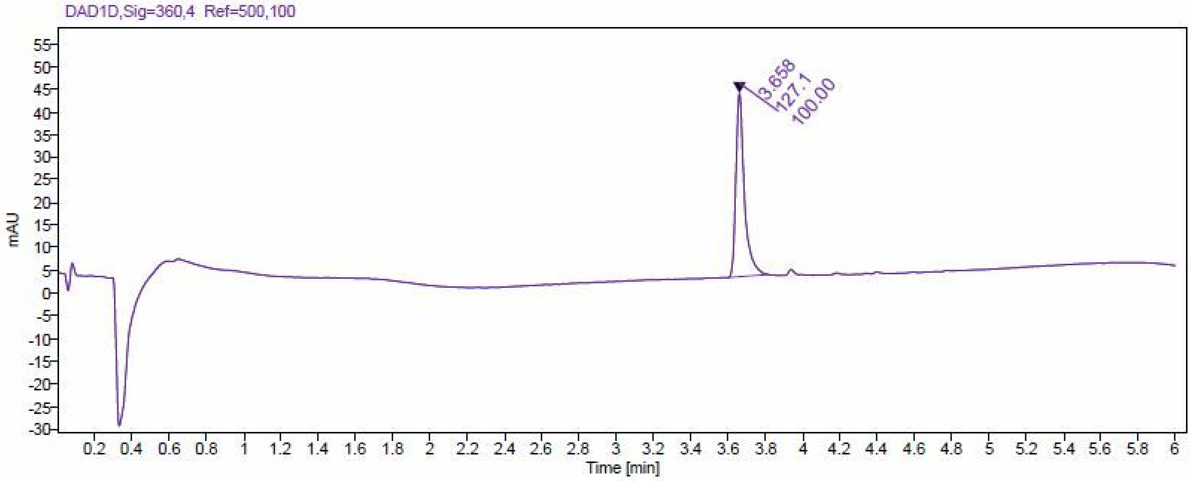

LC-MS Spectrum of **40** (*E/Z* mixture):

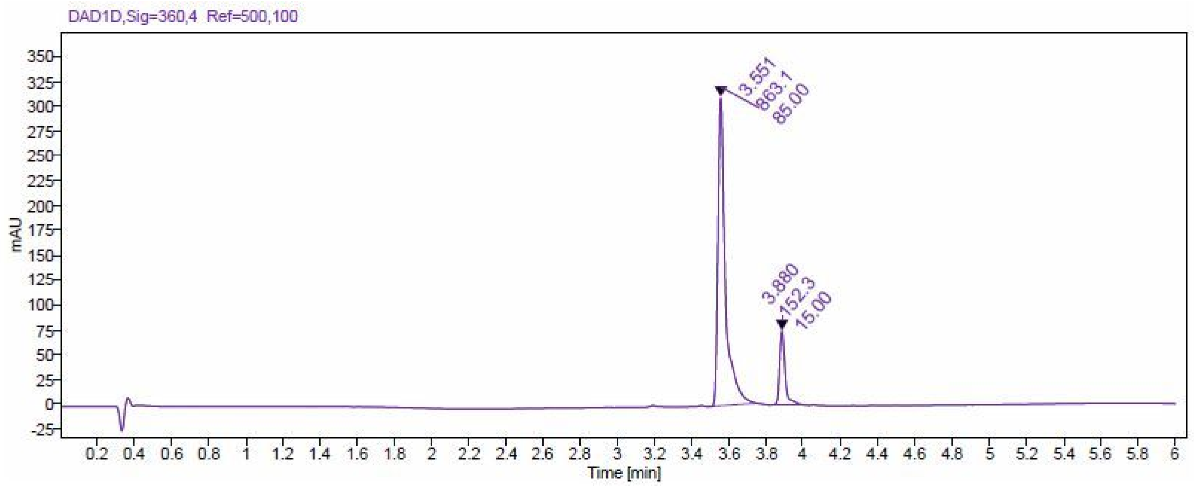

LC-MS Spectrum of **41** (*E/Z* mixture):

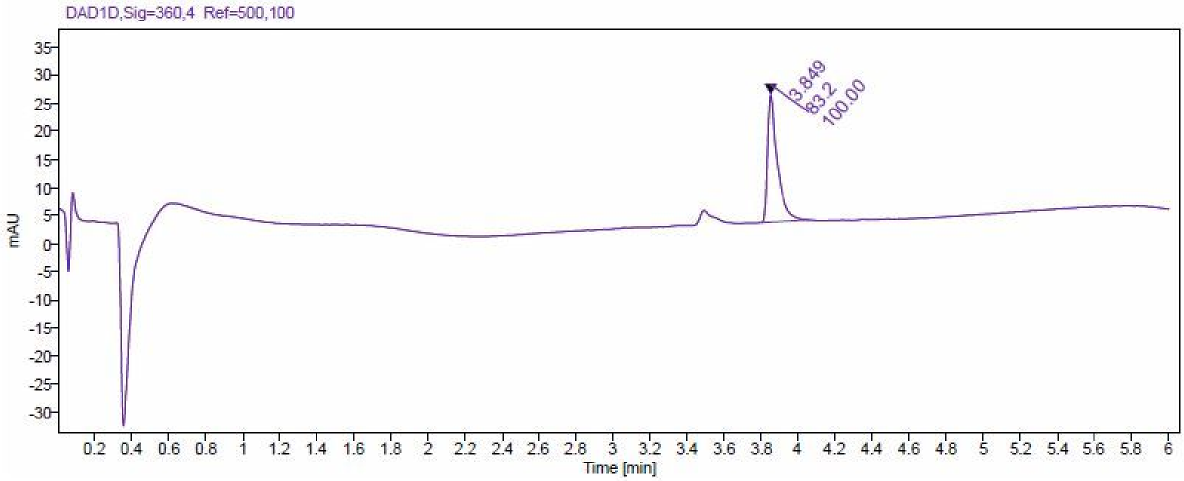

LC-MS Spectrum of **42**:

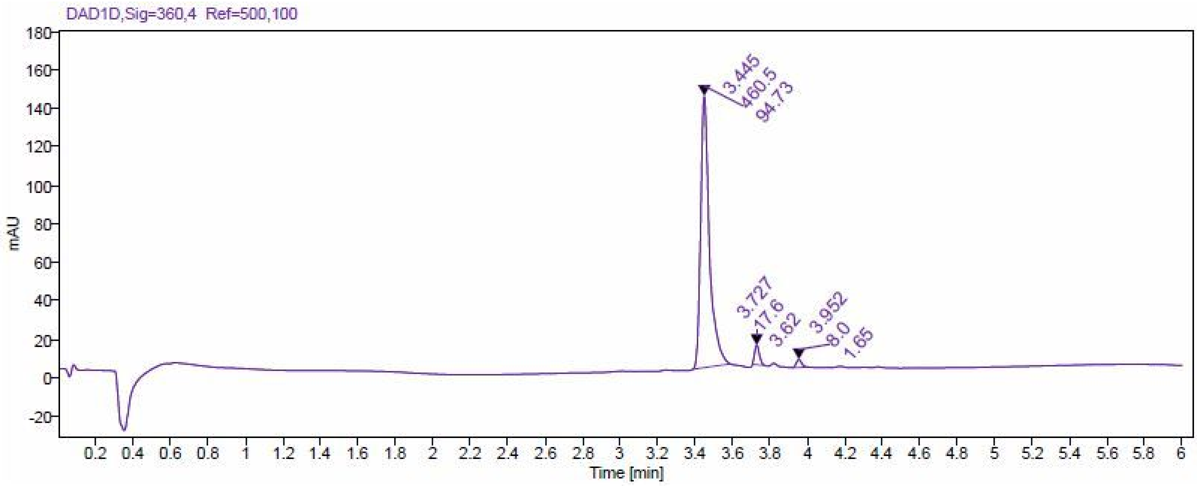

LC-MS Spectrum of **43** (*E/Z* mixture):

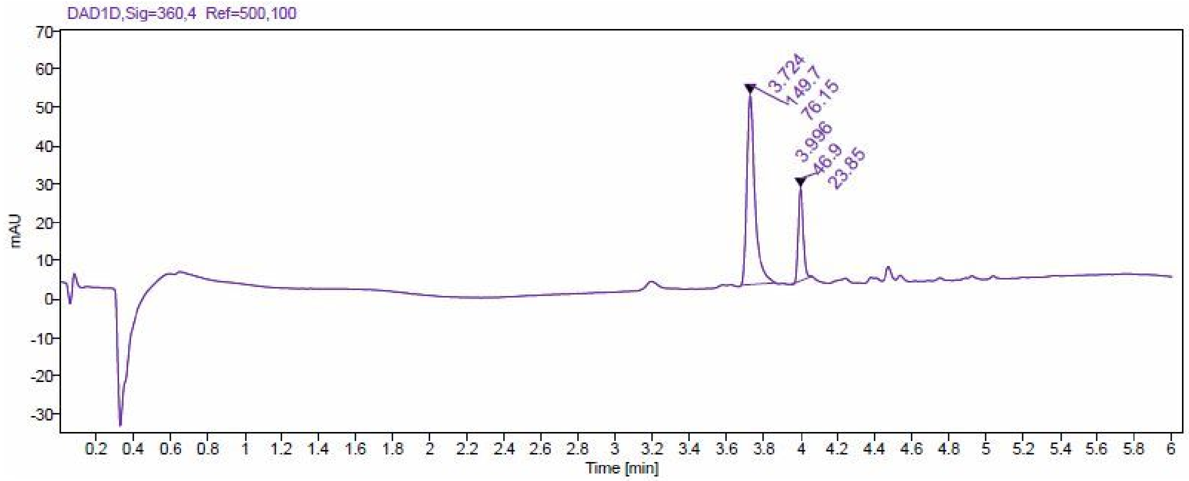

LC-MS Spectrum of **44** (*E/Z* mixture):

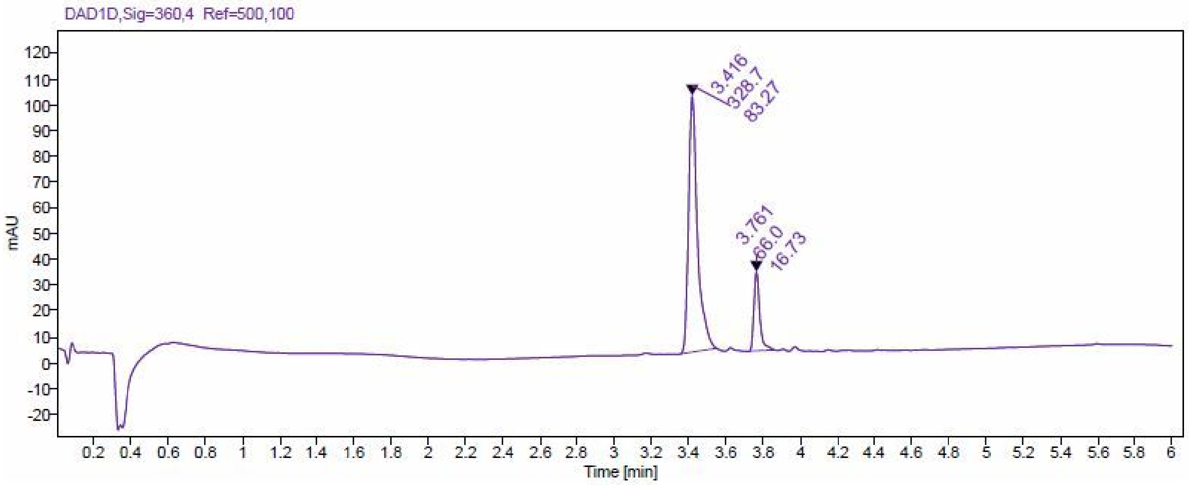

LC-MS Spectrum of **45**:

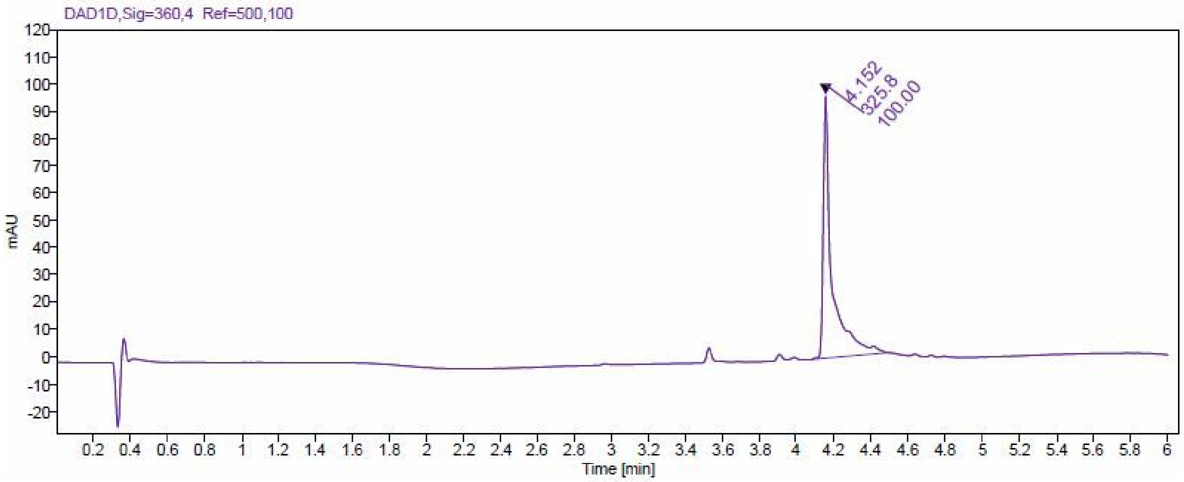

LC-MS Spectrum of **46**:

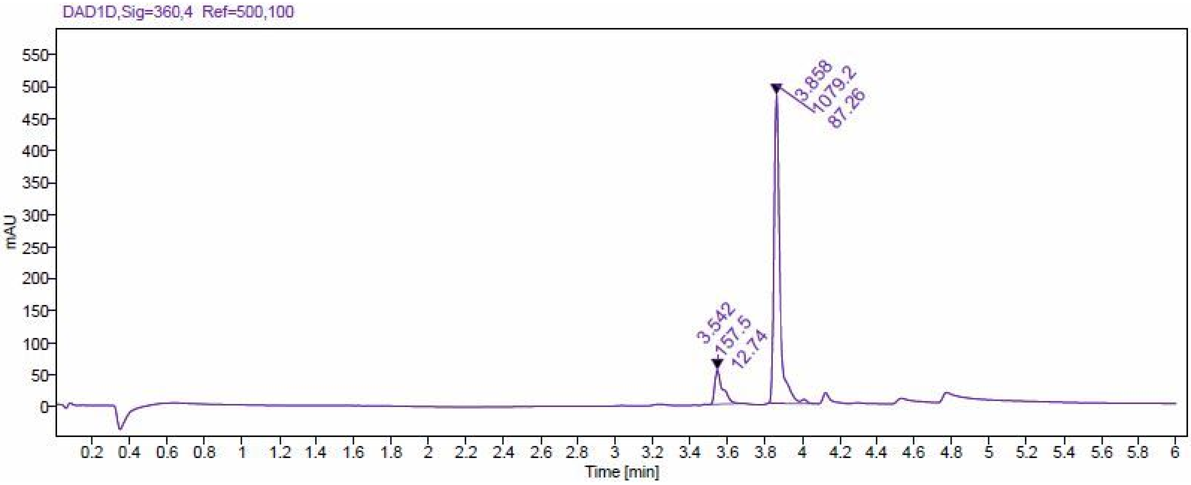

LC-MS Spectrum of **47**:

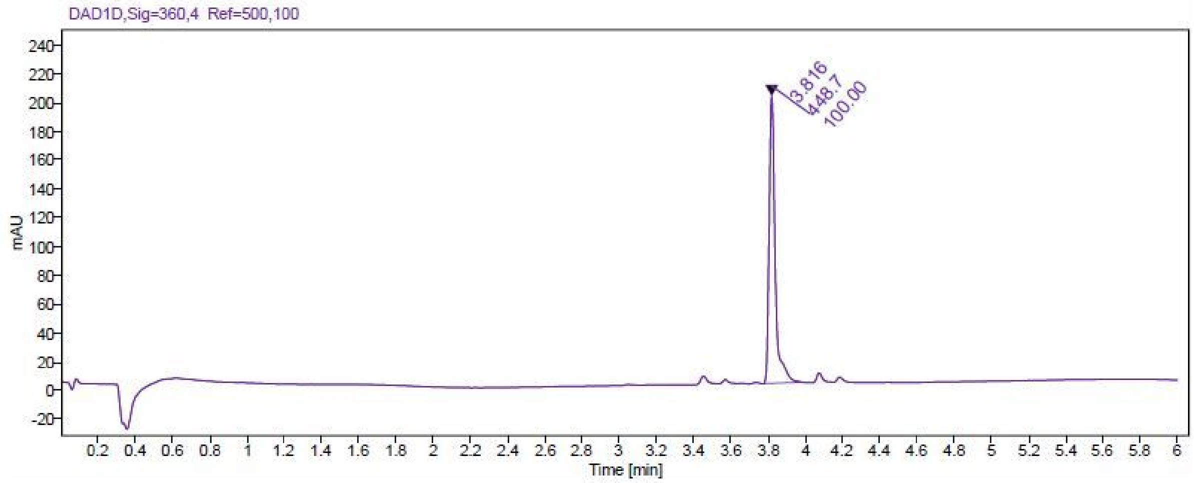

LC-MS Spectrum of **48**:

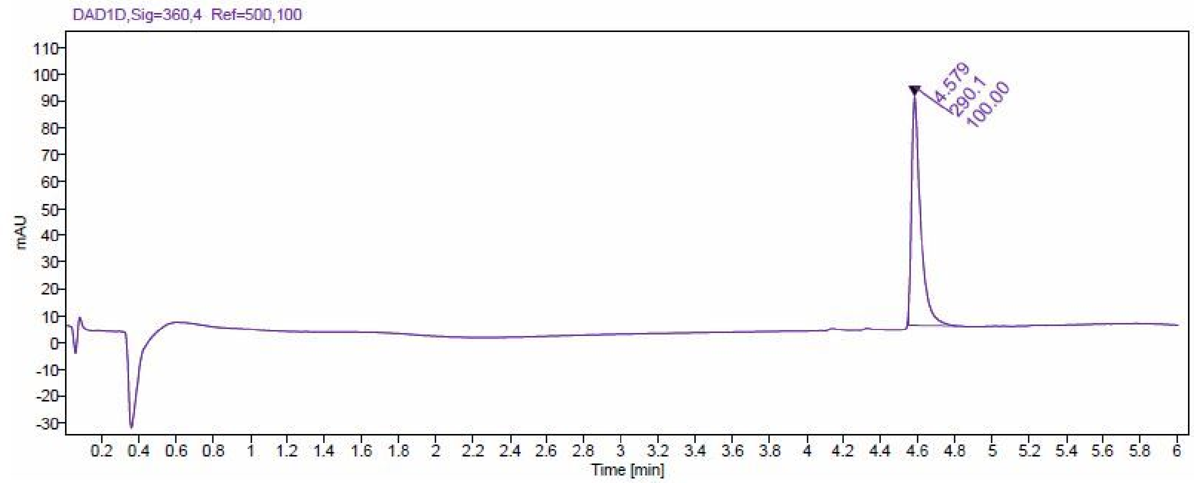

LC-MS Spectrum of **49**:

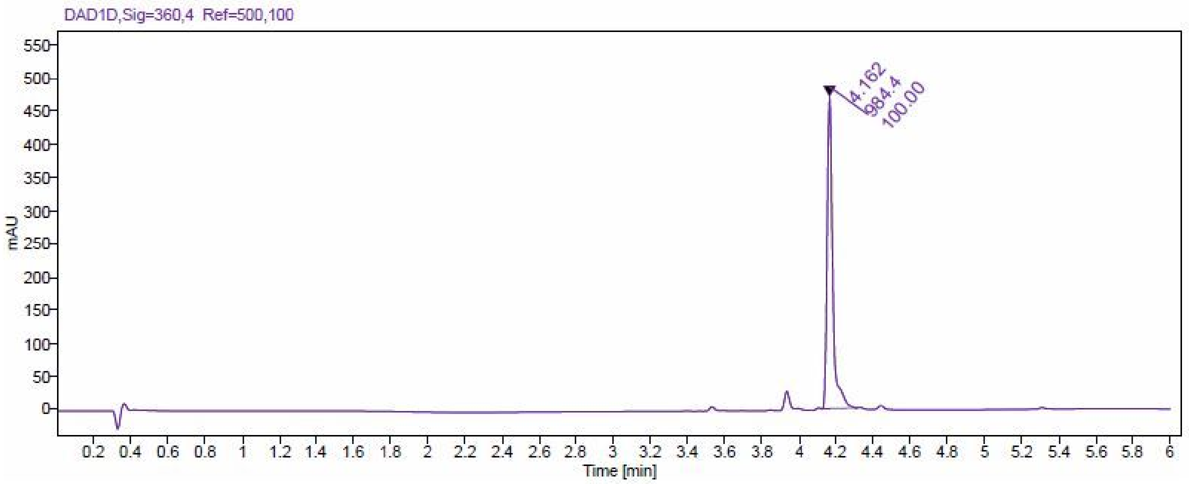

References

1. Andronescu, M.; Aguirre-Hernandez, R.; Condon, A.; Hoos, H. H., RNAsoft: A suite of RNA secondary structure prediction and design software tools. *Nucleic Acids Res* **2003,** *31* (13), 3416-22.

2. Ruszkowska, A.; Ruszkowski, M.; Dauter, Z.; Brown, J. A., Structural insights into the RNA methyltransferase domain of METTL16. *Sci. Rep.* **2018,** *8*, 5311.

3. Doxtader, K. A.; Wang, P.; Scarborough, A. M.; Seo, D.; Conrad, N. K.; Nam, Y., Structural Basis for Regulation of METTL16, an S-Adenosylmethionine Homeostasis Factor. *Mol. Cell* **2018,** *71* (6), 1001-1011.e4.

4. Robertson, M. J.; Deane, F. M.; Stahlschmidt, W.; von Kleist, L.; Haucke, V.; Robinson, P. J.; McCluskey, A., Synthesis of the Pitstop family of clathrin inhibitors. *Nat. Protoc.* **2014,** *9* (7), 1592-1606.
